# The anti-Alzheimer potential of novel spiroindolin-1,2-diazepine derivatives as targeted cholinesterase inhibitors with modified substituents

**DOI:** 10.1038/s41598-023-38236-0

**Published:** 2023-07-24

**Authors:** Hormoz Pourtaher, Alireza Hasaninejad, Shahrokh Zare, Nader Tanideh, Aida Iraji

**Affiliations:** 1 Department of Chemistry, Faculty of Sciences, Persian Gulf University, Bushehr, 75169 Iran; 2 Stem Cells Technology Research Center, Shiraz University of Medical Sciences, Shiraz, Iran; 3 Research Center for Traditional Medicine and History of Medicine, Department of Persian Medicine, School of Medicine, Shiraz University of Medical Sciences, Shiraz, Iran

**Keywords:** Chemical biology, Drug discovery

## Abstract

In this study, a new series of spiro indolin-1,2-diazepine were designed, synthesized, and screened for their cholinesterase inhibitory activities. A novel, green, high-yielding approach was constructed to synthesize spiro indolin-1,2-diazepine derivatives through a cascade reaction of different isatins, malononitrile and 1,1-enediamines (EDAMs) via sequential four-component reactions to produce the target compounds with good to excellent yields. Next the inhibitory potencies of all derivatives were determined spectroscopically at 415 nm using the modified Ellman method. The results of the in vitro screening indicated that **5l** with spiroindolin-1,2-diazepine core bearing 5-NO_2_ at R^1^ and 4-OH at R^2^ was the most potent and selective AChE inhibitor with an IC_50_ value of 3.98 ± 1.07 µM with no significant inhibition against BChE while **5j** was the most active analog against both AChE and BChE enzymes. The structure–activity relationships suggested the variation in the inhibitory activities of derivatives was affected by different substitutions on the indolinone ring as well as the phenyl moiety. The enzyme kinetic studies of the most potent compound **5l** at five different concentrations and acetylthiocholine substrate (0.1–1 mM) by Ellman's method revealed that it inhibited AChE in a mixed mode with a *K*_*i*_ of 0.044 μM. A molecular docking study was performed via induced fit docking protocol to predict the putative binding interaction. It was shown that the moieties used in the initial structure design play a fundamental role in interacting with the enzyme's binding site. Further, molecular dynamics simulations with the Schrödinger package were performed for **5l** in a complex with AChE and revealed that compound **5l** formed the stable complex with the enzyme. The MTT toxicity assessments against the neuroblastoma cell line were executed, and no toxicity was seen for **5l** under the tested concentrations.

## Introduction

Alzheimer's disease (AD) is a progressive neurodegenerative disorder and the most common form of dementia worldwide, characterized by greater memory loss, huge psychological dysfunctions, and other cognitive difficulties^1,2^. The pathogenesis of AD is still unclear; however, several reasons such as abnormal extracellular deposition of misfolded amyloid-β (Aβ) protein, intracellular accumulation of hyperphosphorylated tau proteins as neurofibrillary tangles (NFT), metal ion dyshomeostasis, and inflammation were proposed^3,4^. Also it was shown that amyloid-β (Aβ) contributes to free radical production and may cause neurodegenerative diseases^5,6^.

Also, it has been reported that loss and dysfunctions of cholinergic transmission and reduction of acetylcholine neurotransmitters are the major molecular hallmarks of AD^3,7^. Acetylcholinesterase (AChE) enzyme is involved in the hydrolysis of the neurotransmitter acetylcholine to generate acetic acid and choline, leading to the shortening of the duration of acetylcholine in the hippocampus and cortex of the brain^8^. The increase in butyrylcholinesterase (BChE) was seen at the late stage of AD, perhaps to compensate the reduction of AChE to hydrolysis acetylcholine^9^. As a result of AChE and BChE hydrolysis effects, shortage of the acetylcholine duration in the hippocampus and cortex of the brain is related to AD psychological dysfunctions. The FDA-approved drugs for AD are donepezil, galantamine, rivastigmine, and tacrine aimed to inhibit the AChE at the initial stage of disease, maintaining a balanced acetylcholine level in CNS^10^. Therefore, the limitation of the available effective therapeutic agents has attracted life science researchers to develop novel drug candidates to target AD. With the gradual progression of the disease, the routinely used drugs may not be effective. Therefore, cholinesterase inhibitors that enhance cholinergic transmission can be used as a remedy for AD. Also, there are some reports exhibiting Aβ-ChE inhibitors^11,12^, BACE1-ChE inhibitors and ChE and α-glucosidase inhibitors^13,14^.

**Figure 1:**
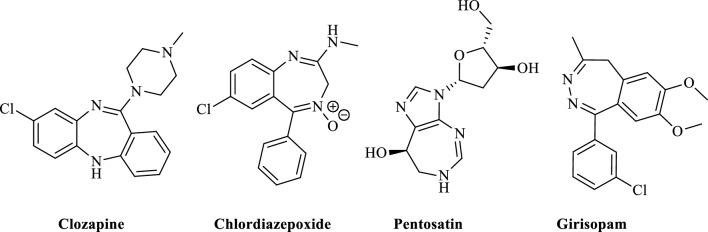
Representative examples of biologically active diazepine derivatives.

Diazepines are an important group of seven-membered heterocyclic compounds with two nitrogen atoms, which form the main active pharmaceutical compounds with various applications in medicinal chemistry^15–17^. Highly substituted diazepines with active functional groups are very interesting compounds due to their additional pharmacological properties^18–20^. Some biologically active diazepines are shown in Fig. 1. Diazepinone specifically comprises a whole class of drugs, including the anxiolytic drug tofisopam^21,22^. Diazepine derivatives have been used as progesterone receptor antagonists and in dealing with epilepsy and gliomas^23,24^. They show varied biological activities such as antiproliferative^25^, anticancer^26^, and anticonvulsant^27^.

Oxindoles are an important family of heterocyclic compounds that represent important building blocks^28,29^ in a wide range of several drugs and natural compounds such as Horsfiline, Coerulescine, Spirotryprostatin A, Welwitindolinone A, Elacomine and Alstonisine. Synthesis of spirooxindoles is of great interest to many organic chemists because these compounds are well-known as microtubule assembly inhibitors (Spirotryprostatin A and B)^30^, serotonin receptor modulators (Isopteropodine and Pteropodine)^31^, Muscarinic M1, and nonpeptidyl growth-hormone secretagogues (MK-0677^32^. Considerable attention has been focused on the development of the synthesis of novel spiroxindole ring systems.

Green chemistry is about developing processes and technologies that lead to more efficient chemical reactions that produce less waste and less environmental emissions than traditional chemical reactions that decrease the negative effects on human health and the environment^33,34^. Mainly, using solvents is a steady source of worry because it gives rise to toxicity, pollution, hazard, and waste treatment issues. As a result, many efforts have been made to find stable reaction environments, especially non-toxic solvents such as water and or ethanol, which have attracted much attention in recent years. Therefore, the design of new multicomponent reactions (MCRs) using green and environmentally compatible solvents has attracted the attention of drug discovery and organic synthesis researchers.

As a result, novel series of novel spiro indolin-1,2-diazepine were designed as AChE and BChE inhibitors. In this context, new methodologies to synthesize novel spiro indolin-1,2-diazepine systems were developed, and the structures of all derivatives were confirmed using different spectroscopical techniques. Next, the inhibitory potential of all derivatives was examined against AChE and BChE. Furthermore, the kinetic study, molecular docking, and molecular dynamic of the most potent compound were performed to get insight into its behavior against enzymes. Also, the neurotoxicity of the best ChE inhibitors was examined against the SH-SY5Y neuroblastoma cell line.

## Result and discussion

### Designing

**Figure 2:**
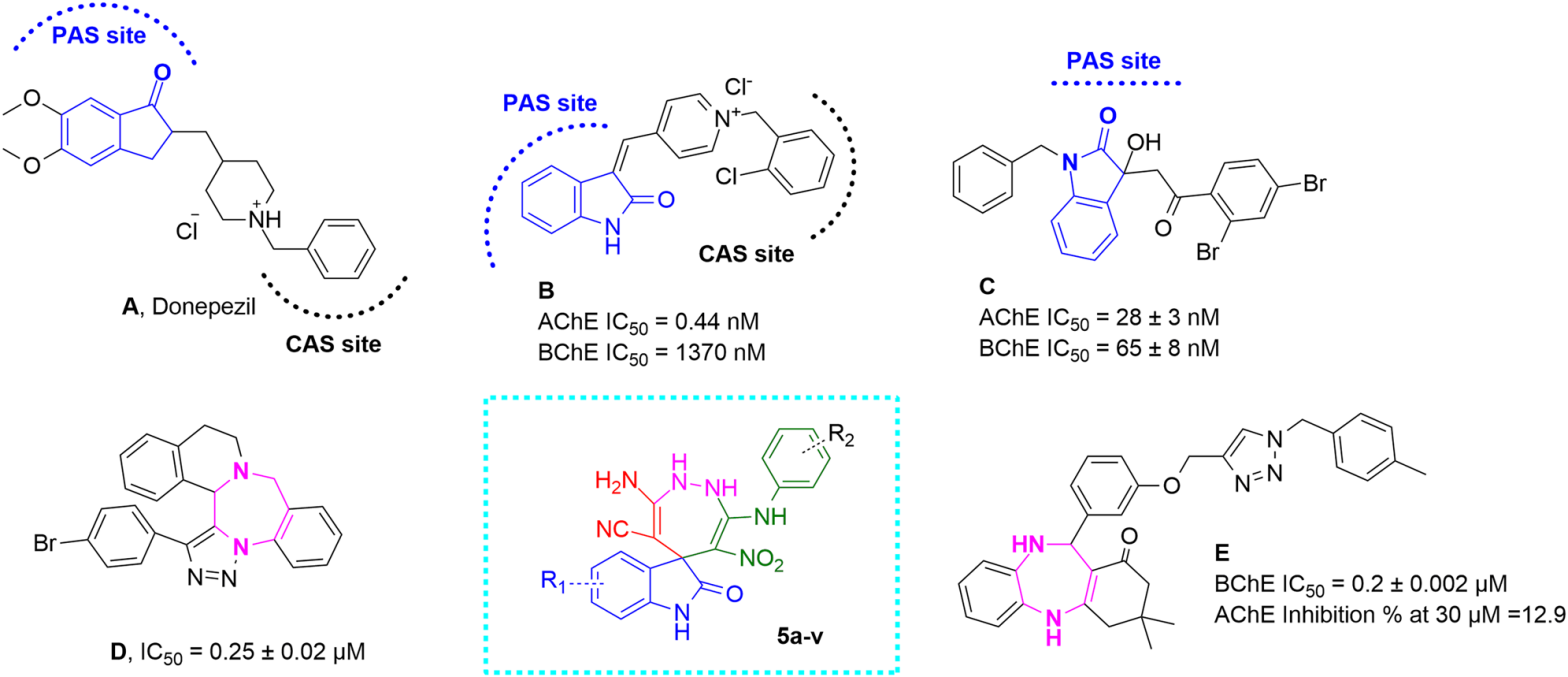
Previously reported ChE inhibitors (**A–E**) and newly designed compound.

Regarding the X-ray crystallographic structure of the AChE, the peripheral anionic site (PAS) at the gorge's entrance comprises Tyr70, Asp72, Tyr121, Trp279, and Tyr334. The catalytic activity site (CAS) of AChE at the bottom of the gorge consists of two sub-units the catalytic triad of the active site, including Ser200, His440, and Glu327, and the catalytic anionic site at the vicinity of the catalytic triad consisting of Trp84, Tyr130, Gly199, His441, and His444^9^. In comparison, BChE active site is larger than AChE and usually tolerates bigger scaffolds than AChE. Regarding the active site of the enzyme, different inhibitors that target CAS or PAS or both pockets were developed. Donepezil (Fig. 2) was introduced as a reference AChE inhibitor, which mimics the binding mode of the ACh neurotransmitter by structural similarity in competitive mode^35^. In the following, several analogs of donepezil were reported as potent ChE inhibitors in which indanone moiety was bioestically replaced with features similar to mentioned ring, such as indole, and indolinone. Indolinone-based compounds bearing benzylpyridinium moiety were designed as dual-binding inhibitors of AChE, and the most potent derivative (compound **B,** Fig. 2) exhibited 32-fold more potent than donepezil as a reference drug^36^. In another study, different oxindole derivatives were designed and exhibited promising potencies against AChE and BchE (compound **C**)^37^.

Fused^1–3^triazolo[1,4]diazepines (compound **D**) were synthesized as possible anti-AD agents, and the most potent analog exhibited good AChE inhibition and BBB penetration^38^. Also, in another study, benzodiazepine-1,2,3-triazole derivatives were synthesized and evaluated as cholinesterase inhibitors (compound **E**). These derivatives exhibited selective inhibitory activities against BChE with an IC_50_ value of 0.2–17.3 µM^39^.

Drug design hybrid strategy combines two or more biologically active molecules into a new molecule that possesses the therapeutic potential of combined derivatives. Thus, we focused on a well-established molecular hybridization strategy incorporating spiro indolinone into diazepine derivatives. Indolinone is categorized as a critical pharmacophore to occupy the ChE pockets and diazepine, as an N-containing ring, could be effective for the interactions with the residues of ChE active site (Fig. 2). Amidic, C = N, or nitro substituents, are key functionalities that participate in H-bound interactions with the active site residues of the ChE enzymes. Next, a new MCR synthetic strategy was developed for the efficient synthesis of novel indolin-1,2-diazepine as AChE and BChE inhibitors. Furthermore, the kinetic studies of the most potent derivative were performed. The most potent compound was then subjected to molecular docking and molecular dynamic (MD) studies to evaluate its binding affinity and mode of action within the enzyme's binding site. Finally, the toxicity of the most potent derivative was performed against the neuroblastoma cell line.

### Chemistry

**Scheme 1.**
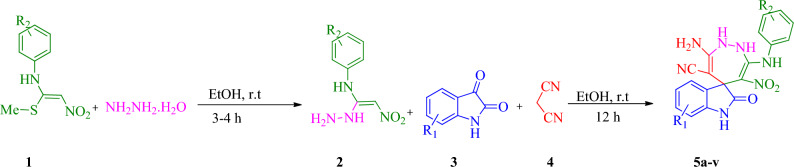
One-pot sequential four-component synthesis of highly substituted spiro indolin-1,2-diazepine derivatives.

A one-pot, sequential four-component synthesis of substituted spiro indolin-1,2-diazepine derivatives is exhibited in Scheme 1. Initially, N-aryl-1-(methylthio)-2-nitroethenamine **1** (1 mmol) and NH_2_NH_2_ (80% aq) (1.2 mmol) were reacted in ethanol (5 mL) at room temperature to form EDAM **2**, after 3-4 h isatin **3** (1 mmol) and malononitrile **4** (1 mmol) were added to obtain the desired product **5**. It should be mentioned the sequential four-component reaction was tested in different solvents including water, ethanol, acetonitrile, tetrahydrofuran (THF), 1,4-dioxane and toluene. The results showed that ethanol was the best solvent. After determining the most optimal reaction conditions, the scope and efficiency of the reaction was discovered using a range of structurally diverse EDAMs and isatin derivatives to form the corresponding products **5a-v** (Table 1).

**Table 1:**
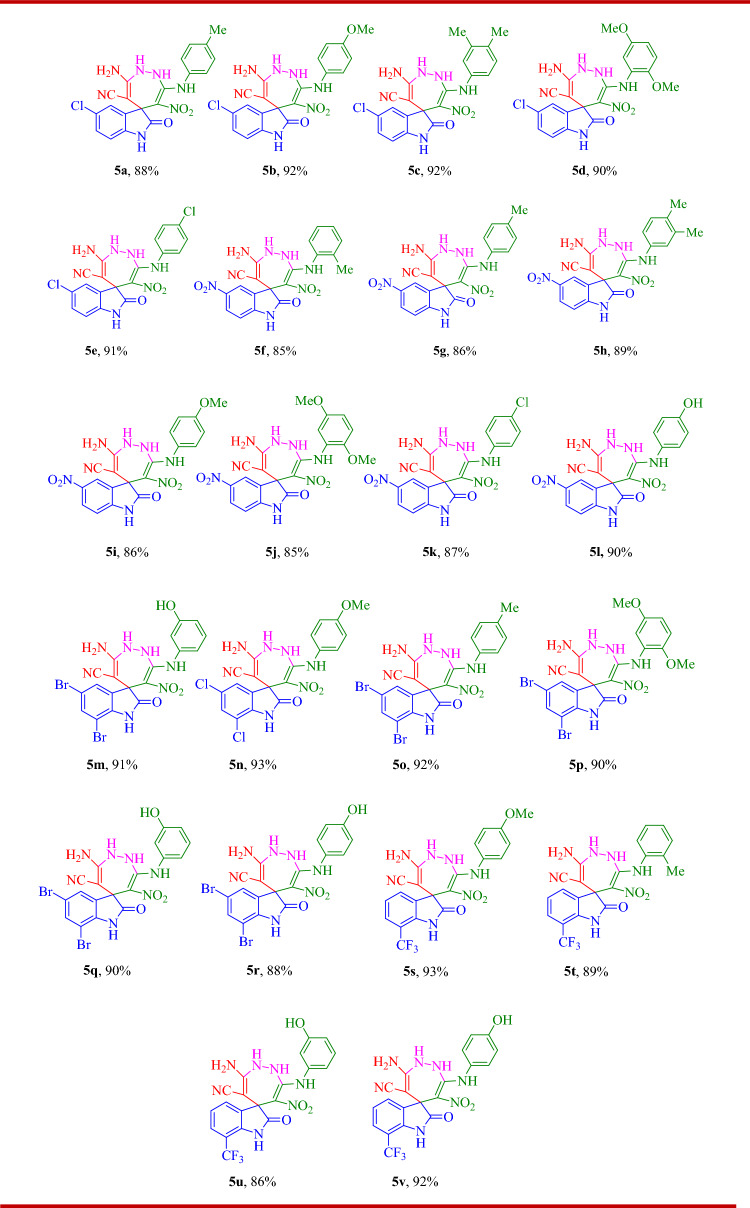
Synthesis of highly substituted spiro indolin-1,2-diazepine derivatives **5a-v**^a,b^

^a^Reaction conditions: N-aryl-1-(methylthio)-2-nitroethenamine (1 mmol), NH_2_NH_2_ (80% aq) (1.2 mmol) were added to ethanol (5 mL) at room temperature and after 3-4 h, isatin derivatives (1 mmol) and malononitrile (1 mmol) were added to obtain the desired product **5**.

^b^Isolated yield.

As shown in Table 1, the different structural groups of N-aryl-1-(methylthio)-2-nitroethenamine were successfully used to produce and their structures did not have a significant influence on the product yield. All N-aryl-1-(methylthio)-2-nitroethenamine derivatives used are good substrates for the cascade reaction for the synthesis of spiro-indolin-1,2 diazepine derivatives. In this study a range of different isatin derivatives was applied for the synthesis of spiro indolin-1,2-diazepine derivatives. As it has shown in Table 1 isatin derivatives having electron-withdrawing group NO_2_ (compounds **5f, 5g, 5h, 5i, 5j, 5k, 5l**) usually produced lower yields in comparison with other substituted isatins.

**Scheme 2.**
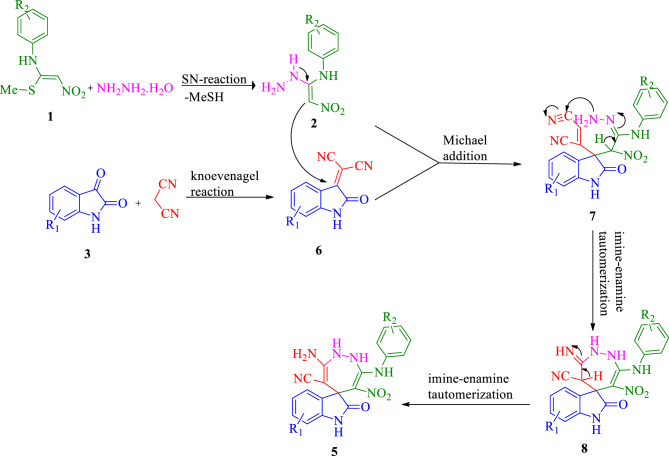
A plausible mechanism for the formation of indolin-1,2-diazepine derivatives.

A plausible mechanism is suggested in Scheme 2. The synthetic way to produce compound **5** is initiated with EDAM** 2** formation from the nucleophilic substitution of the NH_2_ group of hydrazine molecule with methylsulfanyl group of N-aryl-1-(methylthio)-2-nitroethenamine **1.** Then, the Michael addition between EDAM** 2** and intermediate **6**, which has formed from the condensation reaction of isatin with malononitrile, gives the intermediate **7**, which undergoes successive imine-enamine tautomerization, followed by nucleophilic addition of the amine to the cyano group, resulting in the formation of intermediate **8**. Finally, two imine-enamine tautomerizations of intermediates **7** and **8** produce the desired highly substituted spiro indolin-1,2-diazepine derivatives **5**.

### In vitro AChE and BChE inhibition

Seventeen spiro indolin-1,2-diazepine derivatives **5a–v** were synthesized, and all compounds were screened in vitro for inhibition of AChE and BChE (Table 2). The designed compounds exhibited varying degrees of ChEs inhibition compared with the standard inhibitor.

**Table 2:** The anti-AChE and anti-BChE activity of novel spiroindolin-1,2-diazepine derivitives, **5a-v**^**[a]**^**.**


			BChE		AChE	
Compound	R^1^	R^2^	% inhibition at 50 µM	IC_50_ (µM)	% inhibition at 50 µM	IC_50_ (µM)
**5a**	5-Cl	4- CH_3_	18.12 ± 2.88	–	5.37 ± 3.11	–
**5b**	5-Cl	4-OCH_3_	36.84 ± 2.10	–	42.84 ± 2.15	–
**5c**	5-Cl	3,4-diCH_3_	36.97 ± 3.78	–	Not active	–
**5d**	5- Cl	2,5-diOCH_3_	19.19 ± 4.07	–	27.82 ± 5.26	–
**5e**	5- Cl	4-Cl	Not active	–	13.40 ± 3.34	–
**5f**	5-NO_2_	2-CH_3_	23.30 ± 3.97	–	59.63 ± 6.75	22.38 ± 2.11
**5g**	5-NO_2_	4-CH_3_	24.41 ± 2.96	–	66.26 ± 6.57	5.88 ± 0.84
**5h**	5-NO_2_	3,4-diCH_3_	Not active	–	48.57 ± 2.70	–
**5i**	5-NO_2_	4-OCH_3_	31.20 ± 3.65	–	46.09 ± 3.38	–
**5j**	5-NO_2_	2,5-diOCH_3_	58.43 ± 3.55	17.37 ± 3.29	61.68 ± 3.77	20.89 ± 2.96
**5k**	5-NO_2_	4-Cl	17.89 ± 1.81	–	24.64 ± 1.70	-
**5l**	5-NO_2_	4-OH	5.71 ± 2.82	–	87.39 ± 5.93	3.98 ± 1.07
**5m**	5,7-diCl	2-CH_3_	41.94 ± 6.47	–	24.76 ± 5.48	–
**5n**	5,7-diCl	4-OCH_3_	46.50 ± 4.77	–	35.36 ± 7.54	–
**5o**	5,7-diBr	4-CH_3_	14.07 ± 3.70	–	37.57 ± 4.74	–
**5p**	5,7-diBr	2,5-diOCH_3_	59.26 ± 3.15	37.85 ± 4.85	73.36 ± 4.21	11.32 ± 1.65
**5q**	5,7-diBr	3-OH	37.15 ± 0.84	-	63.13 ± 4.43	12.03 ± 2.33
**5r**	5,7-diBr	4-OH	51.66 ± 1.29	45.70 ± 5.62	46.70 ± 1.92	-
**5s**	7-CF_3_	4-OCH_3_	13.38 ± 3.46	–	56.05 ± 4.39	17.78 ± 2.79
**5t**	7-CF_3_	2-CH_3_	7.31 ± 5.07	–	24.72 ± 1.94	-
**5u**	7-CF_3_	3-OH	10.45 ± 1.07	–	16.39 ± 0.53	-
**5v**	7-CF_3_	4-OH	13.03 ± 1.14	–	17.74 ± 1.82	-
**Donepezil**^[b]^				10.6 ± 2.1		0.079 ± 0.05

^**[a]**^Data presented here are the mean ± S.E of three to five independent experiments.

^[b]^Positive control.

To explain the structure–activity relationships (SARs), spiro indolin-1,2-diazepine hybrids were divided into five categories based on the type of substitutions at the R^1^ position, **5a-e**: R^1^ = 5-Cl, **5f–l**: R^1^ = 5-NO_2,_ **5m–n**: R^1^ = 5,7-diCl, **5o-r:** R^1^ = 5,7-diBr, **5s-v**: R^1^ = 7-CF_3_.

First, **5a-e** bearing 5-Cl moiety as a halogen-substituted group at R^1^ was designed and synthesized. As can be seen, **5a** bearing R^2^ = 4-CH_3_ demonstrated weak potency against both ChE enzymes. Next, compound **5b** which developed by the replacement of methyl on **5a** with methoxy was emerged as the most effective inhibitor against both targeted enzymes in this set. This activity might be due to the position and electron-donating effect of the methoxy group. It is worth mentioning that the selective BChE compound in this set was **5c** (R^1^ = 5-Cl) bearing R^2^ = 3,4-diCH_3_ as electron donating groups with 36.97% inhibition against BChE. Compound **5d** bearing 2,5-diOCH_3_ group was found to display reduced BChE inhibition in comparison with compound **5c** with a slight improvement in the anti-AChE activity. Importantly, the replacement of the electron-donating group with electron-withdrawing at the R^2^ position (**5e**) reduced the potency against BChE.

Next, 5-Cl substitution was replaced with 5-NO_2_ moiety at the R^1^ position as a strong electron-withdrawing group capable of forming hydrogen bond interaction (**5f-l)**. Noteworthy, the improvement in the AChE inhibition *vs* BChE was seen in all cases. The most potent derivative against AChE came back to **5l** (R^1^ = 5-NO_2_; R^2^ = 4-OH) with an IC_50_ value of 3.98 ± 1.07 µM. This improved potency may cause by the electron-donating and hydrogen bonding potencies of the OH group to participate in interaction with the enzyme. The other porent AChE inhibitors was **5g** with R^1^ = 5-NO_2_; R^2^ = 4-CH_3_ (IC_50_ = 5.88 ± 0.84 µM), **5j** with R^1^ = 5-NO_2_; R^2^ = 2,5-diOCH_3_ (IC_50_ = 20.89 ± 2.96 µM) and **5f** R^1^ = 5-NO_2_; R^2^ = 2-CH_3_ (IC_50_ = 22.38 µM) which all containing electron donating groups at R^2^. However, the presence of 4-Cl as an electron-withdrawing group at the R^2^ position reduced the potencies compared to the rest of the nitro-containing derivatives. It was proposed that the majority of the electronic density be imposed on the ring at the R^2^ position is in favor of AChE inhibition; in contrast, the reduction of electron density weakens the potency. Interestingly, a different trend was seen in BChE inhibition so that the presence of 5-NO_2_ moiety deteriorated the anti-BChE potencies. By illustration, **5l** and **5g** are the most active AChE inhibitors categorized as the least active agents against BChE. Among different moeities, 2,5-diOCH_3_ substitution at R^2^ (**5j**) was in favor of BChE inhibition with IC_50_ = 17.37 µM (58.43 ± 3.55% inhibition at 50 µM).

Evaluation of **5m** and **5n** containing di electron-withdrawing substitutions (R^1^ = 5,7-diCl) exhibited improved BChE inhibition *vs* AChE.

In the following di-chlorine moiety was replaced with bulk and more lipophilic bromine moiety (**5o-5r**). Overall 5,7-diBr recorded better potency against AChE in comparison with BChE. In assessments of **5o** and **5p**, we noticed a difference in the inhibitory potentials of these compounds concerning the position and the number of the R^2^ substituent. **5p** (R^2^: 2,5-diOCH_3_) having di-substitutions exhibited promising AChE (IC_50_ = 11.32 ± 1.65 µM) and BChE inhibition (IC_50_ = 37.85 ± 4.85 µM) compared with **5o**. It has been determined that compounds with 2,5-diOCH_3_ substitutions have a higher propensity to interact with the active site of targeted enzymes compared to those with 4-CH_3_ moiety.

Consequently, this increased interaction leads to an improvement in the inhibitory potential of these derivatives. The evaluations involving the substitution of the OH moiety have yielded significant findings. Specifically, the derivative **5r**, which contains a 4-OH substitution, exhibited greater potency against BChE with an IC_50_ value of 45.70 ± 5.62 µM, surpassing the activity of **5q** (R^2^ = 3-OH), which showed 37.15 ± 0.84% inhibition at a concentration of 50 µM. Conversely, contrasting results were observed in AChE inhibition. Compound **5q** (R^2^ = 3-OH), displayed better activity, revealing an IC_50_ value of 12.03 ± 2.33 µM, while derivative **5r**, featuring a 4-OH substitution, demonstrated lower activity.

Interesting results were recorded in the case of 7-CF_3_ derivatives so that **5q** (R^2^ = 4-OCH_3_) and **5m** (R^2^ = 2-CH_3_) exhibited improved AChE inhibitory potencies. Additionally, the substitution of the OH group at the *meta* (**5u**) and *para* (**5v**) positions did not result in a successful modification for significantly enhancing AChE and BChE inhibition.

**Figure 3:**
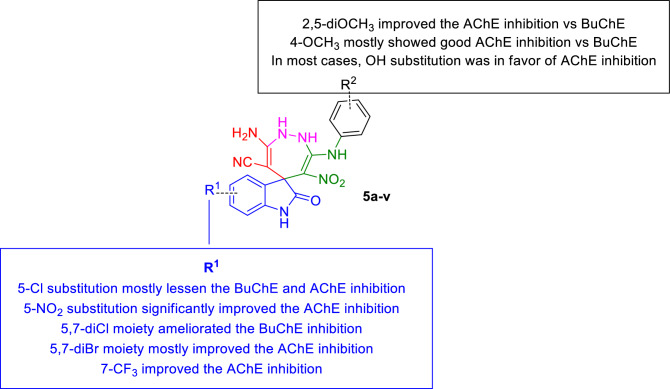
Summary of SAR of novel spiroindolin-1,2-diazepine derivitives as ChE inhibitor.

Overall, it was found that the nature, electron-donating or withdrawing effect, number, and position of the substituent at R^1^ and R^2^ may considerably affect the inhibitory potentials of the synthesized analogs. The summary of SAR is demonstrated in Fig. 3.

### Kinetic studies of AChE inhibition

**Figure 4:**
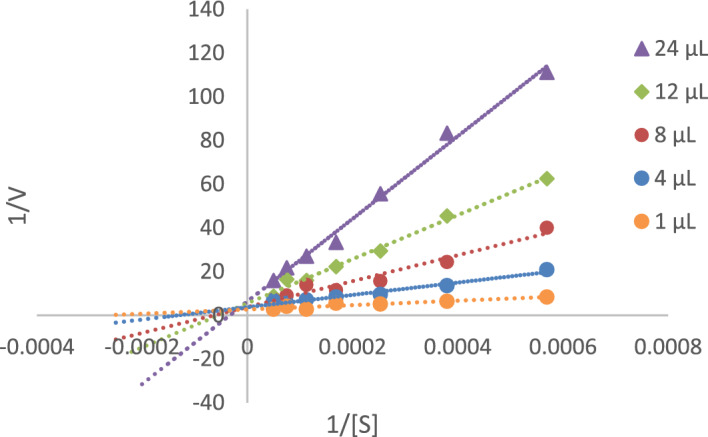
The Lineweaver–Burk plot of the most potent inhibitor **5l** at different concentrations (1, 4, 8, 12, 24 µM) against AChE. The x-axis is the reciprocal of the substrate concentrations (1 / [S]) and the y-axis is the reciprocal of the reaction velocity (1 / V).

To determine the mechanism of inhibition, a kinetic study of **5l** as the most potent AChE inhibitor was done against AChE. The reciprocal Lineweaver–Burk plot (Fig. 4) illustrate that K_m_ and V_max_ reduced with the increasing concentration of inhibitor, which indicates that **5l** is a mix type inhibitor.

**Figure 5:**
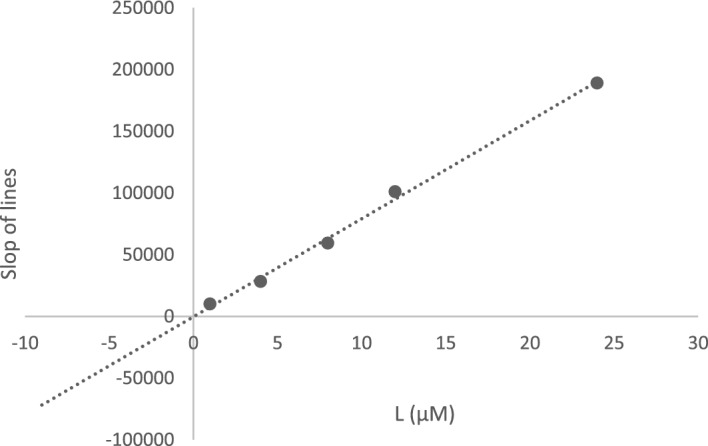
Double reciprocal Lineweaver–Burk plot of **5l** against AChE. The x-axis is the inhibitor concentrations (L) and the y-axis is the slope of the line of the Lineweaver–Burk plot (slop of lines).

Furthermore, the plot of the *K*_*m*_ versus different concentrations of **5l** gave an estimate of the inhibition constant, *K*_*i*_ of 0.044 µM, which is in accordance with the IC_50_ value of 3.98 ± 1.07 µM (Fig. 5).

### Docking study

Molecular docking was executed to understand the binding mechanism of **5l** as the most potent AChE inhibitor against both the targeted enzymes. The binding pocket of AChE, approximately 20 Å deep, comprises CAS pocket, includes Glu202, Ser203, and his447 of the main residues of the catalytic triad, while the anionic subsite consists of Trp86. PAS near the gorge's entrance comprises amino acids of Trp86, Tyr337, and Phe338.

Next, in silico studies of all analogs were executed. The molecular docking analysis of the designed derivatives revealed (Table 3) their docking scores against AChE in the range of -11.390 to -8.475 kcal/mol and against BChE in the range of -8.181 to -5.272 kcal/mol. These docking scores correlated with the observed biological activity, indicating that the derivatives exhibited greater activity in inhibiting AChE compared to BChE.

**Table 3:** Docking scores resulted of 5a–v against AChE and BChE.

Compounds	BChE (kcal/mol)	AChE (kcal/mol)
5a	− 6.107	− 8.955
5b	− 6.183	− 8.587
5c	− 5.272	− 8.171
5d	− 5.715	− 8.786
5e	− 6.180	− 9.171
5f	− 7.537	− 8.475
5g	− 6.906	− 11.390
5h	− 6.710	− 8.906
5i	− 8.181	− 8.985
5j	− 7.902	− 9.121
5k	− 6.982	− 8.242
5l	− 7.430	− 9.827
5m	− 5.788	− 9.510
5n	− 7.358	− 9.378
5o	− 6.183	− 9.116
5p	− 7.132	− 10.065
5q	− 6.589	− 9.537
5r	− 6.965	− 8.764
5s	− 7.960	− 9.193
5t	− 6.478	− 9.45
5u	− 6.643	− 8.630
5v	− 6.407	− 9.499

Specifically, in terms of AChE inhibition, compounds **5l** (IC_50_ = 3.98 ± 1.07 µM), **5g** (IC_50_ = 5.88 ± 0.84 µM), **5p** (IC_50_ = 11.32 ± 1.65 µM), and **5q** (IC_50_ = 12.03 ± 2.33 µM) were categorized as active compounds. These derivatives showed docking scores values of -9.827 kcal/mol, -11.390 kcal/mol, -10.065 kcal/mol, and -9.537 kcal/mol, respectively, with relatively lower docking scores against BChE. These results demonstrate that these derivatives exhibit higher selectivity towards inhibiting AChE than BChE, which is consistent with the observed biological activity. Among the derivatives, compound **5j** was identified as the most potent BChE inhibitor, exhibiting a good binding value of -7.902 kcal/mol compared to the other derivatives. However, an exception to this trend was observed with compound **5i**, which demonstrated good binding energy against BChE but displayed weak inhibitory activity in the biological results.

Furthermore, analysis of the binding interactions revealed that potent AChE inhibitors typically interacted with Asp74 (located in the PAS pocket) and His447 (part of the catalytic triad). In contrast, potent BChE inhibitors showed interaction with Trp82 of the PAS pocket. The type of observed interaction indicates that potent AChE inhibitors, by interacting with both critical pockets of AChE, exhibit better potency compared to BChE inhibitors, which only interact with the PAS pocket.

**Figure 6:**
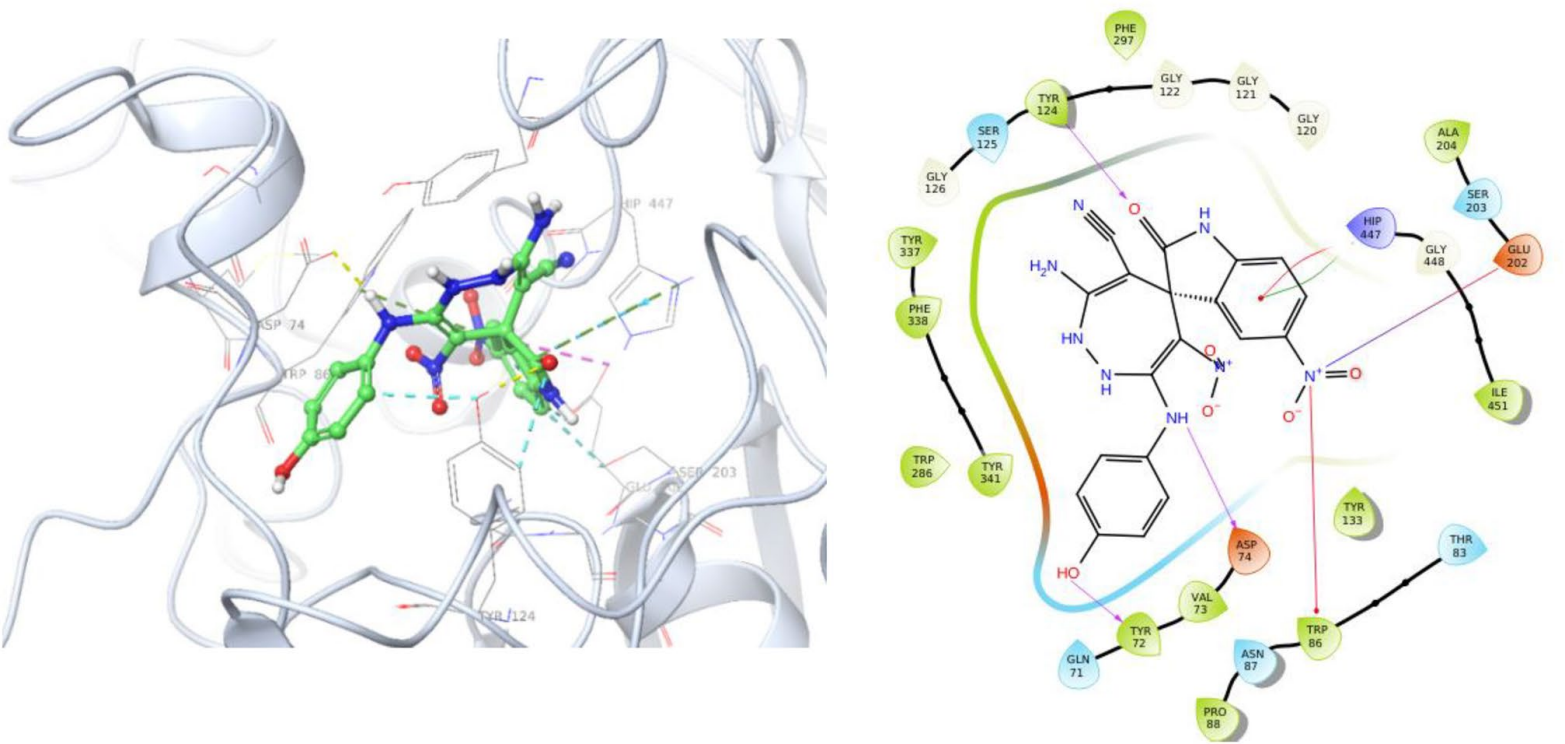
3D and 2D binding model of **5l** within active site of AChE.

The docking results of **5l** against AChE are exhibited in Fig. 6. The 5-nitroindolinone participated in interaction with Trp86 of anionic subsite consists and indolinone ring recorded H-bound interaction with Try124 plus two interactions with His447 of CAS pocket. On the other side of the molecule, hydroxyphenyl amines participated in two H-bound interactions with Tyr72 and Asp74 near the PAS pocket (Fig. 6).

**Figure 7:**
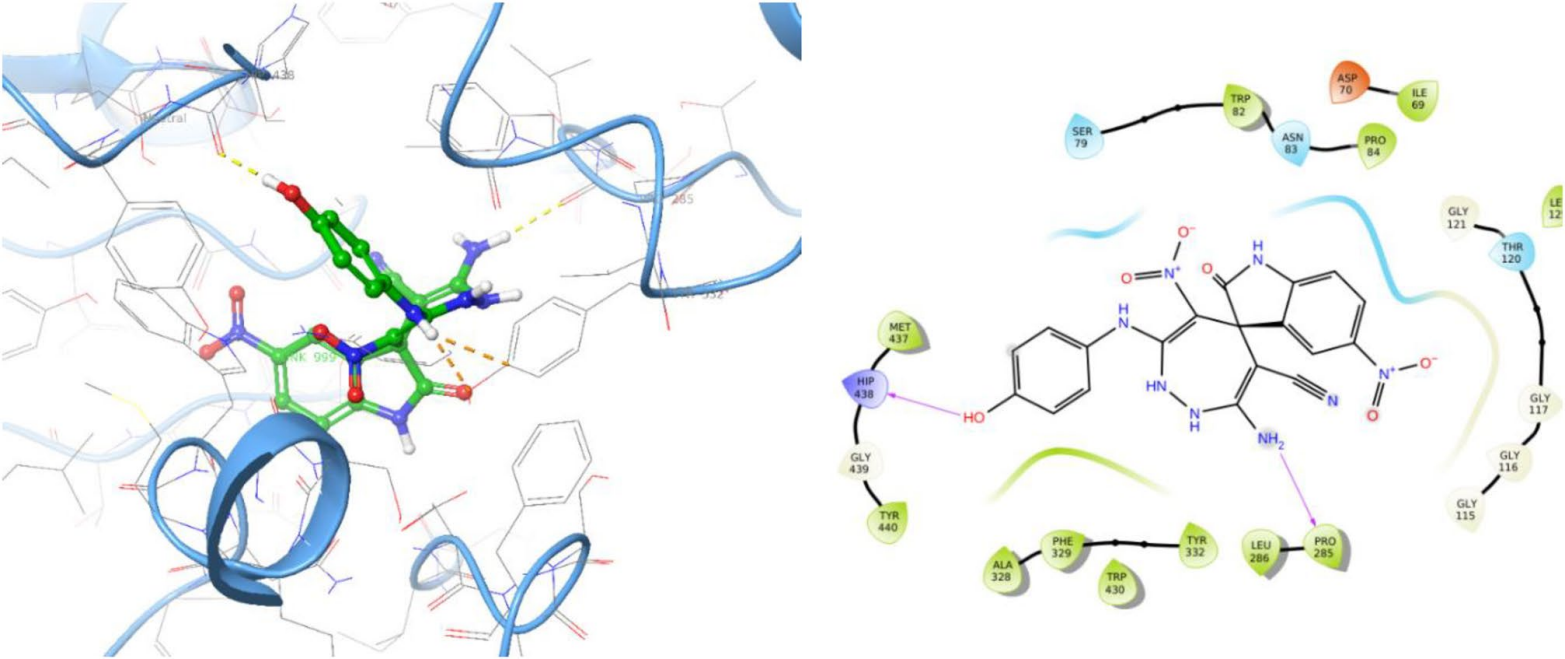
3D and 2D binding model of **5l** within active site of BChE.

Next, the molecular docking study of **5l** as the inactive BChE inhibitor was performed against BChE (Fig. 7). The binding mode showed hydrogen bond interactions with Pro285 and His438; however, this derivative demonstrated two unfavorable interactions with Tyr332 (exhibited in orange dash lines) which justify its low potency against BChE.

### Molecular dynamics simulations

**Figure 8:**
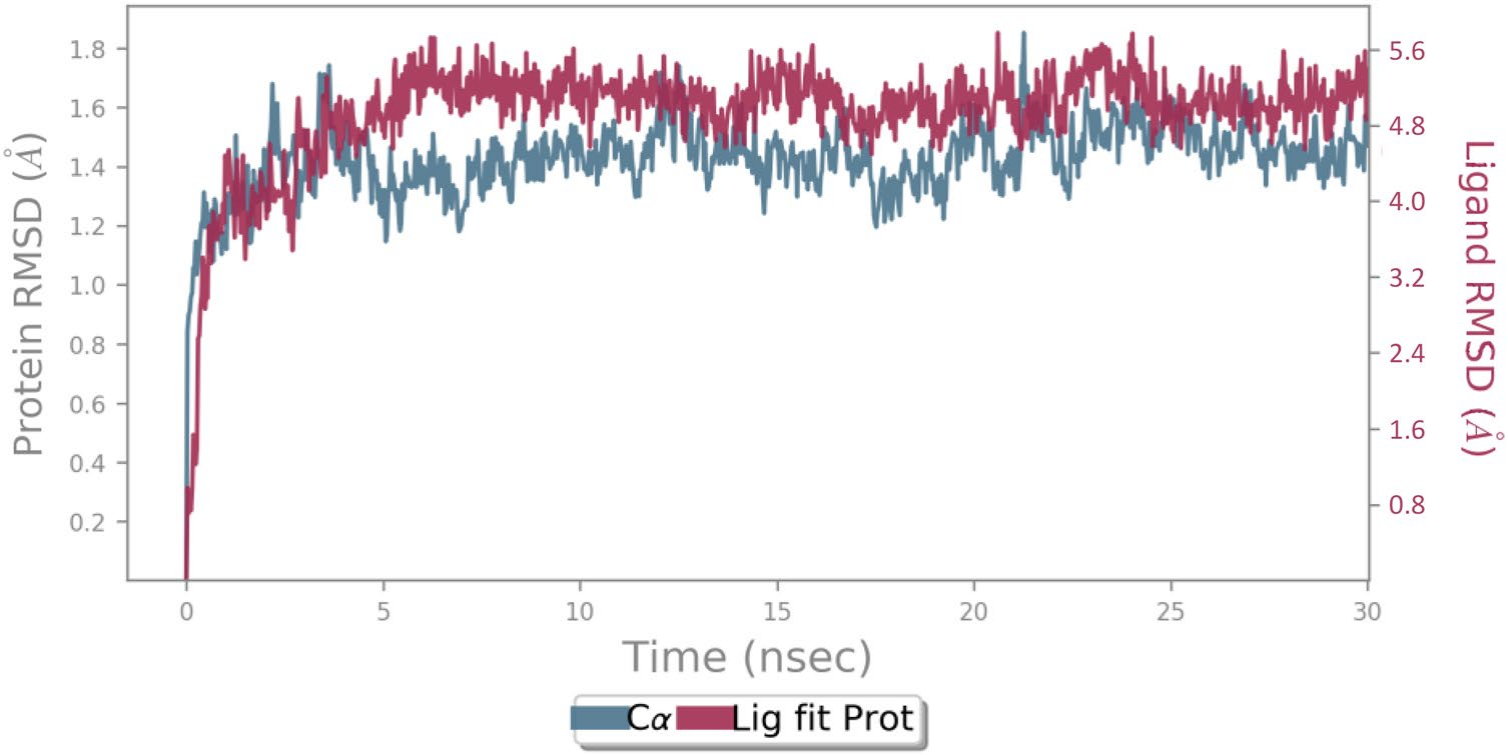
RMSD of the AChE backbone in complexed with compound **5l** (in blue), and the Ca atoms of the protein are depicted in blue.

The MD simulations was performed to understand the effect and structural perturbations of **5l** over the AChE enzyme active site. The root mean square deviation (RMSD) of the AChE was analyzed to evaluate the stability of theprotein–ligand complex. The RMSD value of the complex depicts approximately similar RMSD value compared with the enzyme backbone (Fig. 8). The RMSD value exhibited a sharp increase during the first 2.5 ns followed by a gradual rise up to 5 ns and steadily fluctuated till the end of the simulation time in a round 1.6 Å.

**Figure 9:**
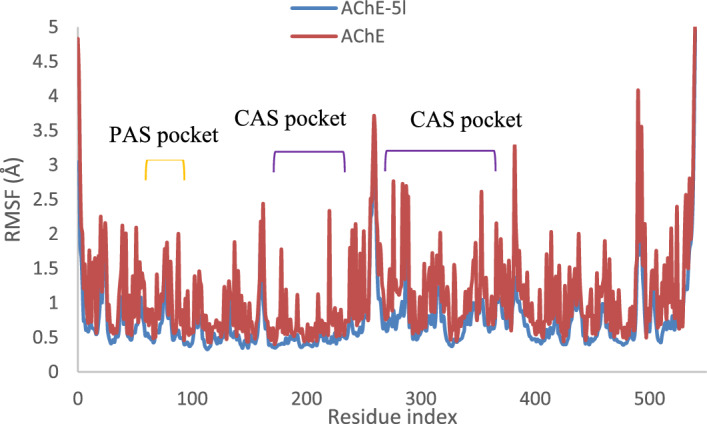
RMSF of the AChE backbone in complexed with compound **5l** (in blue color), and the Ca atoms of the protein (in red color). PAS and CAS pocket is presented in the orange and purple bracket.

The root mean square fluctuation (RMSF) is commonly used to analyze the flexibility of protein structures. In this study, the RMSF of complex AChE with compound **5l** in comparison with the apo form of the enzyme illustrated in Fig. 9. The RMSF analysis revealed that the overall RMSF values were lower in the presence of compound **5l**, indicating reduced flexibility compared to the apo enzyme. As exhibited, compound **5l** participated in favorable interactions with the binding site, resulting in reduced flexibility of both the PAS residues and the residues within the CAS pockets. The N-terminal tail, C-terminal tail, and residues between 255 and 266 showed the highest fluctuation, indicating significant movement.

**Figure 10:**
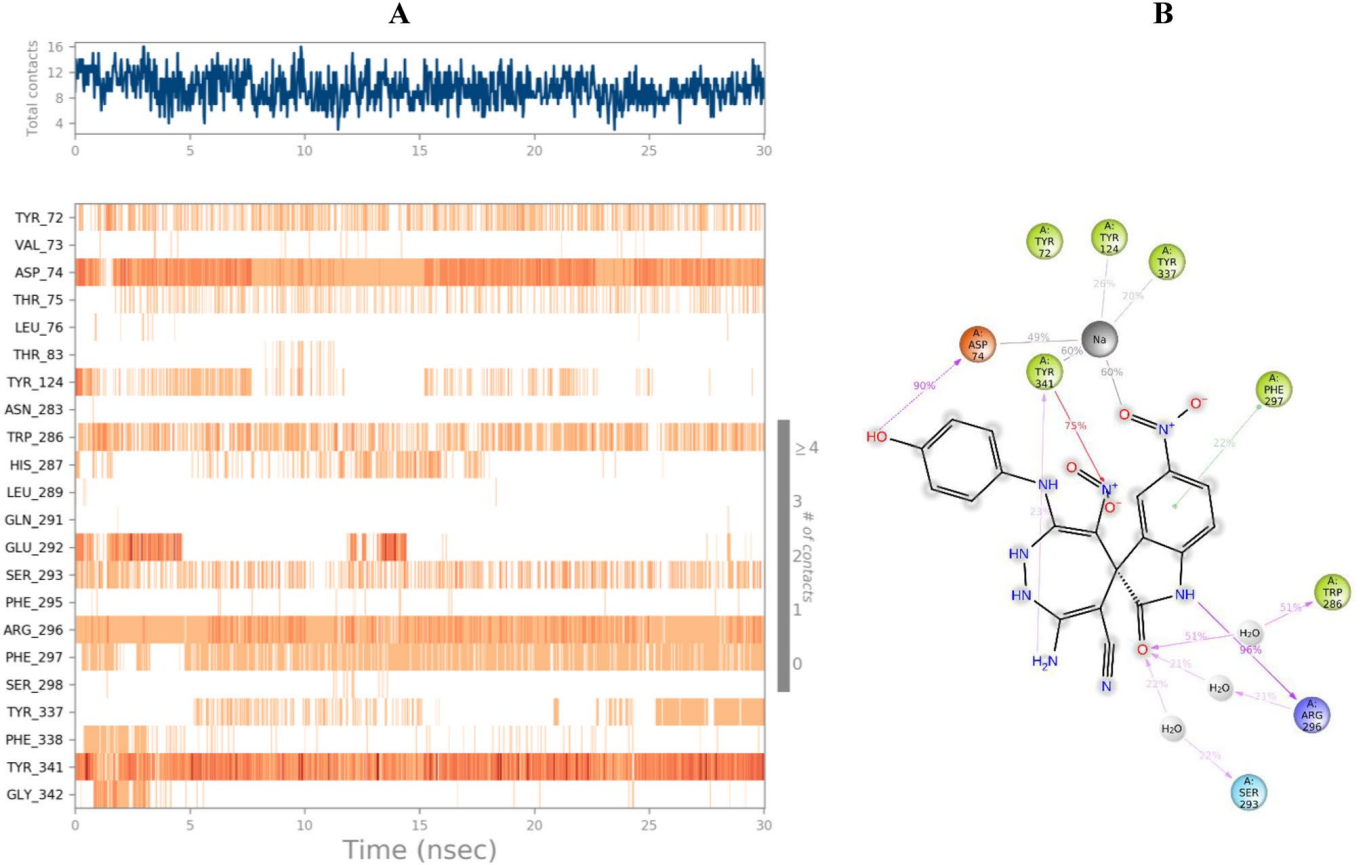
Protein–**5l** interaction during the whole simulation time in AChE. A) The timeline representation of the interactions shows the residues interacting with **5l** in each trajectory frame, B) 2D interaction diagram over simulation time. The residue involved in the interactions is presented. The purple arrow means H-bound, and the red line means pi-cation interaction.

In addition, different residues and types of interactions during the whole MD simulation time were exhibited in Fig. 10. Based on the timeline result, compound **5l** interacted with Asp74, Arg296, and Tyr341 more than 75% in MD run.

### Effect of 5l on SH-SY5Y cell viability

**Figure 11:**
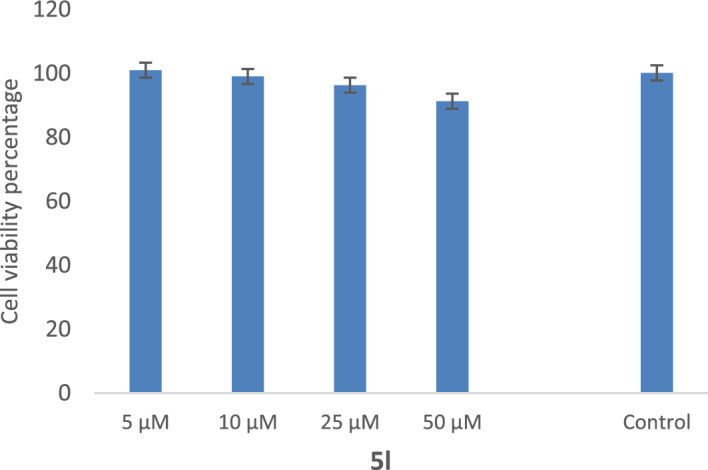
Cytotoxicity of **5l** after 72 h exposure determined by MTT assay. Data represent the mean ± SEM of three independent experiments.

Furthermore, the toxicity of **5l**, which is the most potent derivative against AChE with an IC_50_ value of 3.98 ± 1.07 µM, was evaluated against the SH-SY5Y neuroblastoma cell line. This cell line is commonly used as an in vitro neuronal model for studying neurodegeneration. The results, depicted in Fig. 11, demonstrated that the designed compounds showed no toxicity at the tested concentrations. Notably, despite its low IC_50_ value against AChE, this derivative exhibited no toxicity even at a concentration as high as 50 µM. Consequently, this derivative holds great potential for further investigation without concerns about inducing toxicity.

## Conclusion

In summary, novel series of spiro indolin-1,2-diazepine derivatives **5a–v** were designed as possible anti-AD agents. One-pot, novel, green, efficient, and simple sequential four-component synthesis approaches for the preparation of spiro indolin-1,2-diazepine derivatives using environment-friendly solvents and conditions were developed. The significant advantages of this protocol include readily available substrates, simple filtration, and washing of the crude product to obtain the pure product, minimizing solvent consumption by avoiding traditional purification techniques, such as column chromatography. In vitro inhibitory activities showed that compound **5l** was found to exhibit potential and selective inhibition against AChE (IC_50_ = 3.98 ± 1.07 µM), and **5j** was the potent inhibitor against both AChE (IC_50_ = 20.89 ± 2.96 µM) and BChE (IC_50_ = 17.37 ± 3.29 µM). The kinetic study of **5l** was also executed against AChE and indicated mix-type inhibition with promising *Ki* value of 0.044 µM. This compound did not show neurotoxicity in cell-based assays up to 50 µM against SH-SY5Y. Molecular docking studies of all derivatives against both enzymes indicated a higher affinity of these analogs towards occupying the active site of AChE compared to BChE. This preference could be attributed to the similar sizes of the derivatives, allowing them to better fit within the AChE pocket, which is relatively smaller than the larger BChE pocket. In silico studies also showed that the compound **5l** exhibited pronounced interaction with the essential AChE active site and MD simulation recorded stability of the **5l**-AChE complex. Regarding all aspects of the current study, including facile and appropriate synthetic methodology, as well as enzymatic, cell, and in silico assessments, **5l** can serve as a valuable lead compound and merit further investigations.

## Experimental

### Chemicals and apparatus

All chemicals were purchased from Merck or Fuluka chemical companies. ^1^H-NMR 300 MHz and ^13^C-NMR (75 MHz) spectra were run on a Bruker Avance 300 MHz instrument in DMSO-d_6_. Melting points were recorded as a Buchi B-545 apparatus in open capillary tubes. Mass spectra were recorded with an Agilent-5973 C insert XL MSD mass spectrometer (Ringoes, NJ) operating at an ionization potential of 70 eV. Reaction progress was screened by TLC using silica gel polygram SIL G/UV254 plates.

### General procedure for the synthesis of compounds 5a–v

Initially, to prepare EDAM **2** a mixture of N-aryl-1-(methylthio)-2-nitroethenamine **1** (1 mmol) and NH_2_NH_2_ (80% aq) (1.2 mmol) were stirred in ethanol (5 mL) at room temperature for 3-4 h. After that, isatin derivatives **3** (1 mmol) and malononitrile **4** (1 mmol) were added to this mixture and stirred for 12 h to complete the reaction confirmed by TLC. The crude solid formed, filtered, and washed with ethanol (5 ml) to give the pure product **5.**

#### 3-amino-5'-chloro-6-nitro-2'-oxo-7-(p-tolylamino)-1,2-dihydrospiro[[1,2]diazepine-5,3'-indoline]-4-carbonitrile (5a)

Pale yellow powder; Yield: 88%, m.p: 198–200 °C; (TLC; hexane–EtOAc, 2:5, R_f_ = 0.25); IR (KBr): 3326, 3262, 3162, 1722 (CO), 1644, 1573, 1475, 1353, 1299, 1255, 1203, 1153, 1106, 877, 815, 626. ^1^H NMR (300 MHz, DMSO*-d*_*6*_) δ: 11.08 (1H, s, NH), 10.59 (1H, s, NH), 7.41 (1H, d, *J* = 2.2 Hz, NH), 7.25 (1H, dd, *J* = 8.2, 2.3 Hz, Ar), 7.16 (2H, d, *J* = 8.1 Hz, Ar), 7.07–6.97 (2H, m, Ar), 6.83 (1H, d, *J* = 8.2 Hz, Ar), 6.77 (2H, s, NH_2_), 5.21 (2H, s, NH_2_), 2.29 (3H, s, Me) ; ^13^C NMR (75 MHz, DMSO*-d*_*6*_) δ: 177.5, 154.1, 151.5, 141.1, 137.1, 135.7, 134.5, 129.9, 128.5, 126.2, 123.6, 121.3, 118.6, 111.0, 109.9, 59.3, 51.7, 20.9; MS (m/z): 437 [M^+^], 390, 374, 279, 202, 186, 169, 152, 133, 106, 77.

#### 3-amino-5'-chloro-7-((4-methoxyphenyl)amino)-6-nitro-2'-oxo-1,2-dihydrospiro[[1,2]diazepine-5,3'-indoline]-4-carbonitrile (5b)

Yellow powder; Yield: 92%, m.p: 238–240 °C; (TLC; hexane–EtOAc, 2:5, R_f_ = 0.22); ^1^H NMR (300 MHz, DMSO*-d*_*6*_) δ: 11.22 (1H, s, NH), 10.57 (1H, s, NH), 7.41 (1H, d, *J* = 2.2 Hz, Ar), 7.24 (1H, dd, *J* = 8.2, 2.2 Hz, Ar), 7.15–7.04 (2H, m, Ar), 6.96–6.90 (2H, m, Ar), 6.83 (1H, d, *J* = 8.2 Hz, Ar), 6.75 (2H, s, NH_2_), 5.18 (2H, s, NH_2_), 3.76 (3H, s, OMe); ^13^C NMR (75 MHz, DMSO*-d*_*6*_) δ: 177.4, 157.0, 154.2, 151.8, 141.1, 135.7, 132.5, 128.4, 126.2, 123.6, 123.1, 118.6, 114.7, 111.0, 109.6, 59.3, 55.7, 51.7.

#### 3-amino-5'-chloro-7-((3,4-dimethylphenyl)amino)-6-nitro-2'-oxo-1,2-dihydrospiro[[1,2]diazepine-5,3'-indoline]-4-carbonitrile (5c)

Yellow powder; Yield: 92%, m.p: 238–240 °C; (TLC; hexane–EtOAc, 2:5, R_f_ = 0.21); IR (KBr): 3390, 3359, 3286, 3055, 2973, 2183 (CN), 1714 (CO), 1641, 1619, 1598, 1477, 1355, 1226, 11,991,108, 1070, 871, 630; ^1^H NMR (300 MHz, DMSO*-d*_*6*_) δ: 11.12 (1H, s, NH), 10.58 (1H, s, NH), 7.43 (1H, d, *J* = 2.2 Hz, NH), 7.25 (1H, dd, *J* = 8.2, 2.2 Hz, Ar), 7.11 (1H, d, *J* = 8.1 Hz, Ar), 6.95 (1H, d, *J* = 2.4 Hz, Ar), 6.88–6.82 (3H, m, Ar), 6.78 (2H, s, NH_2_) 5.19 (2H, s, NH_2_), 2.20 (6H, s, Me); ^13^C NMR (75 MHz, DMSO*-d*_*6*_) δ: 177.5, 154.1, 151.6, 141.1, 137.4, 137.2, 135.7, 133.4, 130.4, 128.5, 126.1, 123.7, 122.5, 118.8, 118.6, 111.0, 110.0, 59.3, 51.8, 19.9, 19.2; MS (m/z): 451 [M^+^], 408, 389, 362, 327, 309, 285, 258, 229, 187, 147, 120, 77.

#### 3-amino-5'-chloro-7-((2,5-dimethoxyphenyl)amino)-6-nitro-2'-oxo-1,2-dihydrospiro[[1,2]diazepine-5,3'-indoline]-4-carbonitrile (5d)

Orange powder; Yield: 90%, m.p: 214–216 °C; (TLC; hexane–EtOAc, 2:5, R_f_ = 0.385); IR (KBr): 3289, 3261, 3170, 3079, 2952, 2186 (CN), 1716 (CO), 1635, 1590, 1515, 1494, 1427, 1259, 1106, 1016, 941, 854, 800, 713; ^1^H NMR (300 MHz, DMSO*-d*_*6*_) δ: 11.20 (1H, s, NH), 10.61 (1H, s, NH), 7.43 (1H, d, *J* = 2.2 Hz, Ar), 7.25 (2H, dd, *J* = 8.2, 2.2 Hz, Ar), 7.04 (1H, d, *J* = 9.0 Hz, Ar), 6.89–6.80 (3H, m, Ar), 6.73 (1H, dd, *J* = 9.0, 2.9 Hz, Ar), 6.66 (1H, d, *J* = 3.0 Hz, Ar), 5.28 (2H, s, NH_2_), 3.82 (3H, s, OMe), 3.65 (3H, s, OMe); ^13^C NMR (75 MHz, DMSO*-d*_*6*_) δ: 177.4, 154.0, 153.5, 151.6, 145.4, 141.1, 135.6, 128.6, 128.5, 126.2, 123.6, 118.6, 113.1, 111.4, 111.0, 110.5, 107.2, 59.2, 56.8, 55.7, 51.8; MS (m/z): 483 [M^+^], 437, 390, 349, 285, 204, 180, 150, 108, 79.

#### 3-amino-5'-chloro-7-((4-chlorophenyl)amino)-6-nitro-2'-oxo-1,2-dihydrospiro[[1,2]diazepine-5,3'-indoline]-4-carbonitrile (5e)

Yellow powder; Yield: 91%, m.p: 230–232 °C; (TLC; hexane–EtOAc, 2:5, R_f_ = 0.228); ^1^H NMR (300 MHz, DMSO*-d*_*6*_) δ: 10.72 (1H, s, NH), 10.59 (1H, s, NH), 7.46–7.32 (3H, m, Ar), 7.25 (1H, dd, *J* = 8.3, 2.2 Hz, Ar), 7.16–7.05 (2H, m, Ar), 6.87–6.74 (3H, m, Ar), 5.29 (2H, s, NH_2_); ^13^C NMR (75 MHz, DMSO*-d*_*6*_) δ: ^13^C NMR (75 MHz, DMSO-*d*_*6*_) δ: 177.5, 153.9, 150.8, 141.0, 139.0, 135.9, 129.2, 128.7, 128.6, 126.3, 123.6, 122.6, 118.6, 111.1, 110.2, 59.4, 51.8.

#### 3-amino-5',6-dinitro-2'-oxo-7-(o-tolylamino)-1,2-dihydrospiro[[1,2]diazepine-5,3'-indoline]-4-carbonitrile (5f)

Light yellow powder; Yield: 85%, m.p: 260–262 °C; (TLC; hexane–EtOAc, 2:5, R_f_ = 0.29); IR (KBr): 3345, 3293, 3218, 3070, 2971, 2183 (CN), 1716 (CO), 1643, 1508, 1477, 1334, 1218, 1106, 1068, 900, 817, 732; ^1^H NMR (300 MHz, DMSO*-d*_*6*_) δ: 11.37 (1H, s, NH), 11.23 (1H, s, NH), 8.31 (1H, s, Ar), 8.26–8.16 (1H, m, Ar), 7.32 (1H, d, *J* = 6.9 Hz), 7.17 (3H, t, *J* = 8.1 Hz, Ar), 7.05 (1H, d, *J* = 8.6 Hz, Ar), 6.89 (2H, s, NH_2_), 5.17 (2H, s, NH_2_), 2.34 (3H, s, Me); ^13^C NMR (75 MHz, DMSO*-d*_*6*_) δ: 178.3, 154.3, 153.6, 148.8, 142.9, 138.5, 134.6, 132.0, 131.1, 126.9, 126.4, 126.2, 122.8, 119.3, 118.4, 109.8, 109.1, 58.1, 51.8, 18.5; MS (m/z): 448 [M^+^], 362, 359, 296, 240, 180, 131, 106, 65.

#### 3-amino-5',6-dinitro-2'-oxo-7-(p-tolylamino)-1,2-dihydrospiro[[1,2]diazepine-5,3'-indoline]-4-carbonitrile (5g)

Yellow powder; Yield: 86%, m.p: 212–214 °C; (TLC; hexane–EtOAc, 2:5, R_f_ = 0.235); IR (KBr): 3322, 3261, 3162, 3027, 2192 (CN), 1725 (CO), 1644, 1299, 1257, 1205, 1108, 813, 688, 628; ^1^H NMR (300 MHz, DMSO*-d*_*6*_) δ: 11.21 (1H, s, NH), 11.11 (1H, s, NH), 8.29 (1H, d, *J* = 2.4 H, Ar), 8.20 (1H, dd, *J* = 8.6, 2.4 Hz, Ar), 7.16 (2H, d, *J* = 8.2 Hz, Ar), 7.05 (3H, d, *J* = 8.5 Hz, Ar), 6.87 (2H, s, NH_2_), 5.23 (2H, s, NH_2_), 2.29 (3H, s, Me); ^13^C NMR (75 MHz, DMSO*-d*_*6*_) δ: 178.3, 154.3, 151.6, 148.7, 143.0, 137.0, 134.8, 134.7, 129.9, 126.2, 121.5, 119.3, 118.5, 109.8, 109.2, 58.2, 51.8, 20.9.

#### 3-amino-7-((3,4-dimethylphenyl)amino)-5',6-dinitro-2'-oxo-1,2-dihydrospiro[[1,2]diazepine-5,3'-indoline]-4-carbonitrile (5h)

Yellow powder; Yield: 89%, m.p: 248–250 °C; (TLC; hexane–EtOAc, 2:5, R_f_ = 0.235); IR (KBr): 3361, 3288, 3176, 3075, 2977, 2181 (CN), 1722 (CO), 1644, 1600, 1525, 1482, 1336, 1228, 1128, 1073, 916, 835, 634; ^1^H NMR (300 MHz, DMSO*-d*_*6*_) δ: 11.21 (1H, s, NH), 11.11 (1H, s, NH), 8.29 (1H, d, *J* = 2.4 Hz, Ar), 8.20 (1H, dd, *J* = 8.6, 2.4 Hz, Ar), 7.16 (2H, d, *J* = 8.2 Hz, Ar), 7.05 (3H, d, *J* = 8.5 Hz, Ar), 6.87 (2H, s, NH_2_), 5.23 (2H, s, NH_2_), 2.29 (3H, s, Me); ^13^C NMR (75 MHz, DMSO*-d*_*6*_) δ: 178.4, 154.3, 151.8, 148.7, 142.9, 137.4, 137.2, 134.8, 133.6, 130.4, 126.2, 122.6, 119.3, 119.0, 118.5, 109.8, 109.2, 58.2, 51.8, 19.9, 19.2.

#### 3-amino-7-((4-methoxyphenyl)amino)-5',6-dinitro-2'-oxo-1,2-dihydrospiro[[1,2]diazepine-5,3'-indoline]-4-carbonitrile (5i)

Yellow powder; Yield: 86%, m.p: 220–222 °C; (TLC; hexane–EtOAc, 2:5, R_f_ = 0.285); IR (KBr): 3392, 3307, 3261, 3124, 3062, 2971, 2173 (CN), 1735 (CO), 1646, 1604, 1506, 1477,1388,1245, 1214, 1184, 1066, 905, 842, 777, 628 ; ^1^H NMR (300 MHz, DMSO*-d*_*6*_) δ: 11.22 (2H, s, NH), 8.28 (1H, d, *J* = 2.4 Hz, NH), 8.20 (1H, dd, *J* = 8.6, 2.4 Hz, Ar), 7.14–7.08 (2H, m, Ar), 7.04 (1H, d, *J* = 8.6 Hz), 6.93 (2H, d, *J* = 9.0 Hz, Ar), 6.86 (2H, s, NH_2_), 5.21 (2H, s, NH_2_), 3.76 (3H, s, OMe); ^13^C NMR (75 MHz, DMSO*-d*_*6*_) δ: 178.3, 157.1, 154.4, 151.9, 148.7, 143.0, 134.9, 132.4, 126.2, 123.2, 119.3, 118.5, 114.7, 109.7, 108.8, 58.2, 55.7, 51.8.

#### 3-amino-7-((2,5-dimethoxyphenyl)amino)-5',6-dinitro-2'-oxo-1,2-dihydrospiro[[1,2]diazepine-5,3'-indoline]-4-carbonitrile (5j)

Light yellow powder; yield: 85%, m.p: 216–218 °C; (TLC; hexane–EtOAc, 2:5, R_f_ = 0.22); IR (KBr): 3372, 3313, 3253, 3083, 2994, 2190 (CN), 1722 (CO), 1633, 1513, 1428, 1344, 1261, 1072, 850, 808.; ^1^H NMR (300 MHz, DMSO*-d*_*6*_) δ: 11.39 (1H, s, NH), 11.24 (1H, s, NH), 8.29 (1H, d, *J* = 2.4 Hz, NH), 8.21 (1H, dd, *J* = 8.7, 2.4 Hz, Ar), 7.05 (2H, d, *J* = 8.8 Hz, Ar), 6.94 (2H, s, NH_2_), 6.85–6.68 (2H, m, Ar), 5.28 (2H, s, NH_2_), 3.83 (3H, s, OMe), 3.65 (3H, s, OMe); ^13^C NMR (75 MHz, DMSO*-d*_*6*_) δ: 178.2, 154.3, 153.5, 152.0, 148.8, 145.5, 142.9, 134.7, 128.5, 126.2, 119.2, 118.4, 113.1, 111.6, 109.8, 107.7, 58.1, 56.8, 55.7, 51.8.

#### 3-amino-7-((4-chlorophenyl)amino)-5',6-dinitro-2'-oxo-1,2-dihydrospiro[[1,2]diazepine-5,3'-indoline]-4-carbonitrile (5k)

Yellow powder, Yield: 87%, m.p: 215–217 °C; (TLC; hexane–EtOAc, 2:5, R_f_ = 0.275); IR (KBr): 3378, 3338, 3261, 2186 (CN), 1741, 1716 (CO), 1650, 1481, 1334, 1193, 1097, 1068, 838, 694; ^1^H NMR (300 MHz, DMSO*-d*_*6*_) δ: 11.21 (1H, s, NH), 10.79 (1H, s, NH), 8.30 (1H, d, *J* = 2.4 Hz, NH), 8.20 (1H, dd, *J* = 8.6, 2.4 Hz, Ar), 7.42–7.35 (2H, m, Ar), 7.18–7.08 (2H, m, Ar), 7.05 (1H, d, *J* = 8.6 Hz, Ar), 6.88 (2H, s, NH_2_), 5.31 (2H, s, NH_2_); ^13^C NMR (75 MHz, DMSO*-d*_*6*_) δ: 178.4, 154.1, 151.1, 148.5, 143.0, 138.9, 135.0, 129.2, 128.9, 126.2, 122.8, 119.3, 118.5, 109.8, 109.4, 58.3, 51.8; MS (m/z): 468 [M^+^], 421, 380, 359, 331, 296, 269, 222, 180, 153, 126, 99, 75.

#### 3-amino-7-((4-hydroxyphenyl)amino)-5',6-dinitro-2'-oxo-1,2-dihydrospiro[[1,2]diazepine-5,3'-indoline]-4-carbonitrile (5l)

Yellow powder, Yield: 90%, m.p: 220–222 °C; (TLC; hexane–EtOAc, 1:5, R_f_ = 0.270) ); IR (KBr): 3380, 3320, 3218, 3093, 2967, 2192 (CN), 1735, 1714 (CO), 1650, 1585, 1484, 1359, 1240, 1216, 1070, 933, 734, 588; ^1^H NMR (300 MHz, DMSO) δ: 13.45 (1H, s, OH), 11.71 (1H, s, NH), 11.36 (1H, s, NH), 8.55 (1H, d, *J* = 7.6 Hz, Ar), 7.70–7.66 (3H, m, Ar), 7.37 (2H, d, *J* = 8.4 Hz, Ar), 7.20 (1H, d, *J* = 8.6 Hz, Ar), 7.08 (2H, d, *J* = 8.4 Hz, Ar), 6.82 (2H, s, NH_2_), 6.24 (2H, s, NH_2_); ^13^C NMR (75 MHz, DMSO-*d*_*6*_) δ: 177.5, 154.0, 150.9, 141.0, 139.1, 135.9, 129.9, 128.8, 128.6, 126.3, 123.7, 122.6, 118.6, 111.0, 110.2, 59.7, 51.9.

#### 3-amino-5',7'-dichloro-6-nitro-2'-oxo-7-(o-tolylamino)-1,2-dihydrospiro[[1,2]diazepine-5,3'-indoline]-4-carbonitrile (5m)

Yellow powder; Yield: 91%, m.p: 198–200 °C; (TLC; hexane–EtOAc, 2:5, R_f_ = 0.28); IR (KBr): 3396, 3366, 3268, 2177 (CN), 1741, 1646, 1508, 1479, 1332, 1191, 1106, 1066, 898, 634; ^1^H NMR (300 MHz, DMSO*-d*_*6*_) δ : 11.40 (1H, s, NH), 10.60 (1H, s, NH), 7.44 (1H, d, *J* = 6.7 Hz, NH), 7.34–7.06 (4H, m, Ar), 6.96–6.63 (3H, m), 5.13 (2H, s, NH_2_), 2.33 (3H, s, Me); ^13^C NMR (75 MHz, DMSO*-d*_*6*_) δ: 177.4, 154.1, 153.5, 141.2, 138.5, 135.5, 131.9, 131.1, 128.5, 126.9, 126.2, 126.2, 123.7, 122.7, 118.5, 111.0, 109.9, 56.5, 51.7, 18.4.

#### 3-amino-5',7'-dichloro-7-((4-methoxyphenyl)amino)-6-nitro-2'-oxo-1,2-dihydrospiro[[1,2]diazepine-5,3'-indoline]-4-carbonitrile (5n)

Yellow powder; Yield: 93%, m.p: 2236–238 °C; (TLC; hexane–EtOAc, 2:5, R_f_ = 0.234); IR (KBr): 3359, 3315, 3289, 3162, 3052, 2969, 2177 (CN), 1712 (CO), 1641, 1552, 1348, 1297, 1249, 1114, 1031, 890, 817; ^1^H NMR (300 MHz, DMSO*-d*_*6*_) δ: 11.23 (1H, s, NH), 10.58 (1H, s, NH), 7.46–7.08 (4H, m, Ar), 6.95–6.76 (4H, m, Ar), 5.19 (2H, s, NH_2_), 3.76 (3H, s, OMe); ^13^C NMR (75 MHz, DMSO*-d*_*6*_) δ: 177.4, 157.1, 154.2, 151.8, 141.1, 135.8, 132.4, 128.5, 126.2, 123.6, 123.1, 118.6, 114.7, 111.0, 109.6, 59.3, 55.7, 51.7; MS (m/z): 487 [M^+^], 468, 421, 380, 359, 331, 315, 296, 269, 240, 222, 180, 153, 126, 99, 75.

#### 3-amino-5',7'-dibromo-6-nitro-2'-oxo-7-(p-tolylamino)-1,2-dihydrospiro[[1,2]diazepine-5,3'-indoline]-4-carbonitrile (5o)

Light brown; Yield: 92%, m.p: 226–228 °C; (TLC; hexane–EtOAc, 2:5, R_f_ = 0.335); IR (KBr):3343, 3282, 3143, 2923, 2196 (CN), 1716 (CO)1644, 1504, 1349, 1191, 1016, 887, 757; ^1^H NMR (300 MHz, DMSO*-d*_*6*_) δ: 11.09 (1H, s, NH), 10.95 (1H, s, NH), 7.65 (1H, d, *J* = 1.9 Hz, Ar), 7.59 (1H, d, *J* = 1.9 Hz, Ar), 7.16 (2H, d, *J* = 8.1 Hz, Ar), 7.03 (2H, d, *J* = 8.4 Hz, Ar), 6.86 (2H, s, NH_2_), 5.20 (2H, s, NH_2_), 2.29 (3H, s, Me); ^13^C NMR (75 MHz, DMSO*-d*_*6*_) δ: 177.3, 154.2, 151.5, 141.2, 137.3, 137.0, 134.6, 133.3, 129.9, 125.6, 121.4, 118.5, 114.4, 109.6, 103.0, 58.8, 52.9, 20.9.

#### 3-amino-5',7'-dibromo-7-((2,5-dimethoxyphenyl)amino)-6-nitro-2'-oxo-1,2-dihydrospiro[[1,2]diazepine-5,3'-indoline]-4-carbonitrile (5p)

Yellow pow﻿der; Yield: 90%, m.p: 223–225 °C; (TLC; hexane–EtOAc, 2:5, R_f_ = 0.315); ^1^H NMR (300 MHz, DMSO*-d*_*6*_): 11.21 (1H, s, NH), 10.97 (1H, s, NH), 7.65 (1H, d, *J* = 1.9 Hz, Ar), 7.60 (1H, d, *J* = 1.9 Hz, Ar), 7.04 (1H, d, *J* = 9.0 Hz, Ar), 6.92 (2H, s, NH_2_), 6.74 (1H, dd, *J* = 9.0, 3.0 Hz, Ar), 6.66 (1H, d, *J* = 3.0 Hz, Ar), 5.26 (2H, s, NH_2_), 3.82 (3H, s, OMe), 3.66 (3H, s, OMe); ^13^C NMR (75 MHz, DMSO*-d*_*6*_) δ: 177.2, 154.2, 153.5, 151.6, 145.4, 141.2, 137.2, 133.3, 128.5, 125.6, 118.5, 114.3, 113.1, 111.6, 110.1, 107.3, 103.0, 58.6, 56.8, 55.8, 52.9.

#### 3-amino-5',7'-dibromo-7-((3-hydroxyphenyl)amino)-6-nitro-2'-oxo-1,2-dihydrospiro[[1,2]diazepine-5,3'-indoline]-4-carbonitrile (5q)

Yellow powder; Yield: 90%, m.p: 230–232 ºC; (TLC; hexane-EtOAc, 2:5, R_f_ = 0.305); ^1^H NMR (300 MHz, DMSO-*d*_*6*_) δ: 11.75 (1H, s, OH), 10.94 (1H, s, NH), 9.61 (1H, s, NH), 7.60 (2H, dd, *J *= 15.1, 1.8 Hz, Ar), 7.09 (1H, t, *J* = 8.1 Hz, Ar), 6.87 (2H, s, Ar), 6.60 – 6.46 (3H, m, Ar), 5.19 (2H, s, NH); ^13^C NMR (75 MHz, DMSO-*d*_*6*_) δ: 177.3, 158.4, 154.1, 151.2, 141.1, 140.6, 137.3, 133.3, 130.1, 125.7, 118.5, 114.4, 112.5, 111.8, 110.0, 108.6, 103.0, 58.6, 56.5.       

#### 3-amino-5',7'-dibromo-7-((4-hydroxyphenyl)amino)-6-nitro-2'-oxo-1,2-dihydrospiro[[1,2]diazepine-5,3'-indoline]-4-carbonitrile (5r)

Yellow powder; Yield: 88%, m.p: 236–238 ºC; (TLC; hexane-EtOAc, 2:5, R_f_ = 0.315); ^1^H NMR (300 MHz, DMSO-*d*_*6*_) δ: 11.43 (1H, s, OH), 10.93 (1H, s, NH), 9.56 (1H, s, NH), 7.59 (2H, dd, J = 24.9, 1.9 Hz, Ar), 6.98 (2H, d, *J* = 8.4 Hz, Ar), 6.84 (2H, s, Ar), 6.73 (2H, d, *J* = 8.4 Hz, Ar), 5.09 (2H, s, NH); ^13^C NMR (75 MHz, DMSO-*d*_*6*_) δ: 177.3, 155.8, 154.4, 152.1, 141.29, 137, 133.2, 130.6, 125.6, 123.5, 118.5, 116.0, 114.3, 109.3, 103.0, 58.3, 52.8.

#### 3-amino-7-((4-methoxyphenyl)amino)-6-nitro-2'-oxo-7'-(trifluoromethyl)-1,2-dihydrospiro[[1,2]diazepine-5,3'-indoline]-4-carbonitrile (5s)

Yellow powder; Yield: 93%, m.p: 227–229 °C; (TLC; hexane–EtOAc, 2:5, R_f_ = 0.33); IR (KBr): 3359, 3315, 3289, 3052, 2969, 2177 (CN), 1712 (CO), 1641, 1508, 1477, 1348, 1297, 1249, 1197, 1114, 1031, 890, 763; ^1^H NMR (300 MHz, DMSO*-d*_*6*_): 11.04 (1H, s, NH), 10.91 (1H, s, NH), 7.59 (1H, d, *J* = 7.4 Hz, Ar), 7.50 (1H, d, *J* = 8.0 Hz, Ar), 7.18 (1H, t, *J* = 7.7 Hz, Ar), 7.08 (2H, d, *J* = 8.8 Hz, Ar), 6.97–6.87 (2H, m, Ar), 6.80 (2H, s, NH_2_), 5.25 (2H, s, NH_2_), 3.75 (3H, s, OMe); ^13^C NMR (75 MHz, DMSO*-d*_*6*_) δ: 178.2, 157.0, 154.1, 151.6, 139.6, 135.8, 132.4, 127.3, 126.0, 125.2, 122.9, 122.3, 118.5, 114.6, 110.8, 109.2, 59.0, 55.7, 50.7; MS (m/z): 487 [M^+^], 425, 390, 345, 320, 263, 228, 202, 149, 108, 81.

#### 3-amino-6-nitro-2'-oxo-7-(o-tolylamino)-7'-(trifluoromethyl)-1,2-dihydrospiro[[1,2]diazepine-5,3'-indoline]-4-carbonitrile (5t)

Yellow powder; Yield: 89%, m.p: 138–140 °C; (TLC; hexane–EtOAc, 2:5, R_f_ = 0.255); IR (KBr): 3338, 3288, 3166, 3093, 2996, 2200 (CN), 1722 (CO), 1646, 1608, 1504, 1357, 1292, 1236, 1201, 1139, 1020, 730, 568; ^1^H NMR (300 MHz, DMSO*-d*_*6*_) δ: 11.20 (1H, s, NH), 10.92 (1H, s, NH), 7.61 (1H, d, *J* = 7.4 Hz, Ar), 7.50 (1H, d, *J* = 8.0 Hz, Ar), 7.31 (1H, d, *J* = 6.9 Hz, Ar), 7.23–7.04 (4H, m), 6.82 (2H, s, NH_2_), 5.20 (2H, s, NH_2_), 2.33 (3H, s, Me).

#### 3-amino-7-((3-hydroxyphenyl)amino)-6-nitro-2'-oxo-7'-(trifluoromethyl)-1,2-dihydrospiro[[1,2]diazepine-5,3'-indoline]-4-carbonitrile (5u)

Yellow powder; Yield: 86%, m.p: 214–216 ºC; (TLC; hexane-EtOAc, 2:5, R_f_ = 0.310); ^1^H NMR (300 MHz, DMSO-*d*_*6*_): 10.90 (1H, s, OH), 10.64 (1H, s, NH), 9.59 (1H, s, NH), 7.57 (1H, d, *J *= 7.3 Hz, Ar), 7.48 (1H, d,* J* = 8.0 Hz, Ar), 7.16 (1H, t, *J* = 7.7 Hz, Ar), 7.07 (1H, t,* J* = 7.9 Hz, Ar), 6.82 (1H, s, Ar), 6.51 (3H, td, *J* = 8.6, 8.0, 4.2 Hz, Ar), 5.26 (2H, s, NH); ^13^C NMR (75 MHz, DMSO-*d*_*6*_) δ: 178.2, 158.4, 154.0, 150.9, 140.7, 135.8, 130.1, 127.4, 126.0, 122.3, 118.5, 112.3, 111.6, 109.9, 108.3, 58.9, 53.2.

#### 3-amino-7-((4-hydroxyphenyl)amino)-6-nitro-2'-oxo-7'-(trifluoromethyl)-1,2-dihydrospiro[[1,2]diazepine-5,3'-indoline]-4-carbonitrile (5v)

Yellow powder; Yield: 92%, m.p: 218–220 ºC; (TLC; hexane-EtOAc, 2:5, R_f_ = 0.320); ^1^H NMR (300 MHz, DMSO-*d*_*6*_) δ: 11.22 (1H, s, OH), 10.88 (1H, s, NH), 9.52 (1H, s, NH), 7.55 (1H, d, *J* = 7.4 Hz, Ar), 7.47 (1H, d,* J* = 8.1 Hz, Ar), 7.15 (1H, t, *J *= 7.8 Hz, Ar), 6.95 (2H, d, *J* = 8.4 Hz, Ar), 6.82 – 6.66 (4H, m, Ar), 5.18 (2H, s, NH); ^13^C NMR (75 MHz, DMSO-*d*_*6*_) δ: 165.6, 158.3, 157.3, 152.5, 146.0, 144.0, 139.9, 132.5, 130.1, 126.9, 122.2, 117.3, 115.1, 112.3, 110.7, 108.6, 72.8, 53.3.

### AChE and BChE inhibition

Cholinesterase inhibitory activities of all analogs were evaluated spectrometrically using the modified Ellman method as previously reported^40,41^. 20 µL AChE 0.18 units/mL, or 20 µL BChE iodide 0.162 units/mL and 20 µL DTNB (301 μM) were added to 200 μl sodium phosphate buffer (0.1 mol/L, pH 7.4) in separate wells of a 96-well microplate and gently mixed. Then, 10 μl of different concentrations of test compounds were added to each well and incubated for 15 min at 37 °C followed by the addition of acetylthiocholine (ATCh) or butyrylthiocholine (BTCh) (20 μl, final concentration of 452 μM) to produce the yellow anion of 5-thio-2-nitrobenzoic acid. The absorbance of each well was measured at 415 nm using a microplate reader. IC_50_ values and inhibition values were calculated with the software GraphPad Prism as the mean of three independent experiments and are expressed as mean ± SEM.

### Enzyme kinetic studies

As previously reported, the kinetic study of AChE was carried out at five different concentrations of compound **5l** and acetylthiocholine substrate (0.1–1 mM) by Ellman's method^42^.

### Molecular docking

The molecular docking approach was performed using induced-fit molecular docking (IFD) of the Schrodinger package. The SMILE format of **5l** was converted to a three-dimensional structure within the Maestro software package. The X-ray structures of AChE (PDB code: 4EY7) and BChE (PDB code: 4BDS) were prepared with the Protein Preparation Wizard interface of Maestro via removing the ligand and water molecules, adding hydrogen atoms, optimizing their position, and assigning the ionization states of acid and basic residues according to PROPKA prediction at pH 7.0. The molecular docking was performed using IFD mode with the ligands as flexible, the force field was set as OPLS-2005, and all other parameters were set to default. The binding site was used to generate the grid for IFD calculation. The maximum 20 poses with receptor and ligand van der Waals radii of 0.7 and 0.5, respectively considered. Residues within 8 Å of the crystallographic ligands at the active site were refined, followed by side-chain optimization. Structures in which prime energy is more than 30 kcal/mol are eliminated. The re-docking experiment for validation of the used docking protocol was done and recorded the RMSD value of 0.79, indicating the docking experiment is reliable^43,44^.

### Molecular dynamic simulations

Molecular simulations of this study were performed using the Desmond v5.3 using the Maestro interface (from Schrödinger 2018‐4 suite). The appropriate pose for the MD simulation procedure of the compound was achieved by the IFD method. To build the system for MD simulation, the protein–ligand complex was solvated with SPC explicit water molecules and placed in the center of an orthorhombic box of appropriate size in the periodic boundary condition. Sufficient counter‐ions and a 0.15 M solution of NaCl were also utilized to neutralize the system and to simulate the real cellular ionic concentrations, respectively. The MD protocol involved minimization, pre-production, and finally, production MD simulation steps. In the minimization procedure, the entire system was allowed to relax for 2500 steps by the steepest descent approach. Then the temperature of the system was raised from 0 to 300 K with a small force constant on the enzyme to restrict any drastic changes. MD simulations were performed via NPT (constant number of atoms, constant pressure i.e. 1.01325 bar, and constant temperature i.e. 300 K) ensemble. The optimum system was finally subjected to produce MD simulations for 30 ns for the protein–ligand complex. During the simulation, every 1000 ps of the actual frame was stored. The dynamic behavior and structural changes of the systems were analyzed by the calculation of the RMSD and RMSF. Subsequently, the representative structures of the simulation were extracted based on the clustering method from the equilibrated trajectory system for investigating of ligand–protein complex interaction.

### Toxicity assay on SH-SY5Y

SH-SY5Y cells were maintained in Dulbecco's modified Eagle medium with Ham's F12 medium (DMEM/F12) containing 15% fetal bovine serum100 units/ml penicillin and 100 µg/ml streptomycin. Cells were seeded into flasks containing supplemented medium and maintained at 37 ˚C in a humidified atmosphere of 5% CO_2_ and 95% air. Cell viability, virtually the mitochondrial activity of living cells, was measured by quantitative colorimetric assay with MTT, as described previously. MTT reagent, at a final concentration of 0.5 mg/ml, was added to each well at the end of the incubation period, and the plate was placed in a humidified incubator for an additional two h periods. Metabolically active cells convert the yellow MTT tetrazolium compound to a purple formazan product. Then, the insoluble formazan was dissolved with dimethylsulfoxide; colorimetric determination of MTT reduction was measured at 540 nm. Control cells treated media were taken as 100% viability.

## Supplementary Information

Supplementary Information.

## Data Availability

The datasets generated and/or analyzed during the current study are available in the Worldwide Protein Data Bank with PDB ID of 4EY7 and 4BDS repository.
