# Supplementary material for: The anti-Alzheimer potential of novel spiroindolin-1,2-diazepine derivatives as targeted cholinesterase inhibitors with modified substituents
Source: Sci Rep. 2023 Jul 24;13:11952. doi: 10.1038/s41598-023-38236-0 (PMC10366214; doi:10.1038/s41598-023-38236-0)
Supplement: Supplementary file 1 — Supplementary Information. [file 41598_2023_38236_MOESM1_ESM.pdf]

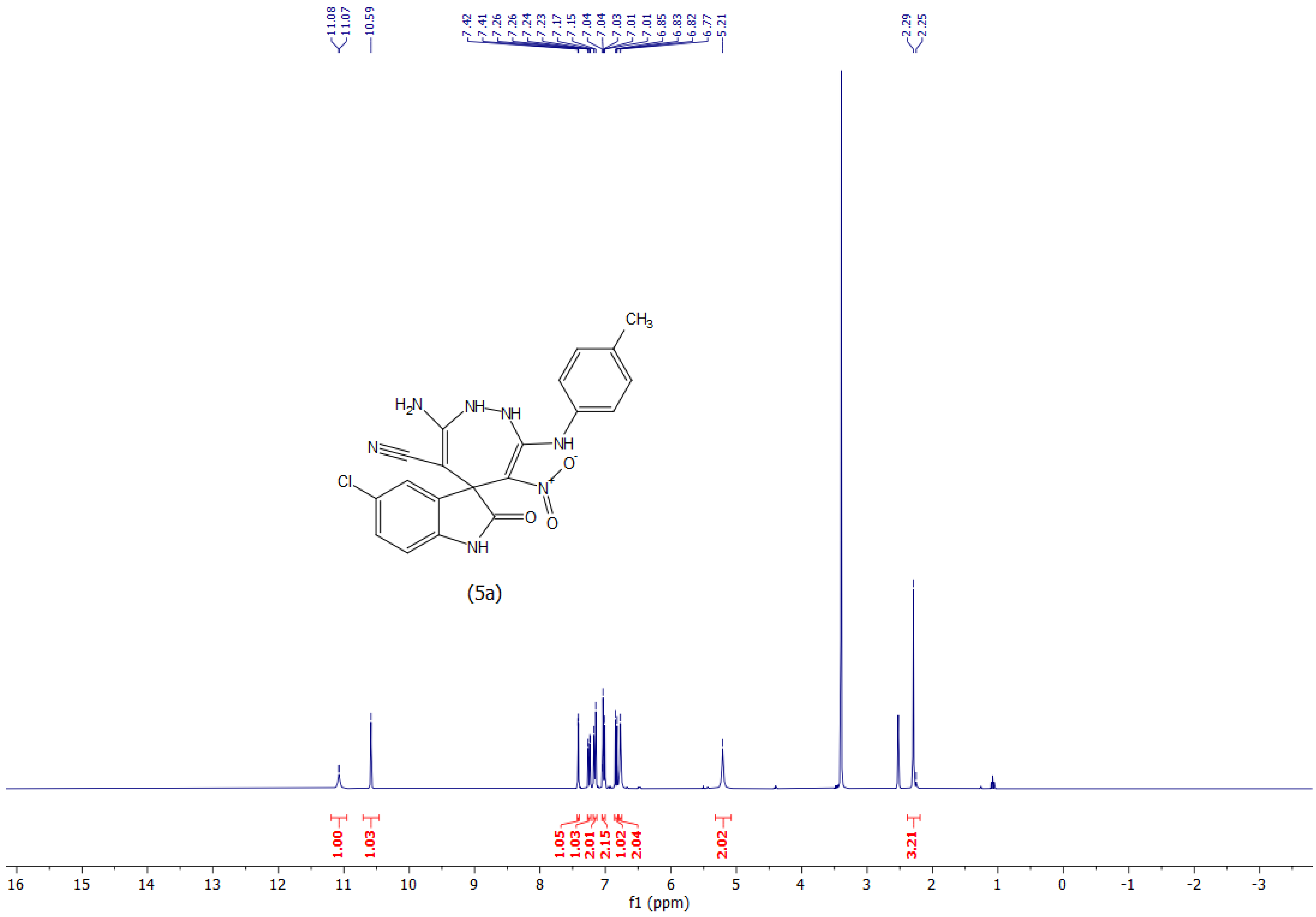

11.08
11.07
10.59
7.42
7.41
7.26
7.26
7.24
7.23
7.17
7.15
7.04
7.04
7.03
7.01
7.01
6.85
6.83
6.82
6.77
5.21
2.29
2.25
H_2N
NH
NH
NH
N
Cl
CH_3
O^-
N^+
O
O
NH
1.00
1.03
1.05
1.03
2.01
2.15
1.02
2.04
2.02
3.21
16
15
14
13
12
11
10
9
8
7
6
5
4
3
2
1
0
-1
-2
-3
f1 (ppm)

(5a)

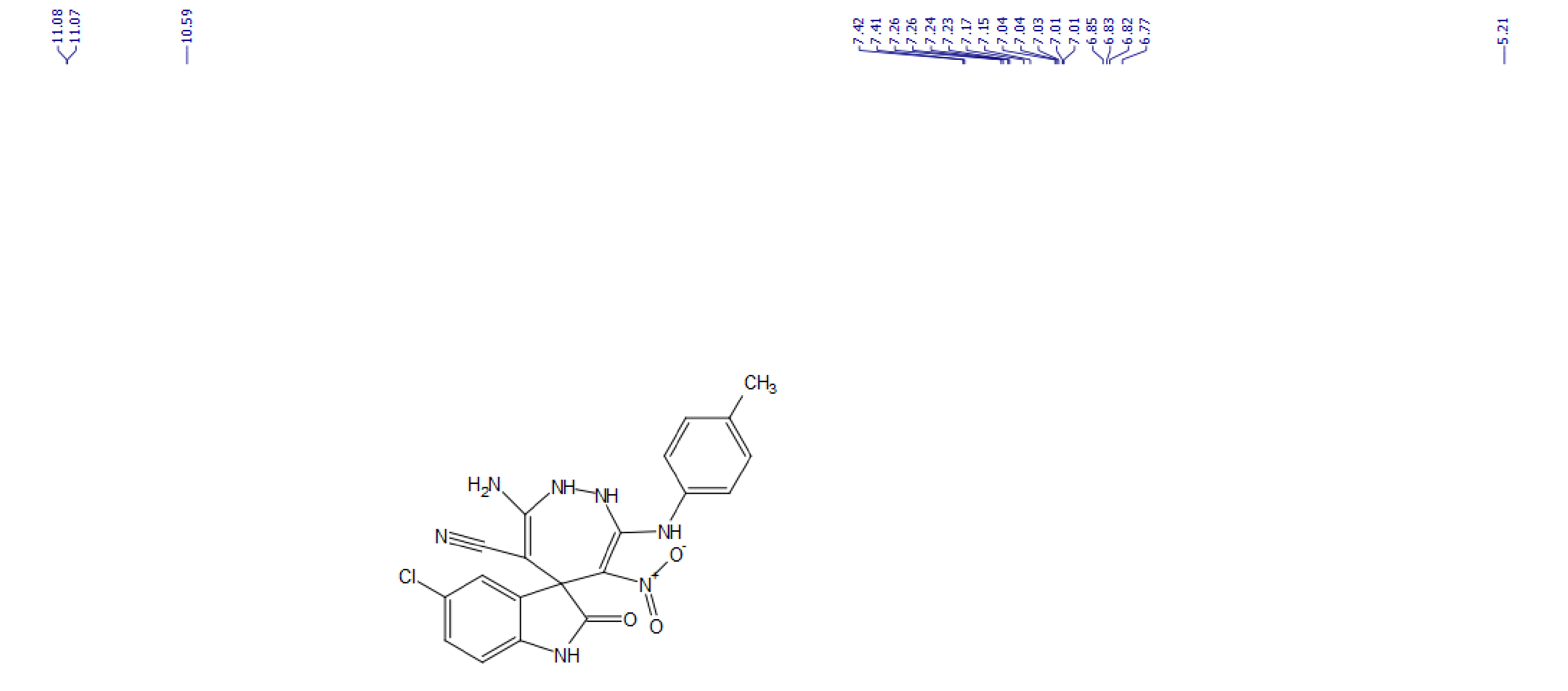
11.08
11.07
10.59
7.42
7.41
7.26
7.26
7.24
7.23
7.17
7.15
7.04
7.04
7.03
7.01
7.01
6.85
6.83
6.82
6.77
5.21
H_2N
NH
NH
NH
N
Cl
N^+
O^-
O
O
NH
CH_3
1.00
1.03
1.05
1.03
2.01
2.15
1.02
2.04
2.02
11.2
11.0
10.8
10.6
10.4
10.2
10.0
9.8
9.6
9.4
9.2
9.0
8.8
8.6
8.4
8.2
8.0
7.8
7.6
7.4
7.2
7.0
6.8
6.6
6.4
6.2
6.0
5.8
5.6
5.4
5.2
5.0
f1 (ppm)

(5a)

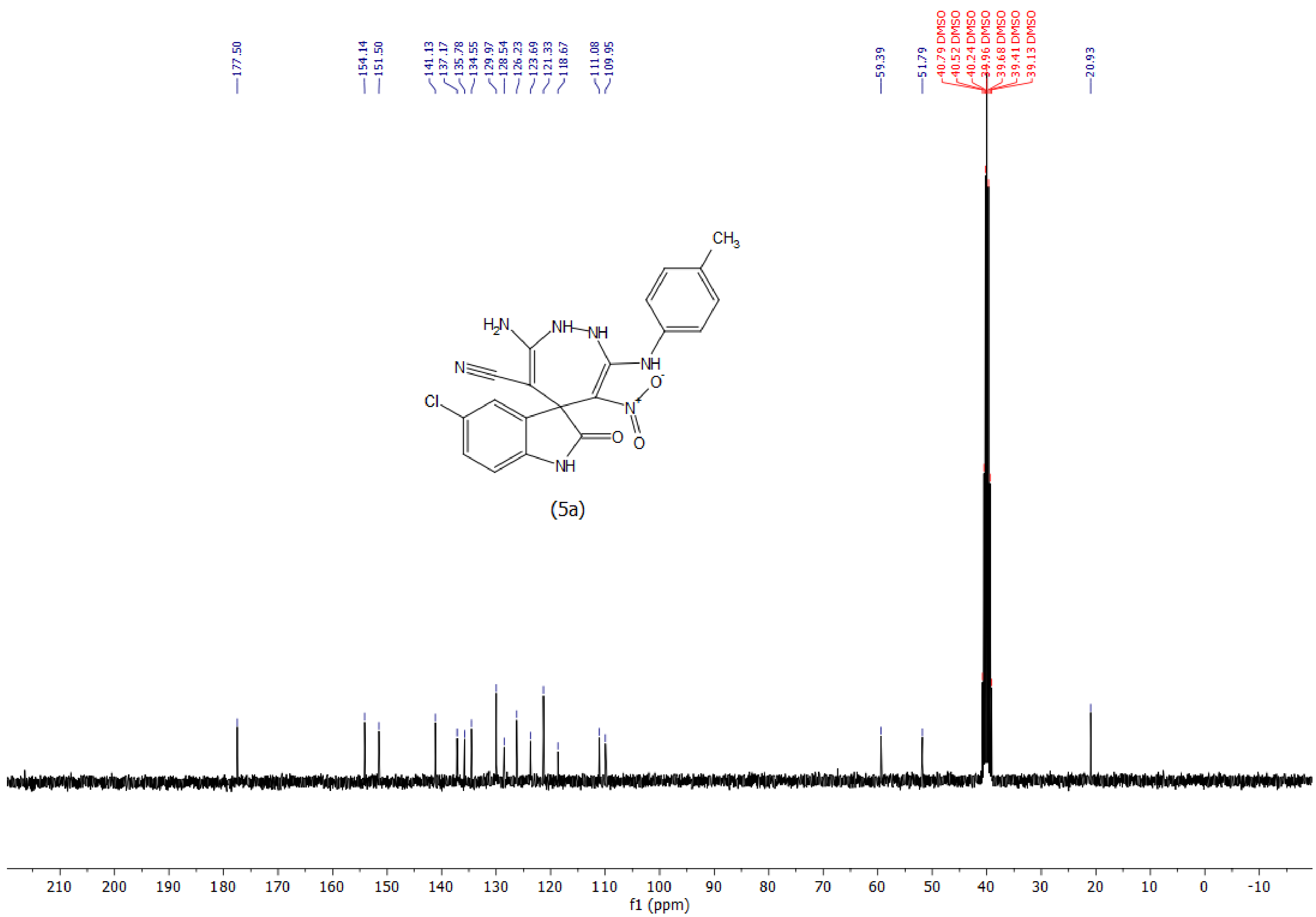
177.50
154.14
151.50
141.13
137.17
135.78
134.55
129.97
128.54
126.23
123.69
121.33
118.67
111.08
109.95
59.39
51.79
40.79 DMSO
40.52 DMSO
40.24 DMSO
39.96 DMSO
39.68 DMSO
39.41 DMSO
39.13 DMSO
20.93
H2N
NH
NH
NH
N
O-
N+
O
CH3
Cl
O
NH
(5a)
f1 (ppm)

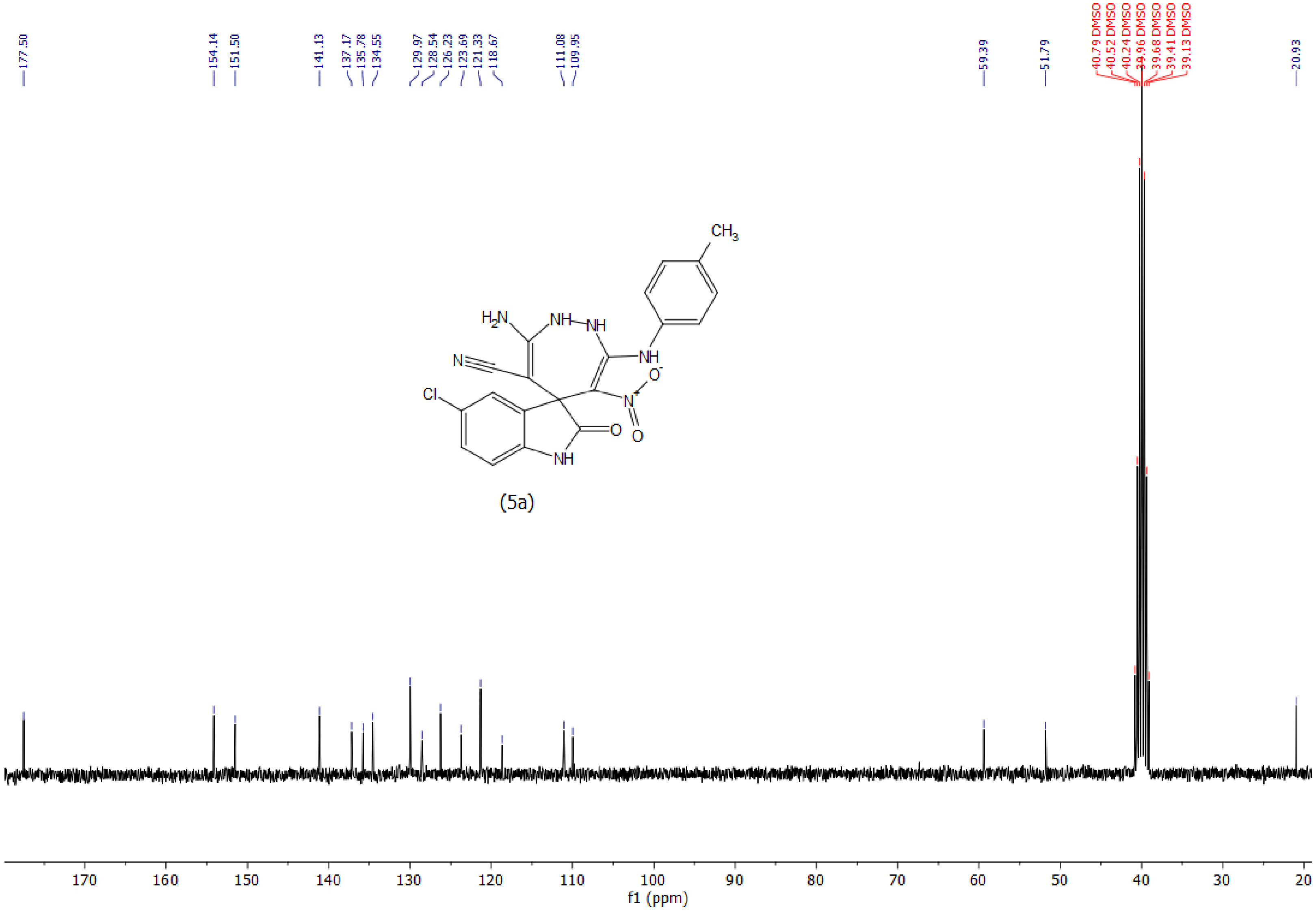
177.50
154.14
151.50
141.13
137.17
135.78
134.55
129.97
128.54
126.23
123.69
121.33
118.67
111.08
109.95
59.39
51.79
40.79 DMSO
40.52 DMSO
40.24 DMSO
39.96 DMSO
39.68 DMSO
39.41 DMSO
39.13 DMSO
20.93
H_2N
NH
NH
NH
N
Cl
O^-
N^+
O
O
NH
CH_3
(5a)
170 160 150 140 130 120 110 100 90 80 70 60 50 40 30 20
f1 (ppm)

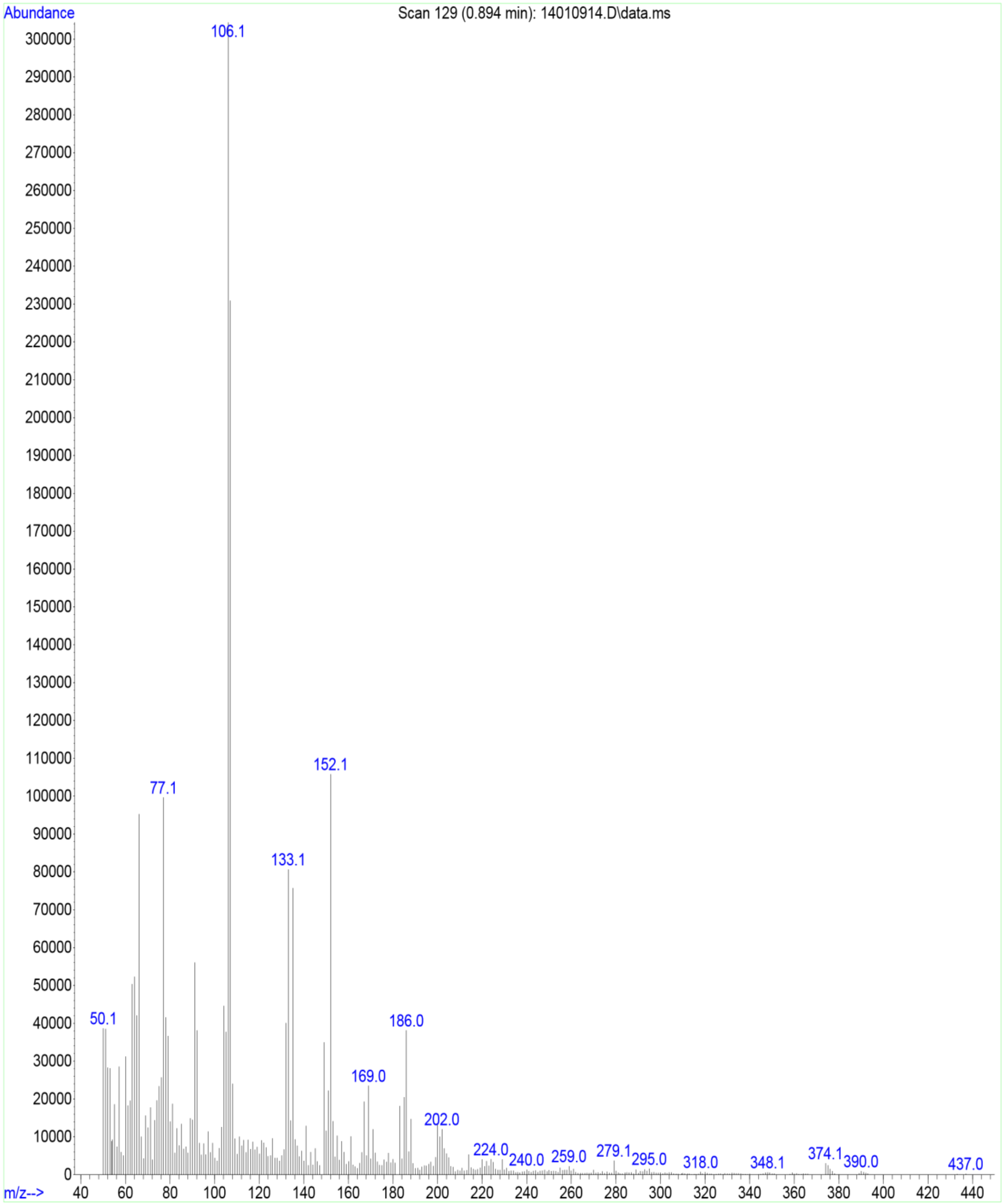

$C_{20}H_{16}ClN_7O_3$ **(5a)**

(437)

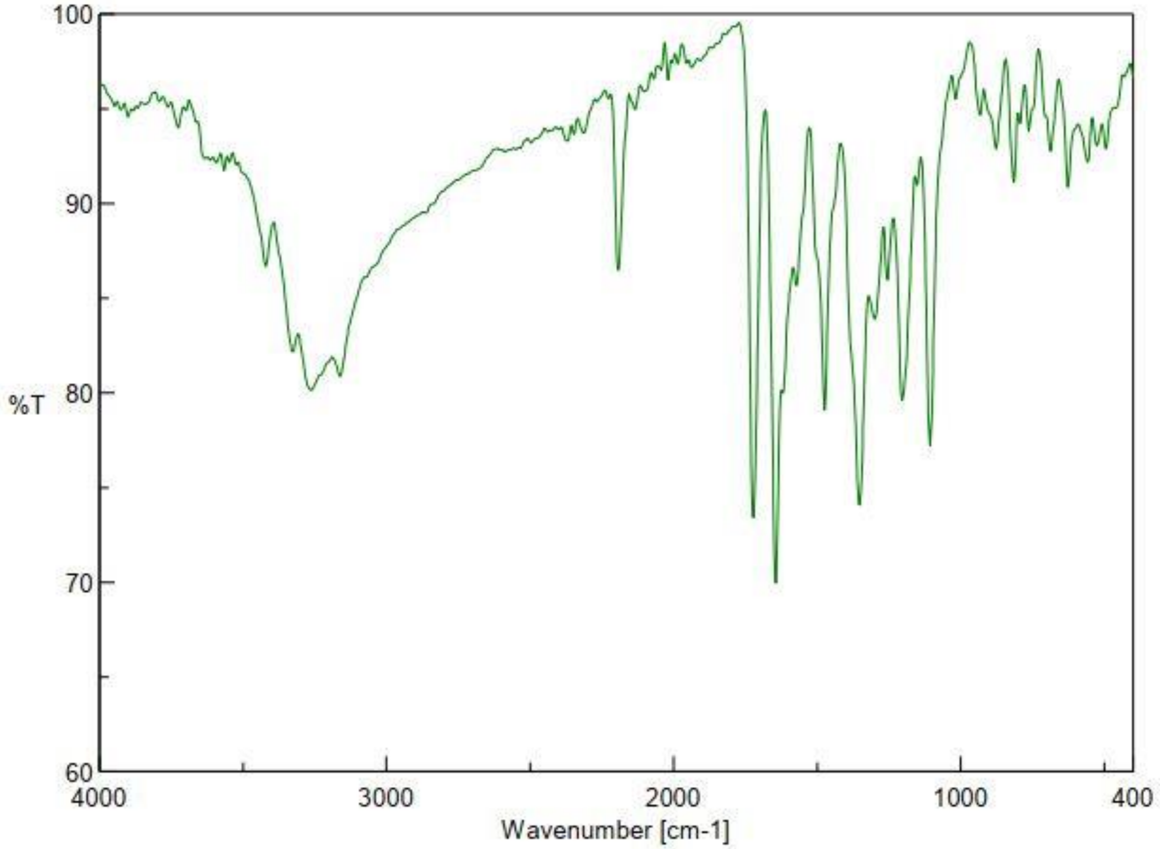

(5a)

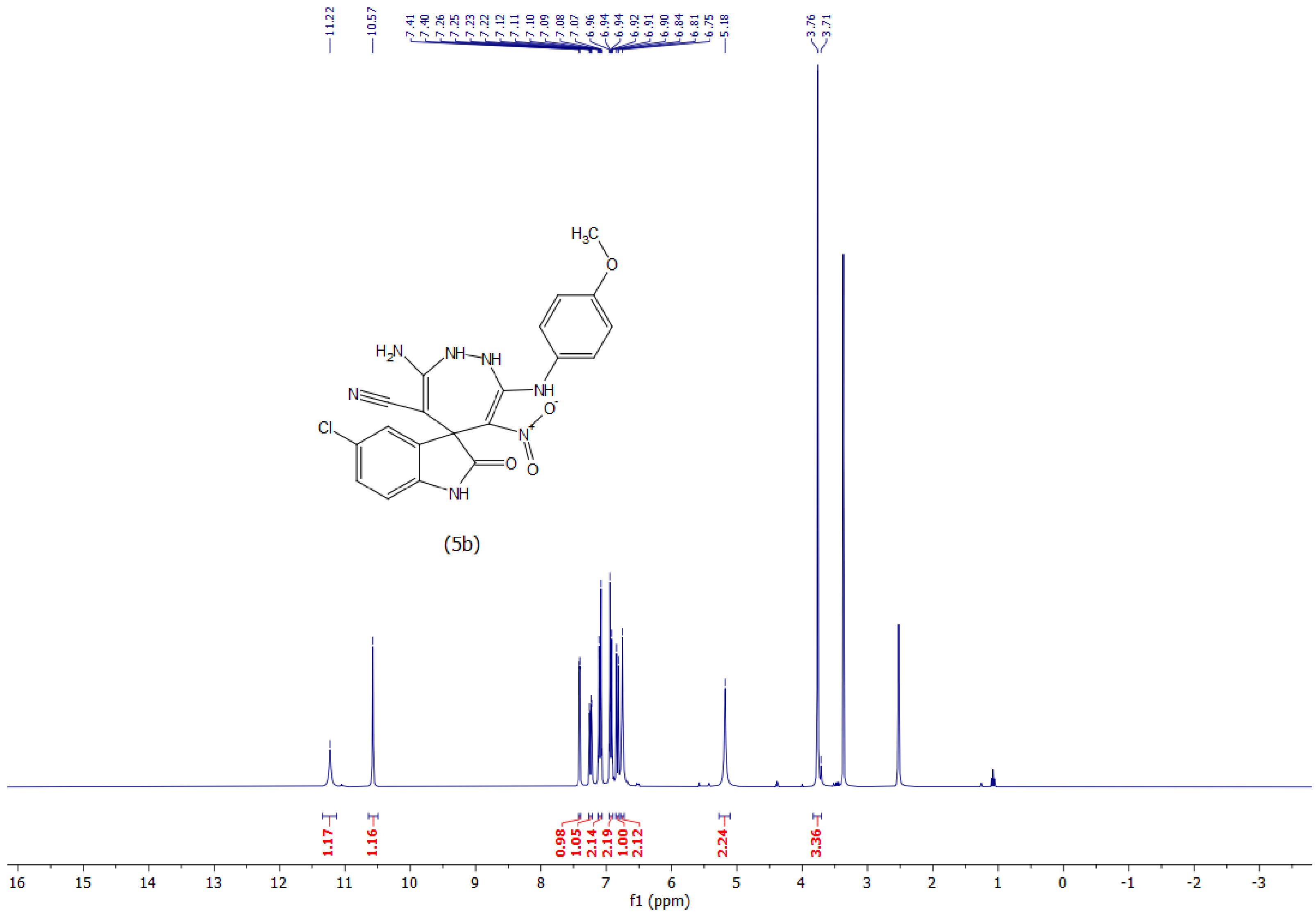
11.22
10.57
7.41
7.40
7.26
7.25
7.23
7.22
7.12
7.11
7.10
7.09
7.08
7.07
6.96
6.94
6.94
6.92
6.91
6.90
6.84
6.81
6.75
5.18
3.76
3.71
(5b)
1.17
1.16
0.98
1.05
2.14
2.19
1.00
2.12
2.24
3.36
16
15
14
13
12
11
10
9
8
7
6
5
4
3
2
1
0
-1
-2
-3
f1 (ppm)

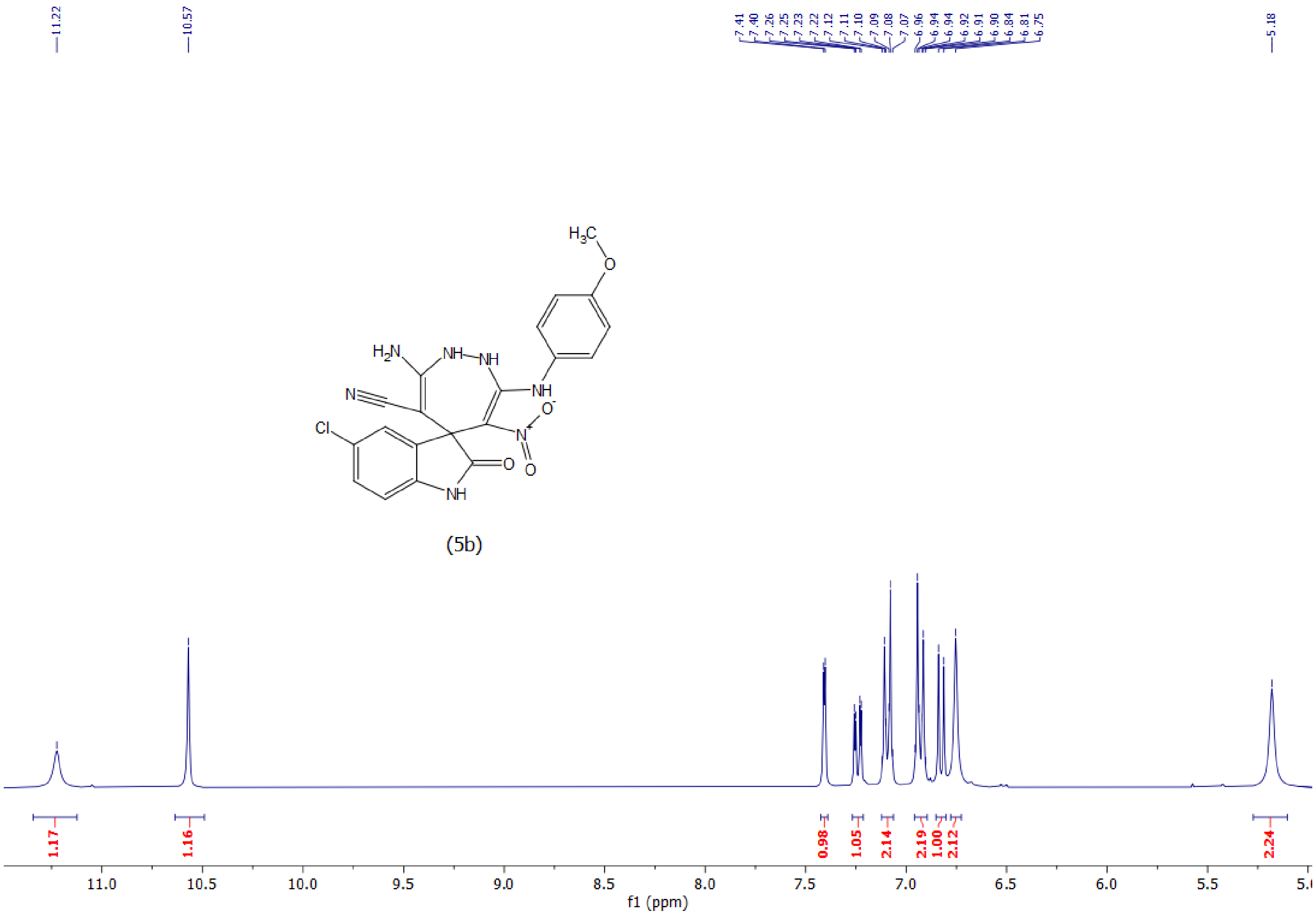
11.22
10.57
7.41
7.40
7.26
7.25
7.23
7.22
7.12
7.11
7.10
7.09
7.08
7.07
6.96
6.94
6.94
6.92
6.91
6.90
6.84
6.81
6.75
5.18
H_3C
O
H_2N
NH
NH
NH
N
Cl
O^-
N^+
O
O
NH
1.17
1.16
0.98
1.05
2.14
2.19
1.00
2.12
2.24
11.0
10.5
10.0
9.5
9.0
8.5
8.0
7.5
7.0
6.5
6.0
5.5
f1 (ppm)

(5b)

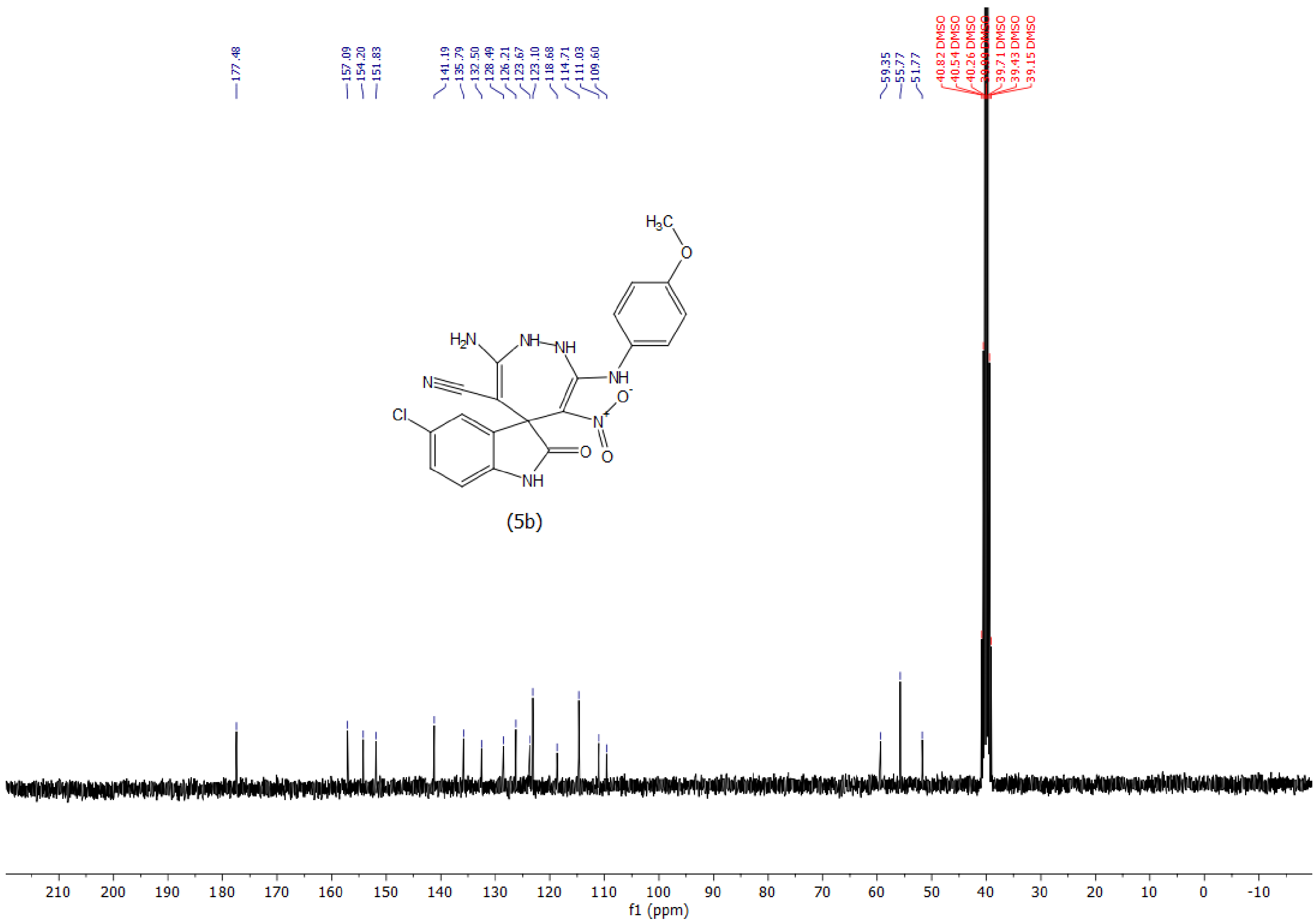
177.48
157.09
154.20
151.83
141.19
135.79
132.50
128.49
126.21
123.67
123.10
118.68
114.71
111.03
109.60
59.35
55.77
51.77
40.82 DMSO
40.54 DMSO
40.26 DMSO
39.99 DMSO
39.71 DMSO
39.43 DMSO
39.15 DMSO
H3C
O
NH
O-
N+
O
NH
NH
H2N
N
Cl
O
NH
(5b)
210 200 190 180 170 160 150 140 130 120 110 100 90 80 70 60 50 40 30 20 10 0 -10
f1 (ppm)

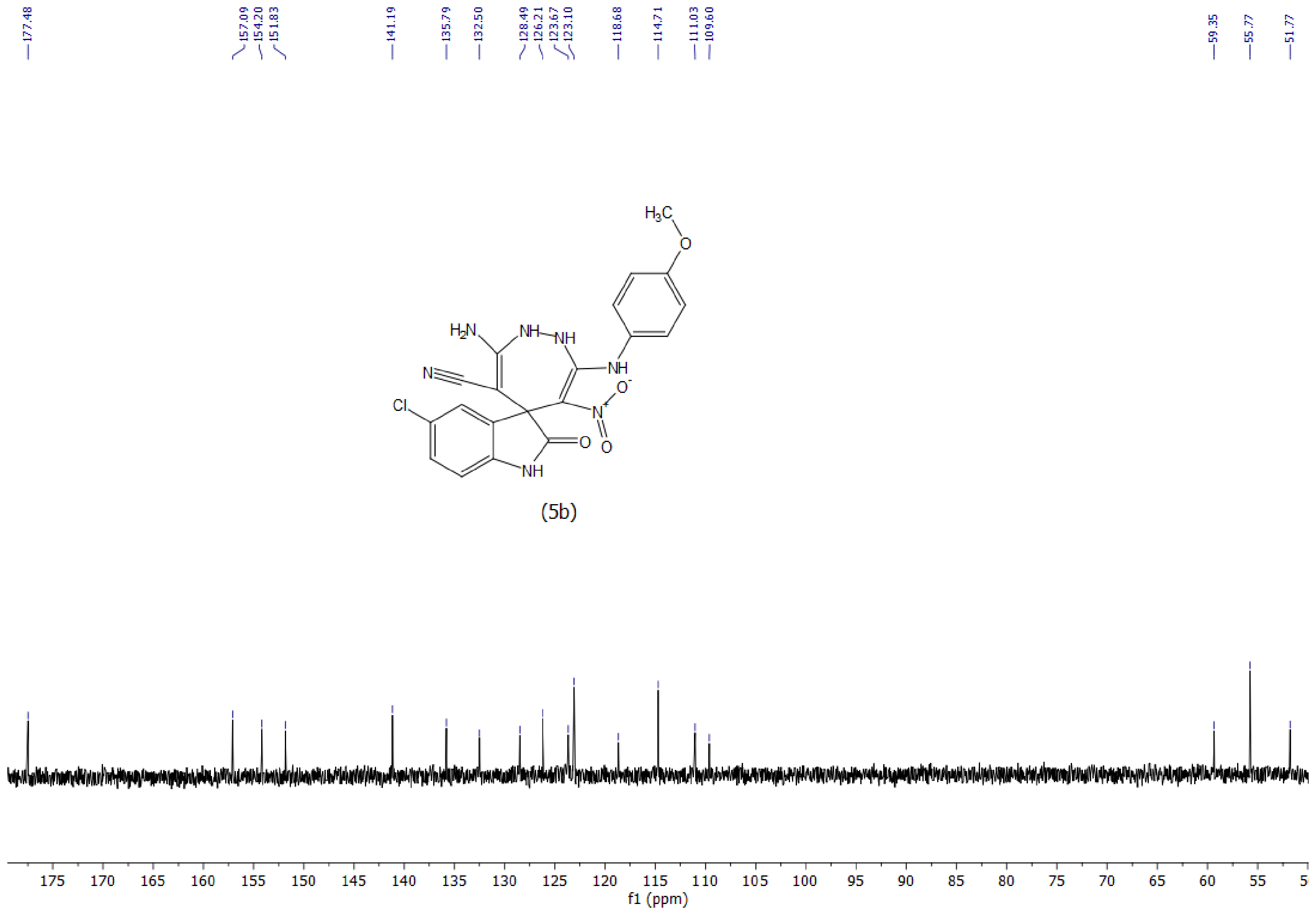
177.48
157.09
154.20
151.83
141.19
135.79
132.50
128.49
126.21
123.67
123.10
118.68
114.71
111.03
109.60
59.35
55.77
51.77
H3C
O
H2N
NH
NH
N
NH
O-
N+
O
Cl
O
NH
(5b)
175 170 165 160 155 150 145 140 135 130 125 120 115 110 105 100 95 90 85 80 75 70 65 60 55 5
f1 (ppm)

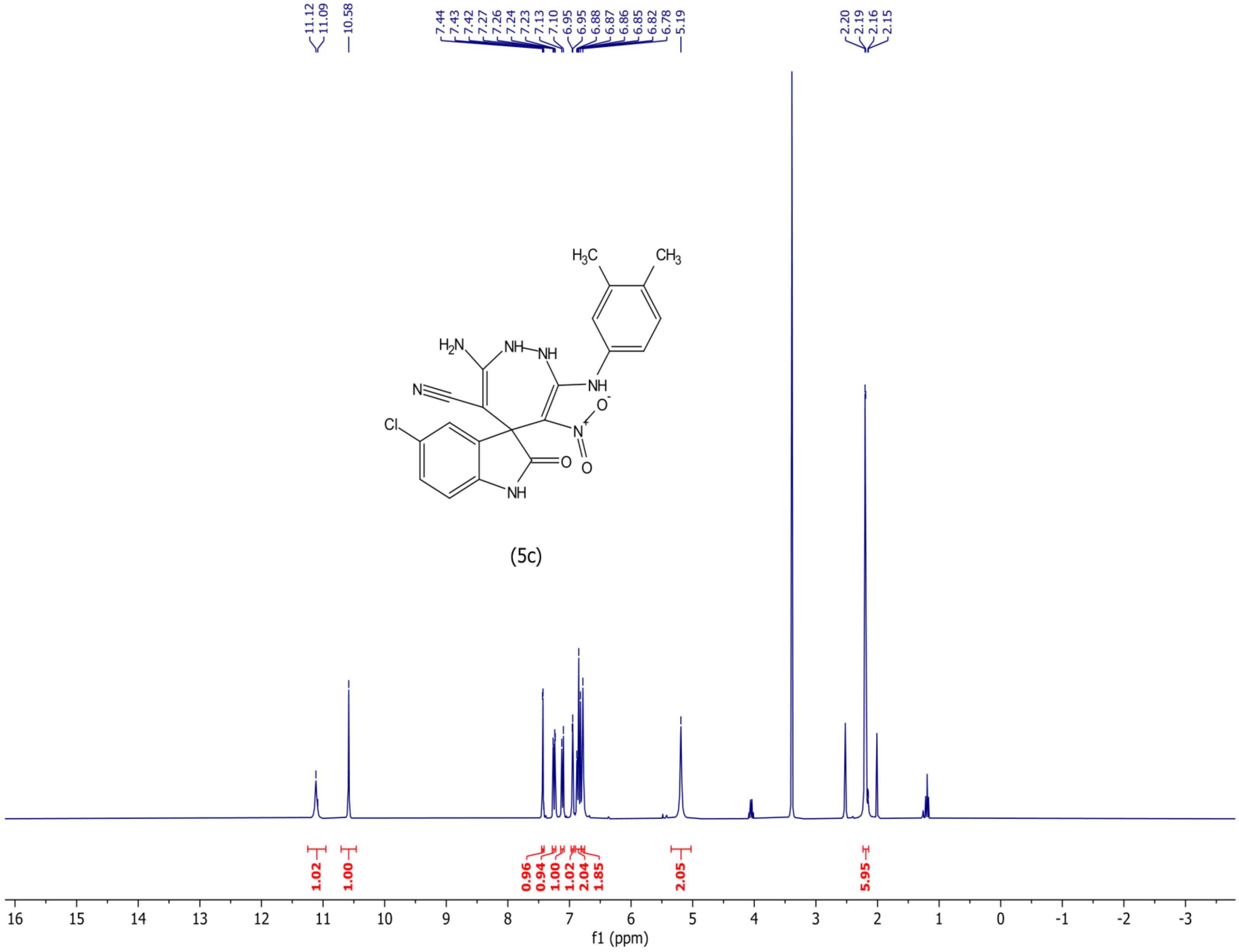
11.12
11.09
10.58
7.44
7.43
7.42
7.27
7.26
7.24
7.23
7.13
7.10
6.95
6.95
6.88
6.87
6.86
6.85
6.82
6.78
5.19
2.20
2.19
2.16
2.15
H_3C
CH_3
H_2N
NH
NH
NH
N
Cl
O^-
N^+
O
O
NH
1.02
1.00
0.96
0.94
1.00
1.02
2.04
1.85
2.05
5.95
16
15
14
13
12
11
10
9
8
7
6
5
4
3
2
1
0
-1
-2
-3
f1 (ppm)

(5c)

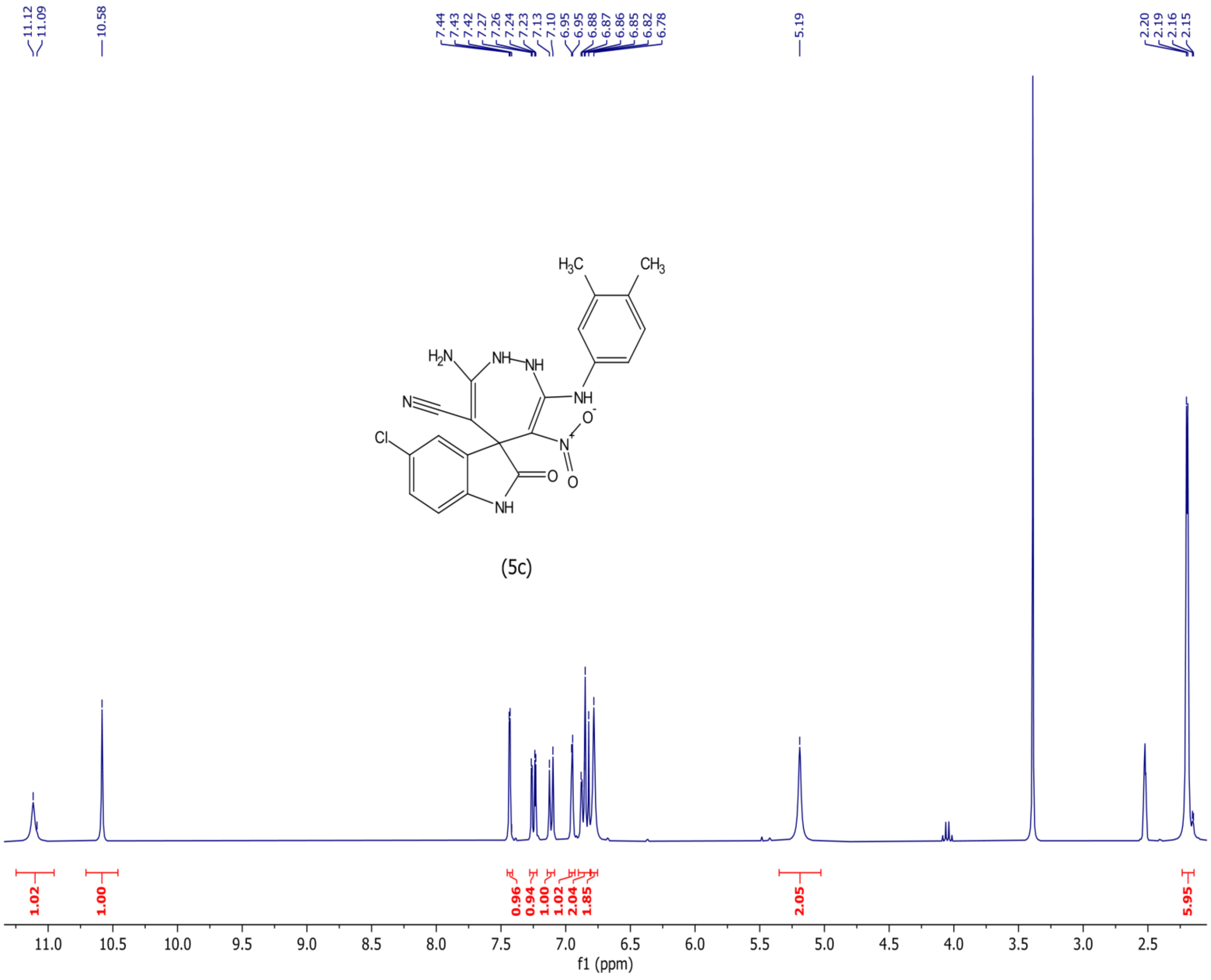
11.12
11.09
10.58
7.44
7.43
7.42
7.27
7.26
7.24
7.23
7.13
7.10
6.95
6.95
6.88
6.87
6.86
6.85
6.82
6.78
5.19
2.20
2.19
2.16
2.15
H$_2$N
NH
NH
NH
N
Cl
H$_3$C
CH$_3$
O$^-$
N$^+$
O
O
NH
1.02
1.00
0.96
0.94
1.00
1.02
2.04
1.85
2.05
5.95
11.0
10.5
10.0
9.5
9.0
8.5
8.0
7.5
7.0
6.5
6.0
5.5
5.0
4.5
4.0
3.5
3.0
2.5
f1 (ppm)

(5c)

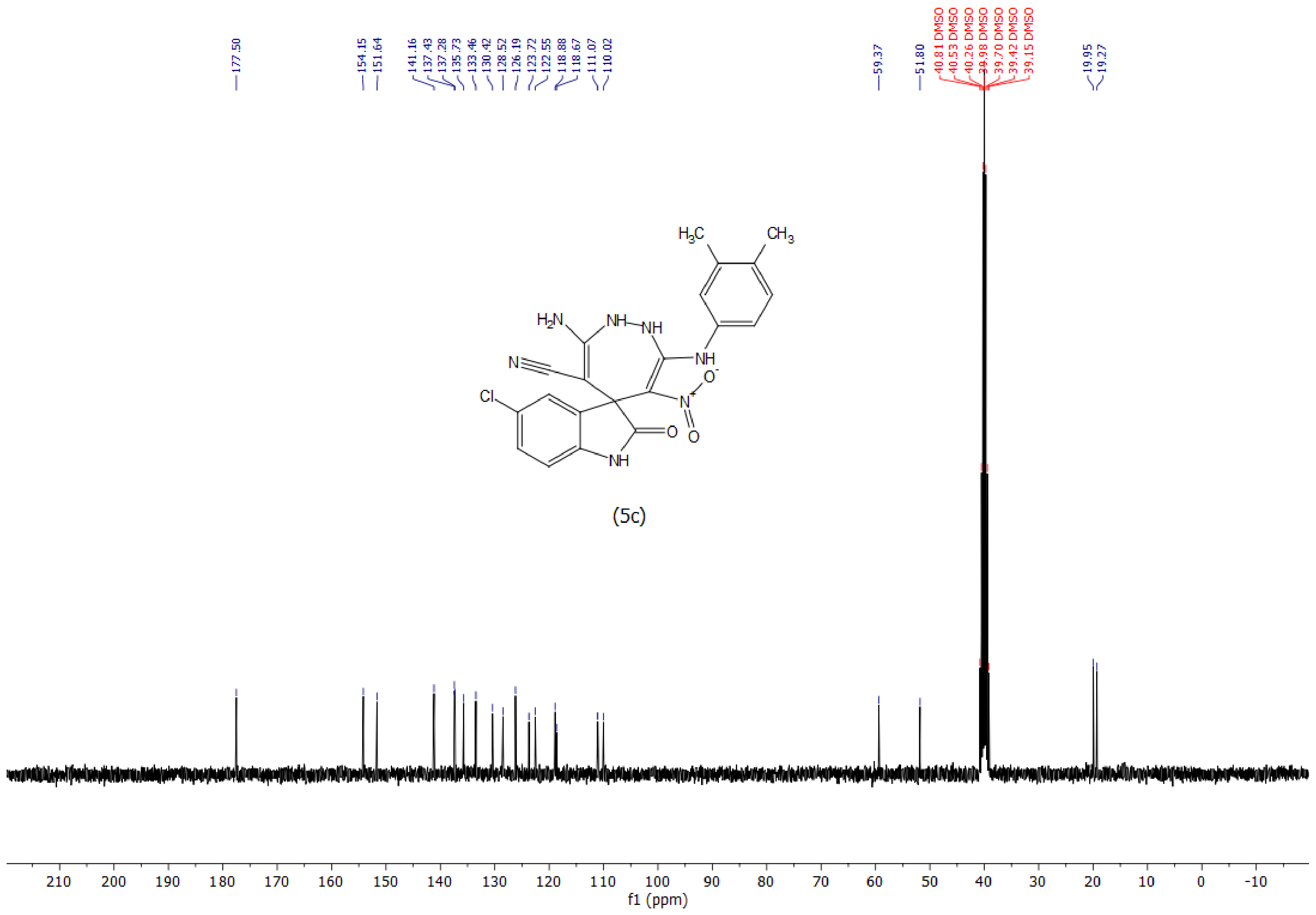

177.50
154.15
151.64
141.16
137.43
137.28
135.73
133.46
130.42
128.52
126.19
123.72
122.55
118.88
118.67
111.07
110.02
59.37
51.80
40.81 DMSO
40.53 DMSO
40.26 DMSO
39.98 DMSO
39.70 DMSO
39.42 DMSO
39.15 DMSO
19.95
19.27
H3C
CH3
H2N
NH
NH
NH
N
O-
N+
O
Cl
O
NH
210 200 190 180 170 160 150 140 130 120 110 100 90 80 70 60 50 40 30 20 10 0 -10
f1 (ppm)

(5c)

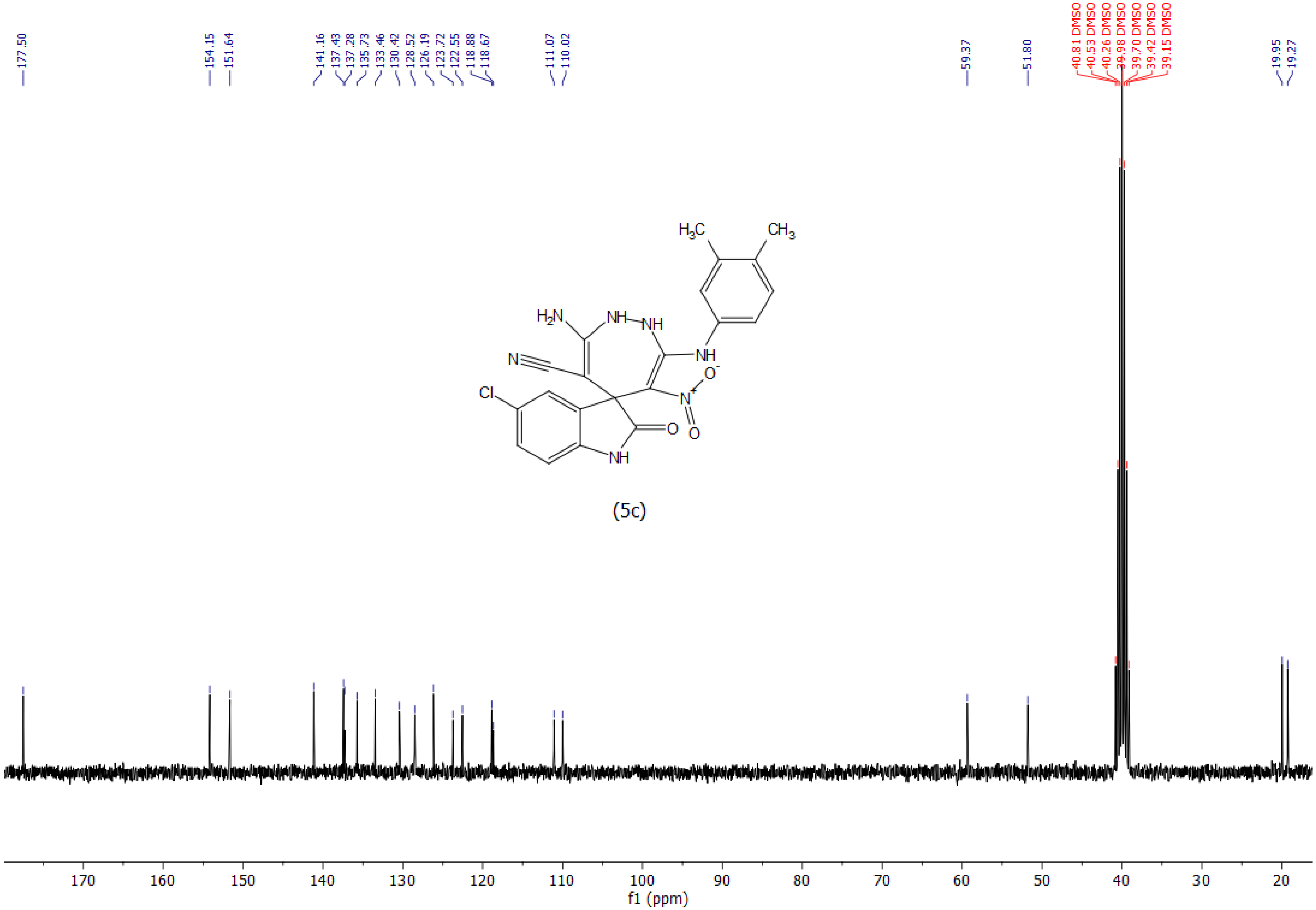
177.50
154.15
151.64
141.16
137.43
137.28
135.73
133.46
130.42
128.52
126.19
123.72
122.55
118.88
118.67
111.07
110.02
59.37
51.80
40.81 DMSO
40.53 DMSO
40.26 DMSO
39.98 DMSO
39.70 DMSO
39.42 DMSO
39.15 DMSO
19.95
19.27
H3C
CH3
H2N
NH
NH
NH
N
O-
N+
O
Cl
O
NH
f1 (ppm)

(5c)

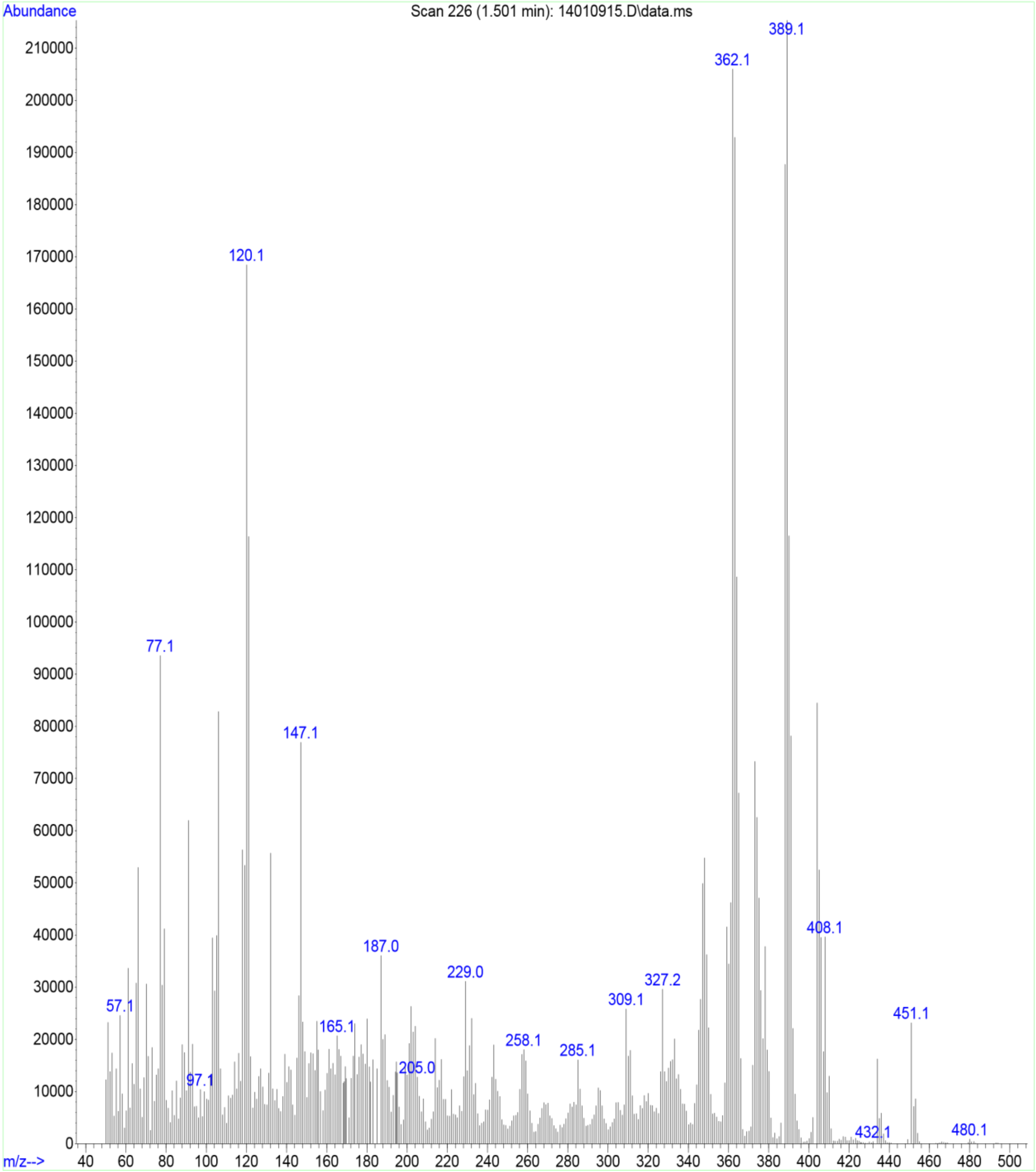

$C_{21}H_{18}ClN_7O_3$ **(5c)**

(451)

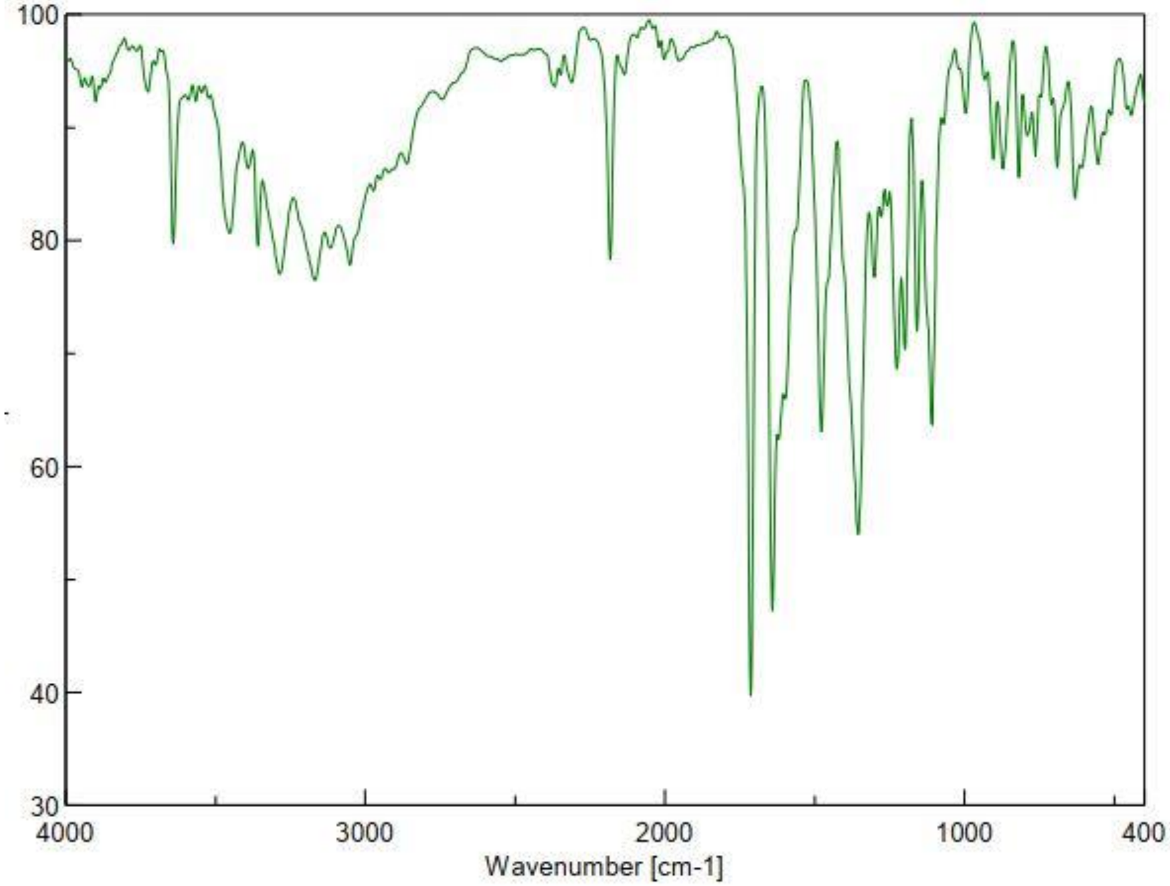

(5c)

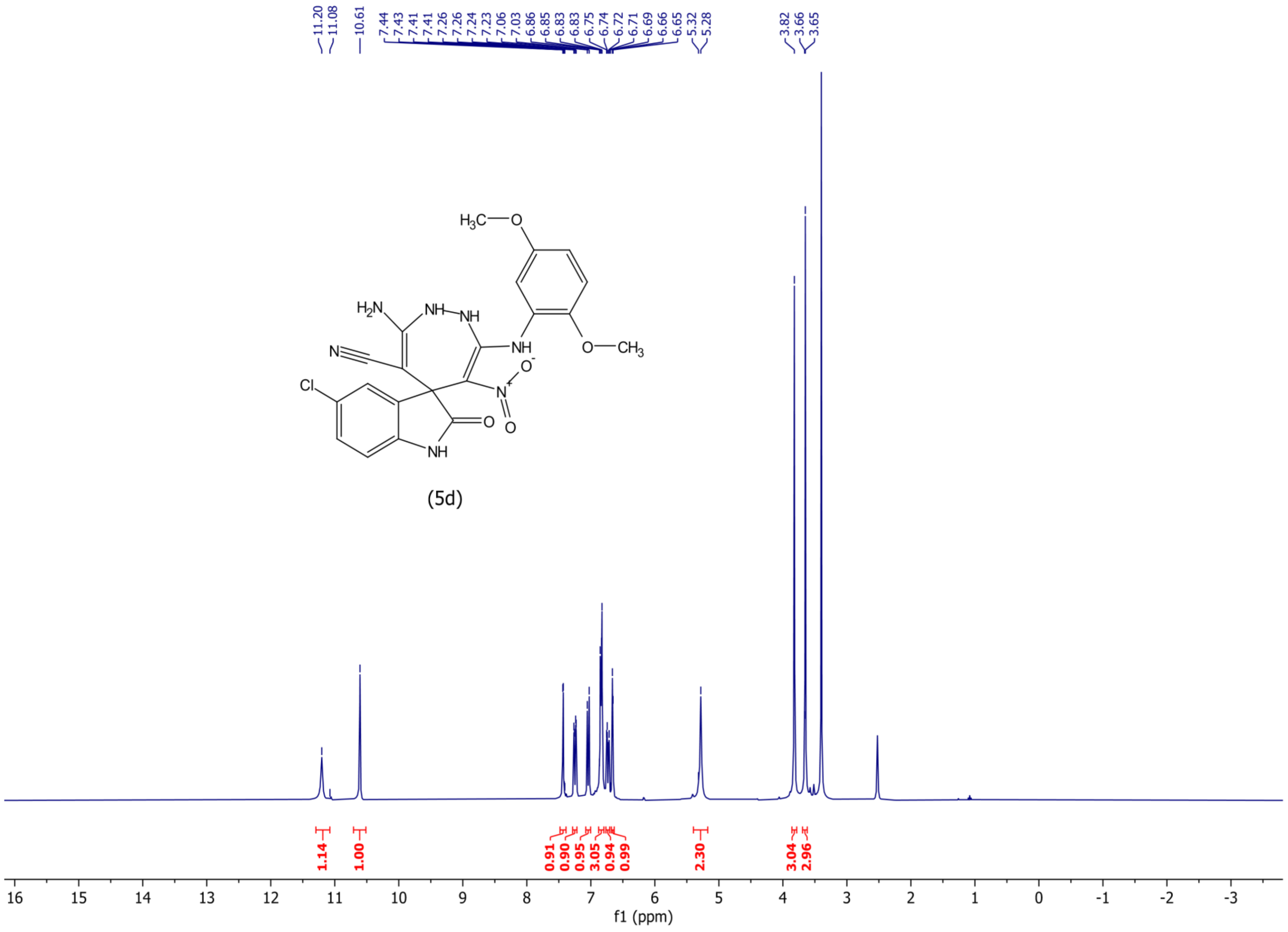
11.20
11.08
10.61
7.44
7.43
7.41
7.41
7.26
7.26
7.24
7.23
7.06
7.03
6.86
6.85
6.83
6.83
6.75
6.74
6.72
6.71
6.69
6.66
6.65
5.32
5.28
3.82
3.66
3.65
H3C
O
H2N
NH
NH
NH
N
O-
N+
O
CH3
Cl
O
NH
(5d)
1.14
1.00
0.91
0.90
0.95
3.05
0.94
0.99
2.30
3.04
2.96
16 15 14 13 12 11 10 9 8 7 6 5 4 3 2 1 0 -1 -2 -3
f1 (ppm)

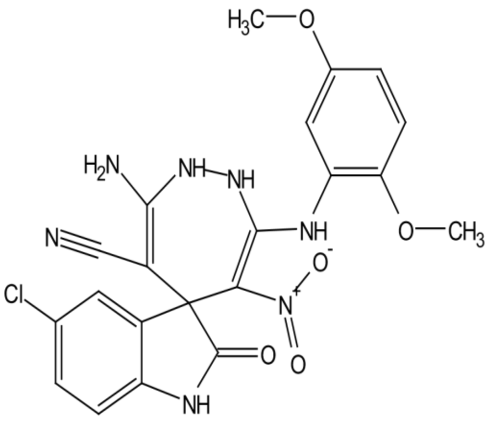

H3C—O
H2N
NH
NH
NH
O—CH3
N
O-
N+
Cl
O
O
NH

(5d)

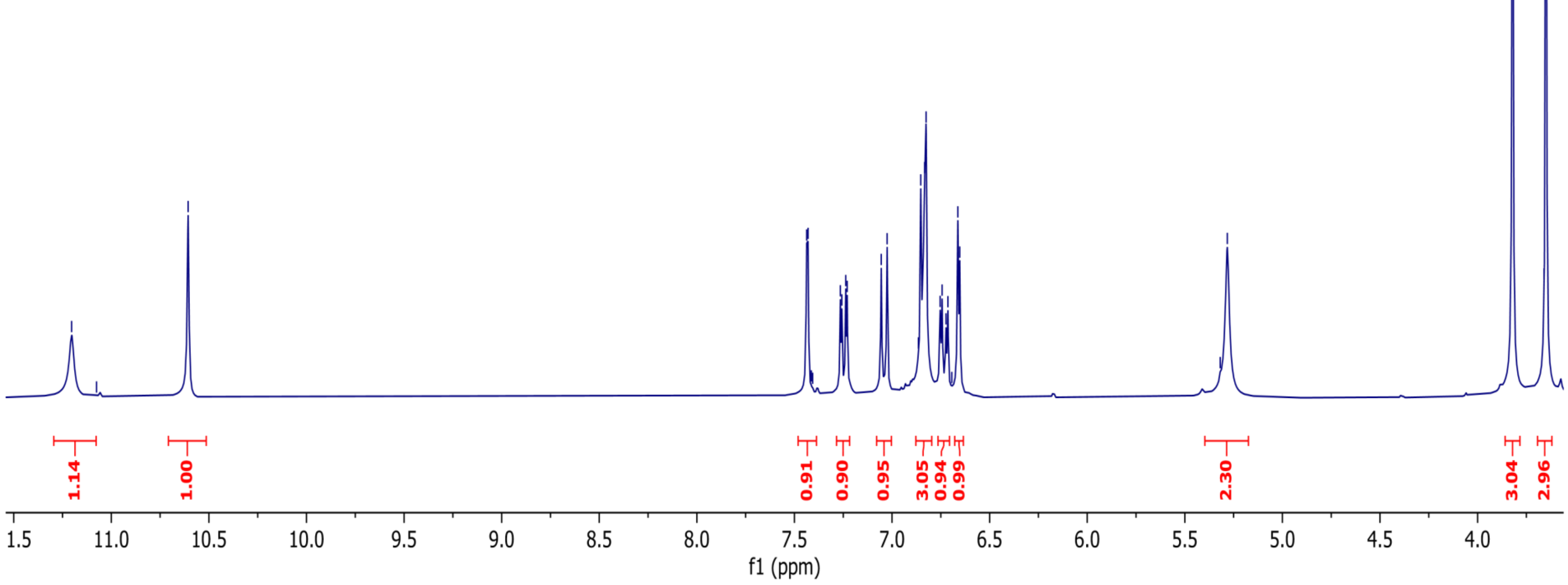

1.14
1.00
0.91
0.90
0.95
3.05
0.94
0.99
2.30
3.04
2.96
1.5
11.0
10.5
10.0
9.5
9.0
8.5
8.0
7.5
7.0
6.5
6.0
5.5
5.0
4.5
4.0
f1 (ppm)

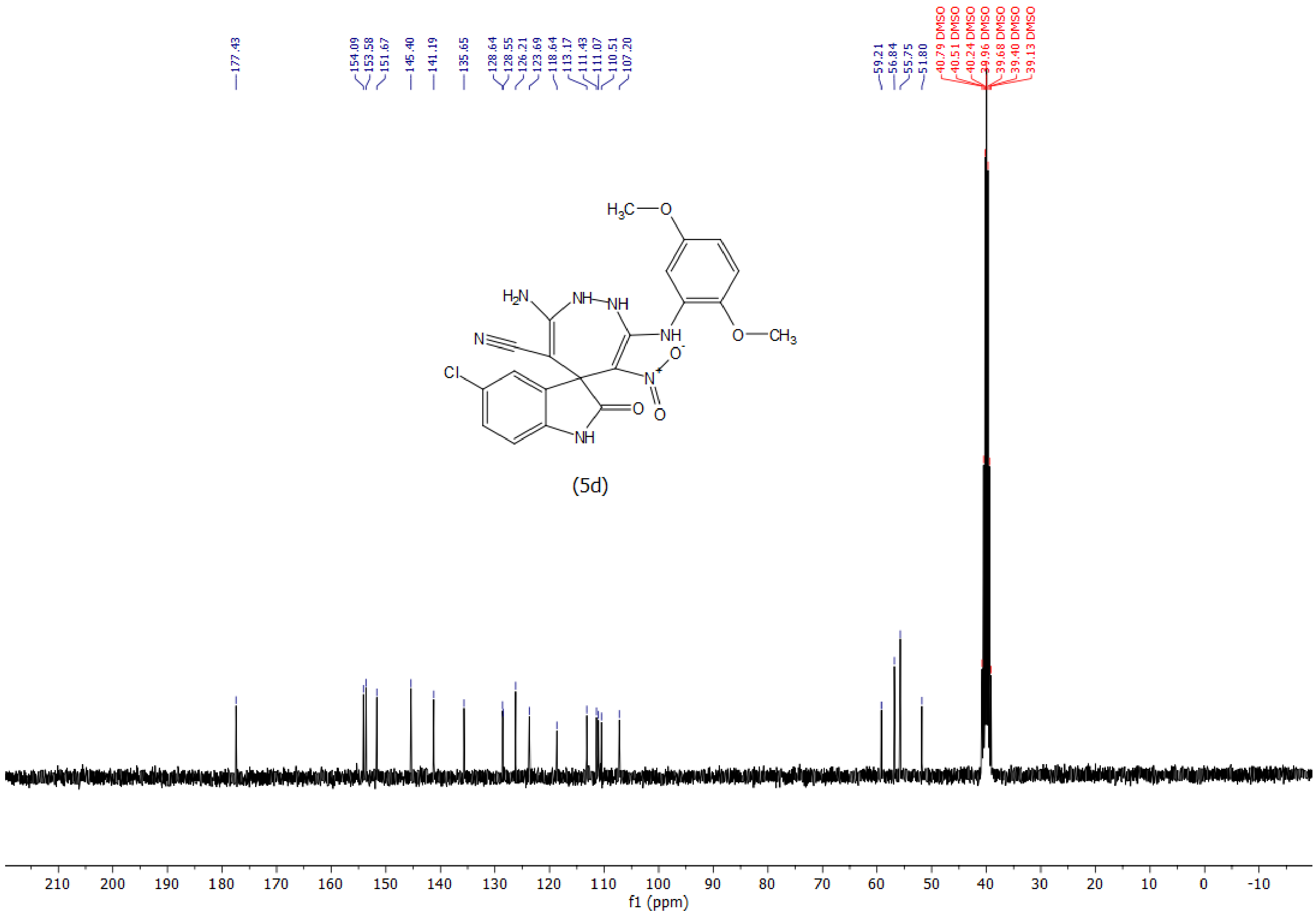
177.43
154.09
153.58
151.67
145.40
141.19
135.65
128.64
128.55
126.21
123.69
118.64
113.17
111.43
111.07
110.51
107.20
59.21
56.84
55.75
51.80
40.79 DMSO
40.51 DMSO
40.24 DMSO
39.96 DMSO
39.68 DMSO
39.40 DMSO
39.13 DMSO
H_3C—O
H_2N
NH—NH
NH
O—CH_3
N
Cl
N^+
O^-
O
O
NH
(5d)
210 200 190 180 170 160 150 140 130 120 110 100 90 80 70 60 50 40 30 20 10 0 -10
f1 (ppm)

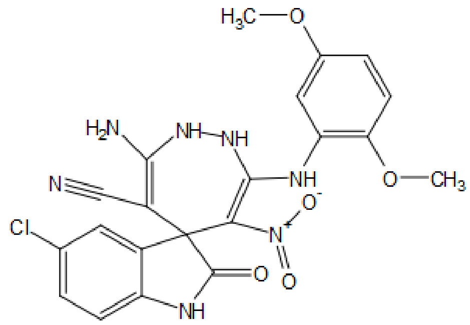

(5d)

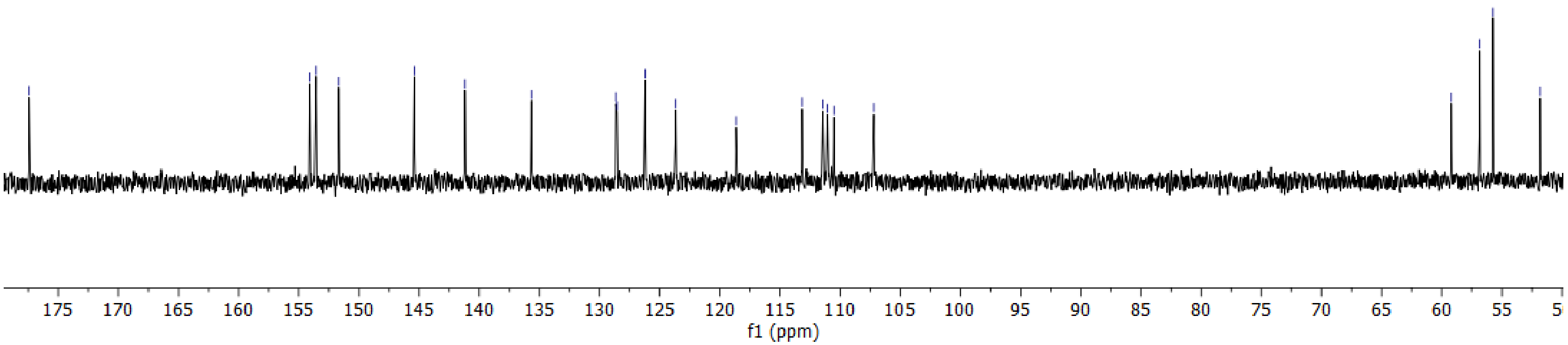

177.43
154.09
153.58
151.67
145.40
141.19
135.65
128.64
128.55
126.21
123.69
118.64
113.17
111.43
111.07
110.51
107.20
59.21
56.84
55.75
51.80
f1 (ppm)

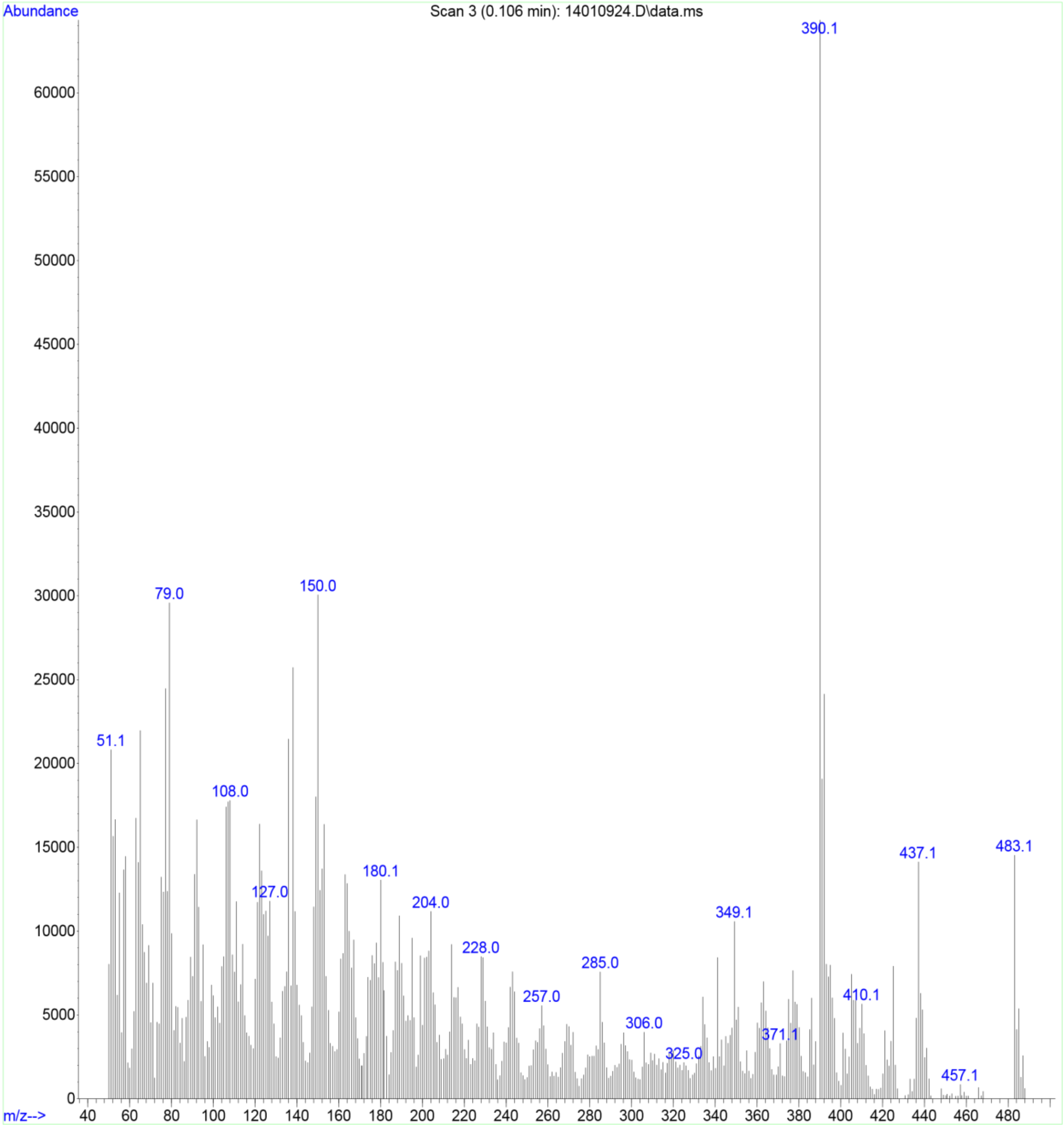

$C_{21}H_{18}ClN_7O_5$ **(5d)**

(483)

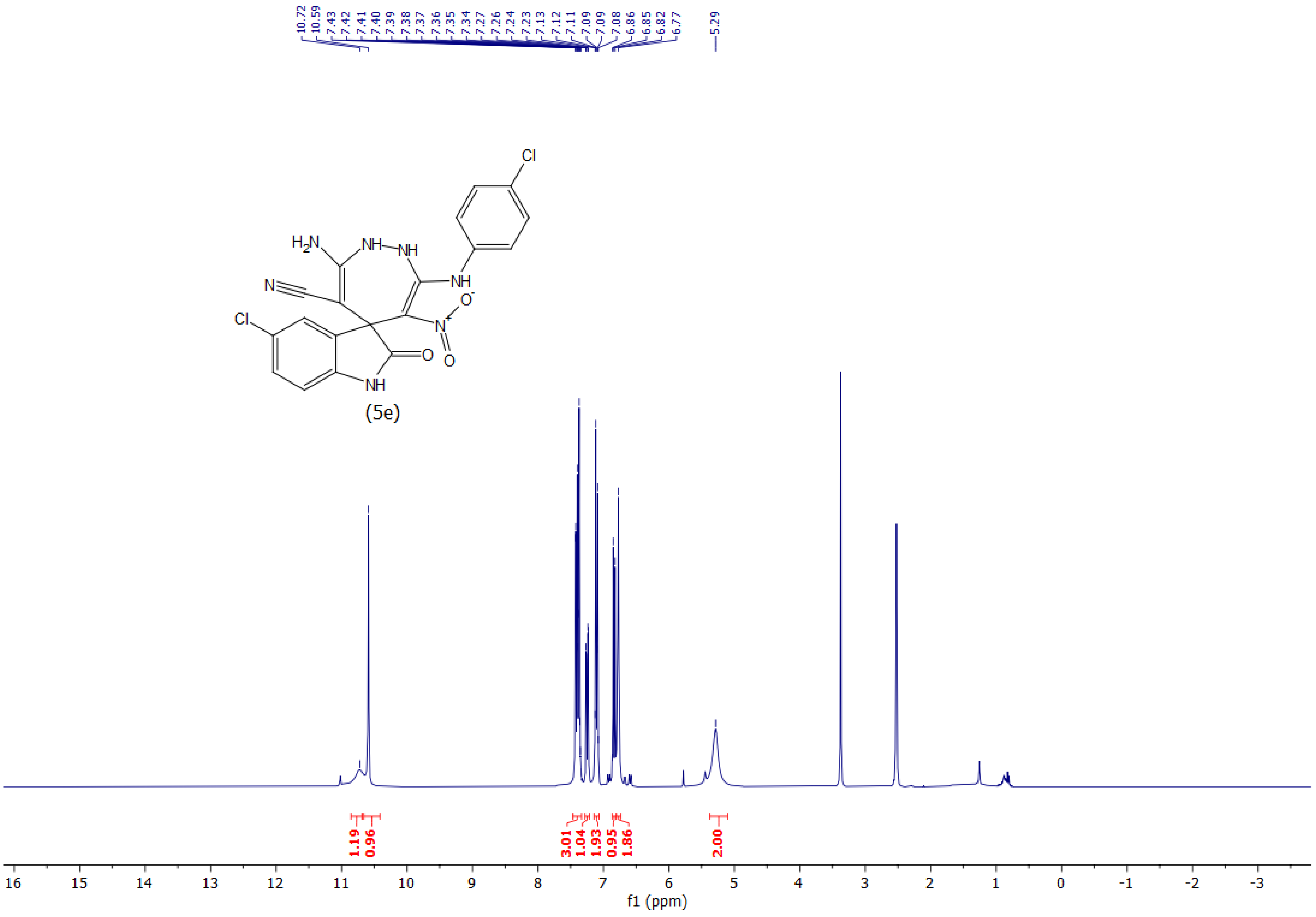
10.72
10.59
7.43
7.42
7.41
7.40
7.39
7.38
7.37
7.36
7.35
7.34
7.27
7.26
7.24
7.23
7.13
7.12
7.11
7.09
7.09
7.08
6.86
6.85
6.82
6.77
5.29
Cl
H_2N
NH
NH
NH
N
O^-
N^+
O
Cl
O
NH
(5e)
1.19
0.96
3.01
1.04
1.93
0.95
1.86
2.00
16
15
14
13
12
11
10
9
8
7
6
5
4
3
2
1
0
-1
-2
-3
f1 (ppm)

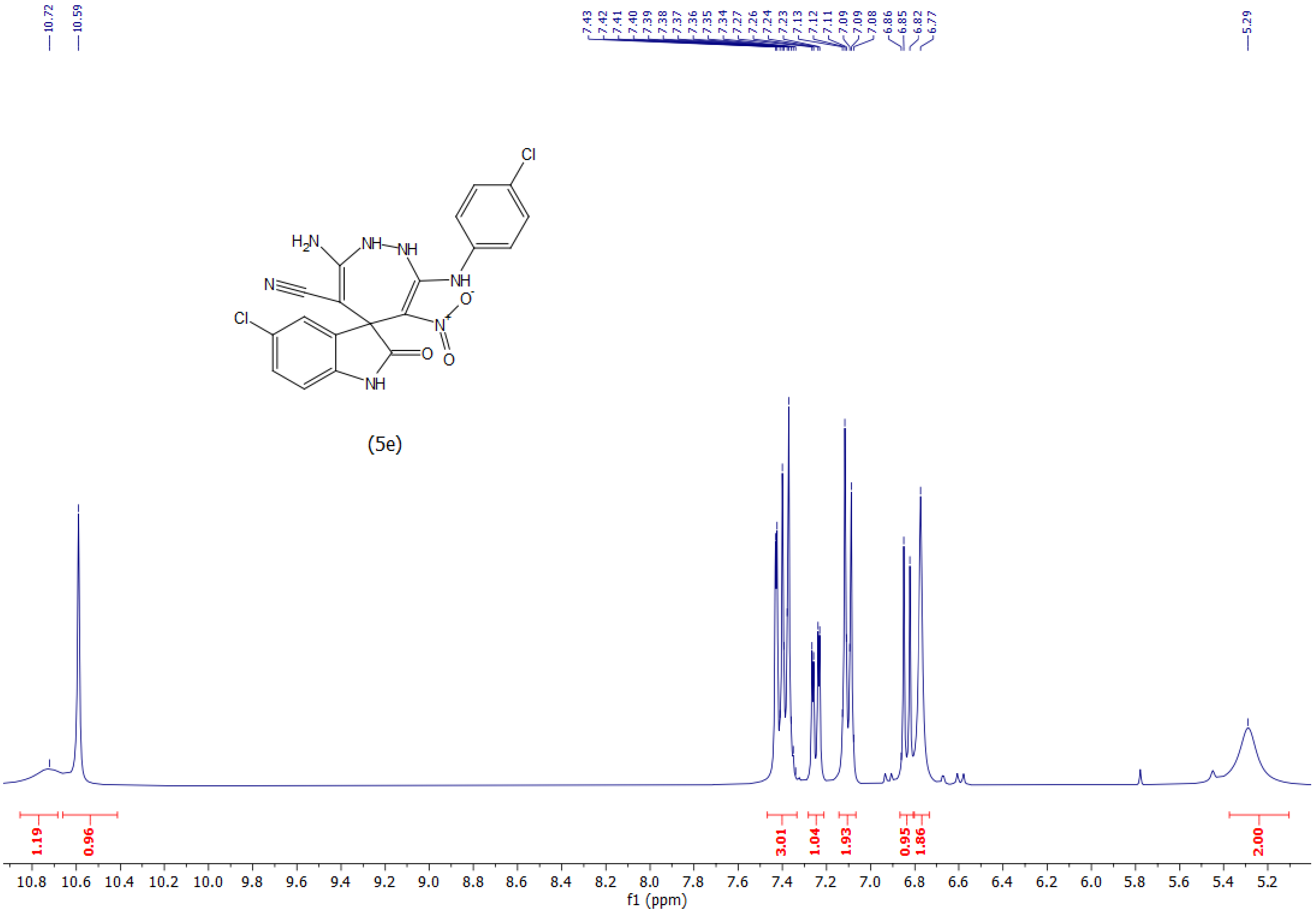

10.72
10.59
7.43
7.42
7.41
7.40
7.39
7.38
7.37
7.36
7.35
7.34
7.27
7.26
7.24
7.23
7.13
7.12
7.11
7.09
7.09
7.08
6.86
6.85
6.82
6.77
5.29
1.19
0.96
3.01
1.04
1.93
0.95
1.86
2.00
10.8
10.6
10.4
10.2
10.0
9.8
9.6
9.4
9.2
9.0
8.8
8.6
8.4
8.2
8.0
7.8
7.6
7.4
7.2
7.0
6.8
6.6
6.4
6.2
6.0
5.8
5.6
5.4
5.2
f1 (ppm)

(5e)

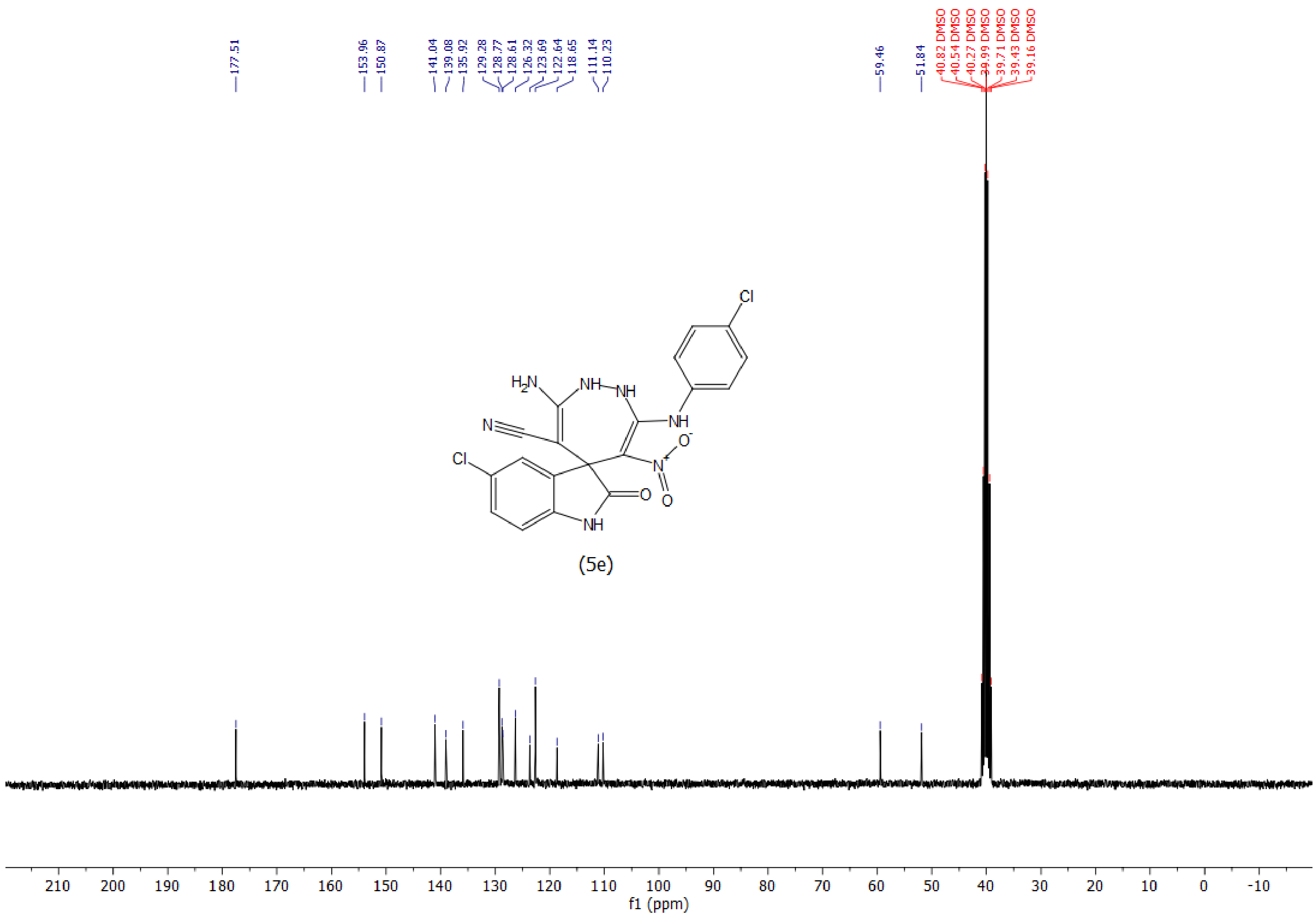
177.51
153.96
150.87
141.04
139.08
135.92
129.28
128.77
128.61
126.32
123.69
122.64
118.65
111.14
110.23
59.46
51.84
40.82 DMSO
40.54 DMSO
40.27 DMSO
39.99 DMSO
39.71 DMSO
39.43 DMSO
39.16 DMSO
Cl
H2N
NH
NH
NH
N
O-
N+
O
Cl
O
NH
(5e)
210
200
190
180
170
160
150
140
130
120
110
100
90
80
70
60
50
40
30
20
10
0
-10
f1 (ppm)

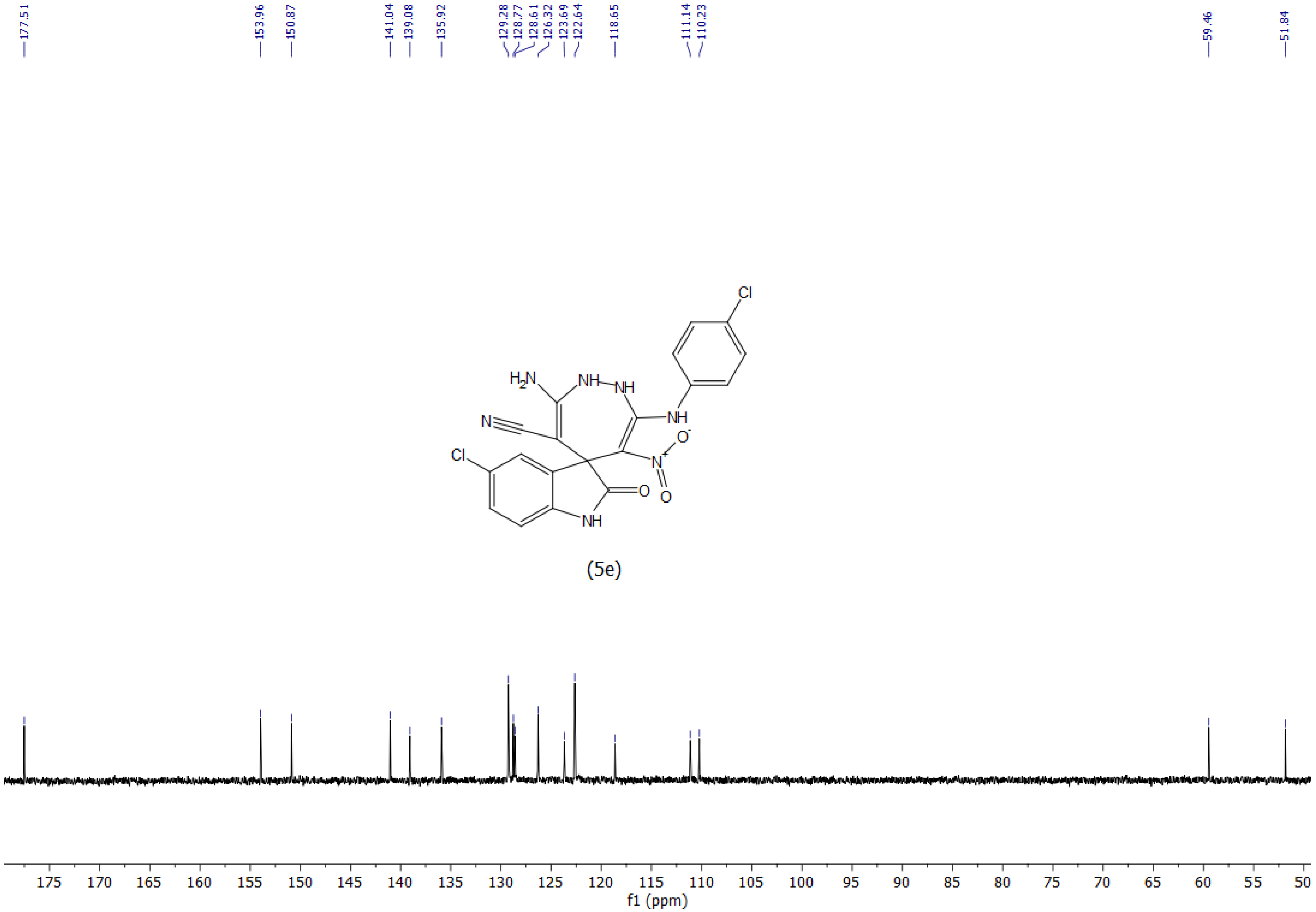
177.51
153.96
150.87
141.04
139.08
135.92
129.28
128.77
128.61
126.32
123.69
122.64
118.65
111.14
110.23
59.46
51.84
H2N
NH
NH
NH
N
Cl
Cl
O
N+
O-
O
NH
175 170 165 160 155 150 145 140 135 130 125 120 115 110 105 100 95 90 85 80 75 70 65 60 55 50
f1 (ppm)

(5e)

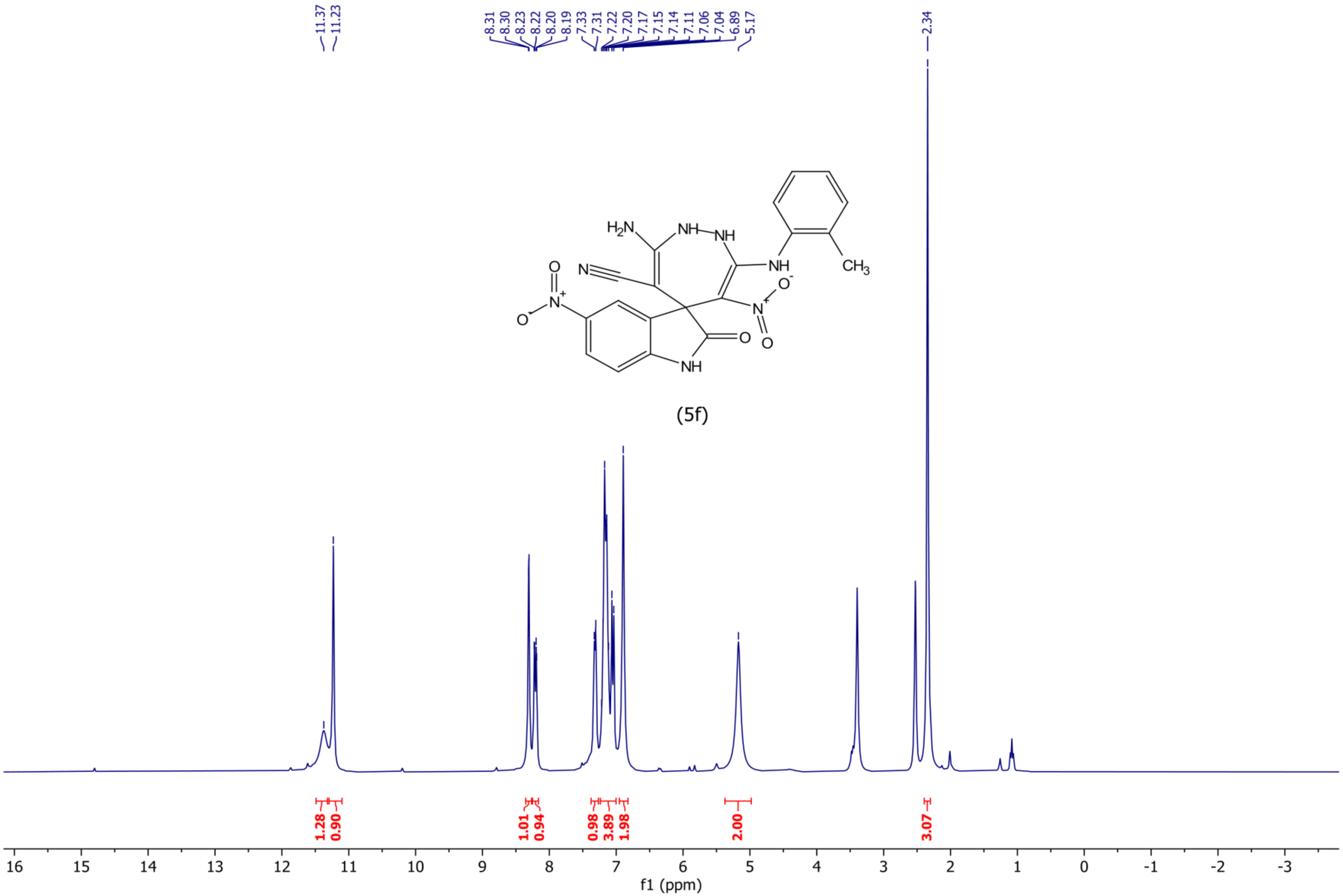

11.37
11.23
8.31
8.30
8.23
8.22
8.20
8.19
7.33
7.31
7.22
7.20
7.17
7.15
7.14
7.11
7.06
7.04
6.89
5.17
2.34
H_2N
NH
NH
NH
N
CH_3
O
N^+
O^-
O^-
N^+
O
O
NH
1.28
0.90
1.01
0.94
0.98
3.89
1.98
2.00
3.07
16
15
14
13
12
11
10
9
8
7
6
5
4
3
2
1
0
-1
-2
-3
f1 (ppm)

(5f)

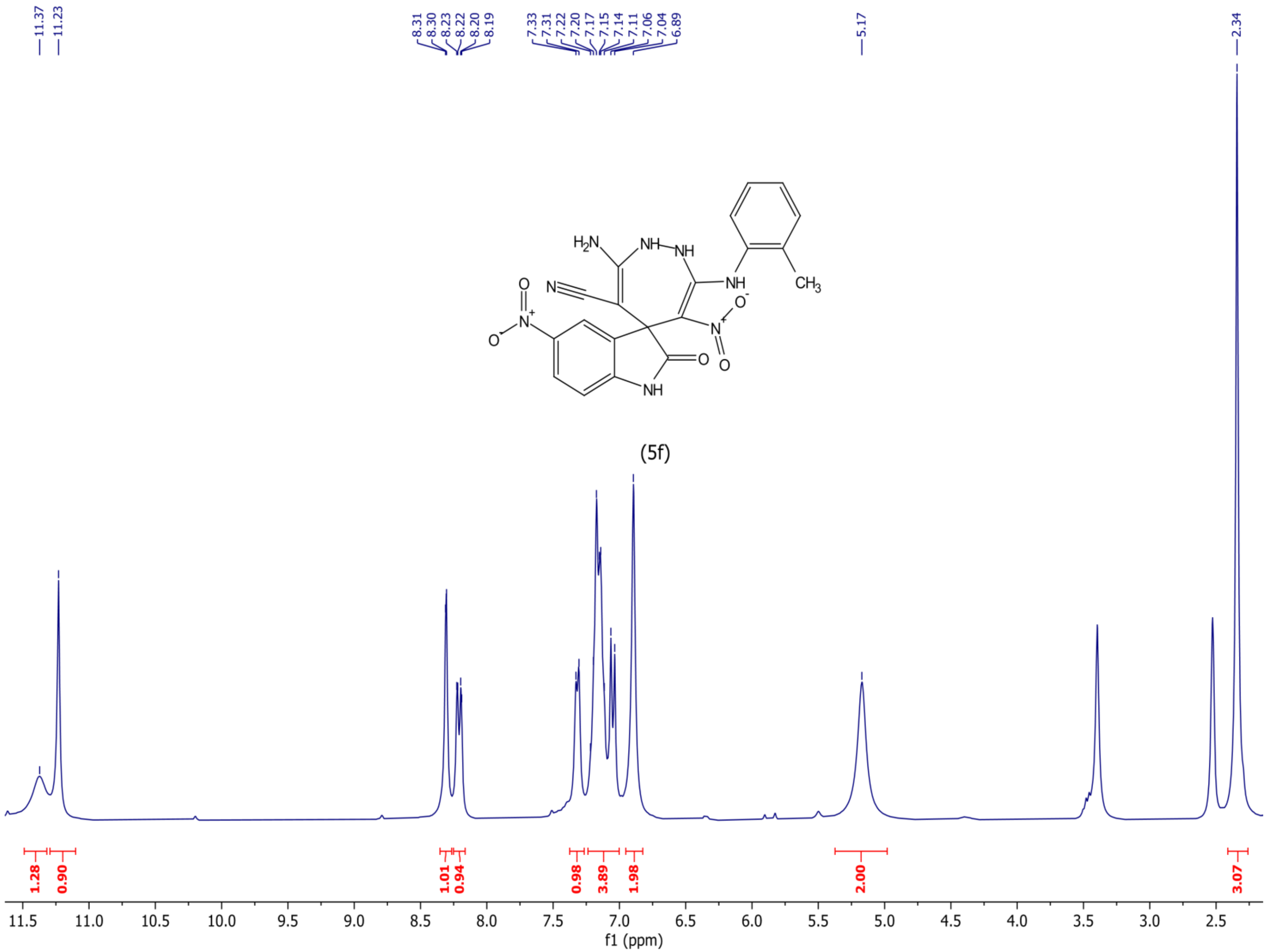
11.37
11.23
8.31
8.30
8.23
8.22
8.20
8.19
7.33
7.31
7.22
7.20
7.17
7.15
7.14
7.11
7.06
7.04
6.89
5.17
2.34
H2N
NH
NH
NH
CH3
N
O
N+
O-
O
N+
O-
O
NH
(5f)
1.28
0.90
1.01
0.94
0.98
3.89
1.98
2.00
3.07
11.5
11.0
10.5
10.0
9.5
9.0
8.5
8.0
7.5
7.0
6.5
6.0
5.5
5.0
4.5
4.0
3.5
3.0
2.5
f1 (ppm)

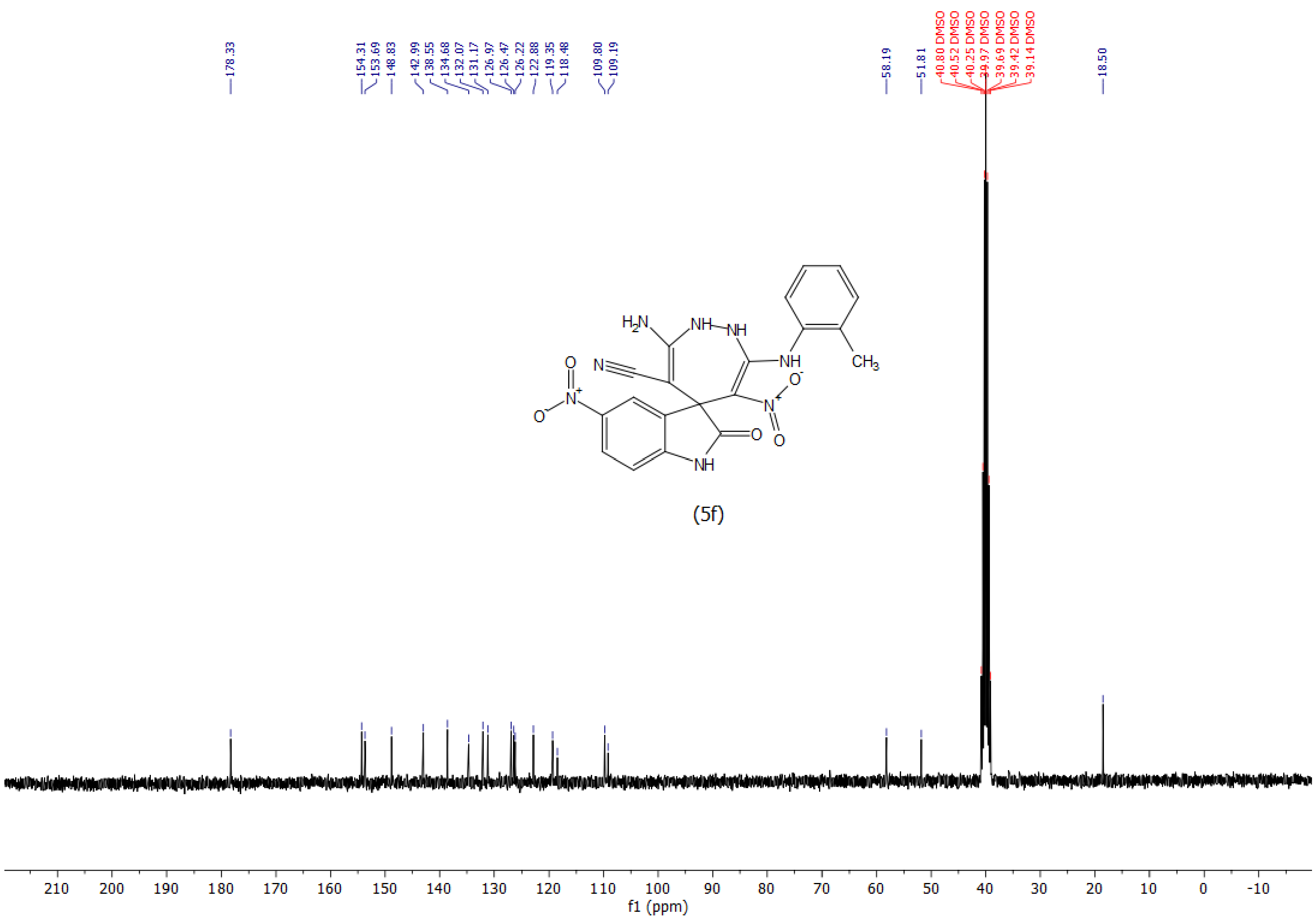
178.33
154.31
153.69
148.83
142.99
138.55
134.68
132.07
131.17
126.97
126.47
126.22
122.88
119.35
118.48
109.80
109.19
58.19
51.81
40.80 DMSO
40.52 DMSO
40.25 DMSO
39.97 DMSO
39.69 DMSO
39.42 DMSO
39.14 DMSO
18.50
H_2N
NH
NH
NH
CH_3
N
O
N^+
O^-
N^+
O^-
O
O
NH
(5f)
210 200 190 180 170 160 150 140 130 120 110 100 90 80 70 60 50 40 30 20 10 0 -10
f1 (ppm)

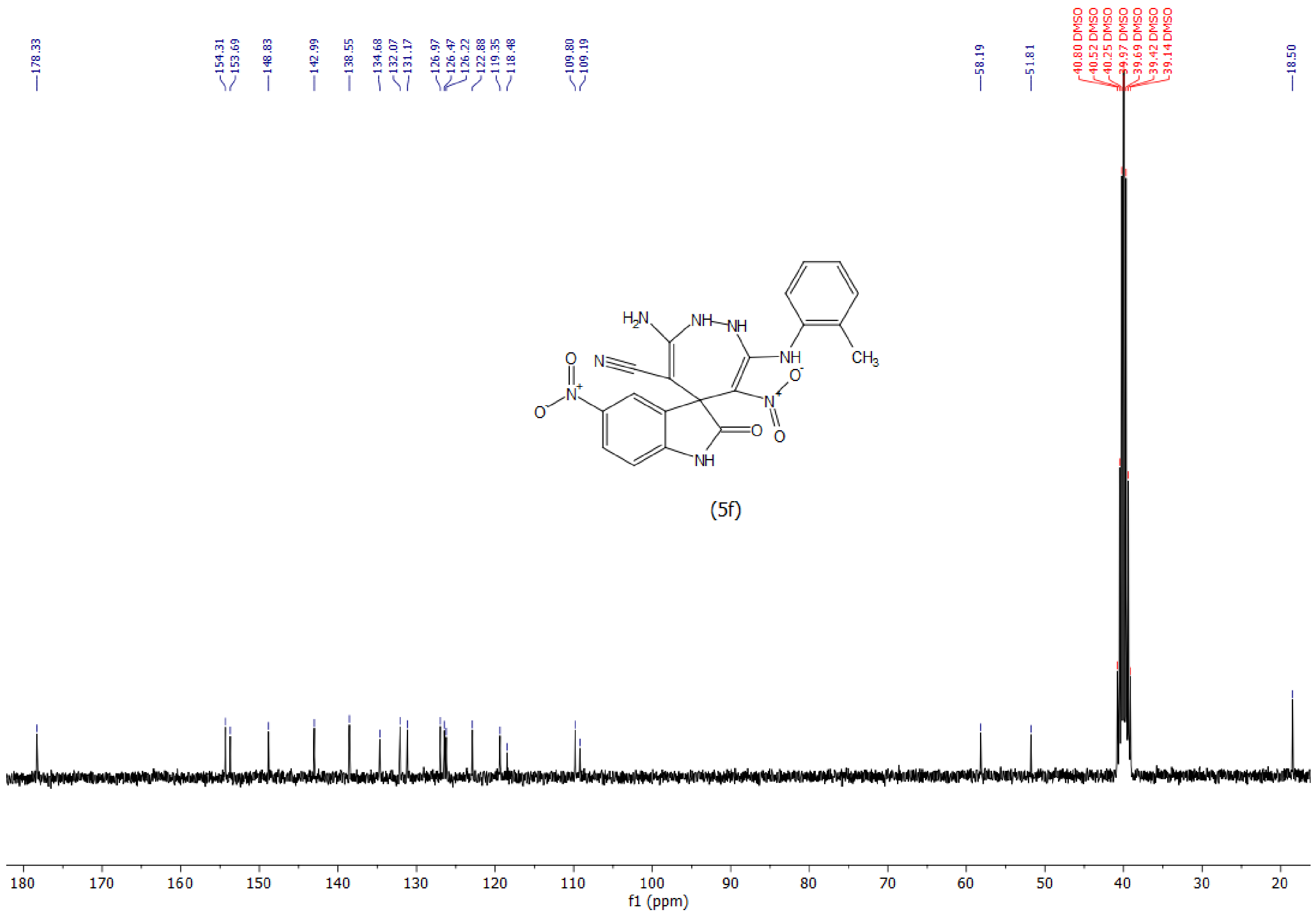
178.33
154.31
153.69
148.83
142.99
138.55
134.68
132.07
131.17
126.97
126.47
126.22
122.88
119.35
118.48
109.80
109.19
58.19
51.81
40.80 DMSO
40.52 DMSO
40.25 DMSO
39.97 DMSO
39.69 DMSO
39.42 DMSO
39.14 DMSO
18.50
H2N
NH
NH
NH
CH3
N
O
O
N+
O-
NH
O
(5f)
180
170
160
150
140
130
120
110
100
90
80
70
60
50
40
30
20
f1 (ppm)

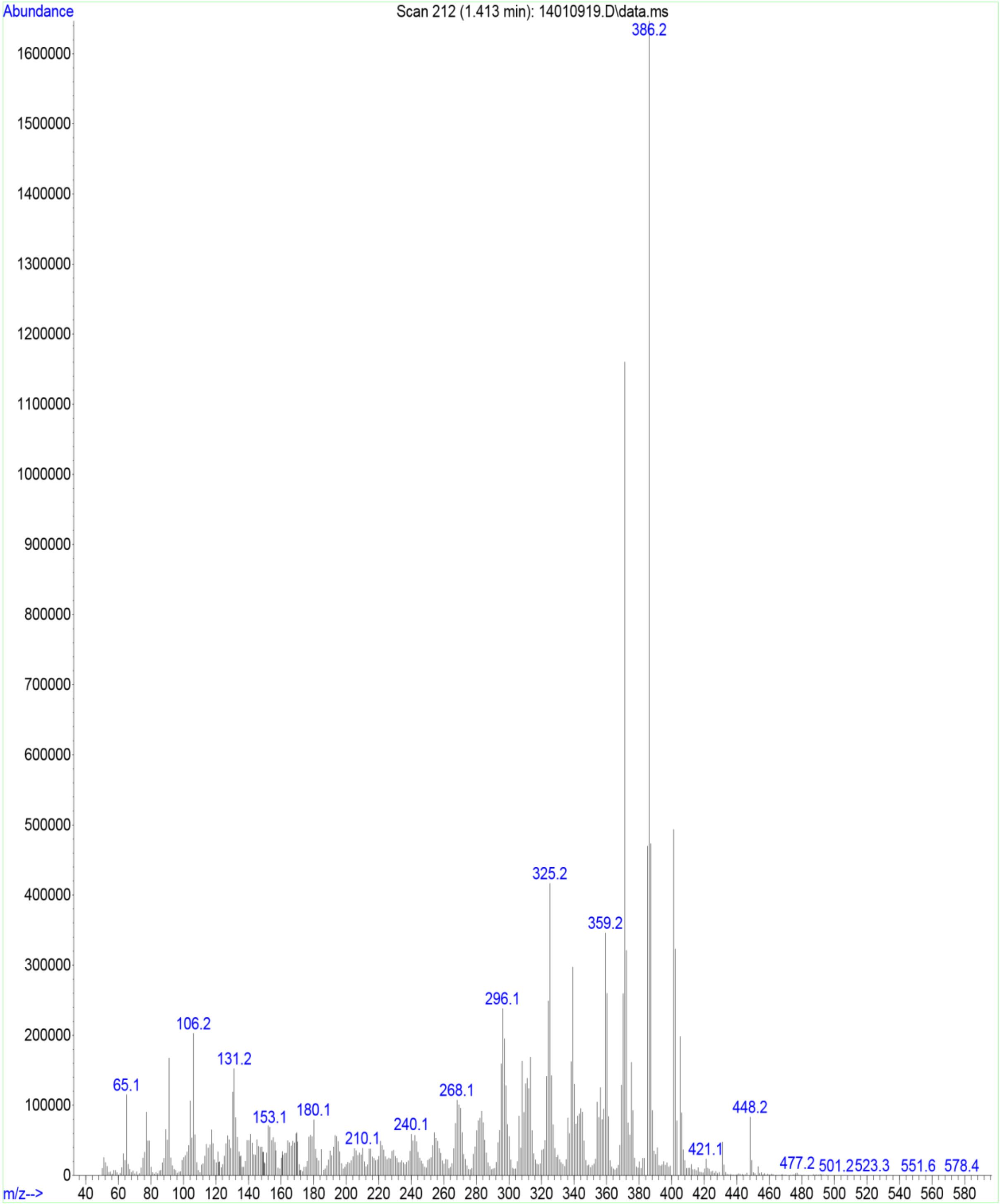

$C_{20}H_{16}N_8O_5$ **(5f)**

(448)

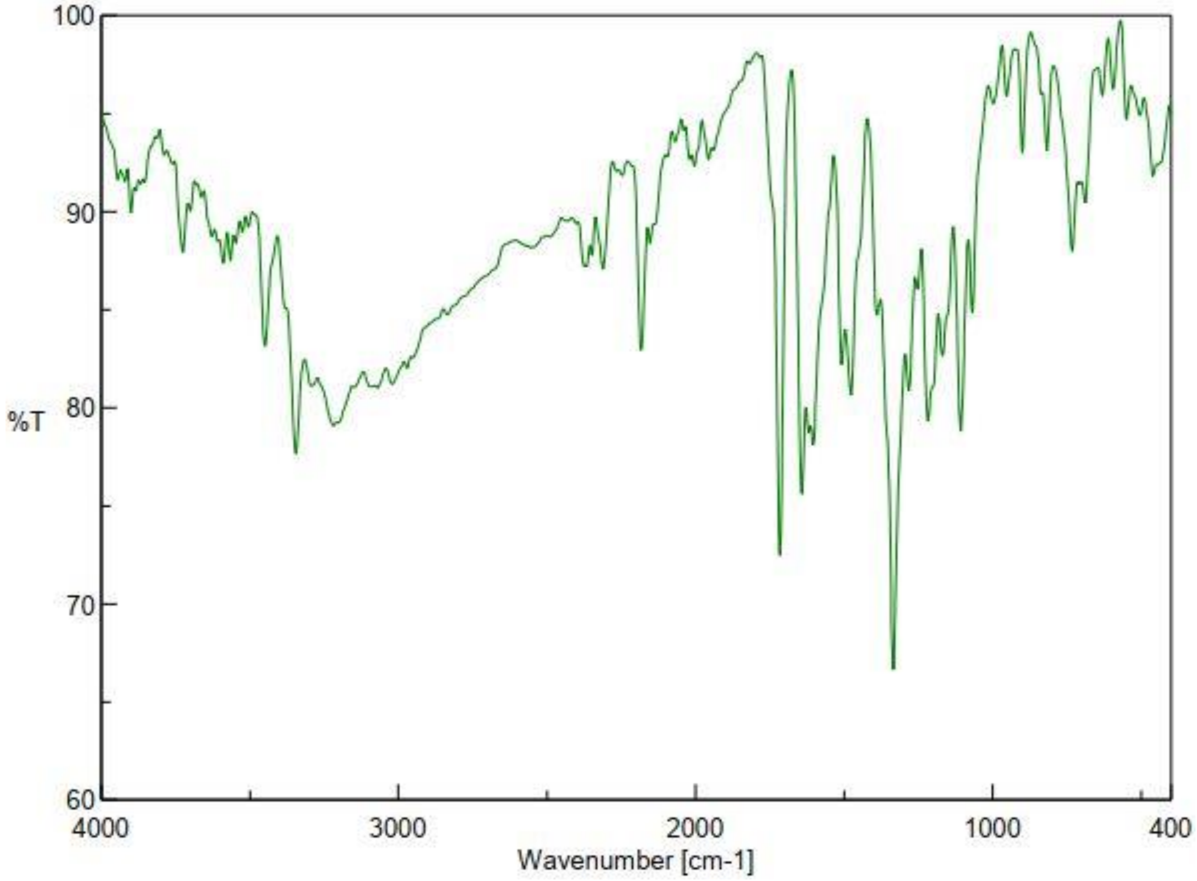

(5f)

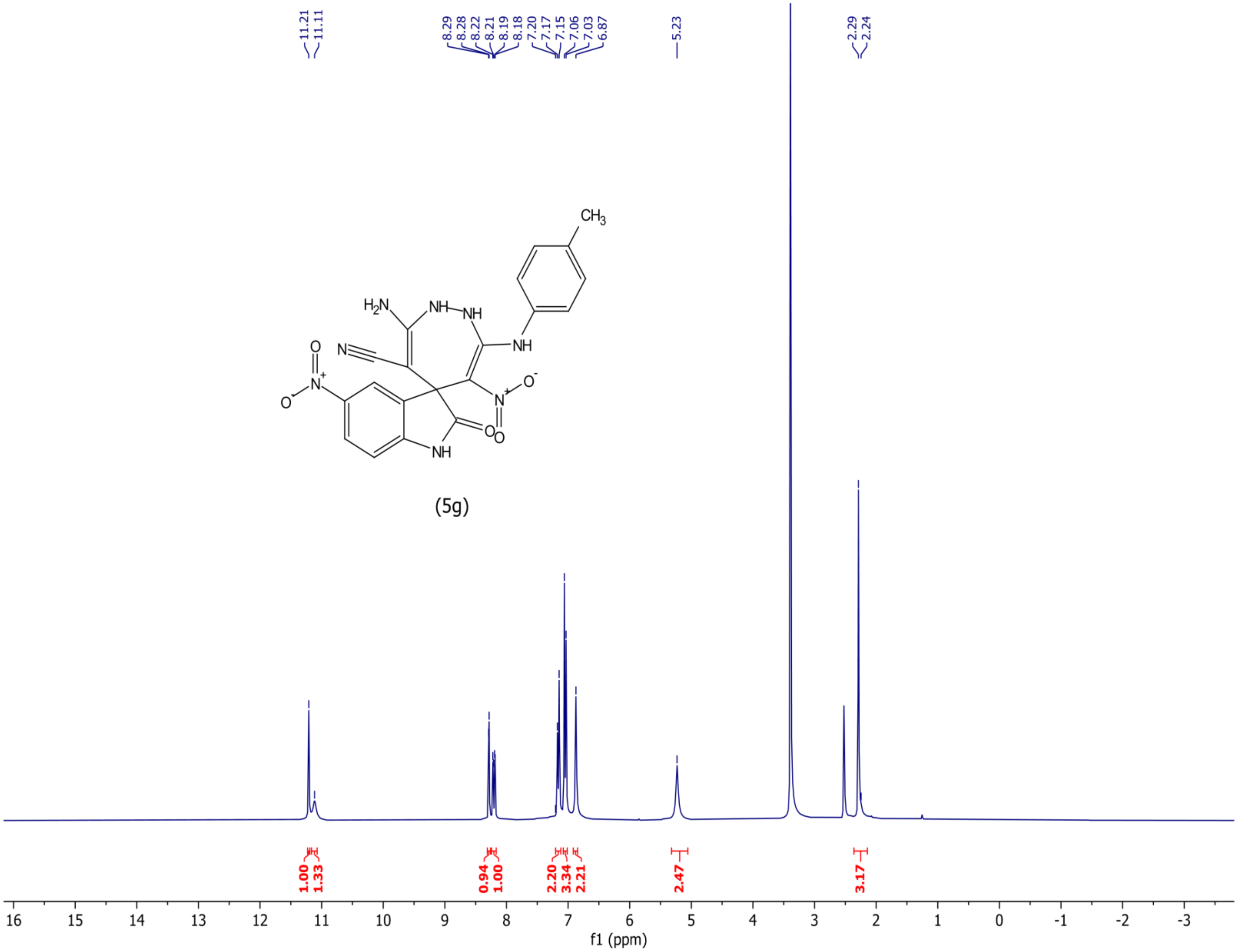
11.21
11.11
8.29
8.28
8.22
8.21
8.19
8.18
7.20
7.17
7.15
7.06
7.03
6.87
5.23
2.29
2.24
CH3
H2N
NH
NH
NH
N
O
N+
O-
N+
O-
O
O
NH
1.00
1.33
0.94
1.00
2.20
3.34
2.21
2.47
3.17
16
15
14
13
12
11
10
9
8
7
6
5
4
3
2
1
0
-1
-2
-3
f1 (ppm)

(5g)

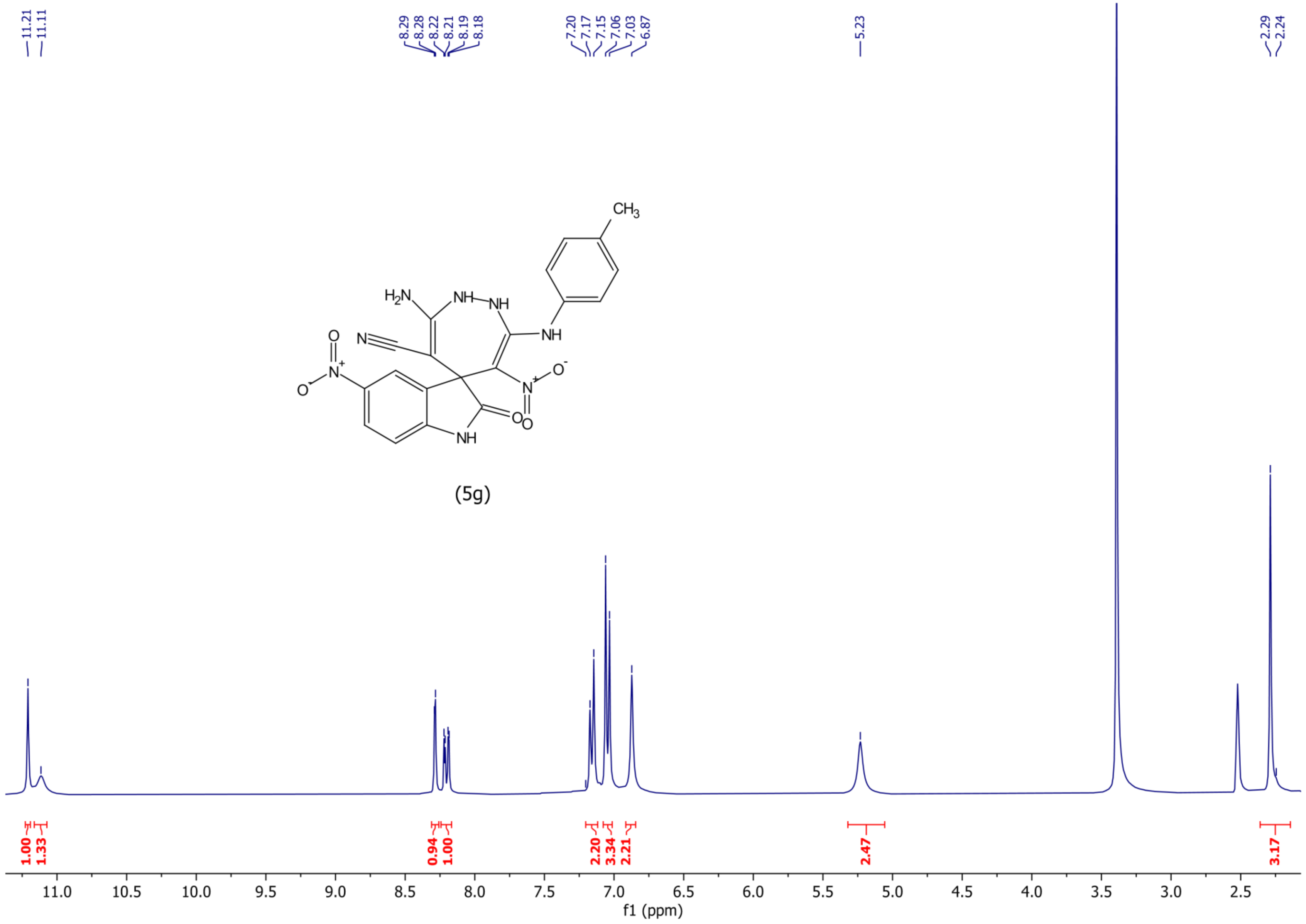

11.21
11.11
8.29
8.28
8.22
8.21
8.19
8.18
7.20
7.17
7.15
7.06
7.03
6.87
5.23
2.29
2.24
1.00
1.33
0.94
1.00
2.20
3.34
2.21
2.47
3.17
f1 (ppm)

(5g)

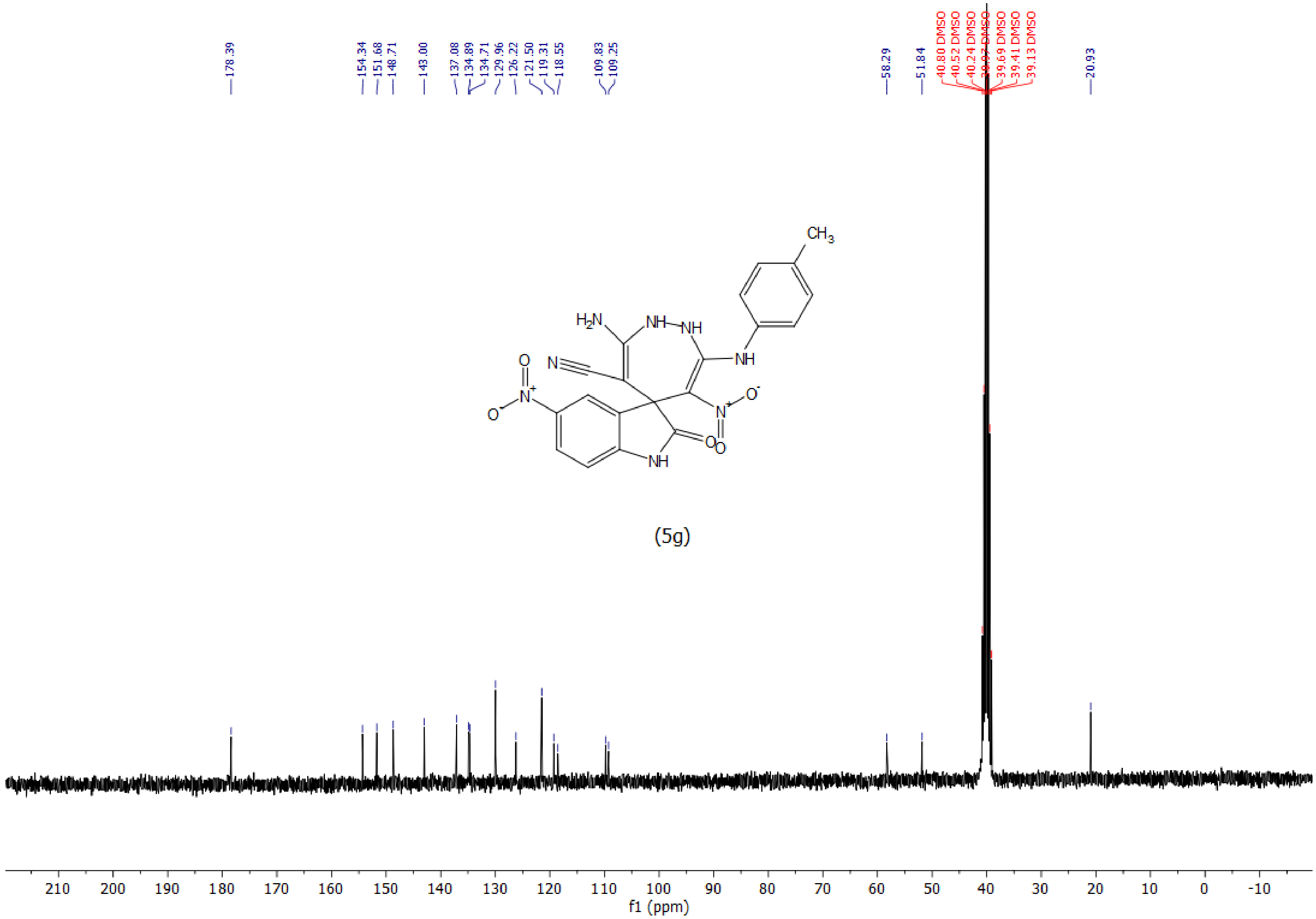

178.39
154.34
151.68
148.71
143.00
137.08
134.89
134.71
129.96
126.22
121.50
119.31
118.55
109.83
109.25
58.29
51.84
40.80 DMSO
40.52 DMSO
40.24 DMSO
39.69 DMSO
39.41 DMSO
39.13 DMSO
20.93
CH3
H2N
NH
NH
NH
N
O
N+
O-
N+
O-
O
NH
f1 (ppm)

(5g)

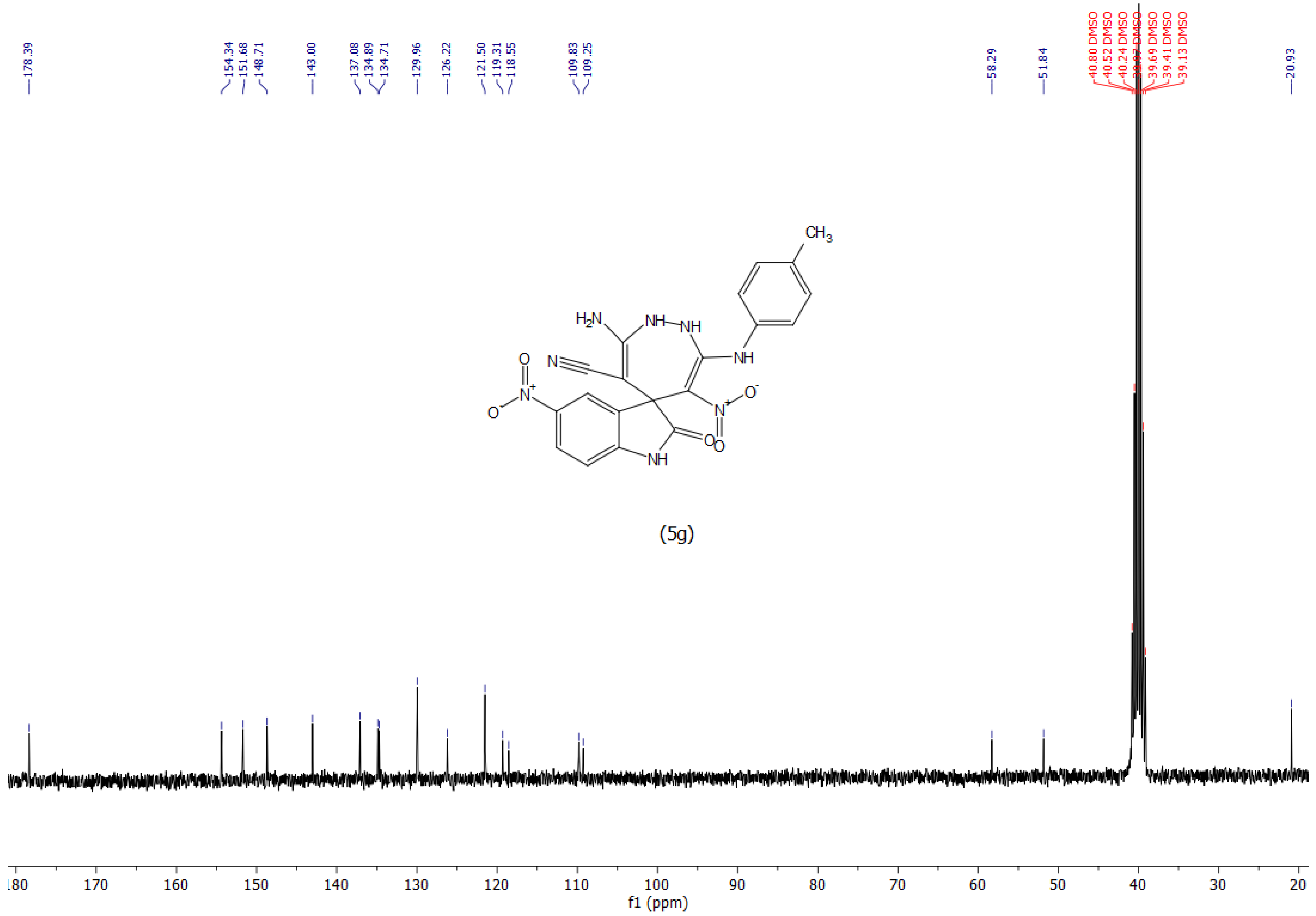

178.39
154.34
151.68
148.71
143.00
137.08
134.89
134.71
129.96
126.22
121.50
119.31
118.55
109.83
109.25
58.29
51.84
40.80 DMSO
40.52 DMSO
40.24 DMSO
39.97 DMSO
39.69 DMSO
39.41 DMSO
39.13 DMSO
20.93
CH3
H2N
NH
NH
NH
N
O
O
N+
O-
N+
O
O
NH
(5g)
180
170
160
150
140
130
120
110
100
90
80
70
60
50
40
30
20
f1 (ppm)

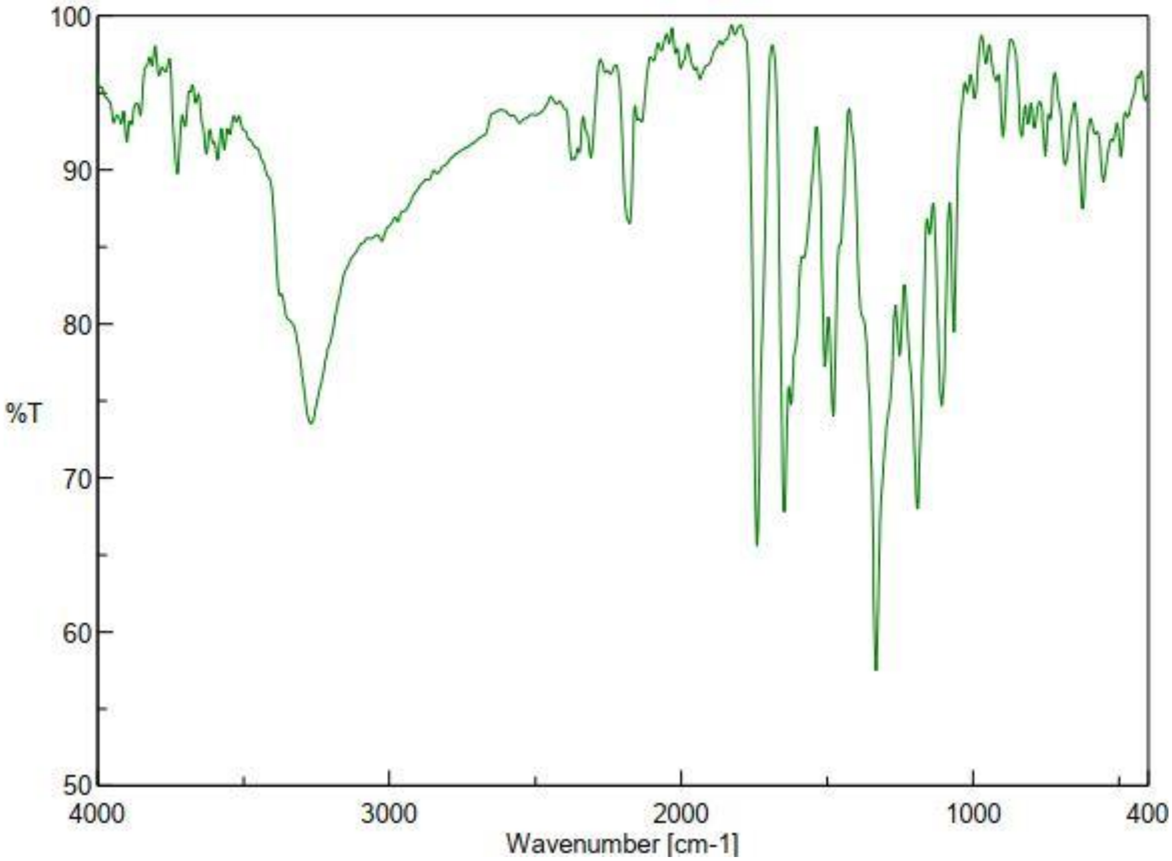

(5g)

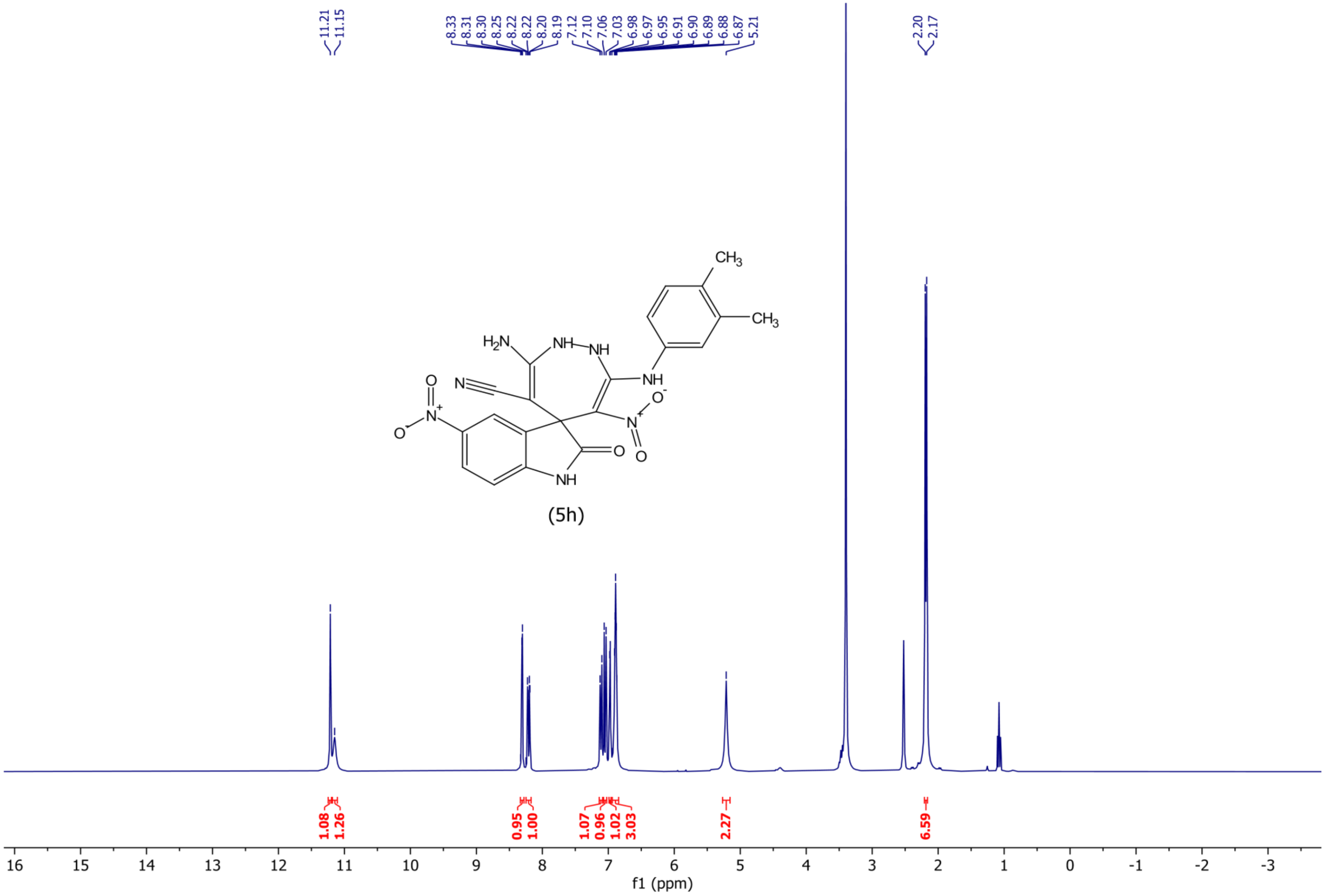
11.21
11.15
8.33
8.31
8.30
8.25
8.22
8.22
8.20
8.19
7.12
7.10
7.06
7.03
6.98
6.97
6.95
6.91
6.90
6.89
6.88
6.87
5.21
2.20
2.17
CH3
CH3
H2N
NH
NH
NH
N
O
N
O
O
N
O
O
NH
(5h)
1.08
1.26
0.95
1.00
1.07
0.96
1.02
3.03
2.27
6.59
16
15
14
13
12
11
10
9
8
7
6
5
4
3
2
1
0
-1
-2
-3
f1 (ppm)

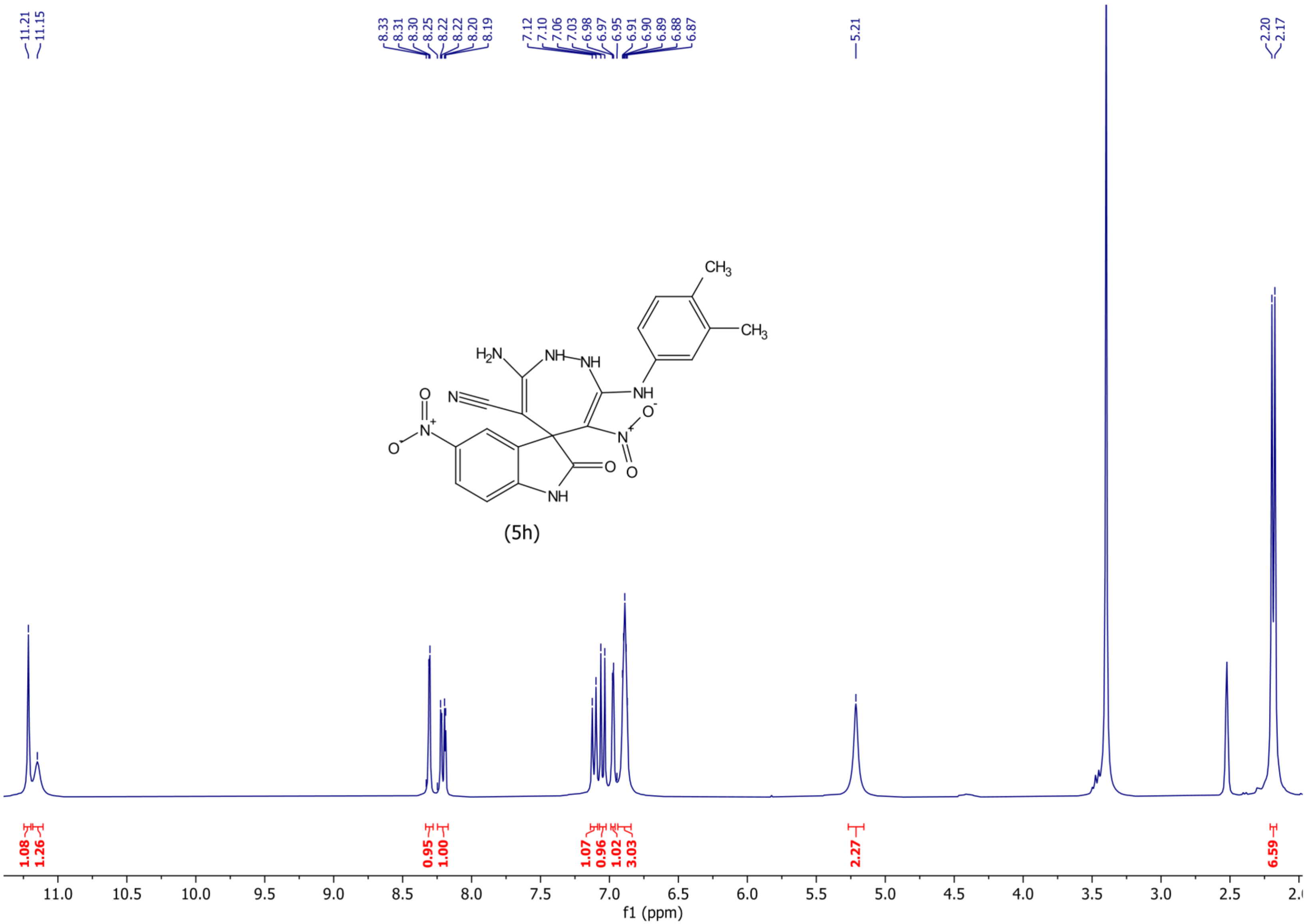
11.21
11.15
8.33
8.31
8.30
8.25
8.22
8.22
8.20
8.19
7.12
7.10
7.06
7.03
6.98
6.97
6.95
6.91
6.90
6.89
6.88
6.87
5.21
2.20
2.17
H2N
NH
NH
NH
CH3
CH3
N
O
N+
O-
O-
N+
O
O
NH
1.08
1.26
0.95
1.00
1.07
0.96
1.02
3.03
2.27
6.59
11.0
10.5
10.0
9.5
9.0
8.5
8.0
7.5
7.0
6.5
6.0
5.5
5.0
4.5
4.0
3.5
3.0
2.5
f1 (ppm)
(5h)

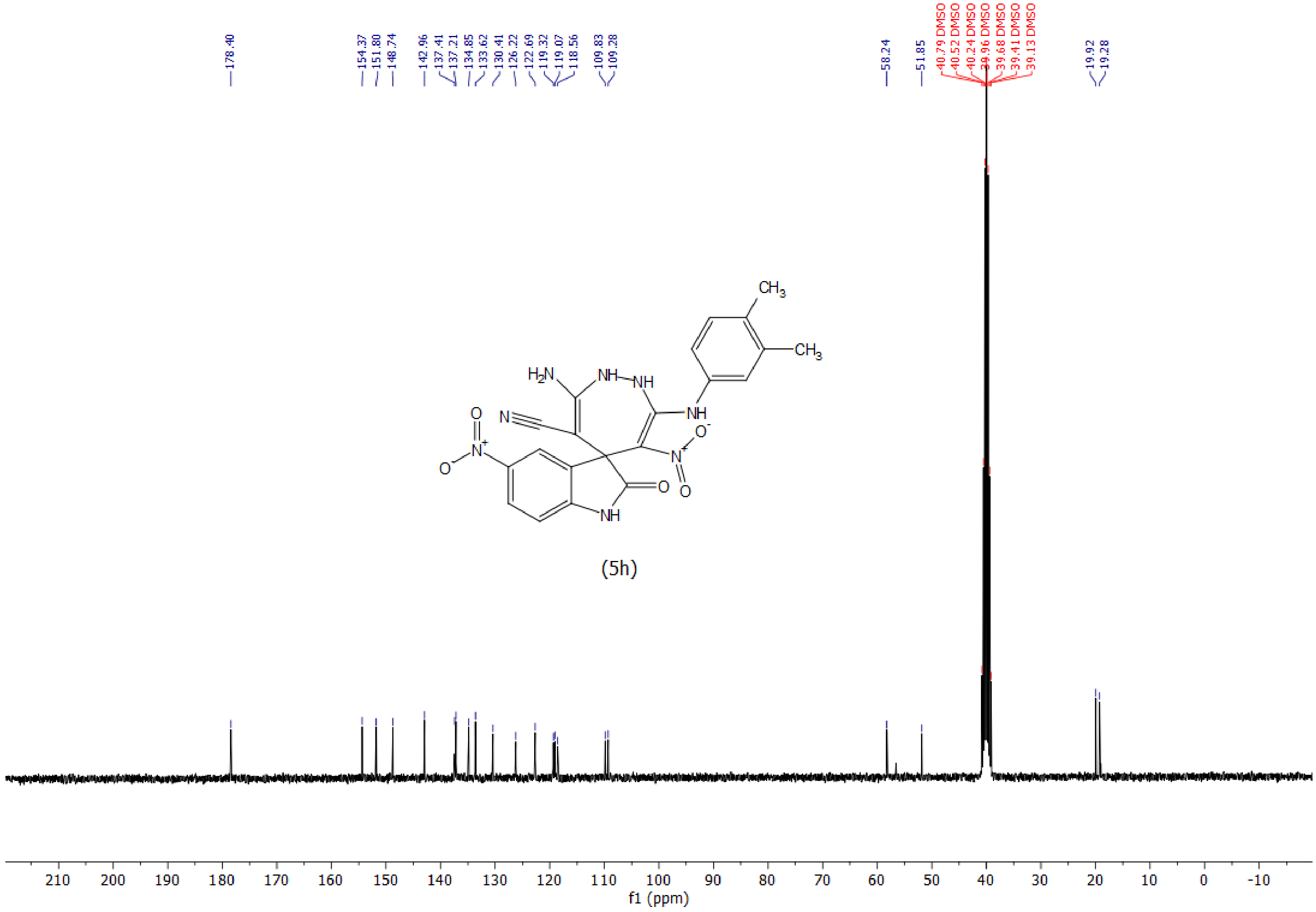
178.40
154.37
151.80
148.74
142.96
137.41
137.21
134.85
133.62
130.41
126.22
122.69
119.32
119.07
118.56
109.83
109.28
58.24
51.85
40.79 DMSO
40.52 DMSO
40.24 DMSO
39.96 DMSO
39.68 DMSO
39.41 DMSO
39.13 DMSO
19.92
19.28
H_2N
NH
NH
NH
N
O
N^+
O^-
CH_3
CH_3
(5h)
210 200 190 180 170 160 150 140 130 120 110 100 90 80 70 60 50 40 30 20 10 0 -10
f1 (ppm)

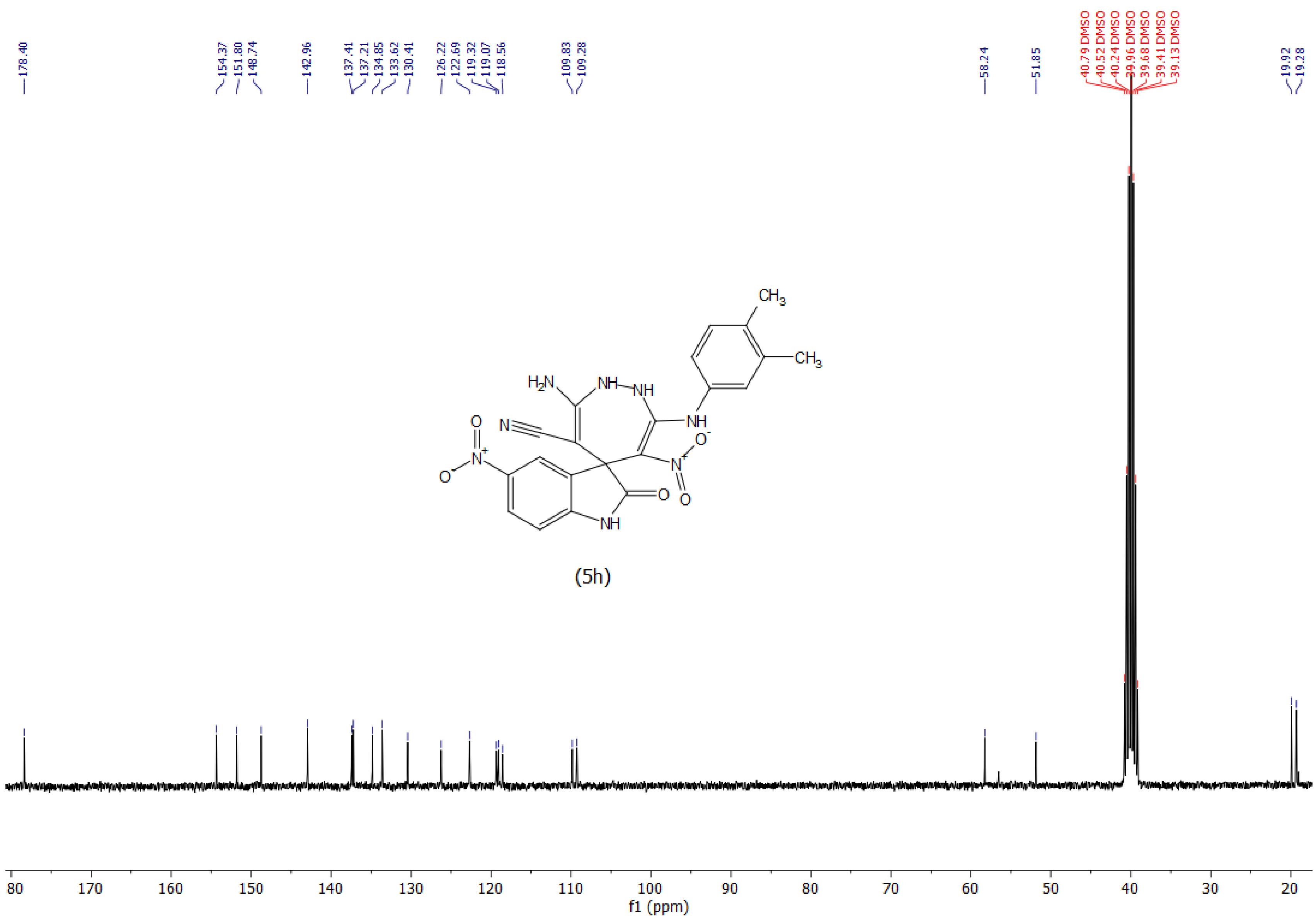

178.40
154.37
151.80
148.74
142.96
137.41
137.21
134.85
133.62
130.41
126.22
122.69
119.32
119.07
118.56
109.83
109.28
58.24
51.85
40.79 DMSO
40.52 DMSO
40.24 DMSO
39.96 DMSO
39.68 DMSO
39.41 DMSO
39.13 DMSO
19.92
19.28
H2N
NH
NH
NH
N
O
O
N+
O
O
N+
O
NH
CH3
CH3
80 170 160 150 140 130 120 110 100 90 80 70 60 50 40 30 20
f1 (ppm)

(5h)

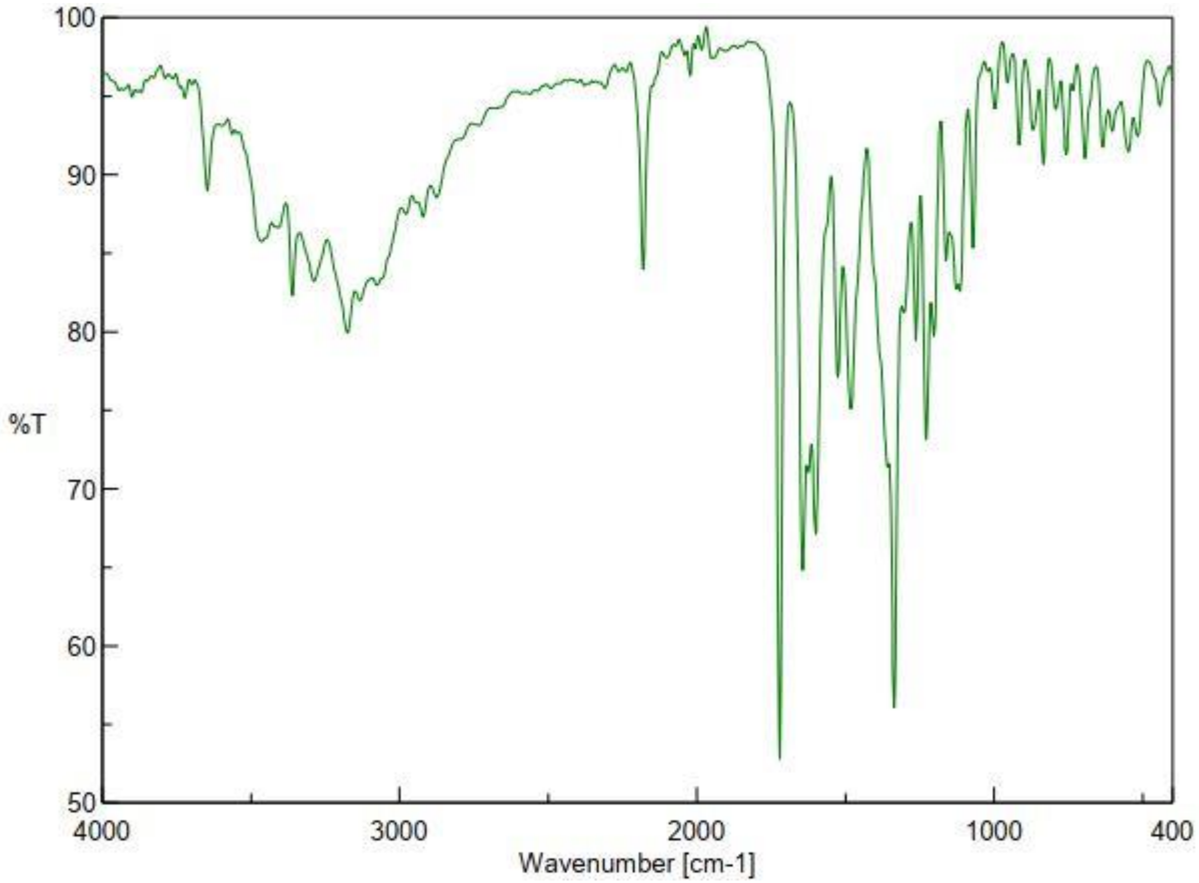

(5h)

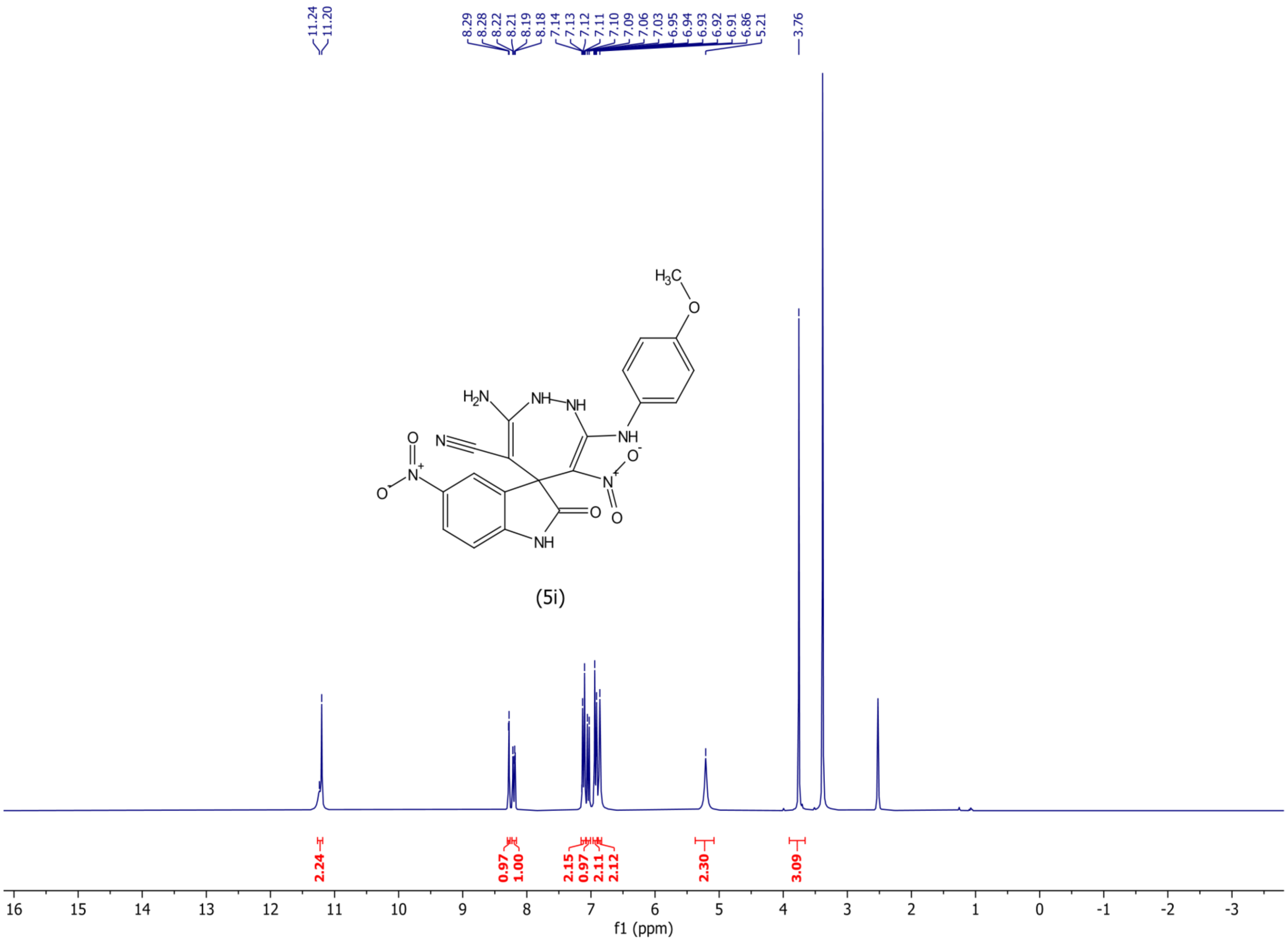
11.24
11.20
8.29
8.28
8.22
8.21
8.19
8.18
7.14
7.13
7.12
7.11
7.10
7.09
7.06
7.03
6.95
6.94
6.93
6.92
6.91
6.86
5.21
3.76
H_2N
NH
NH
NH
N
H_3C
O
N^+
O^-
O
NH
2.24
0.97
1.00
2.15
0.97
2.11
2.12
2.30
3.09
f1 (ppm)

(5i)

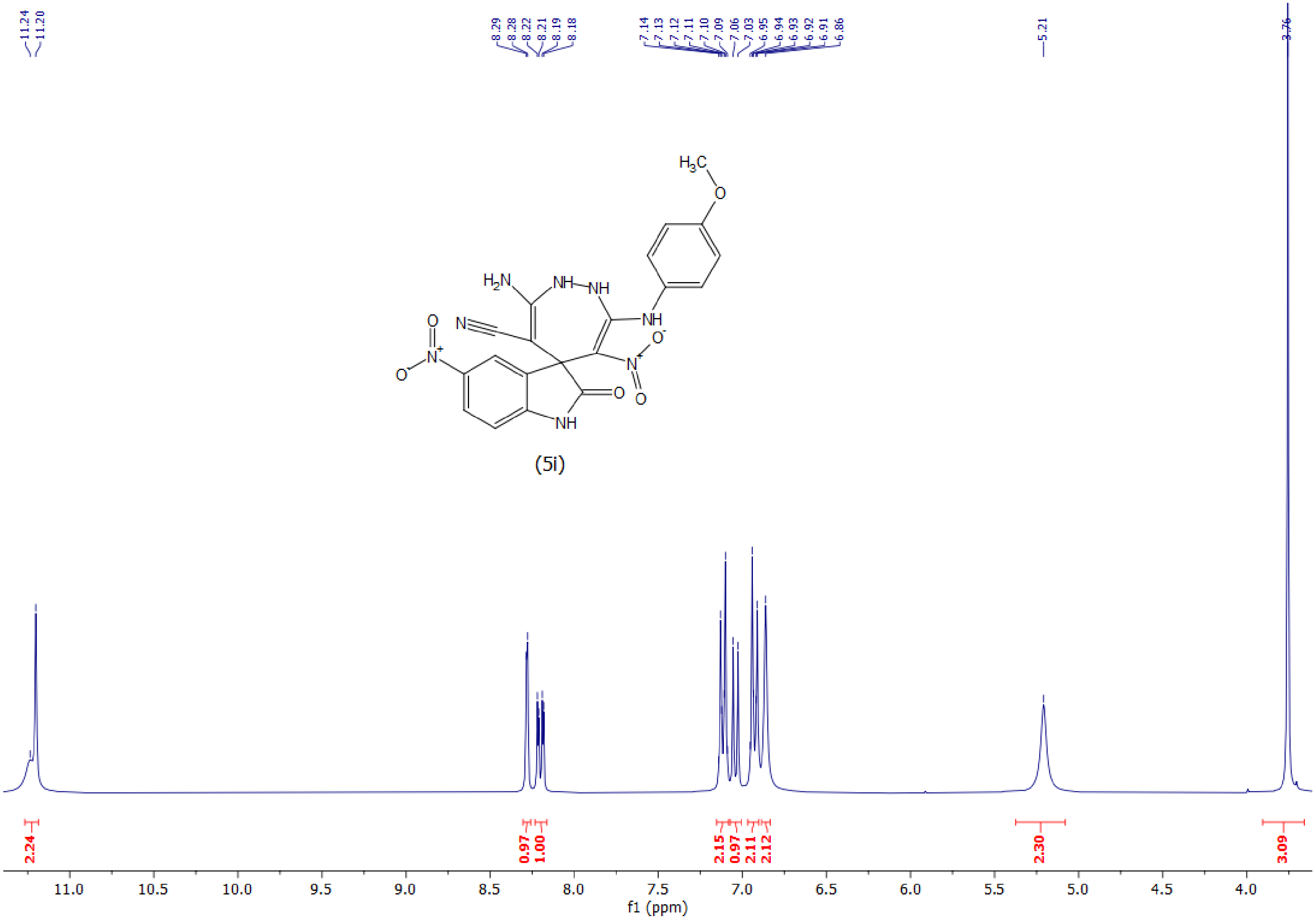
11.24
11.20
8.29
8.28
8.22
8.21
8.19
8.18
7.14
7.13
7.12
7.11
7.10
7.09
7.06
7.03
6.95
6.94
6.93
6.92
6.91
6.86
5.21
3.76
2.24
0.97
1.00
2.15
0.97
2.11
2.12
2.30
3.09
11.0
10.5
10.0
9.5
9.0
8.5
8.0
7.5
7.0
6.5
6.0
5.5
5.0
4.5
4.0
f1 (ppm)

(5i)

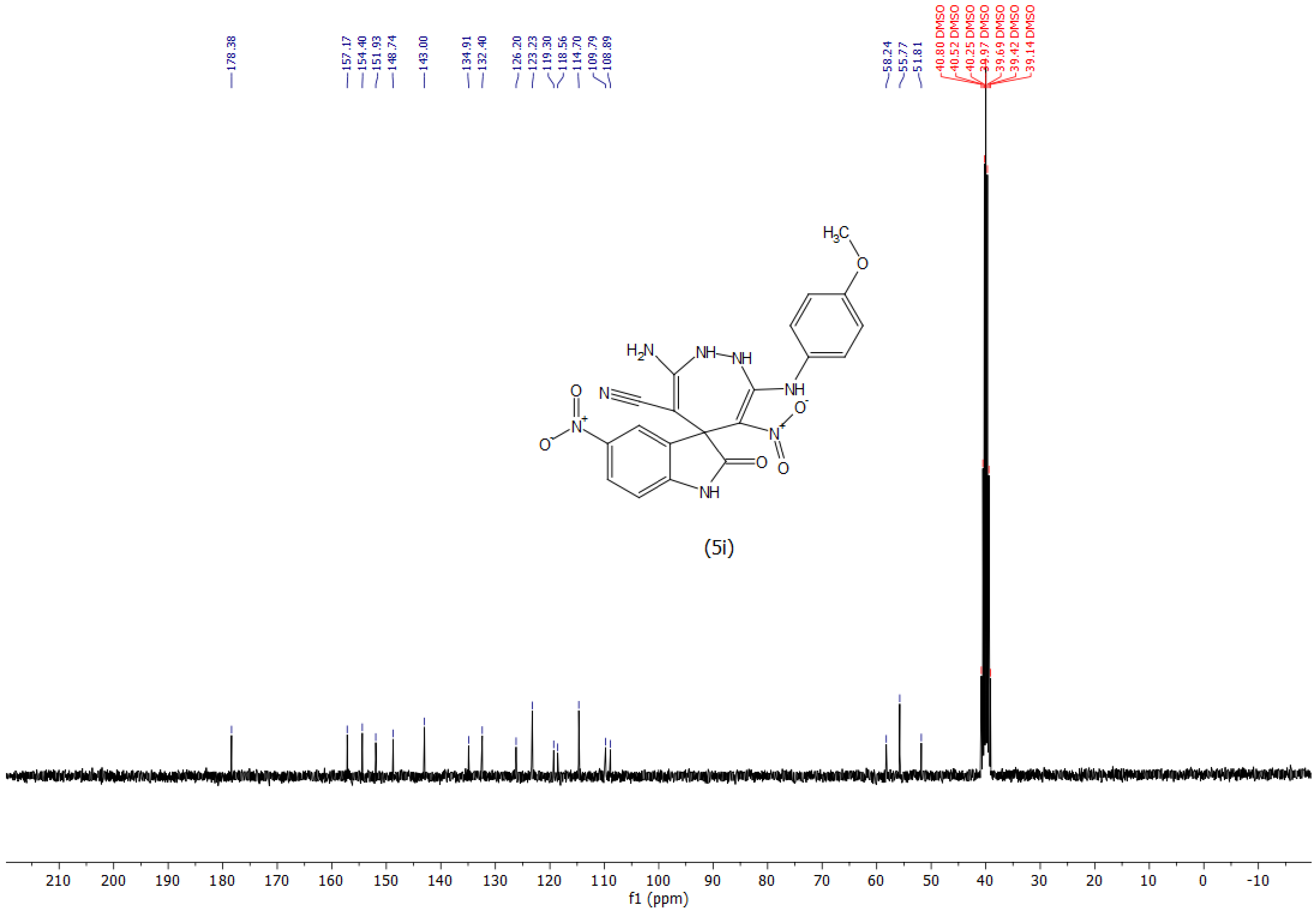
178.38
157.17
154.40
151.93
148.74
143.00
134.91
132.40
126.20
123.23
119.30
118.56
114.70
109.79
108.89
58.24
55.77
51.81
40.80 DMSO
40.52 DMSO
40.25 DMSO
39.97 DMSO
39.69 DMSO
39.42 DMSO
39.14 DMSO
H3C
O
H2N
NH
NH
NH
N
N
O
O
O
O
NH
210
200
190
180
170
160
150
140
130
120
110
100
90
80
70
60
50
40
30
20
10
0
-10
f1 (ppm)

(5i)

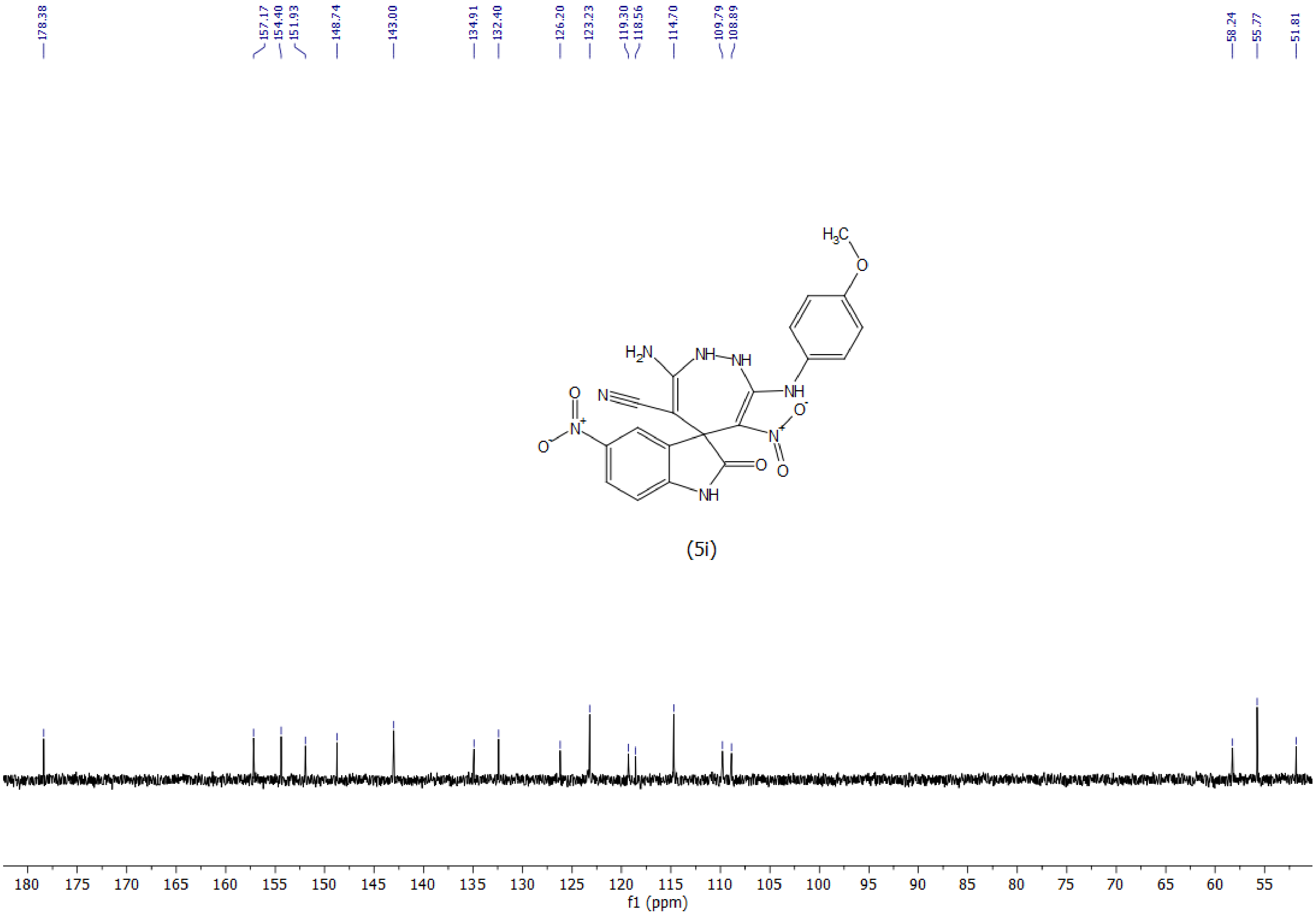

178.38
157.17
154.40
151.93
148.74
143.00
134.91
132.40
126.20
123.23
119.30
118.56
114.70
109.79
108.89
58.24
55.77
51.81
H3C
O
H2N
NH
NH
NH
N
O
N+
O-
O-
N+
O
O
NH
180 175 170 165 160 155 150 145 140 135 130 125 120 115 110 105 100 95 90 85 80 75 70 65 60 55
f1 (ppm)

(5i)

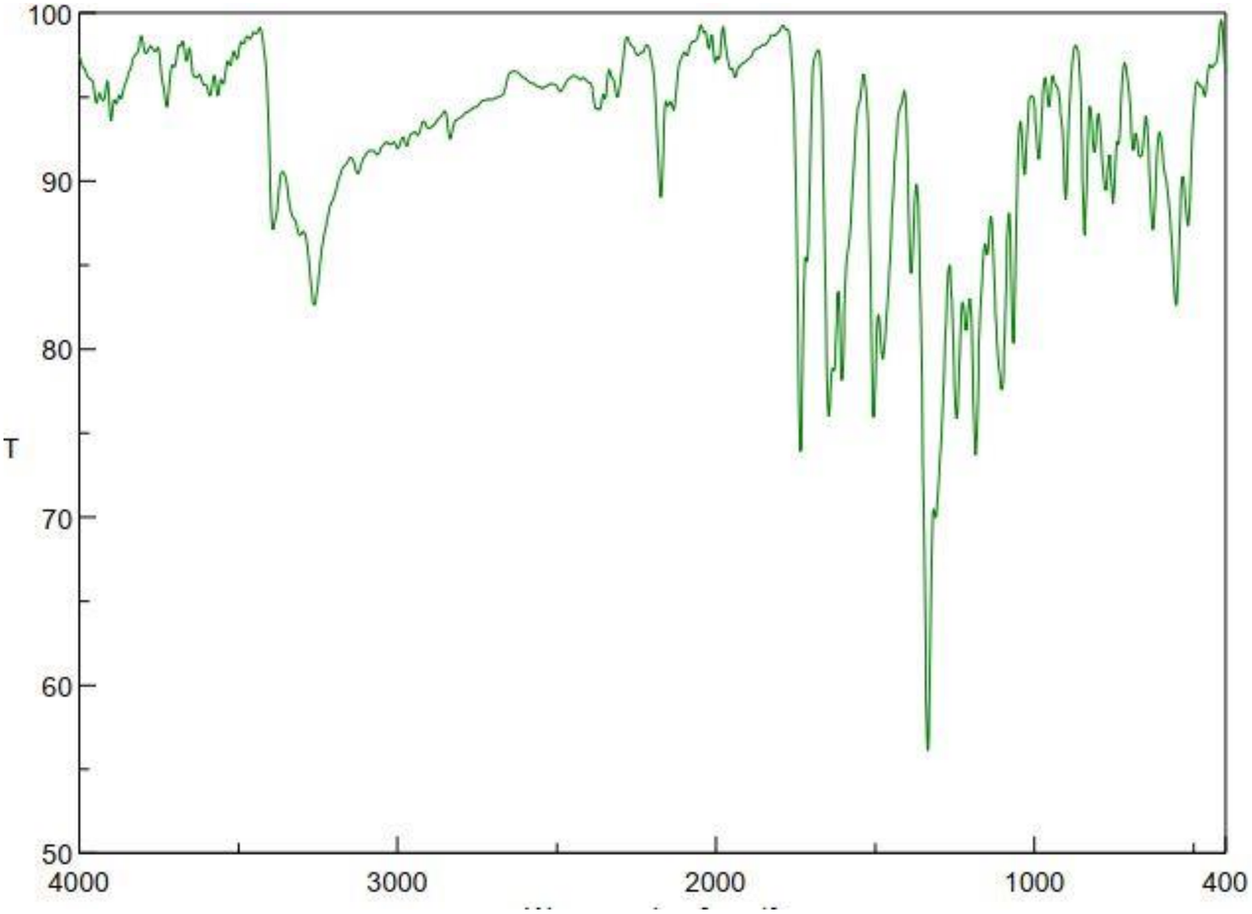

(5i)

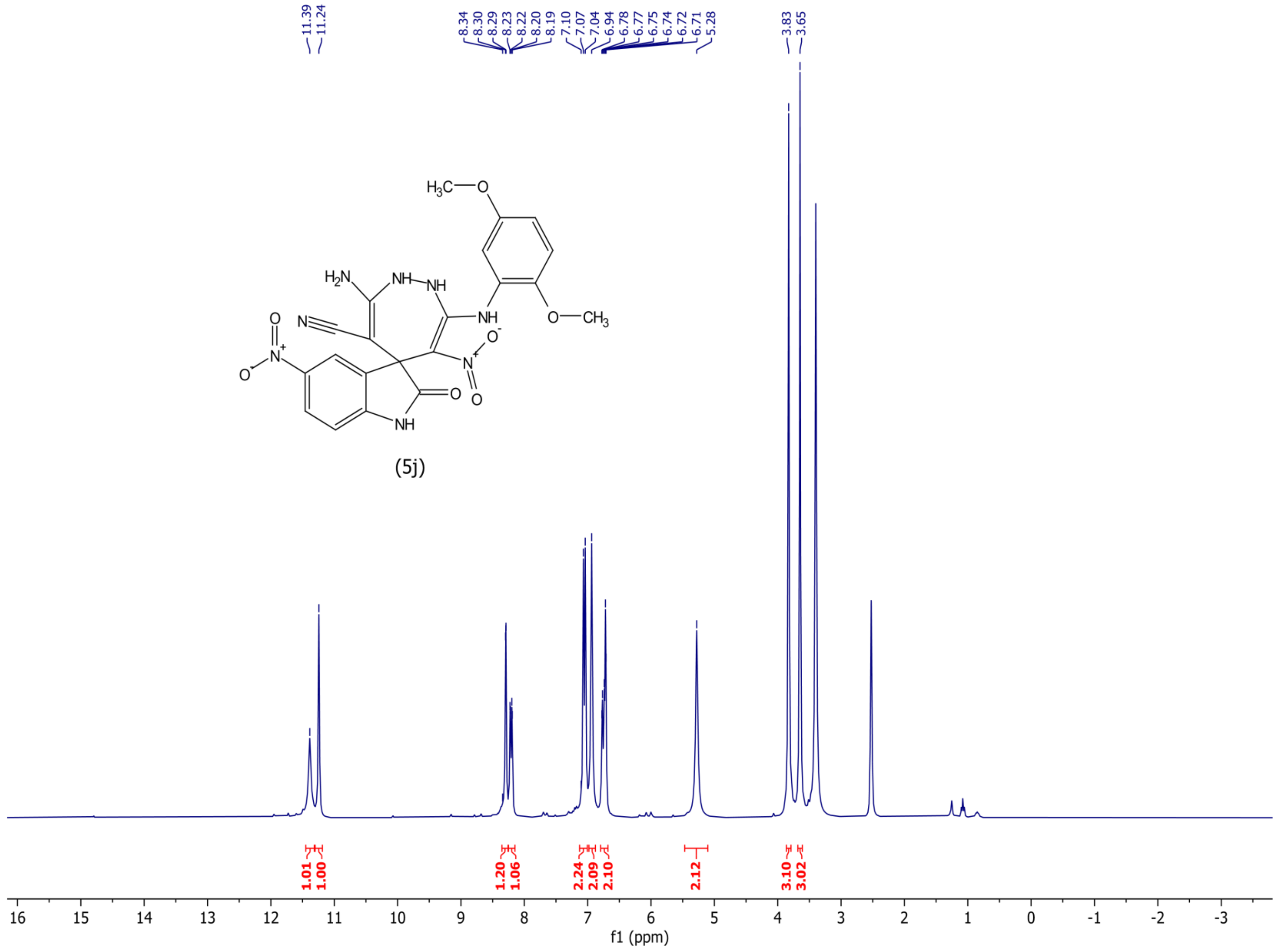

11.39
11.24
8.34
8.30
8.29
8.23
8.22
8.20
8.19
7.10
7.07
7.04
6.94
6.78
6.77
6.75
6.74
6.72
6.71
5.28
3.83
3.65
H3C—O
H2N
NH
NH
NH
O—CH3
N
O
N+
O-
O
NH
1.01
1.00
1.20
1.06
2.24
2.09
2.10
2.12
3.10
3.02
16
15
14
13
12
11
10
9
8
7
6
5
4
3
2
1
0
-1
-2
-3
f1 (ppm)

(5j)

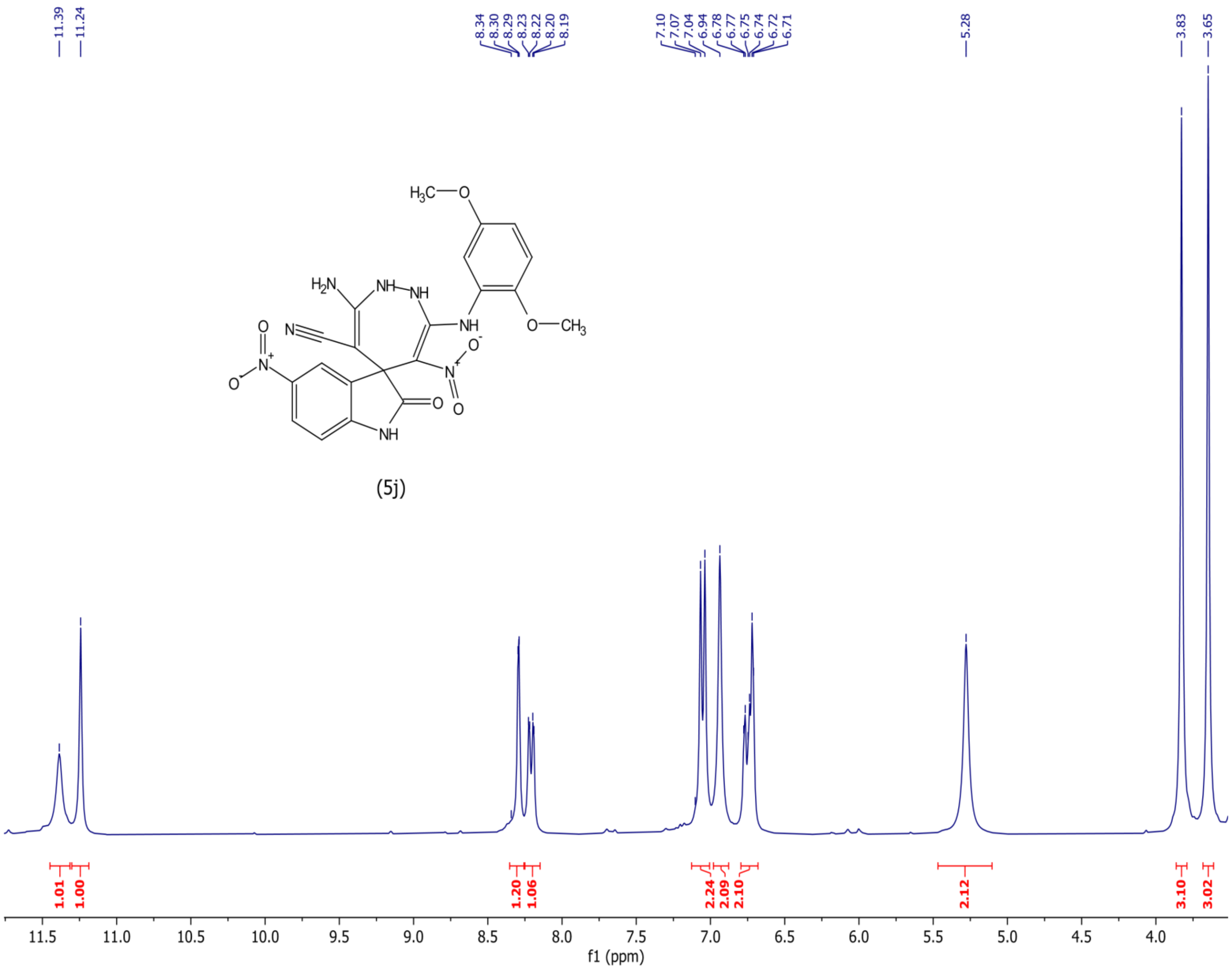

11.39
11.24
8.34
8.30
8.29
8.23
8.22
8.20
8.19
7.10
7.07
7.04
6.94
6.78
6.77
6.75
6.74
6.72
6.71
5.28
3.83
3.65
(5j)
1.01
1.00
1.20
1.06
2.24
2.09
2.10
2.12
3.10
3.02
11.5
11.0
10.5
10.0
9.5
9.0
8.5
8.0
7.5
7.0
6.5
6.0
5.5
5.0
4.5
4.0
f1 (ppm)

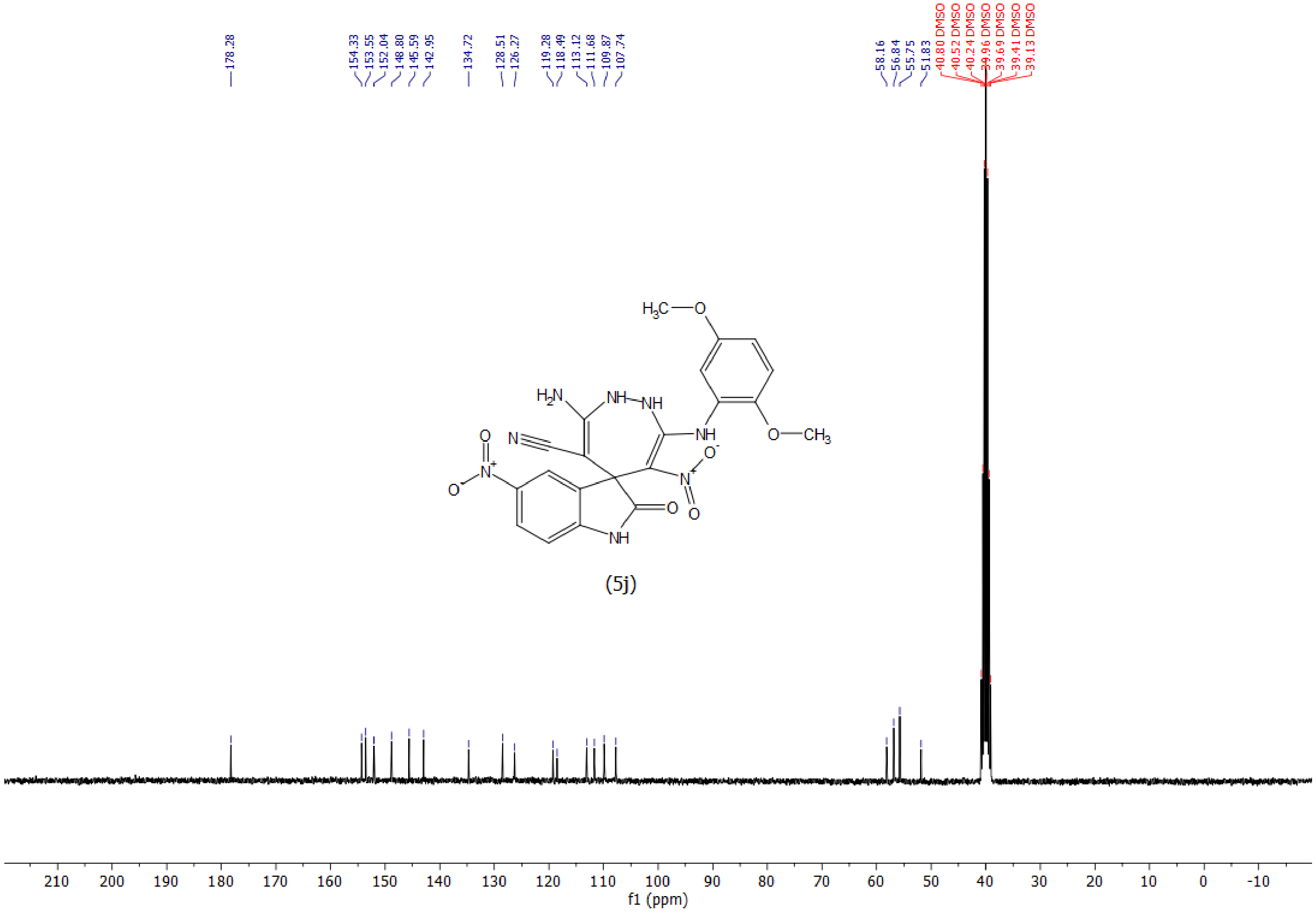

178.28
154.33
153.55
152.04
148.80
145.59
142.95
134.72
128.51
126.27
119.28
118.49
113.12
111.68
109.87
107.74
58.16
56.84
55.75
51.83
40.80 DMSO
40.52 DMSO
40.24 DMSO
39.96 DMSO
39.69 DMSO
39.41 DMSO
39.13 DMSO
H_2N
NH
NH
NH
H_3C
O
O
CH_3
N
O^-
N^+
O
O
NH
(5j)
210 200 190 180 170 160 150 140 130 120 110 100 90 80 70 60 50 40 30 20 10 0 -10
f1 (ppm)

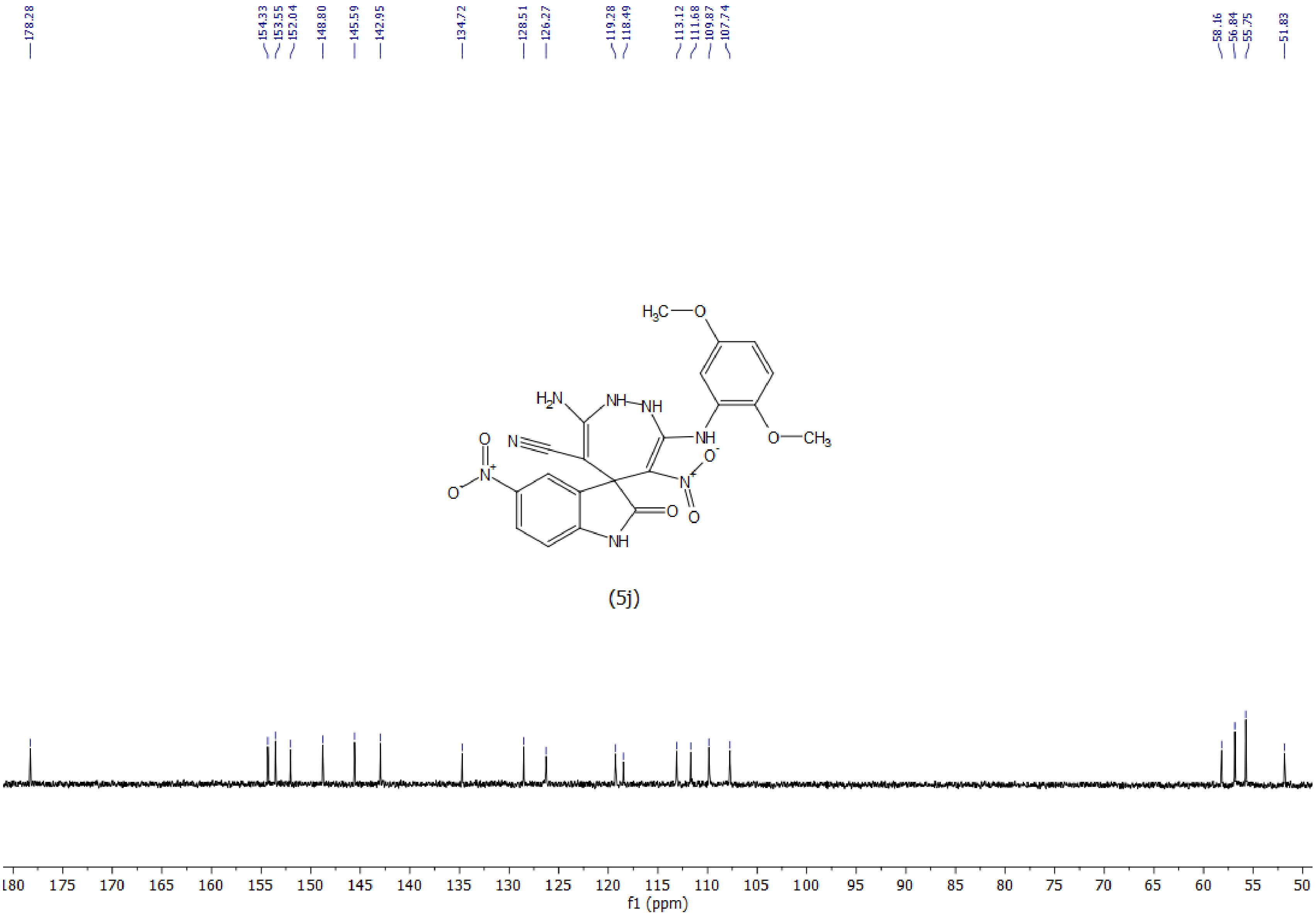

178.28
154.33
153.55
152.04
148.80
145.59
142.95
134.72
128.51
126.27
119.28
118.49
113.12
111.68
109.87
107.74
58.16
56.84
55.75
51.83
H3C—O
H2N
NH—NH
NH
O—CH3
N
O
N+
O-
O-
N+
O
O
NH
180 175 170 165 160 155 150 145 140 135 130 125 120 115 110 105 100 95 90 85 80 75 70 65 60 55 50
f1 (ppm)

(5j)

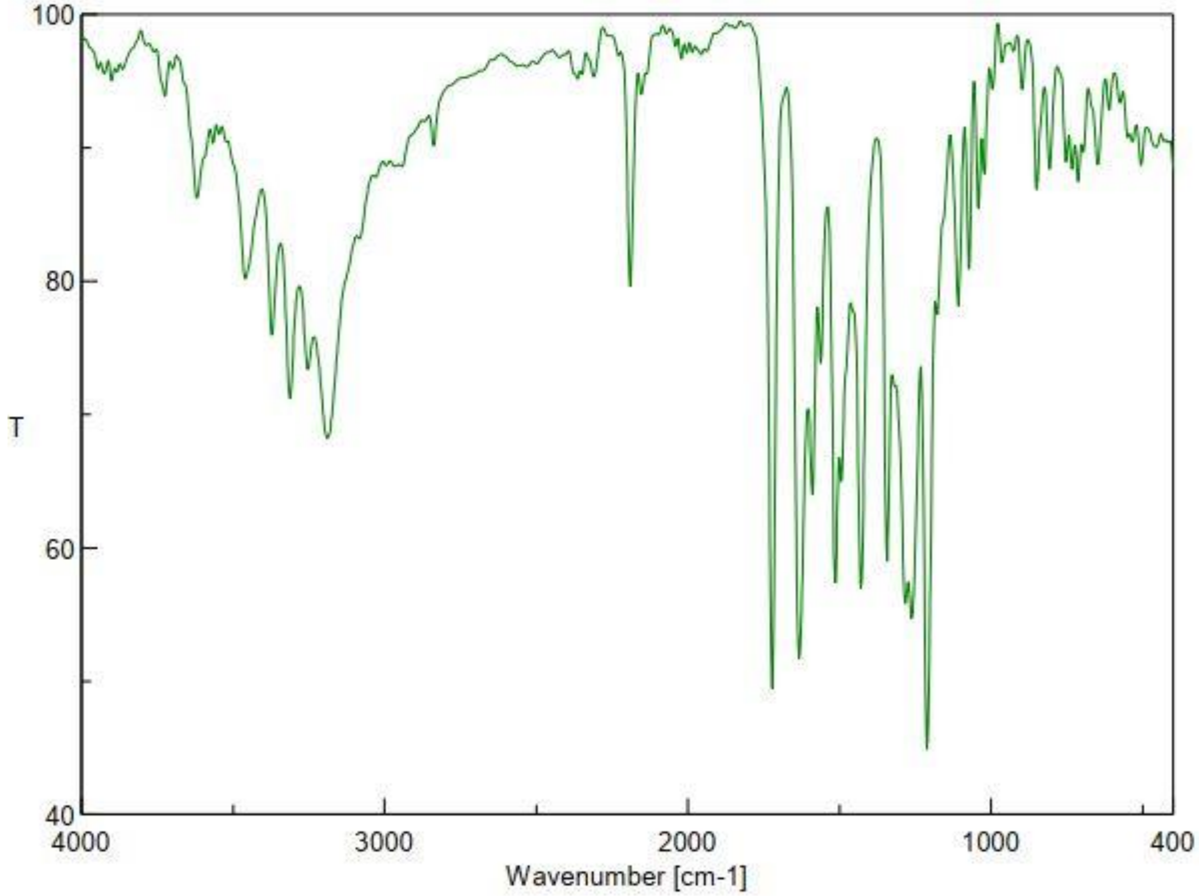

(5j)

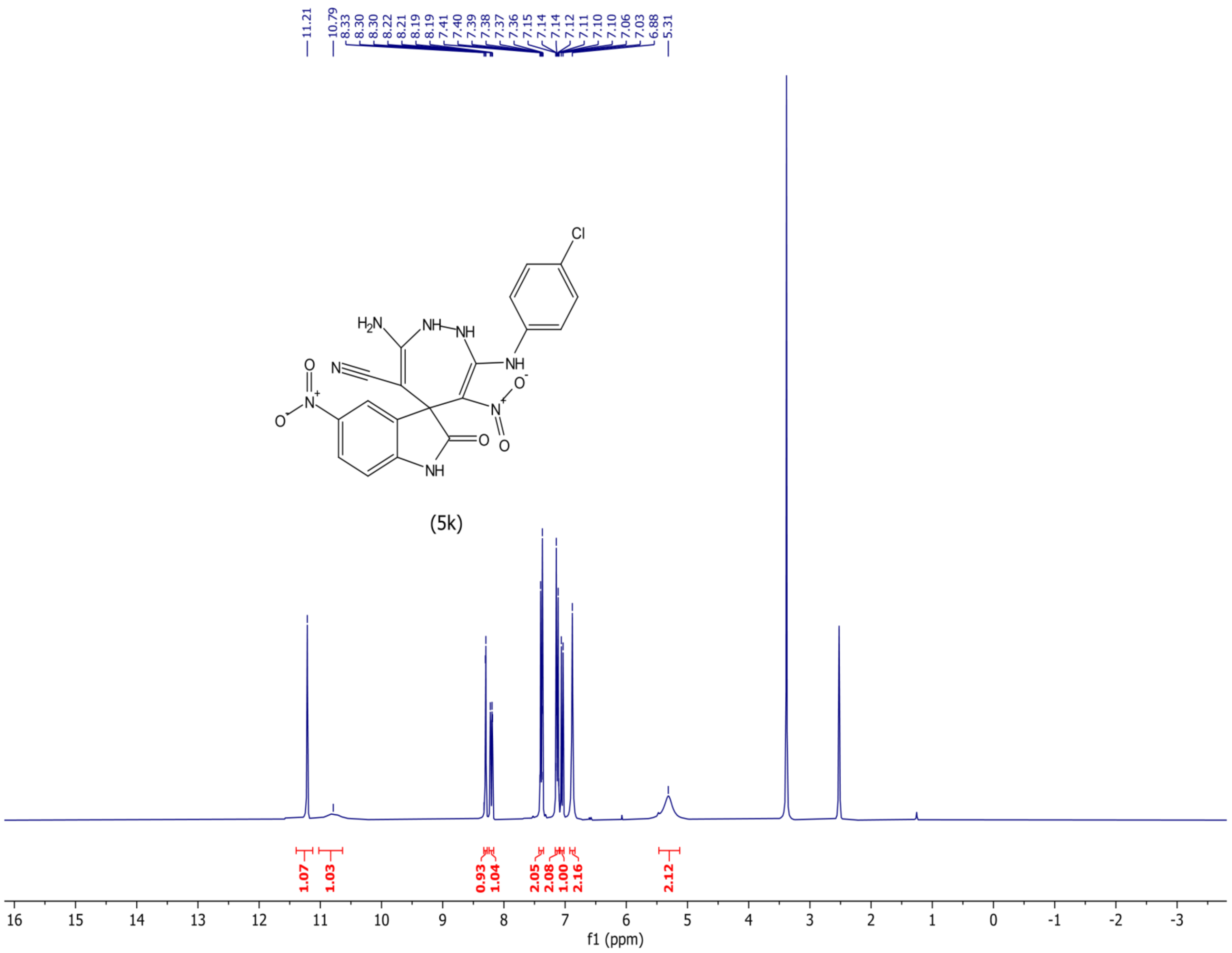

11.21
10.79
8.33
8.30
8.30
8.22
8.21
8.19
8.19
7.41
7.40
7.39
7.38
7.37
7.36
7.15
7.14
7.14
7.12
7.11
7.10
7.10
7.06
7.03
6.88
5.31
1.07
1.03
0.93
1.04
2.05
2.08
1.00
2.16
2.12
16 15 14 13 12 11 10 9 8 7 6 5 4 3 2 1 0 -1 -2 -3
f1 (ppm)

(5k)

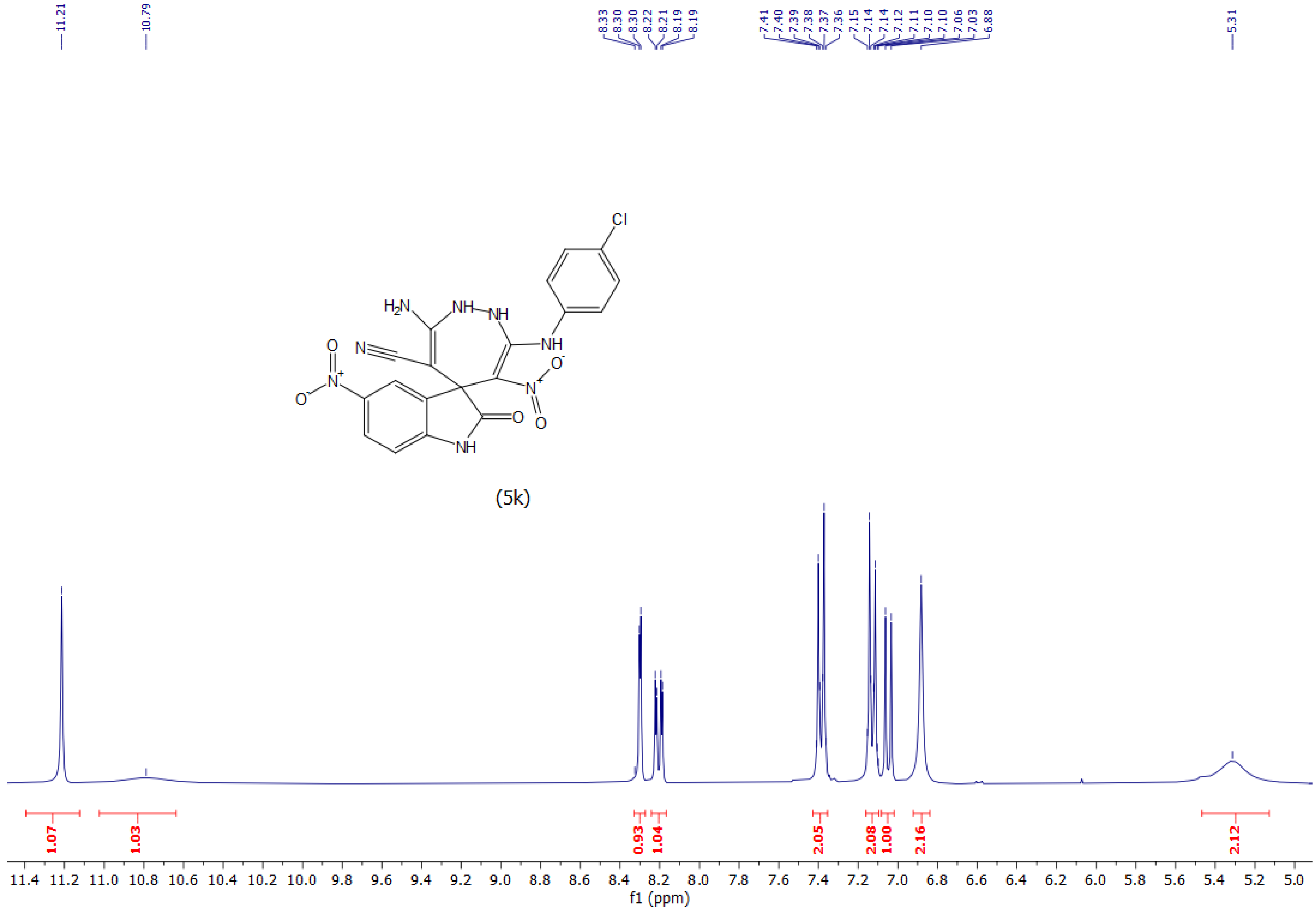
11.21
10.79
8.33
8.30
8.30
8.22
8.21
8.19
8.19
7.41
7.40
7.39
7.38
7.37
7.36
7.15
7.14
7.14
7.12
7.11
7.10
7.10
7.06
7.03
6.88
5.31
1.07
1.03
0.93
1.04
2.05
2.08
1.00
2.16
2.12
f1 (ppm)
Cl
H_2N
NH
NH
NH
N
O
N^+
O^-
O
NH

(5k)

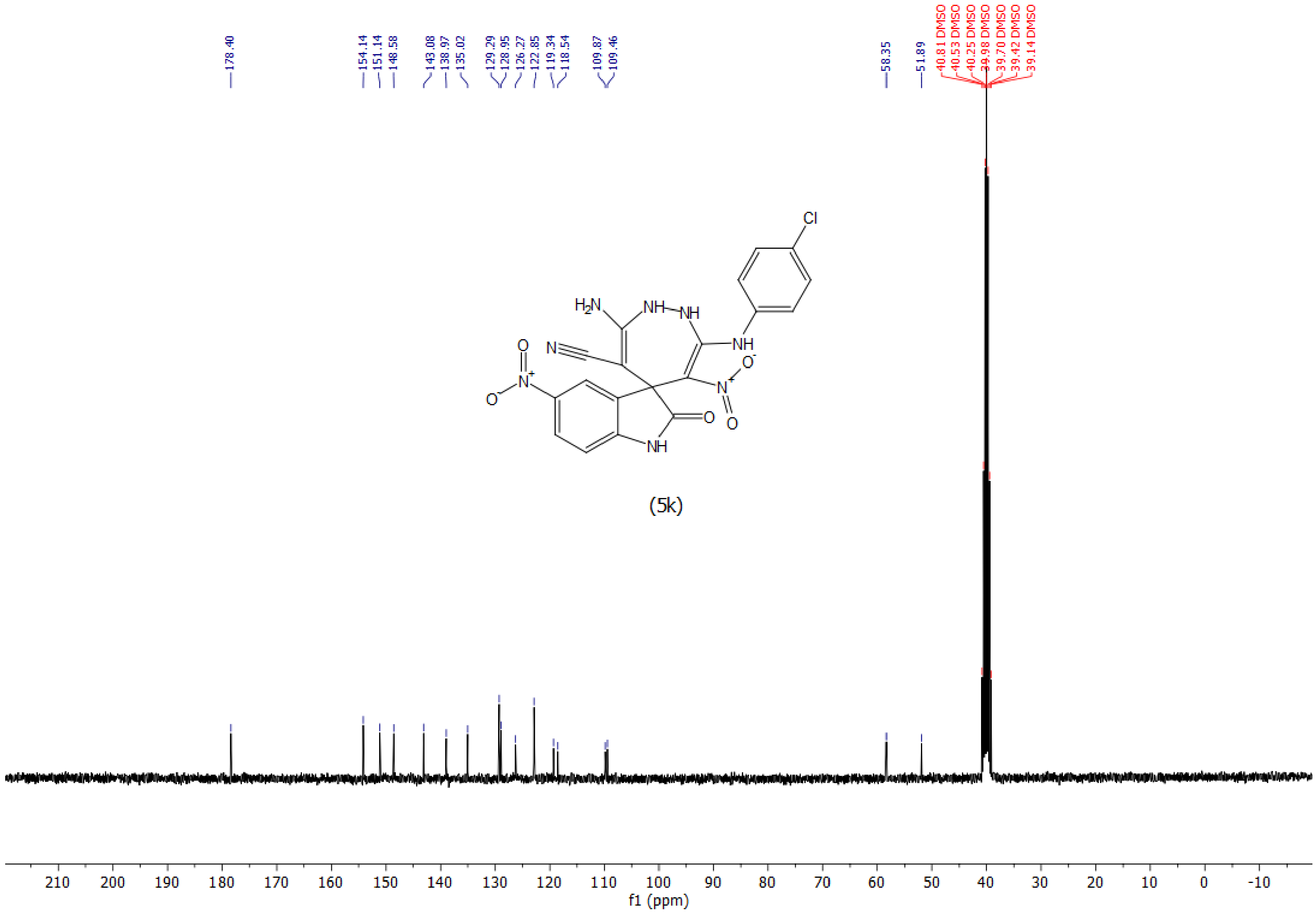
178.40
154.14
151.14
148.58
143.08
138.97
135.02
129.29
128.95
126.27
122.85
119.34
118.54
109.87
109.46
58.35
51.89
40.81 DMSO
40.53 DMSO
40.25 DMSO
39.98 DMSO
39.70 DMSO
39.42 DMSO
39.14 DMSO
Cl
H_2N
NH
NH
NH
N
N^+
O
O^-
NH
(5k)
210 200 190 180 170 160 150 140 130 120 110 100 90 80 70 60 50 40 30 20 10 0 -10
f1 (ppm)

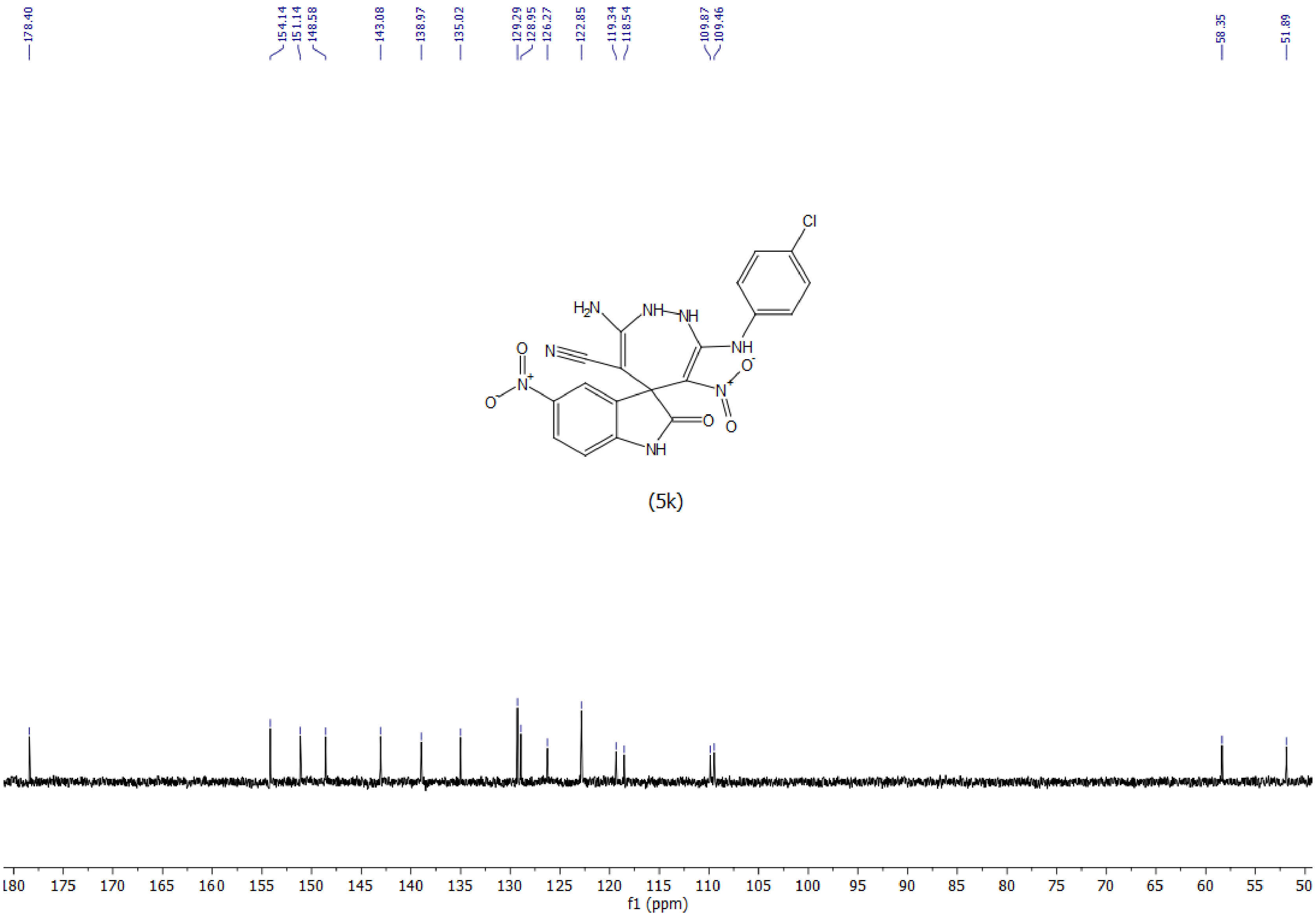
178.40
154.14
151.14
148.58
143.08
138.97
135.02
129.29
128.95
126.27
122.85
119.34
118.54
109.87
109.46
58.35
51.89
Cl
NH
NH
NH
H_2N
N
O
N^+
O^-
N^+
O^-
O
O
NH
(5k)
180 175 170 165 160 155 150 145 140 135 130 125 120 115 110 105 100 95 90 85 80 75 70 65 60 55 50
f1 (ppm)

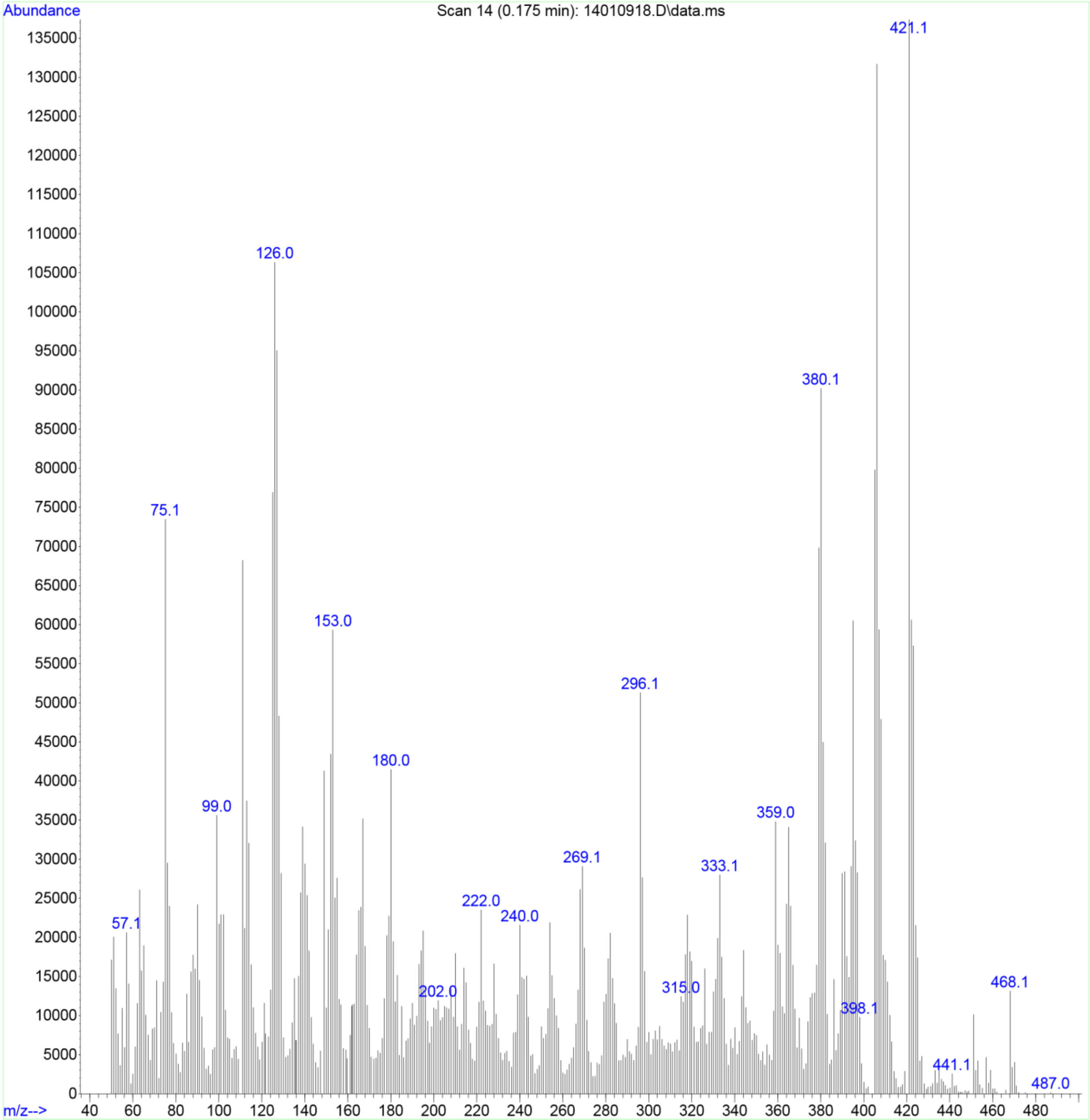

$C_{19}H_{13}ClN_8O_5$ **(5k)**

(468)

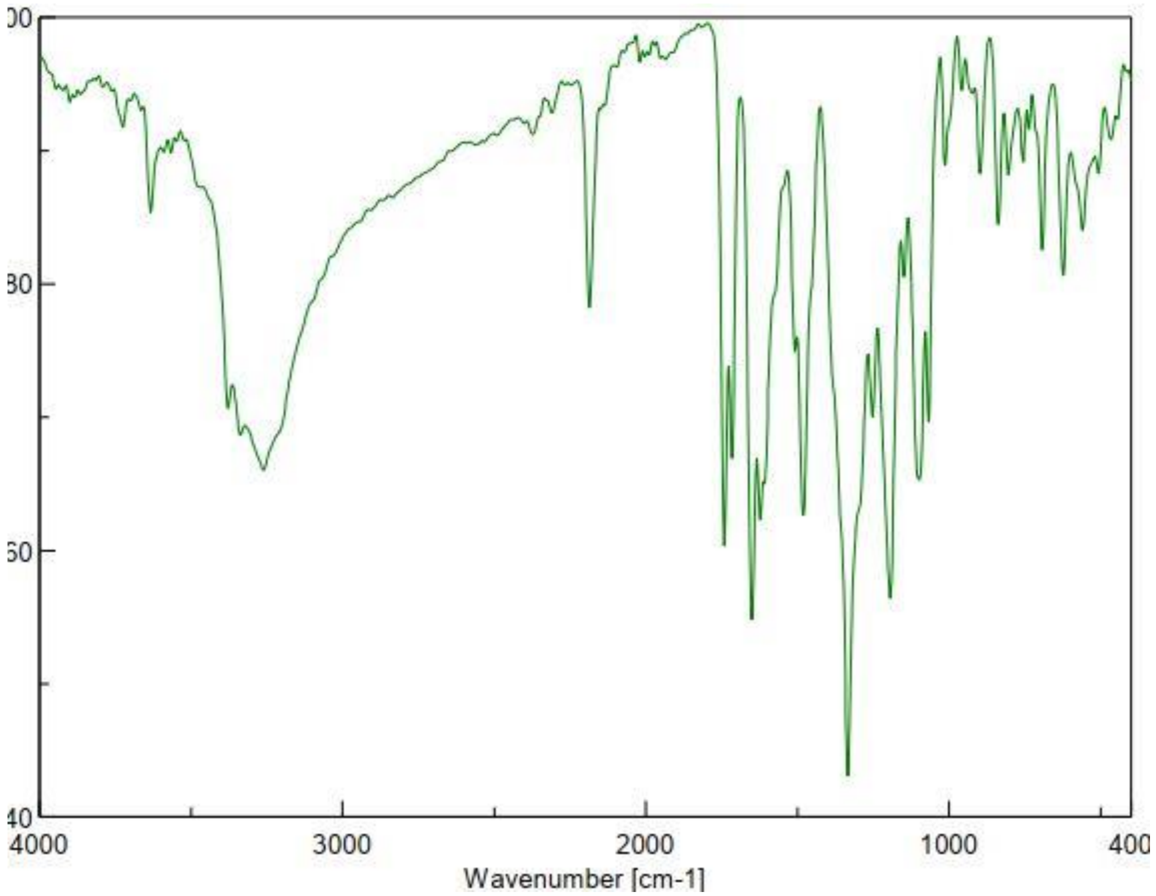

(5k)

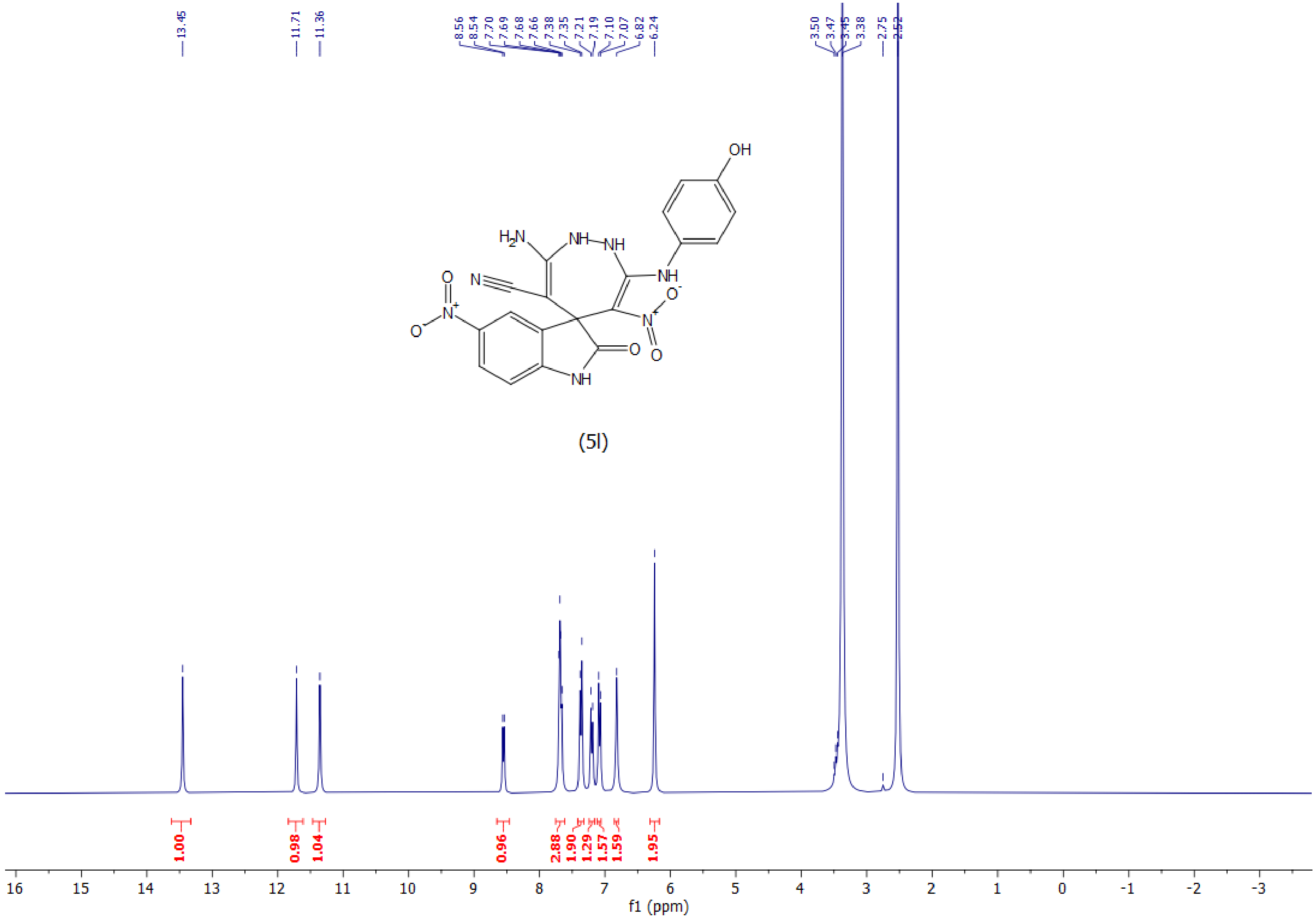
13.45
11.71
11.36
8.56
8.54
7.70
7.69
7.68
7.66
7.38
7.35
7.21
7.19
7.10
7.07
6.82
6.24
3.50
3.47
3.45
3.38
2.75
2.52
1.00
0.98
1.04
0.96
2.88
1.90
1.29
1.57
1.59
1.95
16
15
14
13
12
11
10
9
8
7
6
5
4
3
2
1
0
-1
-2
-3
f1 (ppm)

(5l)

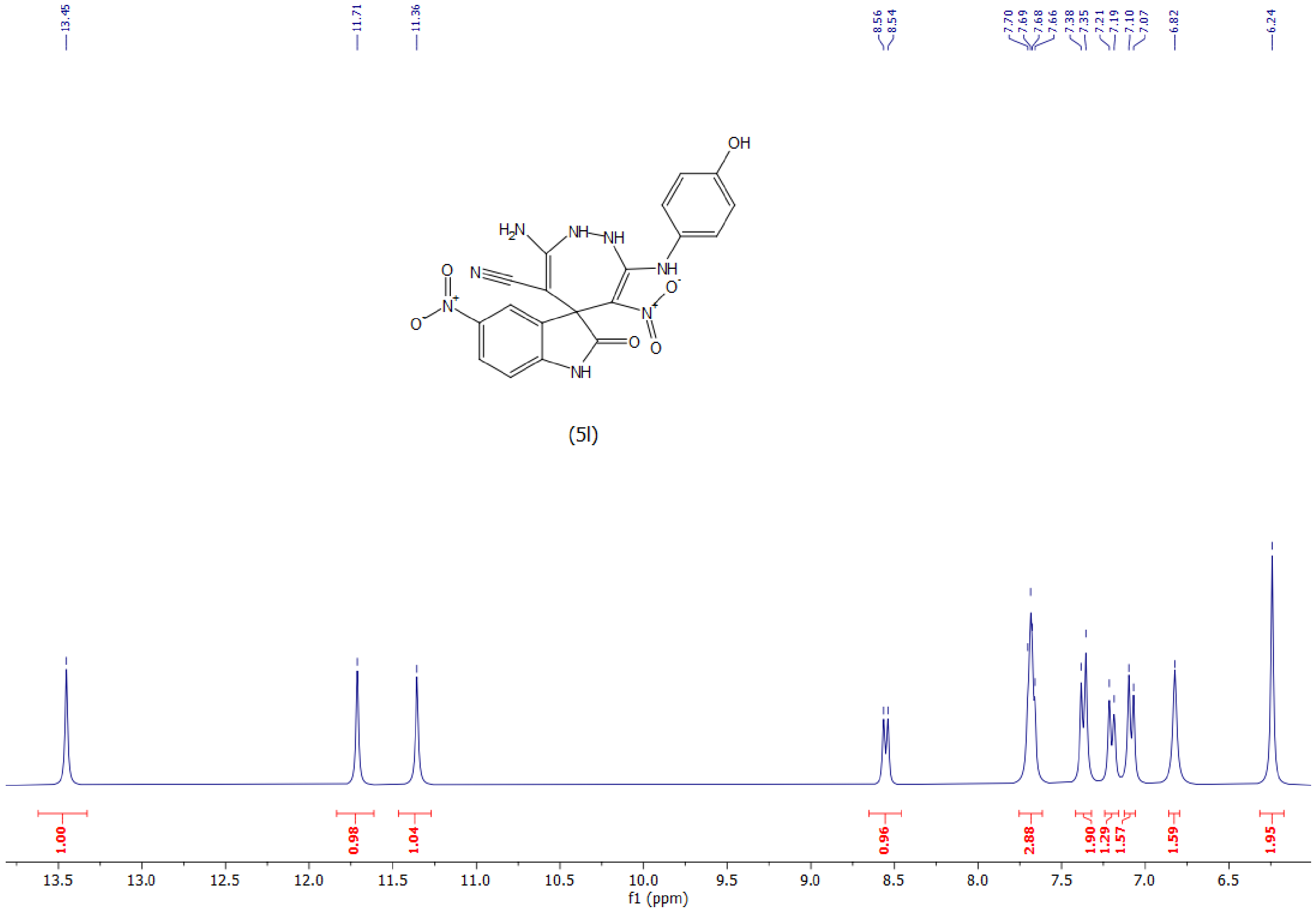

13.45
11.71
11.36
8.56
8.54
7.70
7.69
7.68
7.66
7.38
7.35
7.21
7.19
7.10
7.07
6.82
6.24
OH
H_2N
NH
NH
NH
N
O
N^+
O
O^-
N^+
O^-
O
NH
1.00
0.98
1.04
0.96
2.88
1.90
1.29
1.57
1.59
1.95
13.5
13.0
12.5
12.0
11.5
11.0
10.5
10.0
9.5
9.0
8.5
8.0
7.5
7.0
6.5
f1 (ppm)

(5l)

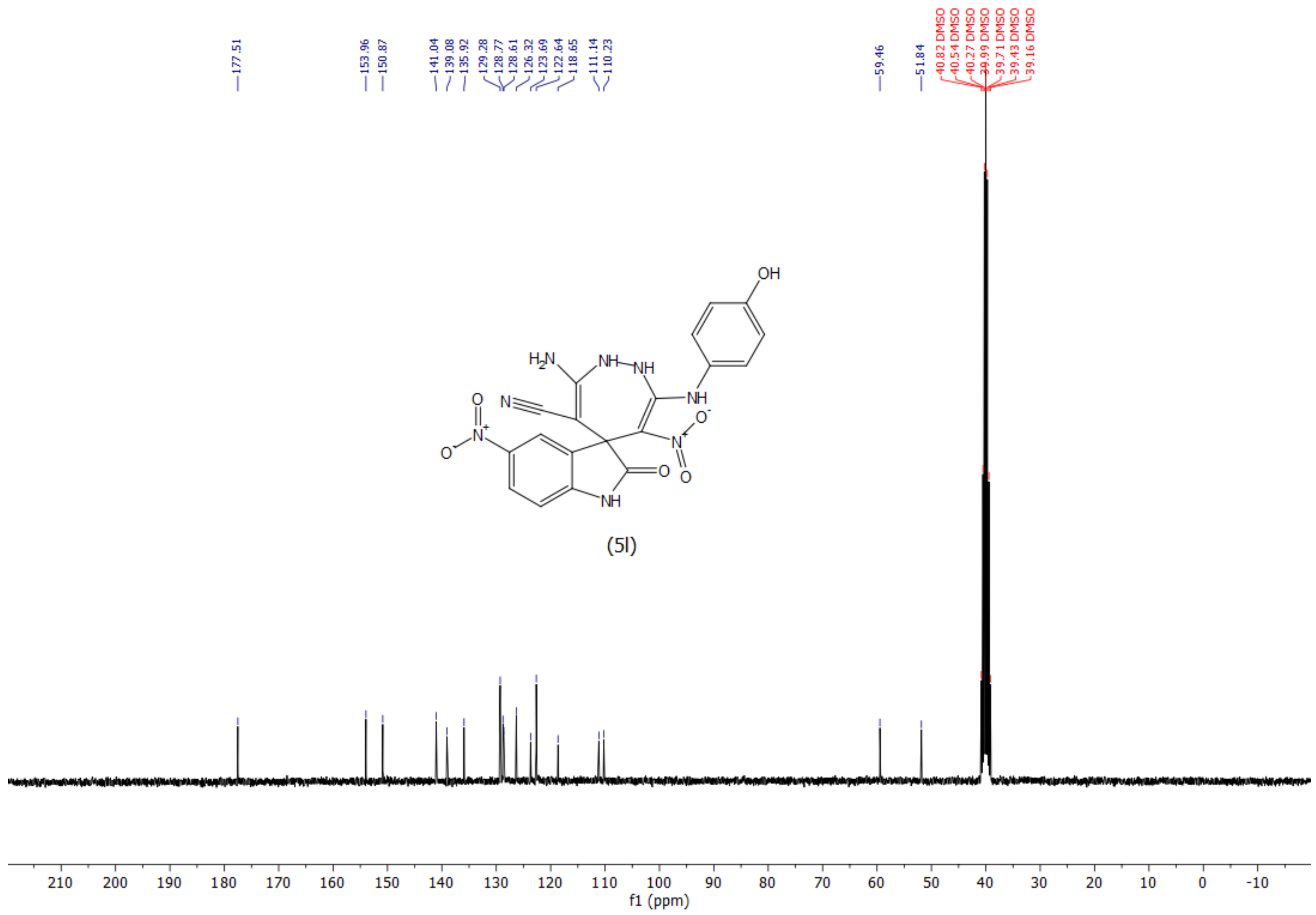
177.51
153.96
150.87
141.04
139.08
135.92
129.28
128.77
128.61
126.32
123.69
122.64
118.65
111.14
110.23
59.46
51.84
40.82 DMSO
40.54 DMSO
40.27 DMSO
39.99 DMSO
39.71 DMSO
39.43 DMSO
39.16 DMSO
(5l)
f1 (ppm)

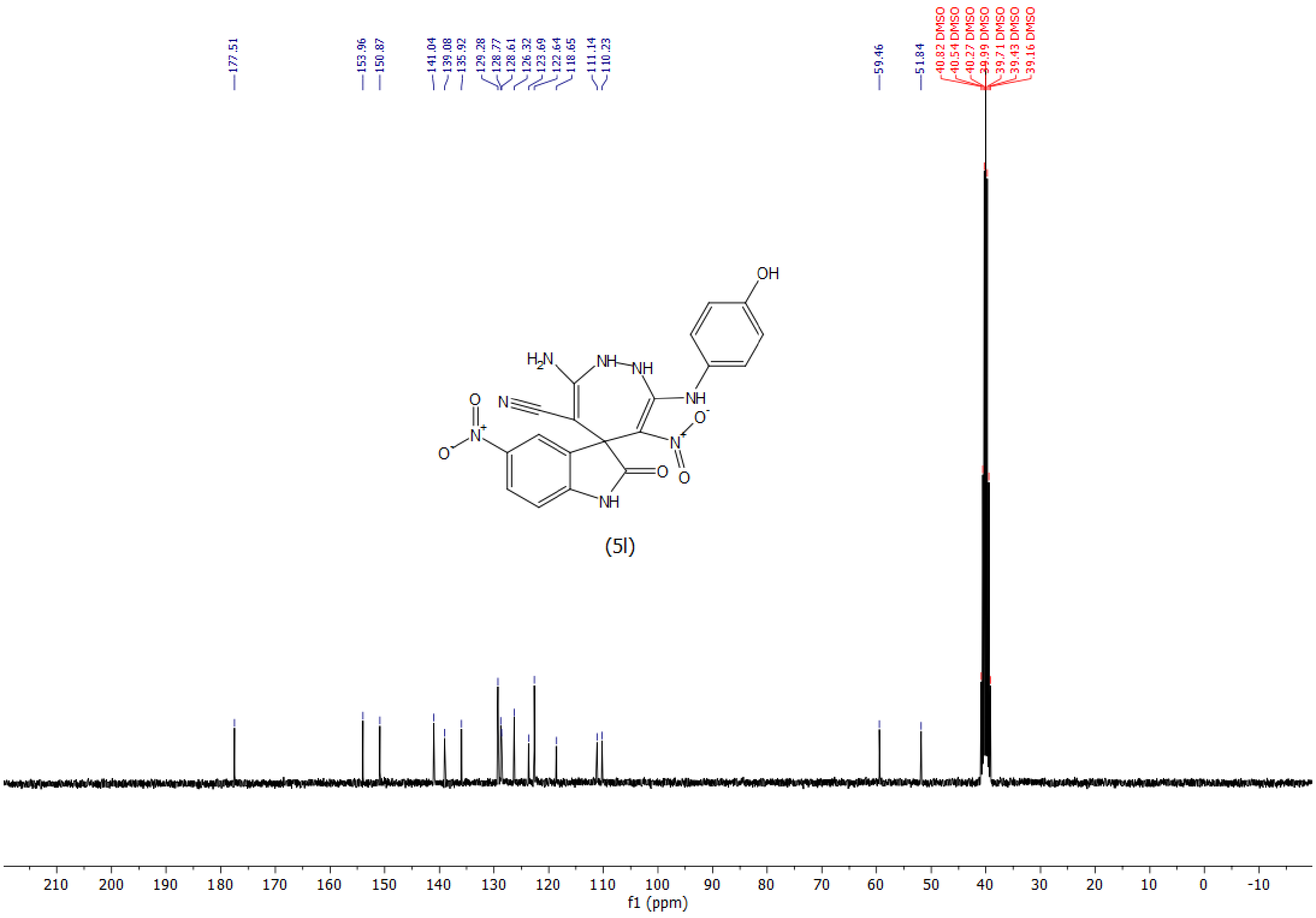

177.51
153.96
150.87
141.04
139.08
135.92
129.28
128.77
128.61
126.32
123.69
122.64
118.65
111.14
110.23
59.46
51.84
40.82 DMSO
40.54 DMSO
40.27 DMSO
39.99 DMSO
39.71 DMSO
39.43 DMSO
39.16 DMSO
H_2N
NH
NH
NH
OH
N
O
N^+
O^-
NH
O
f1 (ppm)

(5l)

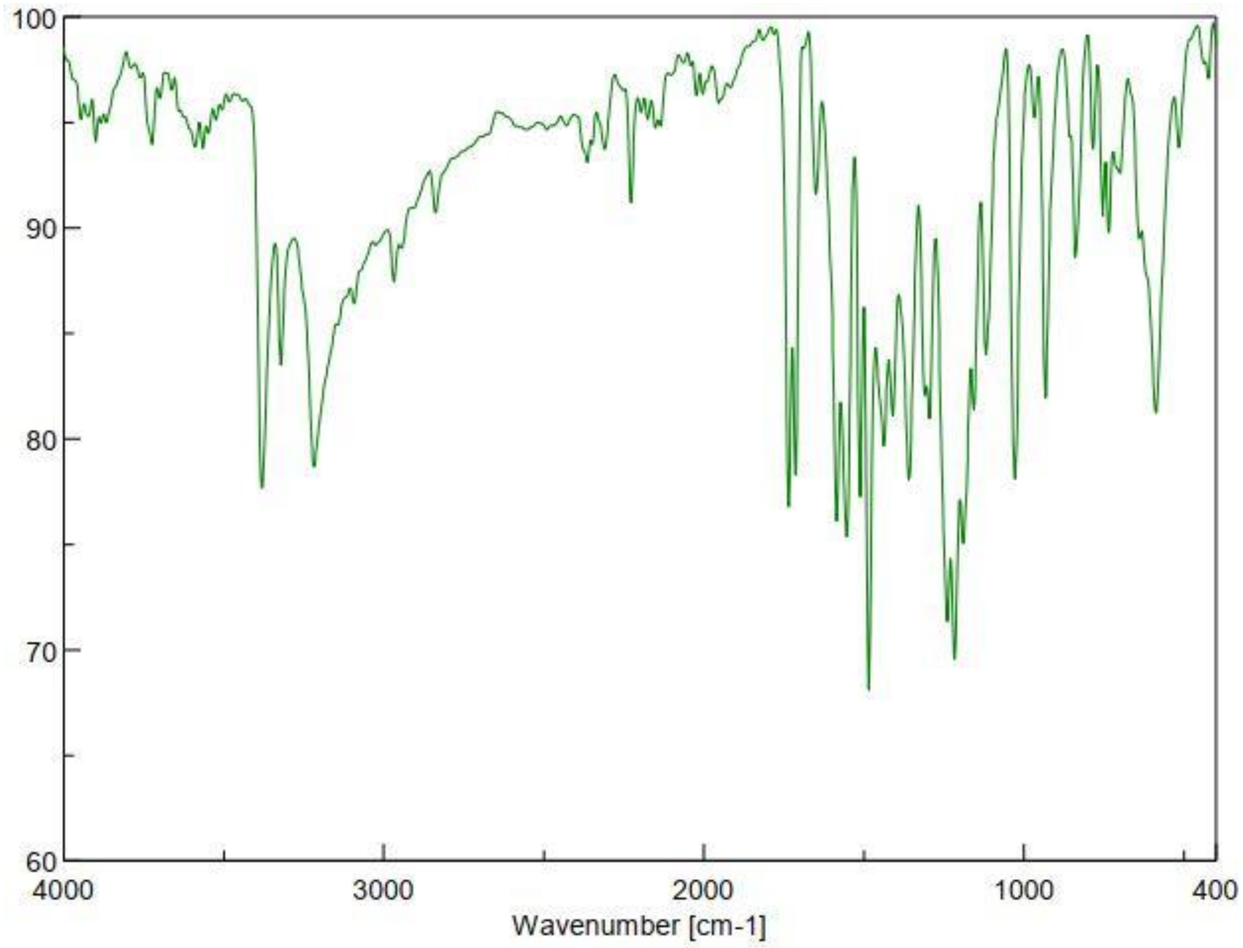

(5l)

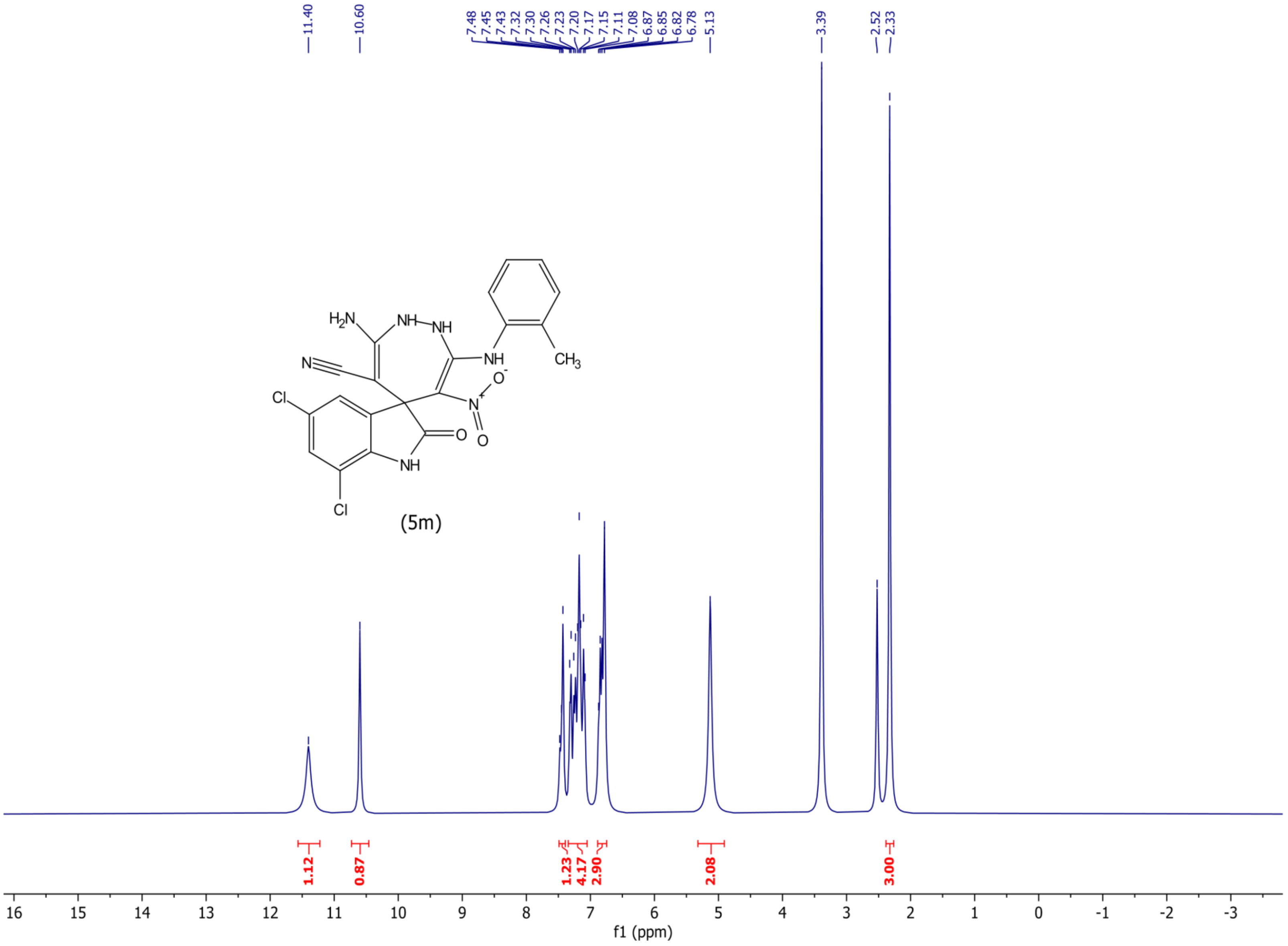

11.40
10.60
7.48
7.45
7.43
7.32
7.30
7.26
7.23
7.20
7.17
7.15
7.11
7.08
6.87
6.85
6.82
6.78
5.13
3.39
2.52
2.33
1.12
0.87
1.23
4.17
2.90
2.08
3.00
f1 (ppm)

(5m)

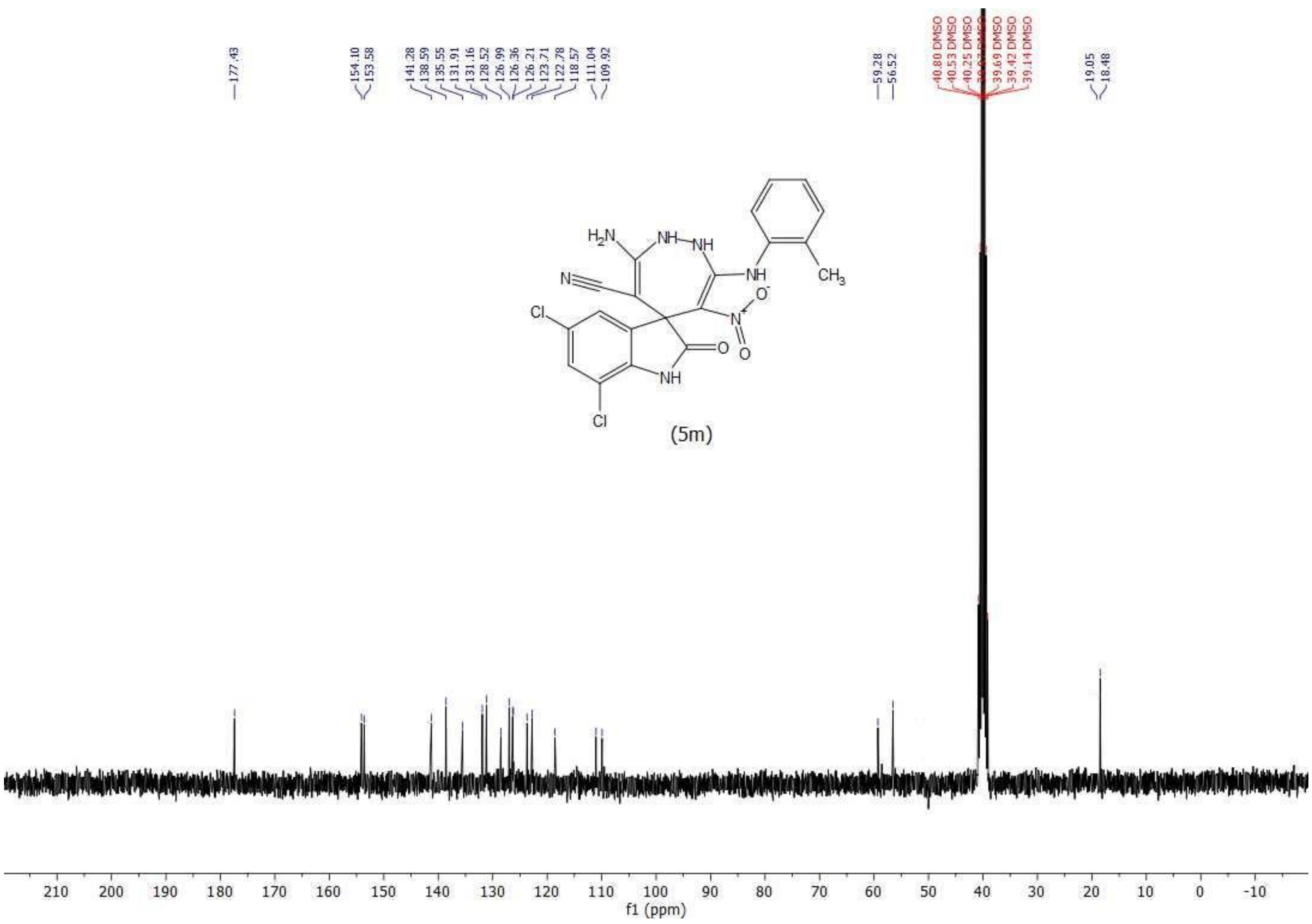
177.43
154.10
153.58
141.28
138.59
135.55
131.91
131.16
128.52
126.99
126.36
126.21
123.71
122.78
118.57
111.04
109.92
59.28
56.52
40.80 DMSO
40.53 DMSO
40.25 DMSO
[illegible] DMSO
39.69 DMSO
39.42 DMSO
39.14 DMSO
19.05
18.48
H_2N
NH
NH
NH
N
CH_3
O^-
N^+
O
O
Cl
Cl
NH
210 200 190 180 170 160 150 140 130 120 110 100 90 80 70 60 50 40 30 20 10 0 -10
f1 (ppm)

(5m)

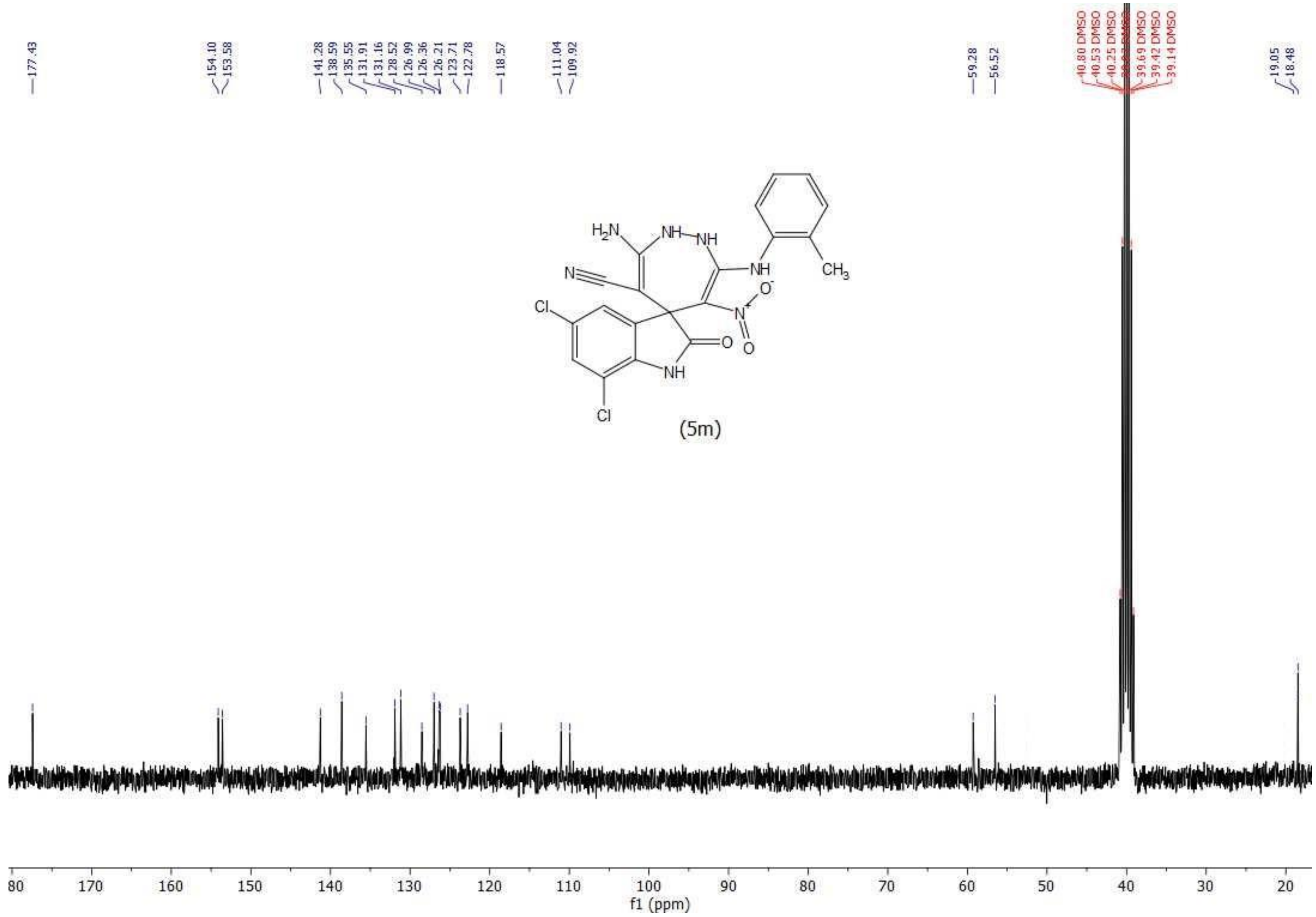
177.43
154.10
153.58
141.28
138.59
135.55
131.91
131.16
128.52
126.99
126.36
126.21
123.71
122.78
118.57
111.04
109.92
59.28
56.52
40.80 DMSO
40.53 DMSO
40.25 DMSO
39.69 DMSO
39.42 DMSO
39.14 DMSO
19.05
18.48
(5m)
f1 (ppm)

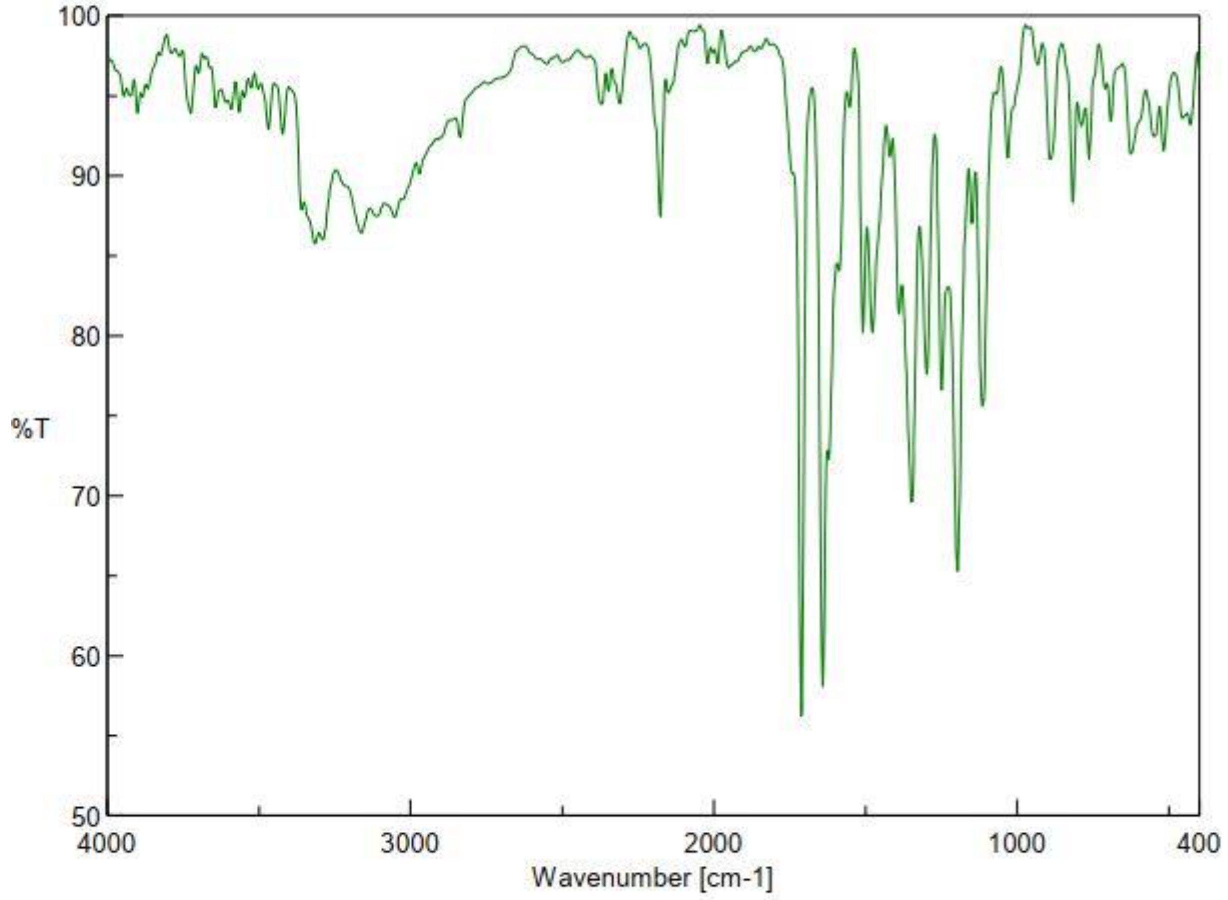

(5m)

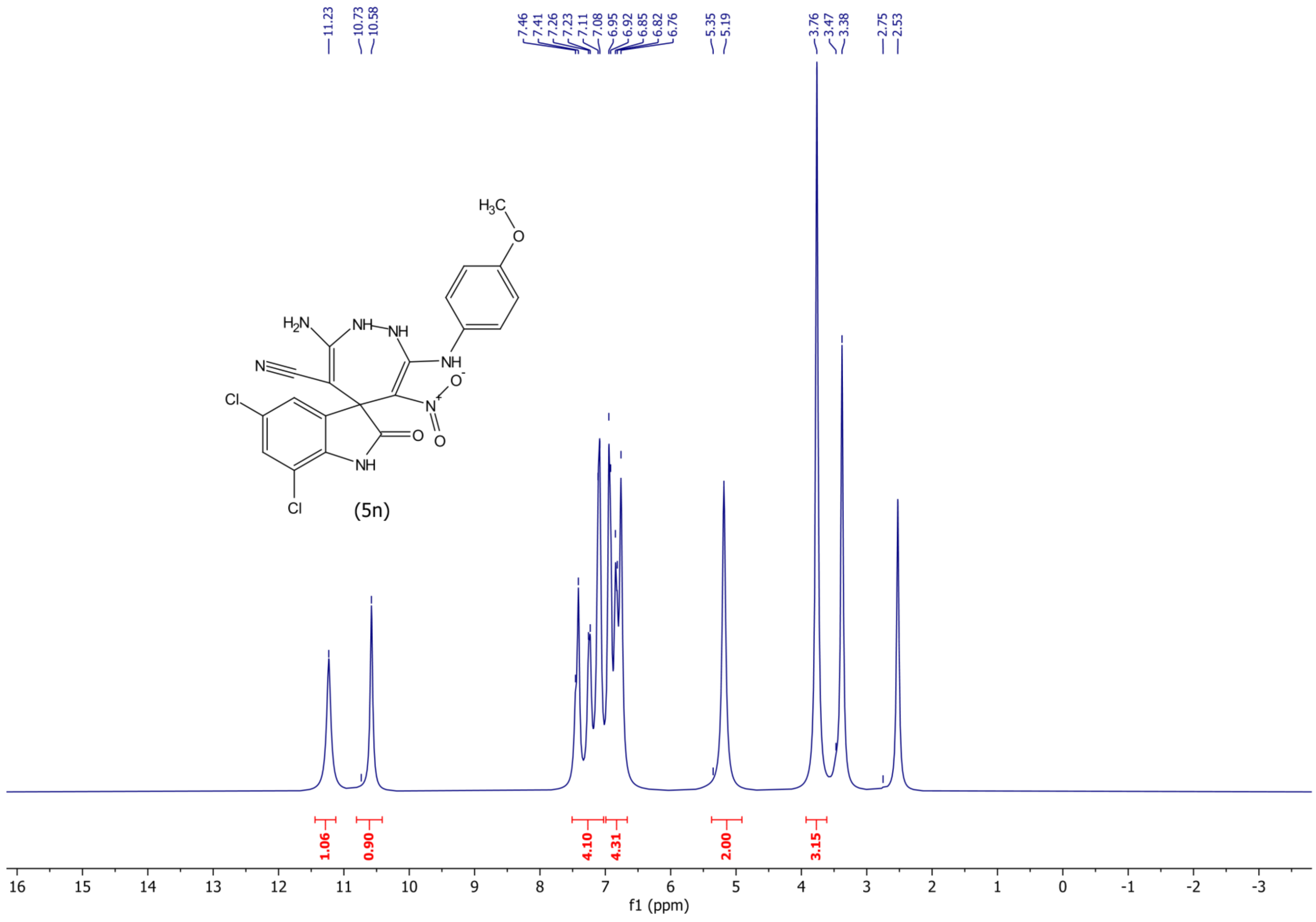

11.23
10.73
10.58
7.46
7.41
7.26
7.23
7.11
7.08
6.95
6.92
6.85
6.82
6.76
5.35
5.19
3.76
3.47
3.38
2.75
2.53
H$_3$C
O
H$_2$N
NH
NH
NH
N
O
N$^+$
O$^-$
O
Cl
Cl
NH
(5n)
1.06
0.90
4.10
4.31
2.00
3.15
16
15
14
13
12
11
10
9
8
7
6
5
4
3
2
1
0
-1
-2
-3
f1 (ppm)

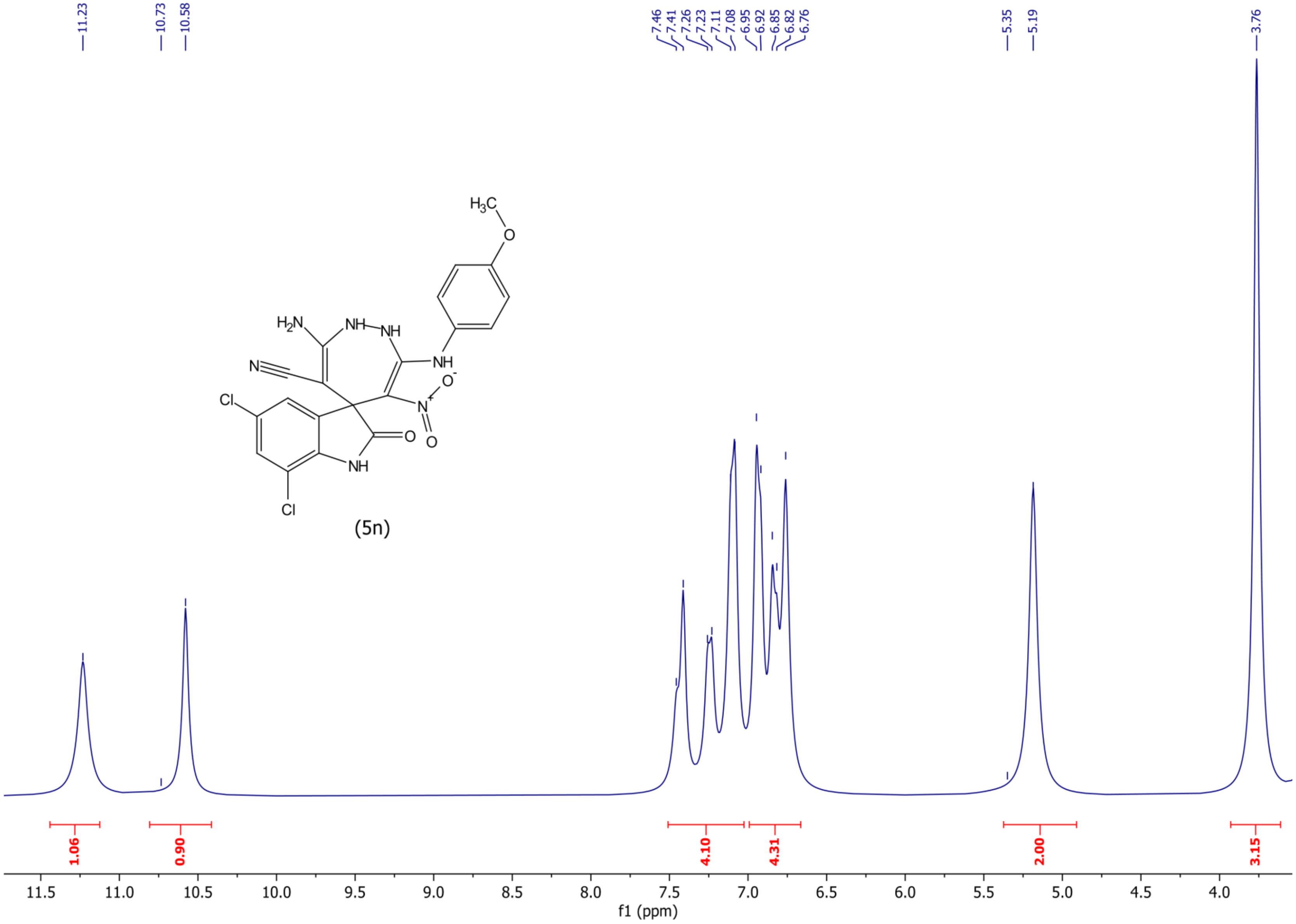

11.23
10.73
10.58
7.46
7.41
7.26
7.23
7.11
7.08
6.95
6.92
6.85
6.82
6.76
5.35
5.19
3.76
1.06
0.90
4.10
4.31
2.00
3.15
11.5
11.0
10.5
10.0
9.5
9.0
8.5
8.0
7.5
7.0
6.5
6.0
5.5
5.0
4.5
4.0
f1 (ppm)

(5n)

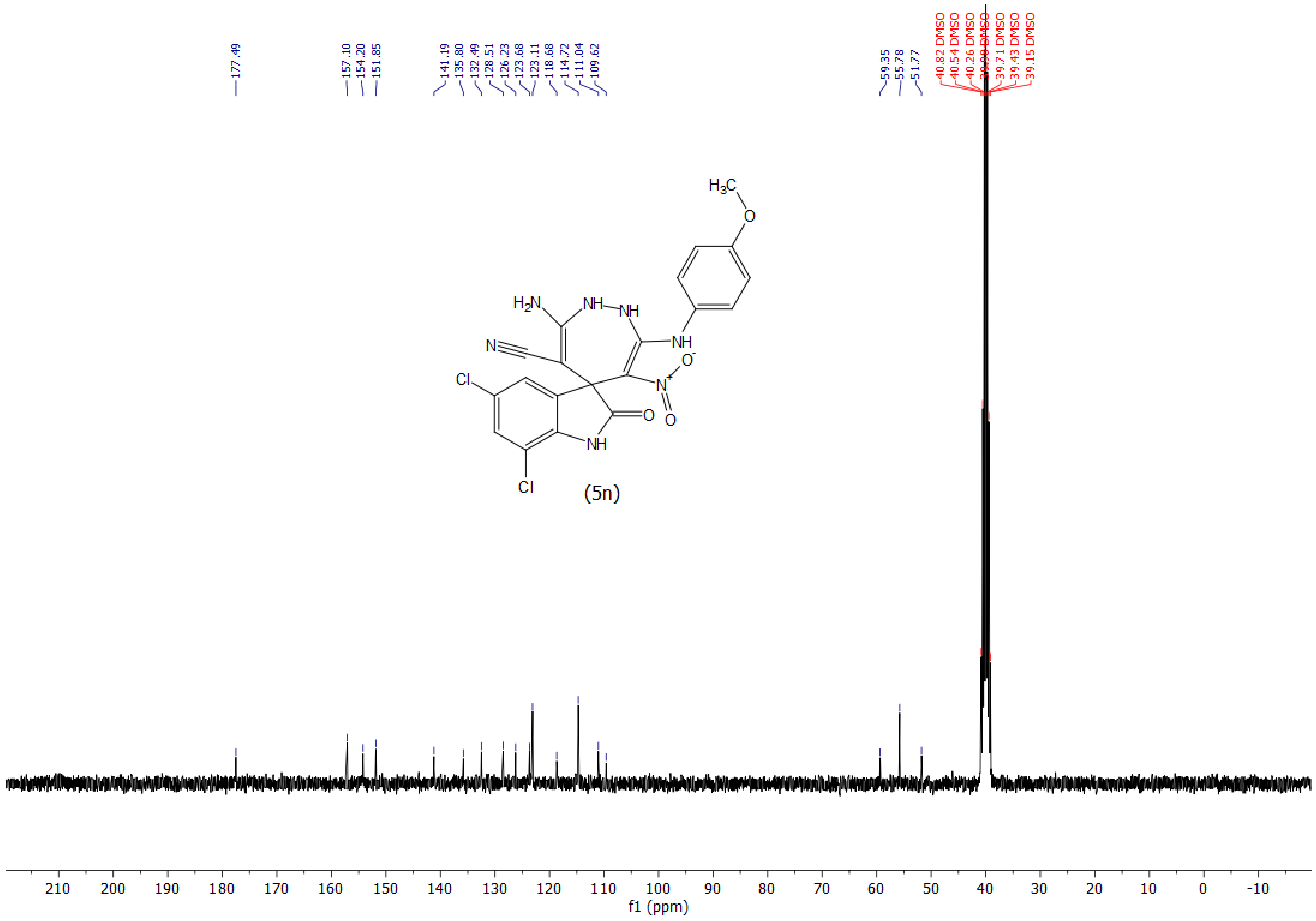
177.49
157.10
154.20
151.85
141.19
135.80
132.49
128.51
126.23
123.68
123.11
118.68
114.72
111.04
109.62
59.35
55.78
51.77
40.82 DMSO
40.54 DMSO
40.26 DMSO
39.71 DMSO
39.43 DMSO
39.15 DMSO
(5n)
210 200 190 180 170 160 150 140 130 120 110 100 90 80 70 60 50 40 30 20 10 0 -10
f1 (ppm)

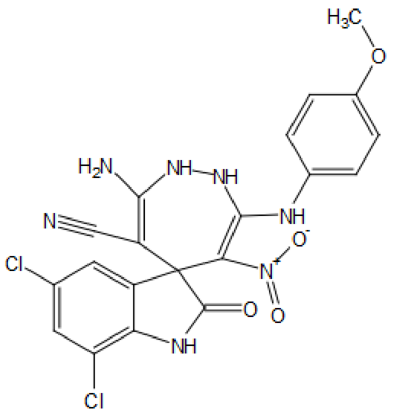
H$_3$C
O
H$_2$N
NH
NH
NH
N
O$^-$
N$^+$
O
Cl
O
NH
Cl

(5n)

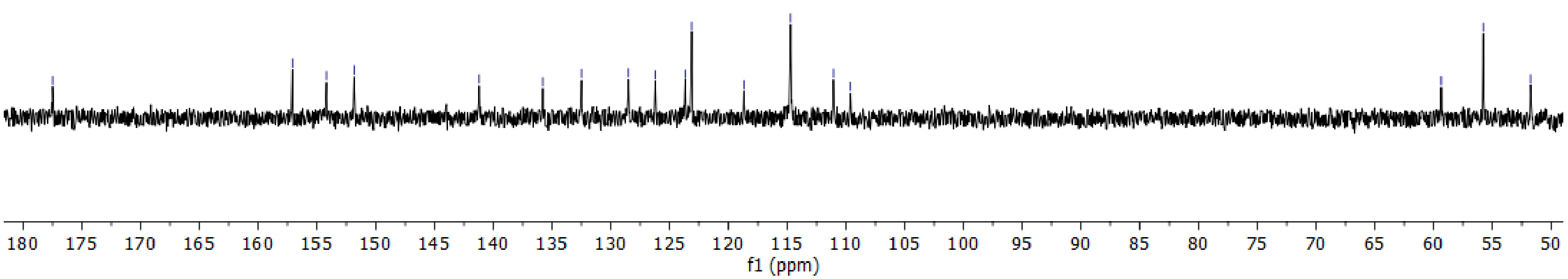
177.49
157.10
154.20
151.85
141.19
135.80
132.49
128.51
126.23
123.68
123.11
118.68
114.72
111.04
109.62
59.35
55.78
51.77
180
175
170
165
160
155
150
145
140
135
130
125
120
115
110
105
100
95
90
85
80
75
70
65
60
55
50
f1 (ppm)

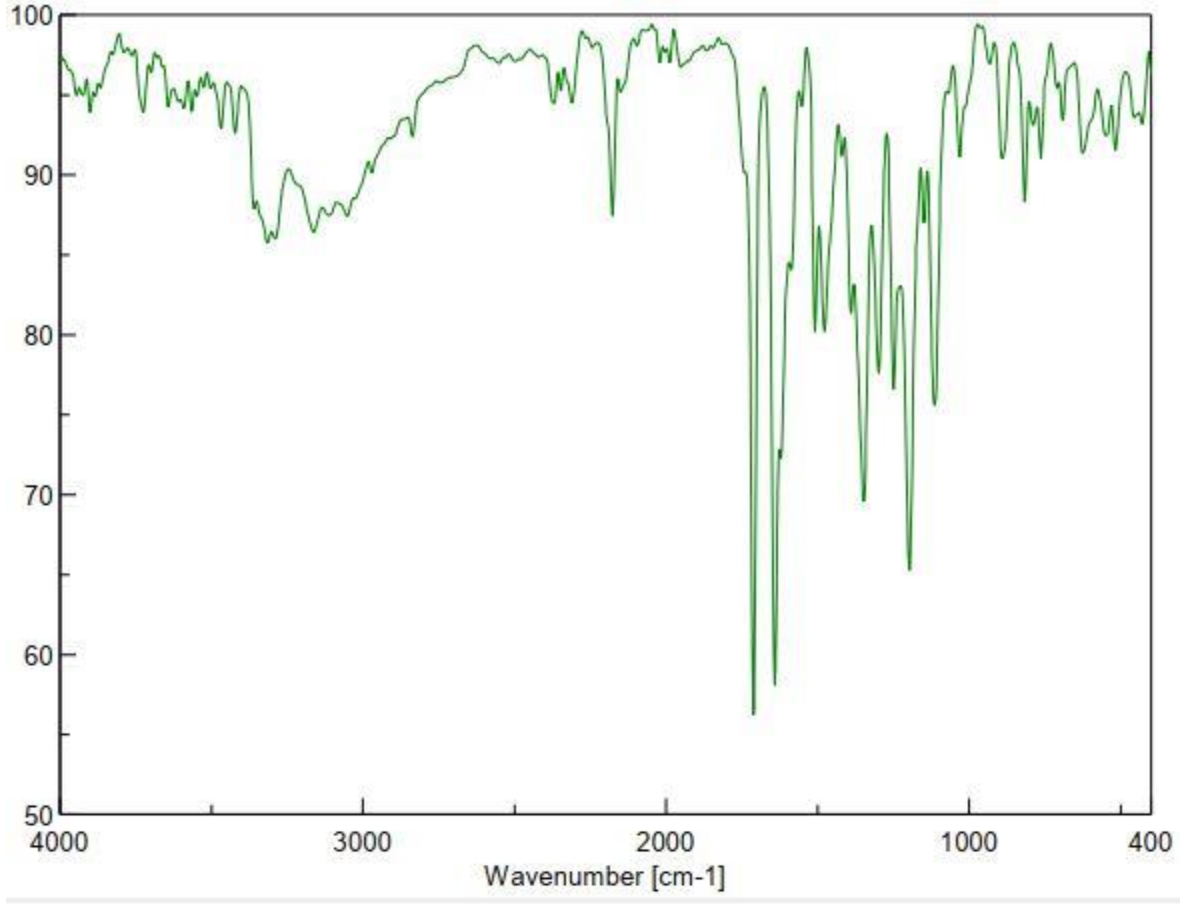

(5n)

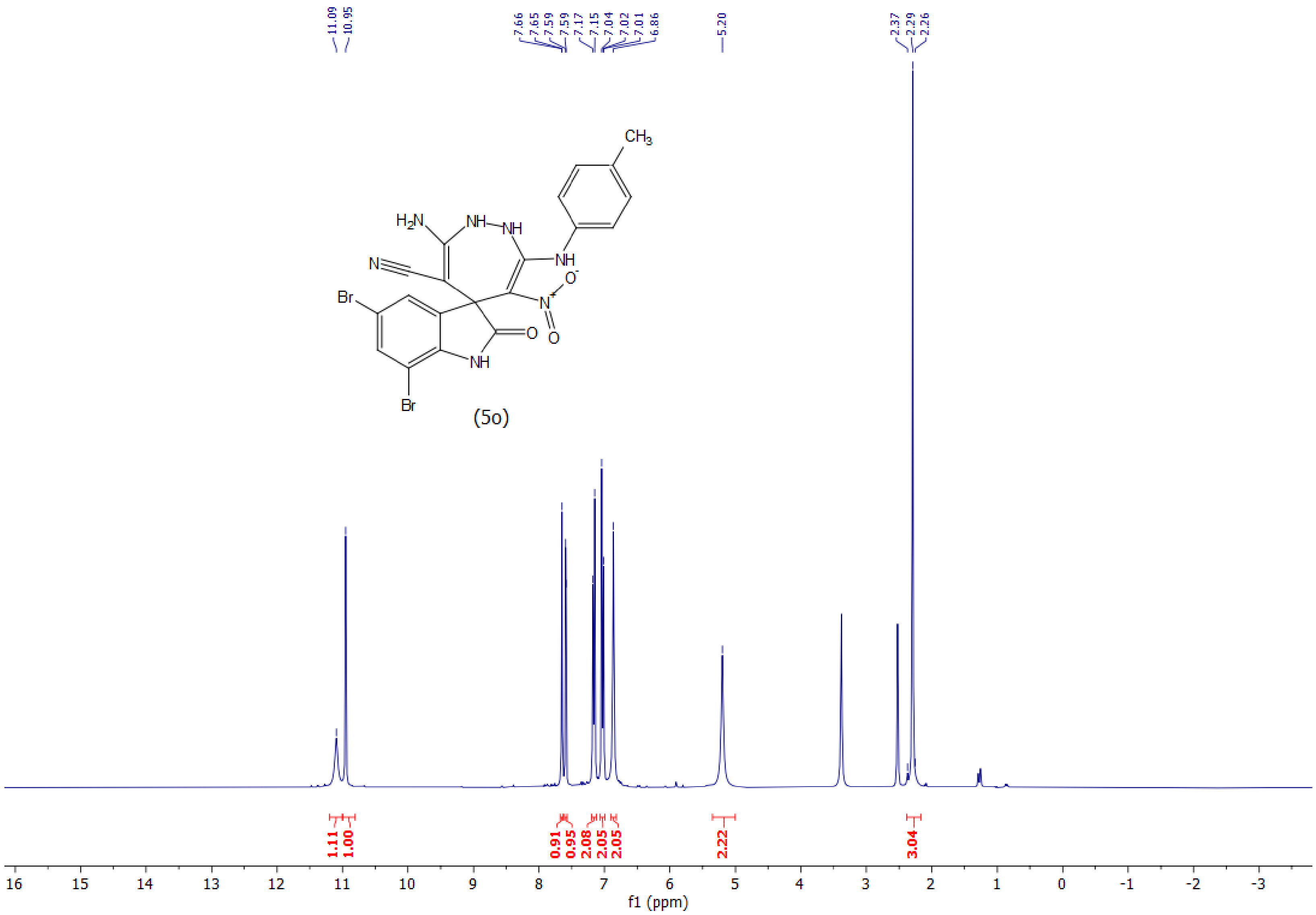
11.09
10.95
7.66
7.65
7.59
7.59
7.17
7.15
7.04
7.02
7.01
6.86
5.20
2.37
2.29
2.26
CH3
H2N
NH
NH
NH
N
O-
N+
O
Br
O
NH
Br
(5o)
1.11
1.00
0.91
0.95
2.08
2.05
2.05
2.22
3.04
16
15
14
13
12
11
10
9
8
7
6
5
4
3
2
1
0
-1
-2
-3
f1 (ppm)

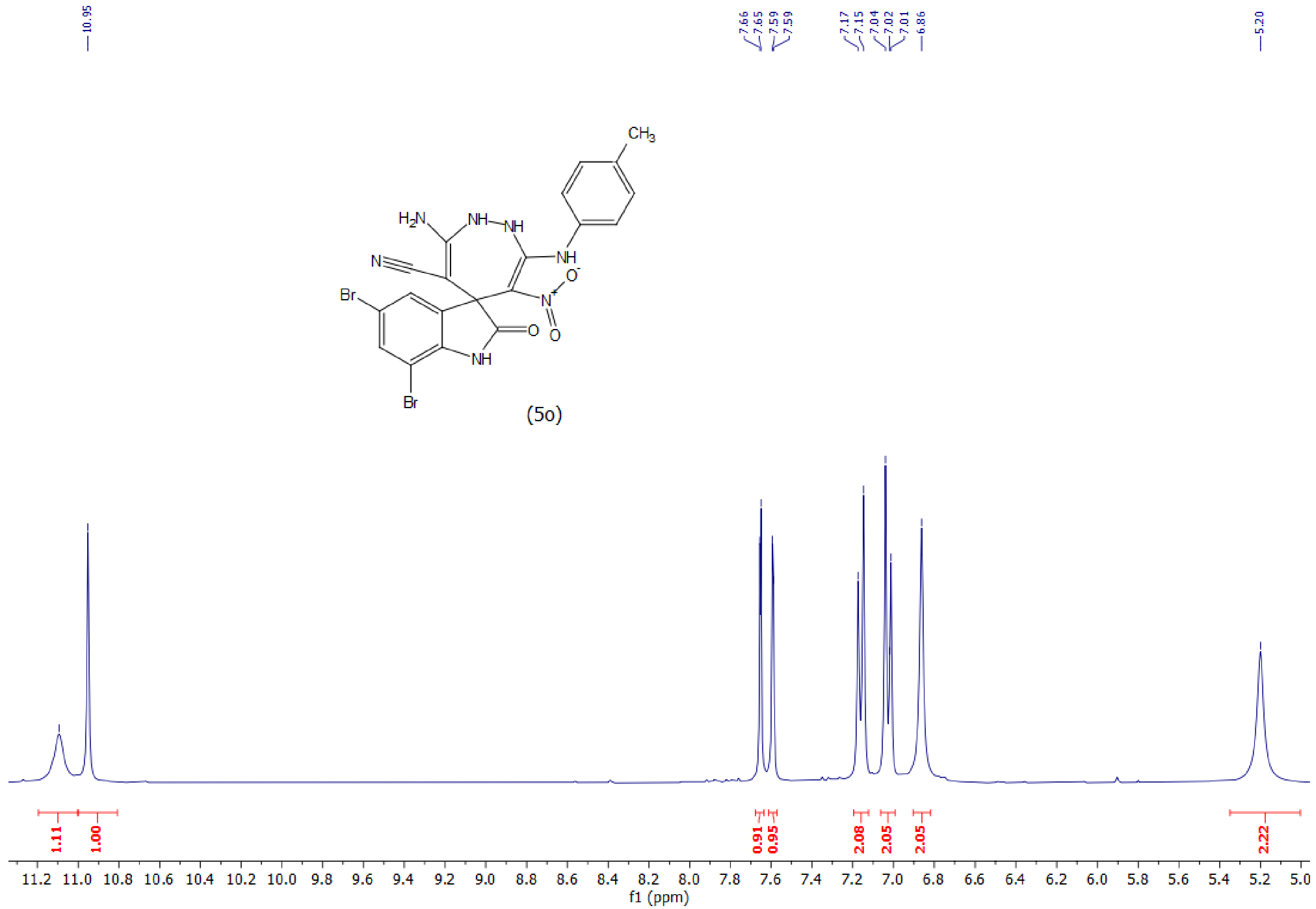

10.95
7.66
7.65
7.59
7.59
7.17
7.15
7.04
7.02
7.01
6.86
5.20
CH3
H2N
NH
NH
NH
O-
N+
O
N
Br
Br
NH
O
1.11
1.00
0.91
0.95
2.08
2.05
2.05
2.22
11.2 11.0 10.8 10.6 10.4 10.2 10.0 9.8 9.6 9.4 9.2 9.0 8.8 8.6 8.4 8.2 8.0 7.8 7.6 7.4 7.2 7.0 6.8 6.6 6.4 6.2 6.0 5.8 5.6 5.4 5.2 5.0
f1 (ppm)

(5o)

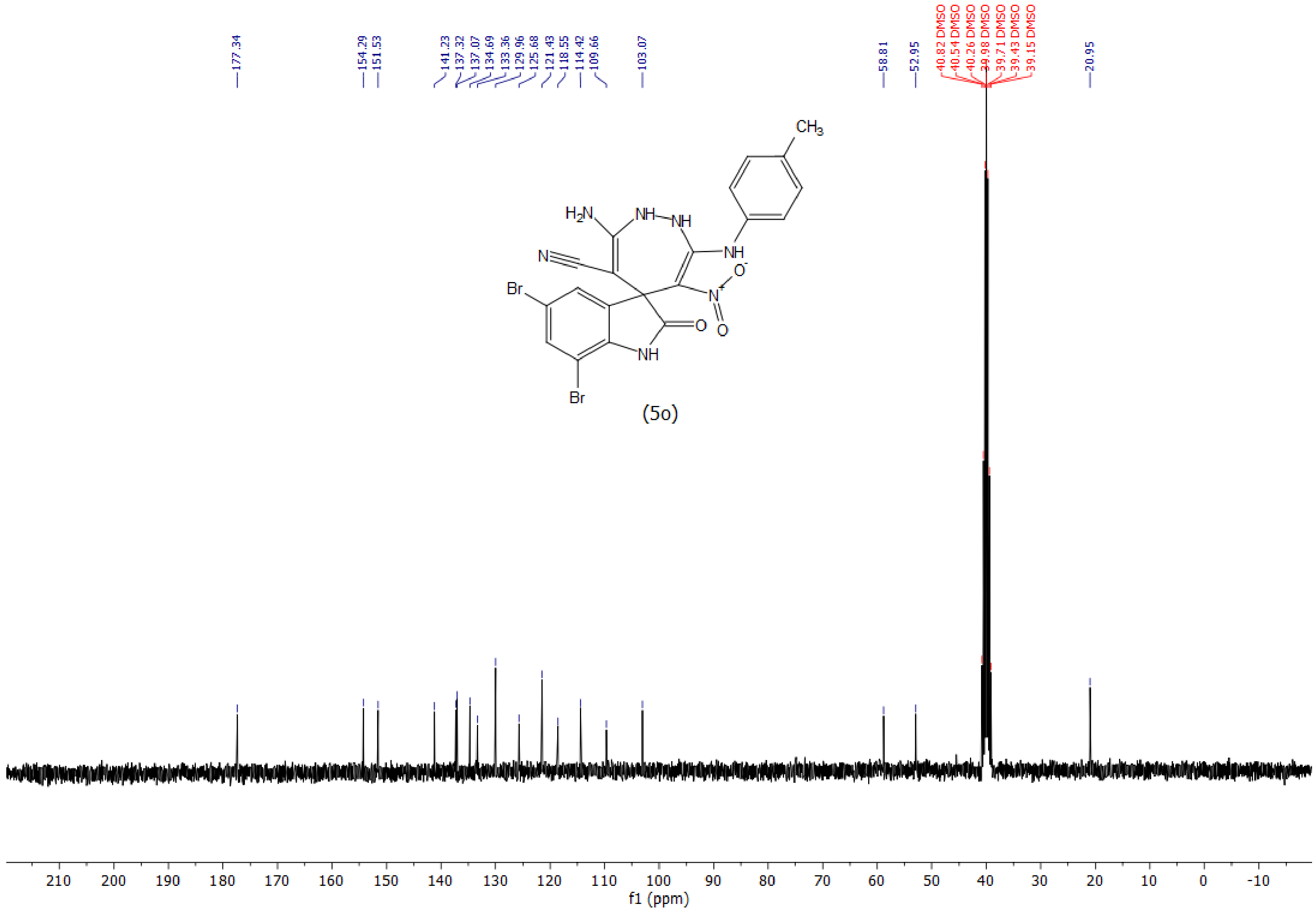
177.34
154.29
151.53
141.23
137.32
137.07
134.69
133.36
129.96
125.68
121.43
118.55
114.42
109.66
103.07
58.81
52.95
40.82 DMSO
40.54 DMSO
40.26 DMSO
39.98 DMSO
39.71 DMSO
39.43 DMSO
39.15 DMSO
20.95
CH3
H2N
NH
NH
NH
N
O
Br
Br
NH
O
O
(5o)
210 200 190 180 170 160 150 140 130 120 110 100 90 80 70 60 50 40 30 20 10 0 -10
f1 (ppm)

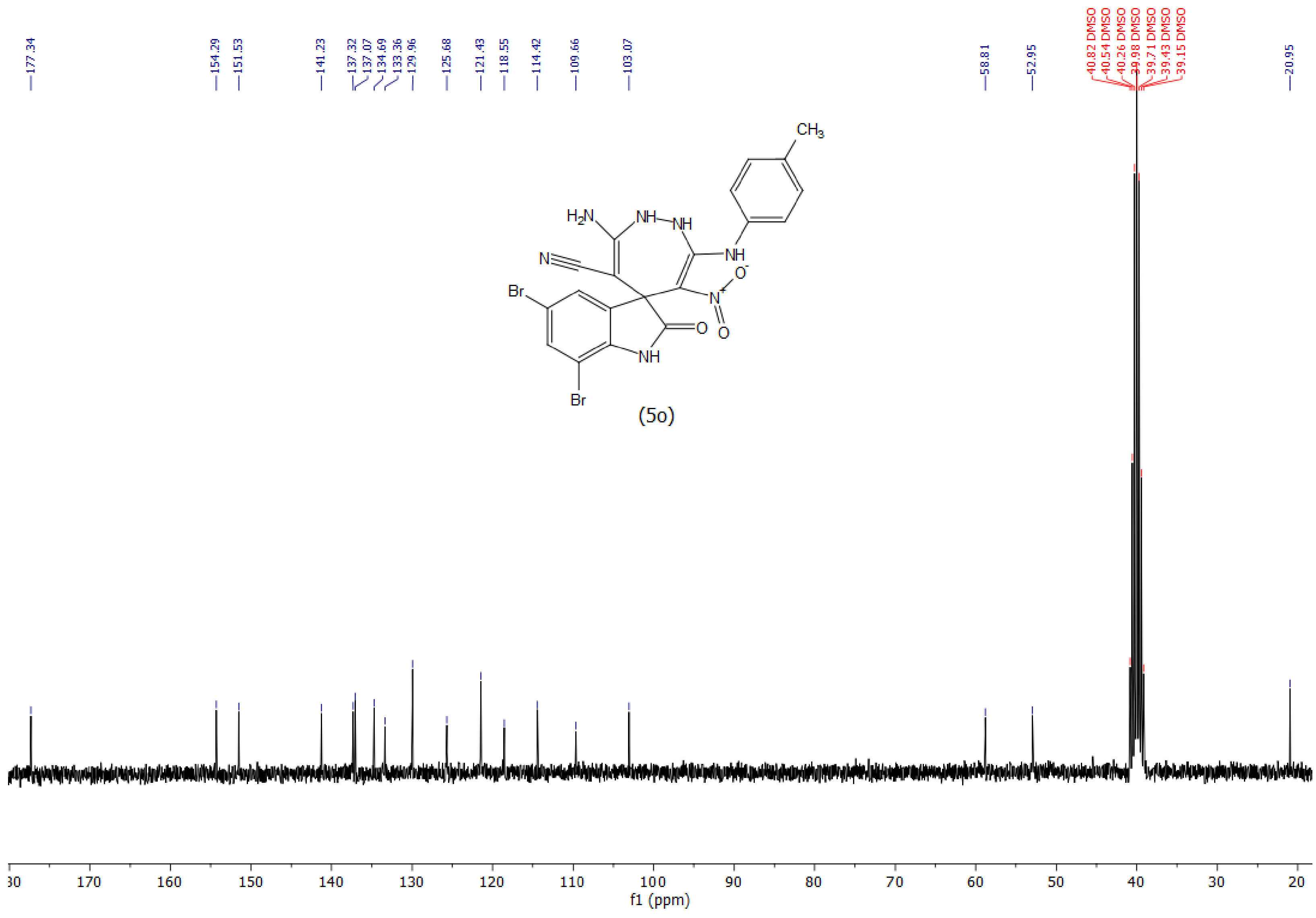
177.34
154.29
151.53
141.23
137.32
137.07
134.69
133.36
129.96
125.68
121.43
118.55
114.42
109.66
103.07
58.81
52.95
40.82 DMSO
40.54 DMSO
40.26 DMSO
39.98 DMSO
39.71 DMSO
39.43 DMSO
39.15 DMSO
20.95
CH3
H2N
NH
NH
NH
N
Br
O-
N+
O
O
NH
Br
(5o)
f1 (ppm)

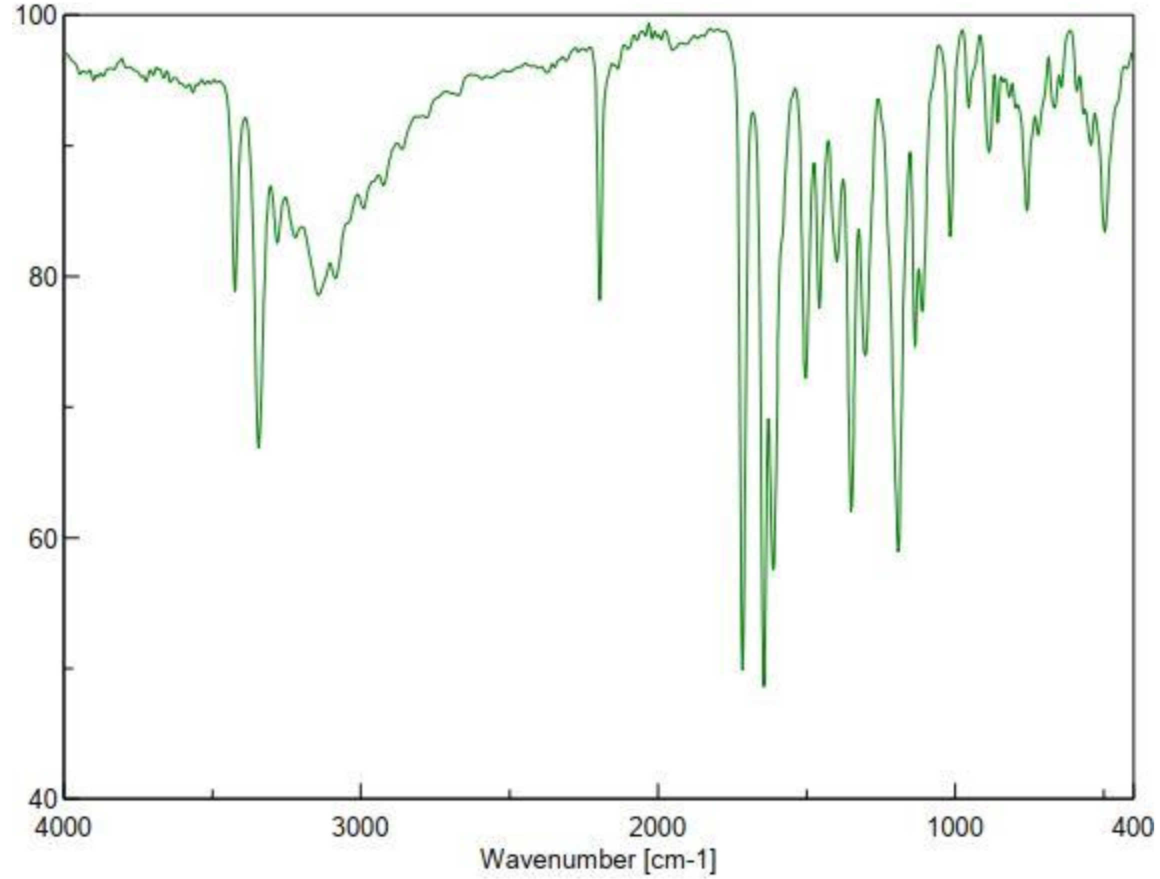

(5o)

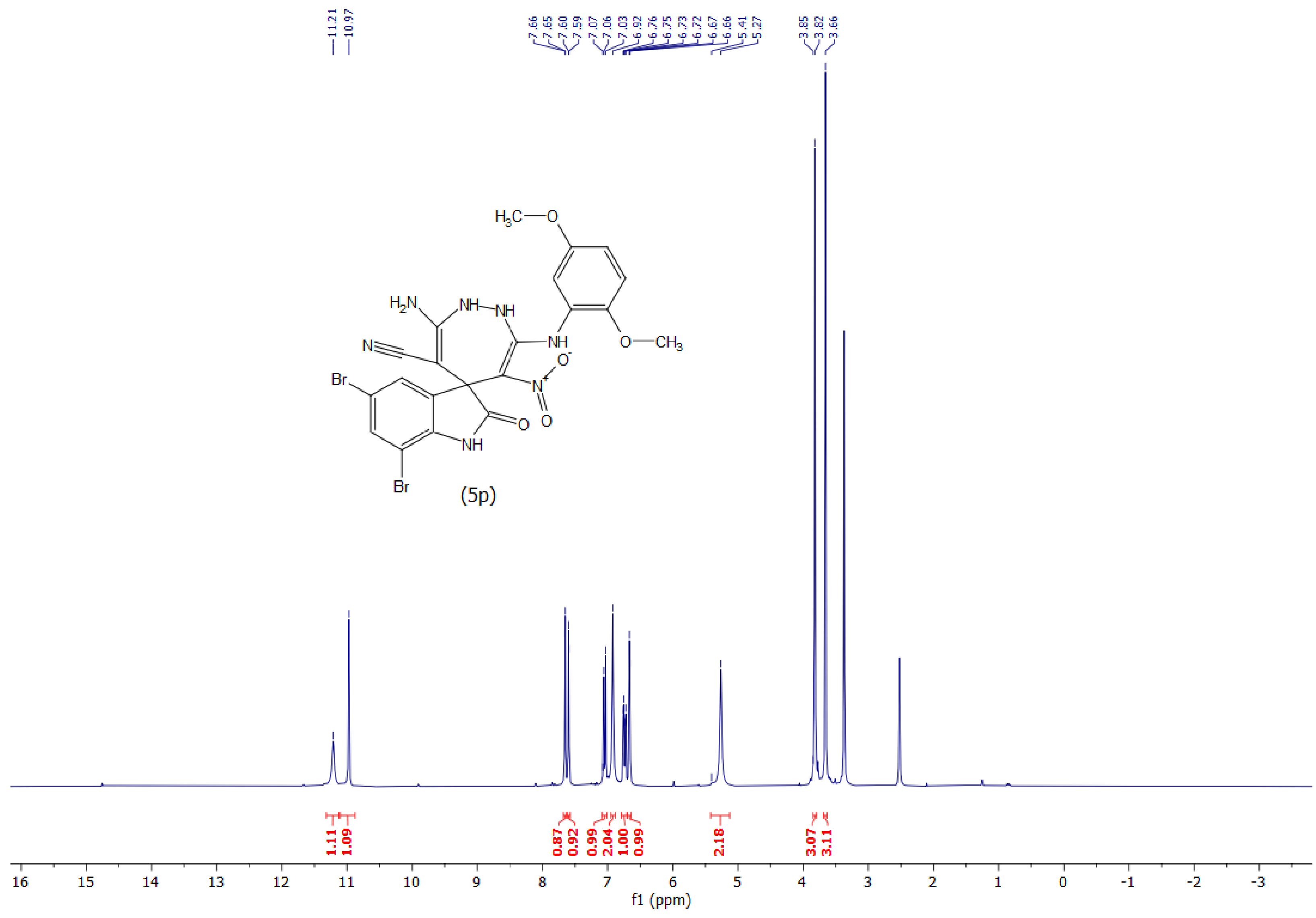
11.21
10.97
7.66
7.65
7.60
7.59
7.07
7.06
7.03
6.92
6.76
6.75
6.73
6.72
6.67
6.66
5.41
5.27
3.85
3.82
3.66
H3C—O
H2N
NH—NH
NH
O—CH3
N≡
O-
N+
O
Br
Br
NH
(5p)
1.11
1.09
0.87
0.92
0.99
2.04
1.00
0.99
2.18
3.07
3.11
16
15
14
13
12
11
10
9
8
7
6
5
4
3
2
1
0
-1
-2
-3
f1 (ppm)

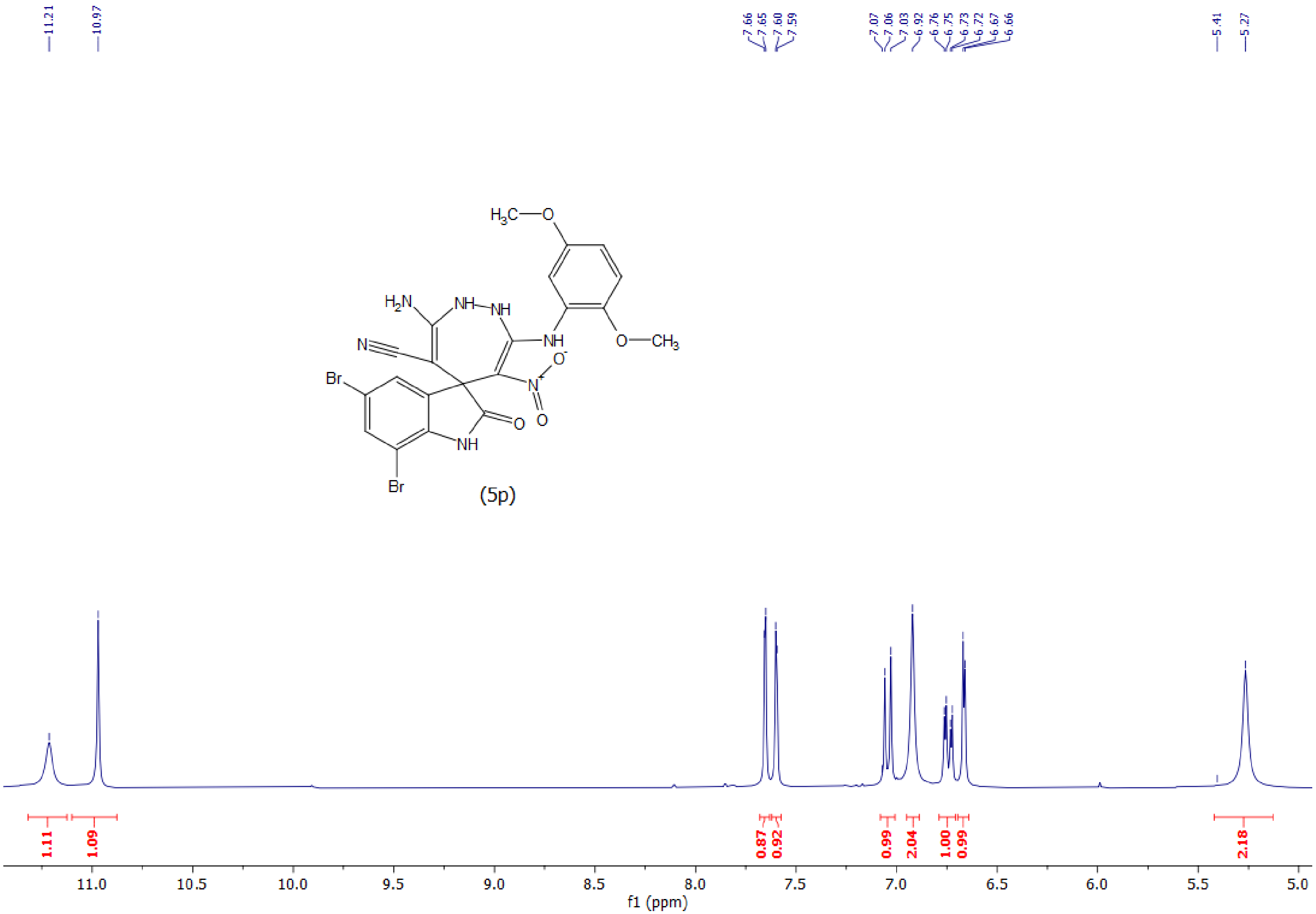
11.21
10.97
7.66
7.65
7.60
7.59
7.07
7.06
7.03
6.92
6.76
6.75
6.73
6.72
6.67
6.66
5.41
5.27
H3C—O
H2N
NH—NH
NH
N
O-
N+
O
O—CH3
Br
Br
NH
O
(5p)
1.11
1.09
0.87
0.92
0.99
2.04
1.00
0.99
2.18
11.0
10.5
10.0
9.5
9.0
8.5
8.0
7.5
7.0
6.5
6.0
5.5
5.0
f1 (ppm)

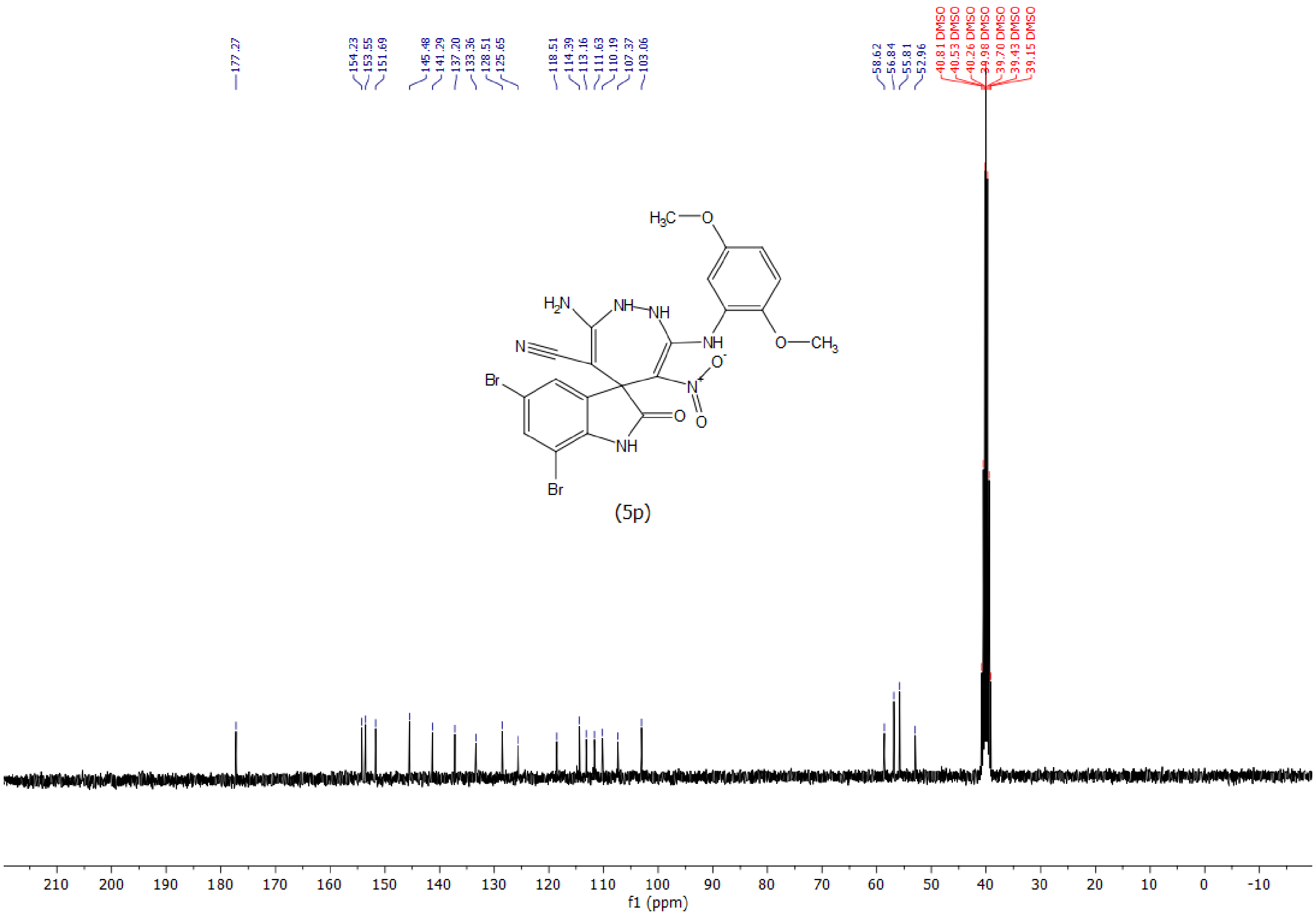
177.27
154.23
153.55
151.69
145.48
141.29
137.20
133.36
128.51
125.65
118.51
114.39
113.16
111.63
110.19
107.37
103.06
58.62
56.84
55.81
52.96
40.81 DMSO
40.53 DMSO
40.26 DMSO
39.98 DMSO
39.70 DMSO
39.43 DMSO
39.15 DMSO
H3C—O
H2N
NH
NH
NH
O—CH3
N
Br
O
NH
Br
(5p)
210 200 190 180 170 160 150 140 130 120 110 100 90 80 70 60 50 40 30 20 10 0 -10
f1 (ppm)

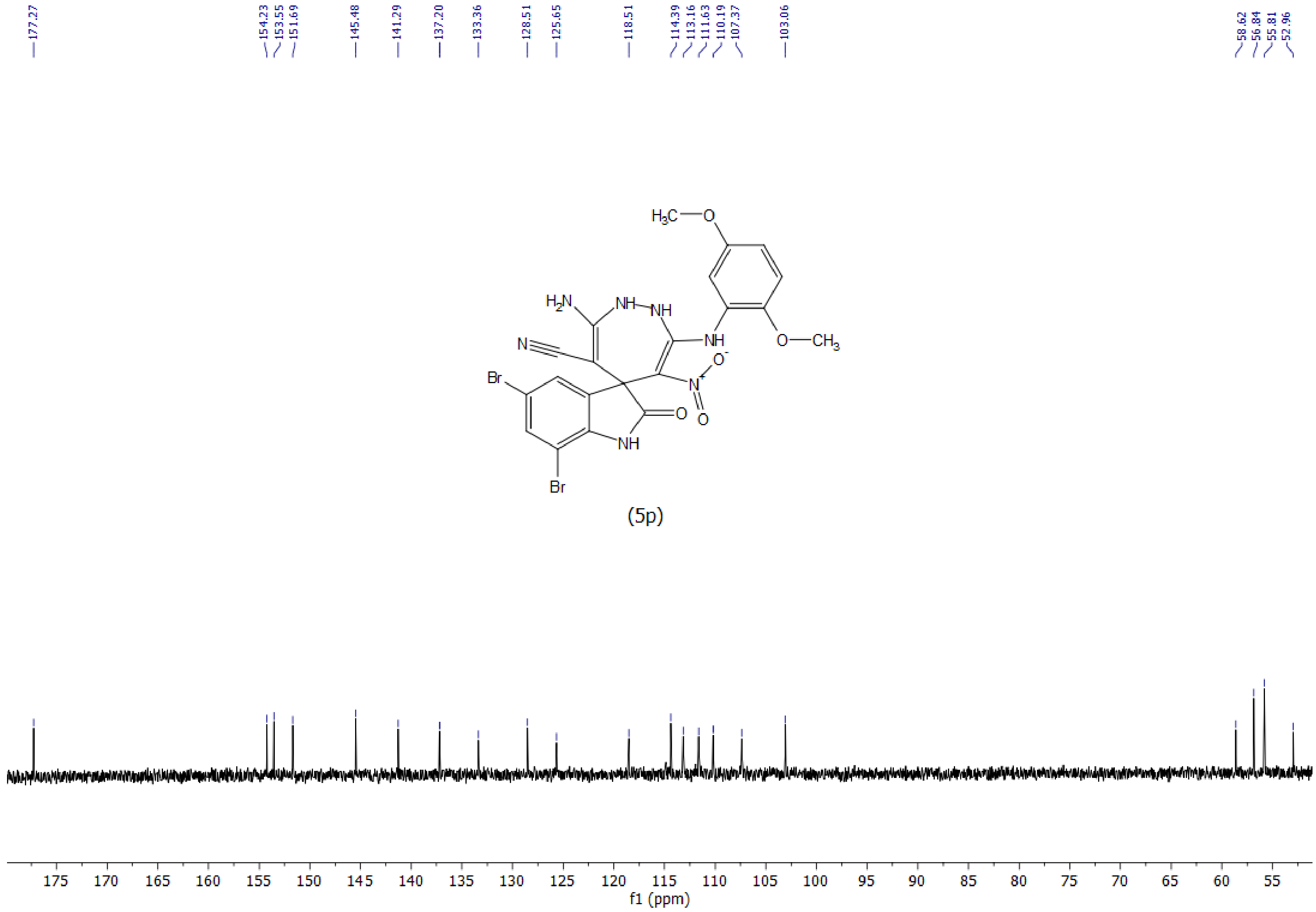
177.27
154.23
153.55
151.69
145.48
141.29
137.20
133.36
128.51
125.65
118.51
114.39
113.16
111.63
110.19
107.37
103.06
58.62
56.84
55.81
52.96
H3C
O
H2N
NH
NH
NH
O-
O
CH3
N
N+
Br
O
NH
Br
(5p)
f1 (ppm)

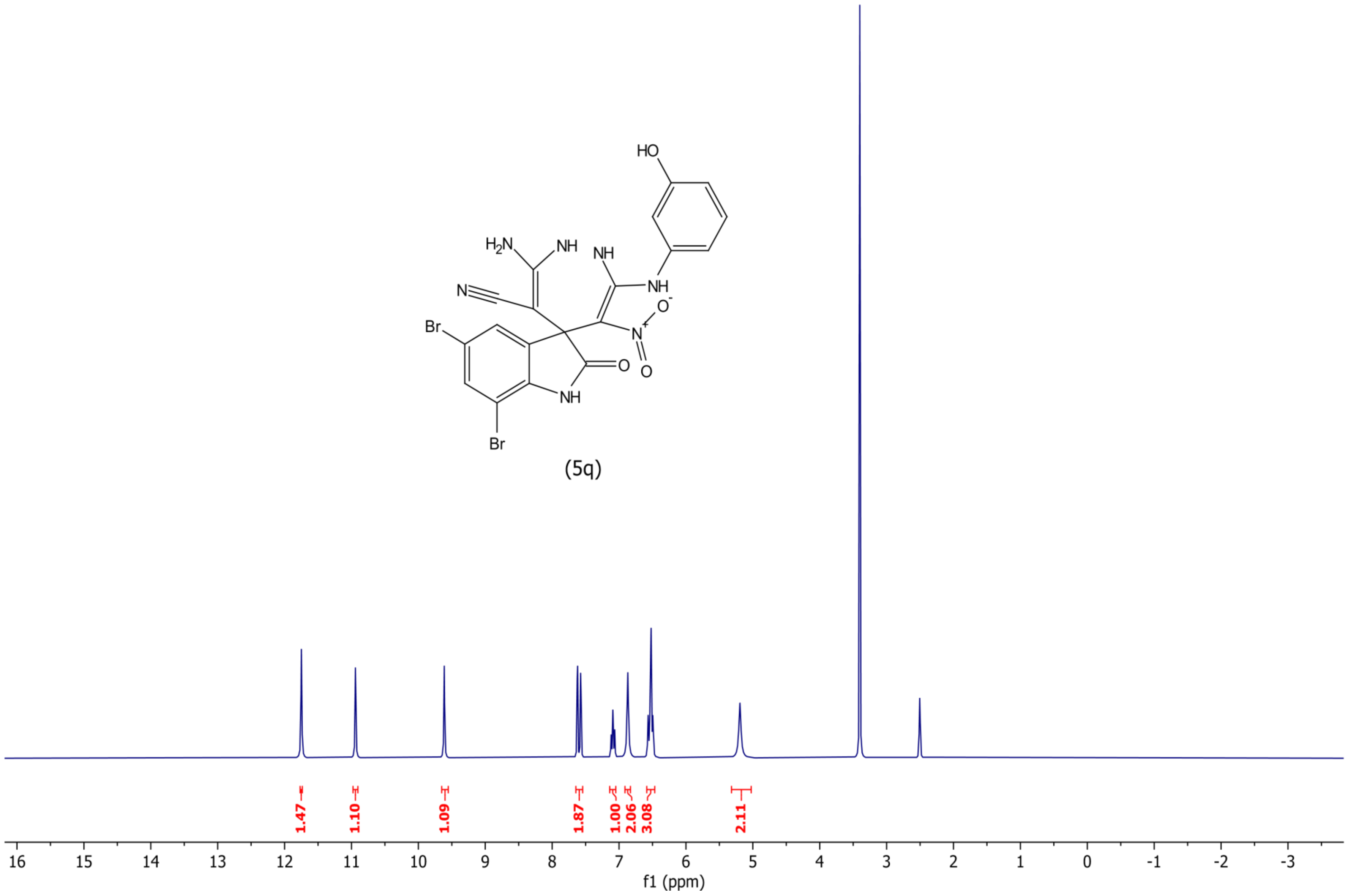
HO
H_2N
NH
NH
N
NH
O
Br
N
O
O
NH
Br
(5q)
1.47
1.10
1.09
1.87
1.00
2.06
3.08
2.11
16
15
14
13
12
11
10
9
8
7
6
5
4
3
2
1
0
-1
-2
-3
f1 (ppm)

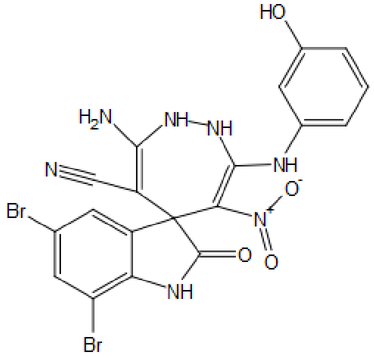
HO
H_2N
NH
NH
N
NH
O
N
O
Br
O
NH
Br

(5q)

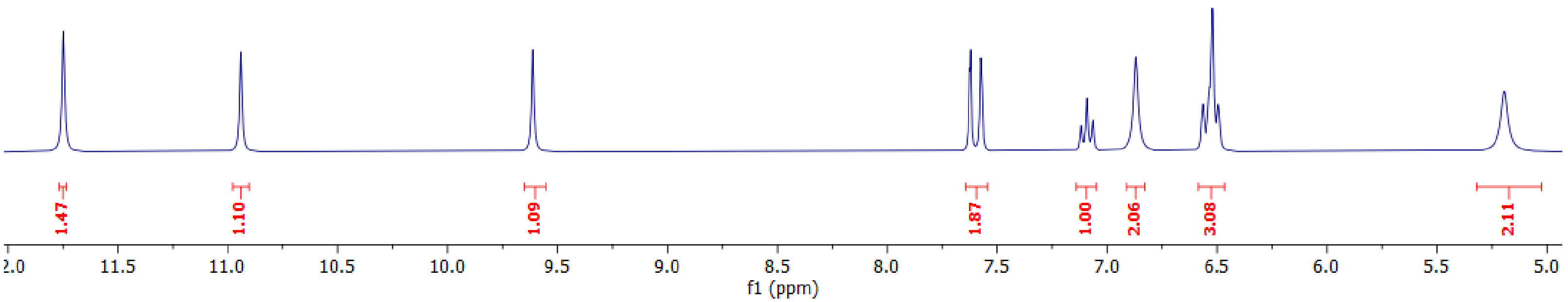
1.47
1.10
1.09
1.87
1.00
2.06
3.08
2.11
12.0
11.5
11.0
10.5
10.0
9.5
9.0
8.5
8.0
7.5
7.0
6.5
6.0
5.5
5.0
f1 (ppm)

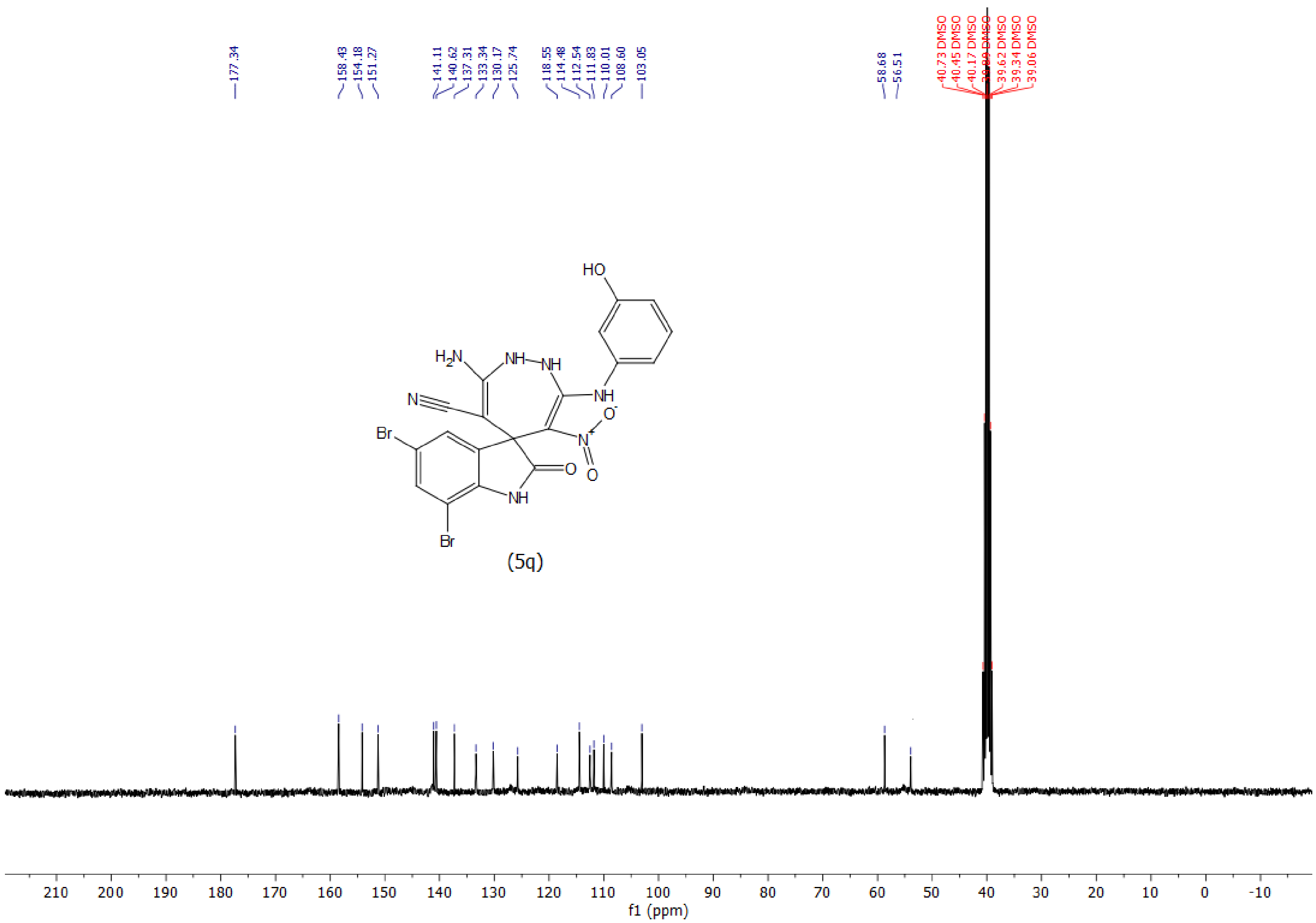
177.34
158.43
154.18
151.27
141.11
140.62
137.31
133.34
130.17
125.74
118.55
114.48
112.54
111.83
110.01
108.60
103.05
58.68
56.51
40.73 DMSO
40.45 DMSO
40.17 DMSO
39.89 DMSO
39.62 DMSO
39.34 DMSO
39.06 DMSO
HO
H2N
NH
NH
N
NH
O-
N+
O
Br
O
NH
Br
(5q)
210 200 190 180 170 160 150 140 130 120 110 100 90 80 70 60 50 40 30 20 10 0 -10
f1 (ppm)

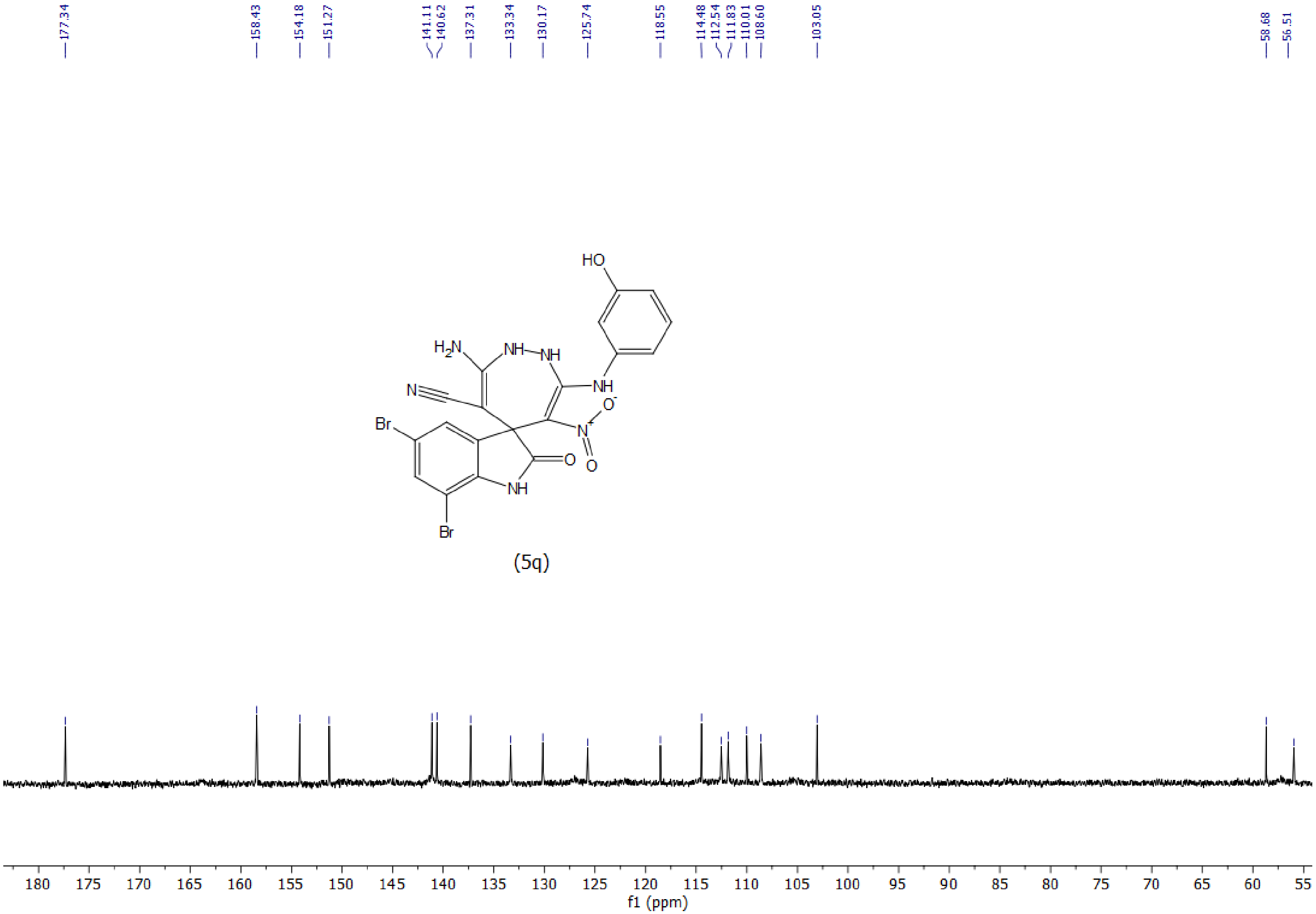
177.34
158.43
154.18
151.27
141.11
140.62
137.31
133.34
130.17
125.74
118.55
114.48
112.54
111.83
110.01
108.60
103.05
58.68
56.51
HO
H_2N
NH
NH
NH
N
O^-
N^+
O
O
Br
Br
NH
180 175 170 165 160 155 150 145 140 135 130 125 120 115 110 105 100 95 90 85 80 75 70 65 60 55
f1 (ppm)

(5q)

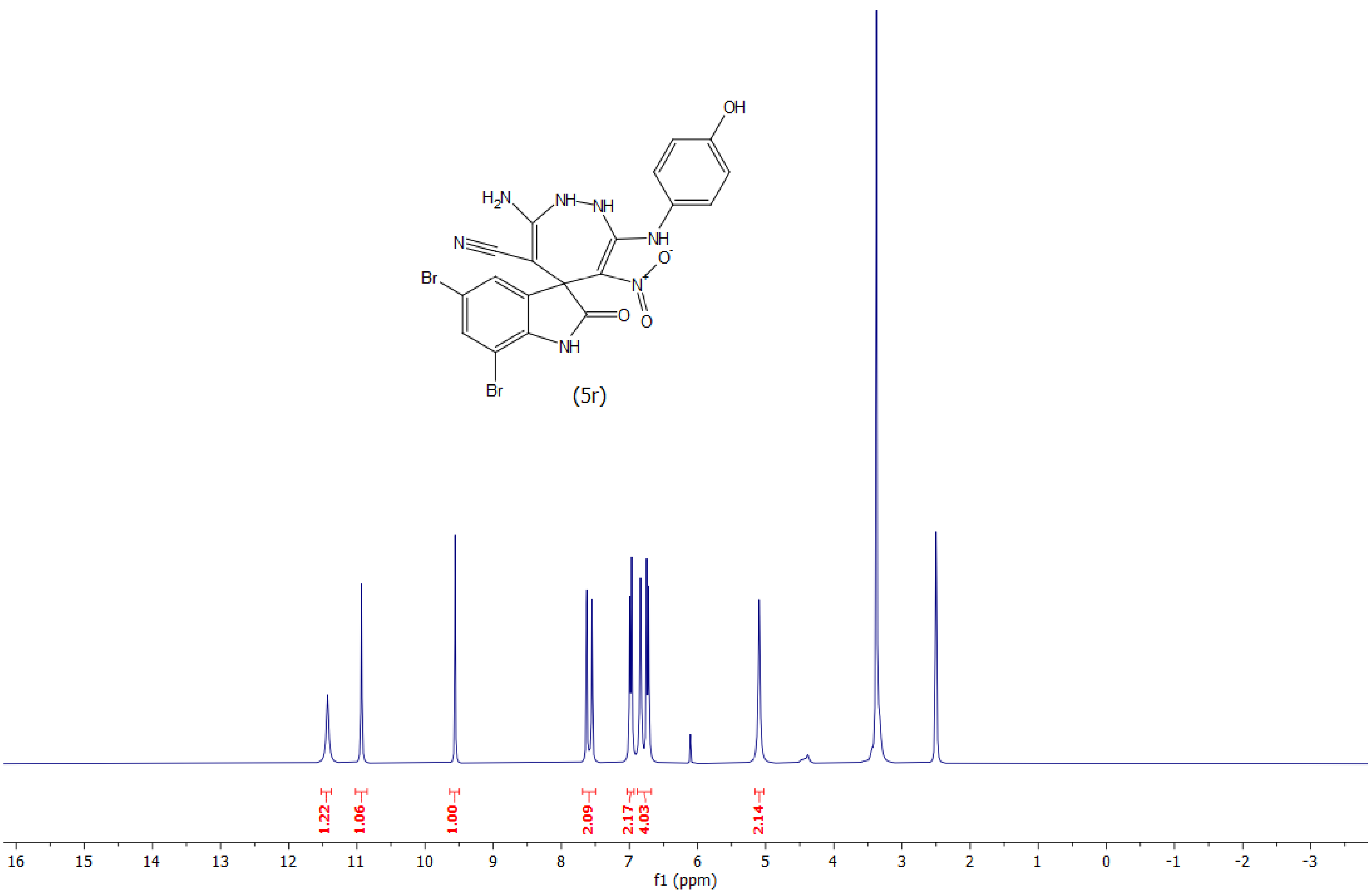

(5r)
H2N
NH
NH
NH
N
O
O
Br
Br
NH
O
OH
1.22
1.06
1.00
2.09
2.17
4.03
2.14
16 15 14 13 12 11 10 9 8 7 6 5 4 3 2 1 0 -1 -2 -3
f1 (ppm)

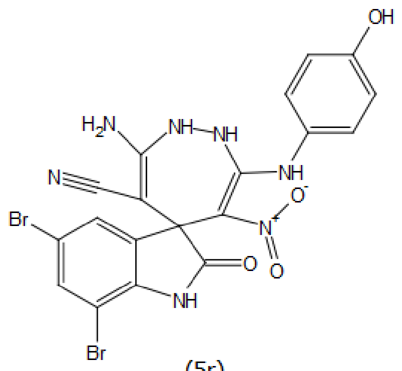

OH
H_2N
NH
NH
NH
N
Br
O^-
N^+
O
O
NH
Br

(5r)

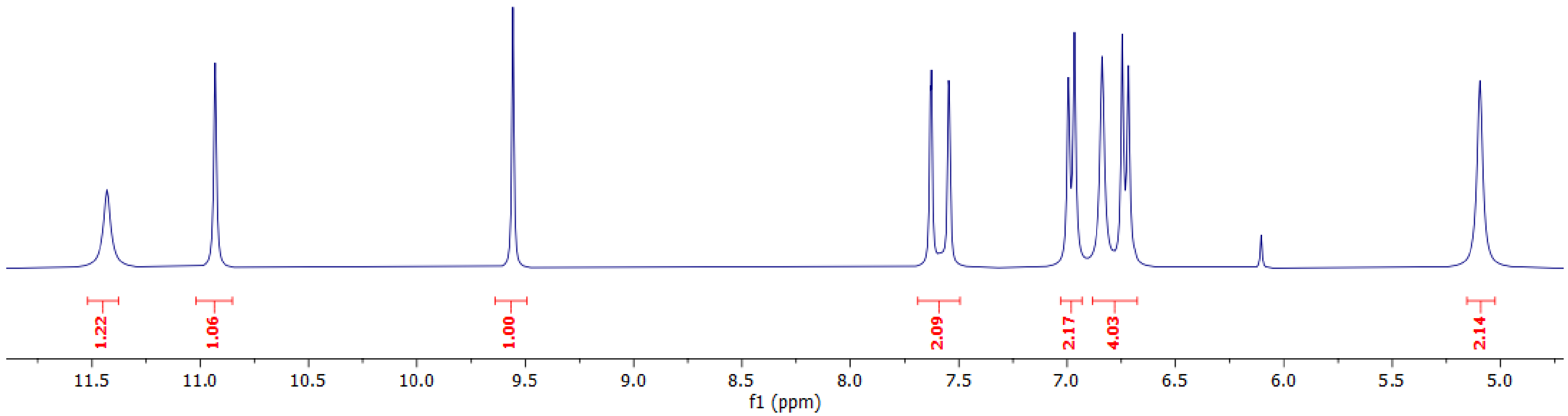

1.22
1.06
1.00
2.09
2.17
4.03
2.14
11.5
11.0
10.5
10.0
9.5
9.0
8.5
8.0
7.5
7.0
6.5
6.0
5.5
5.0
f1 (ppm)

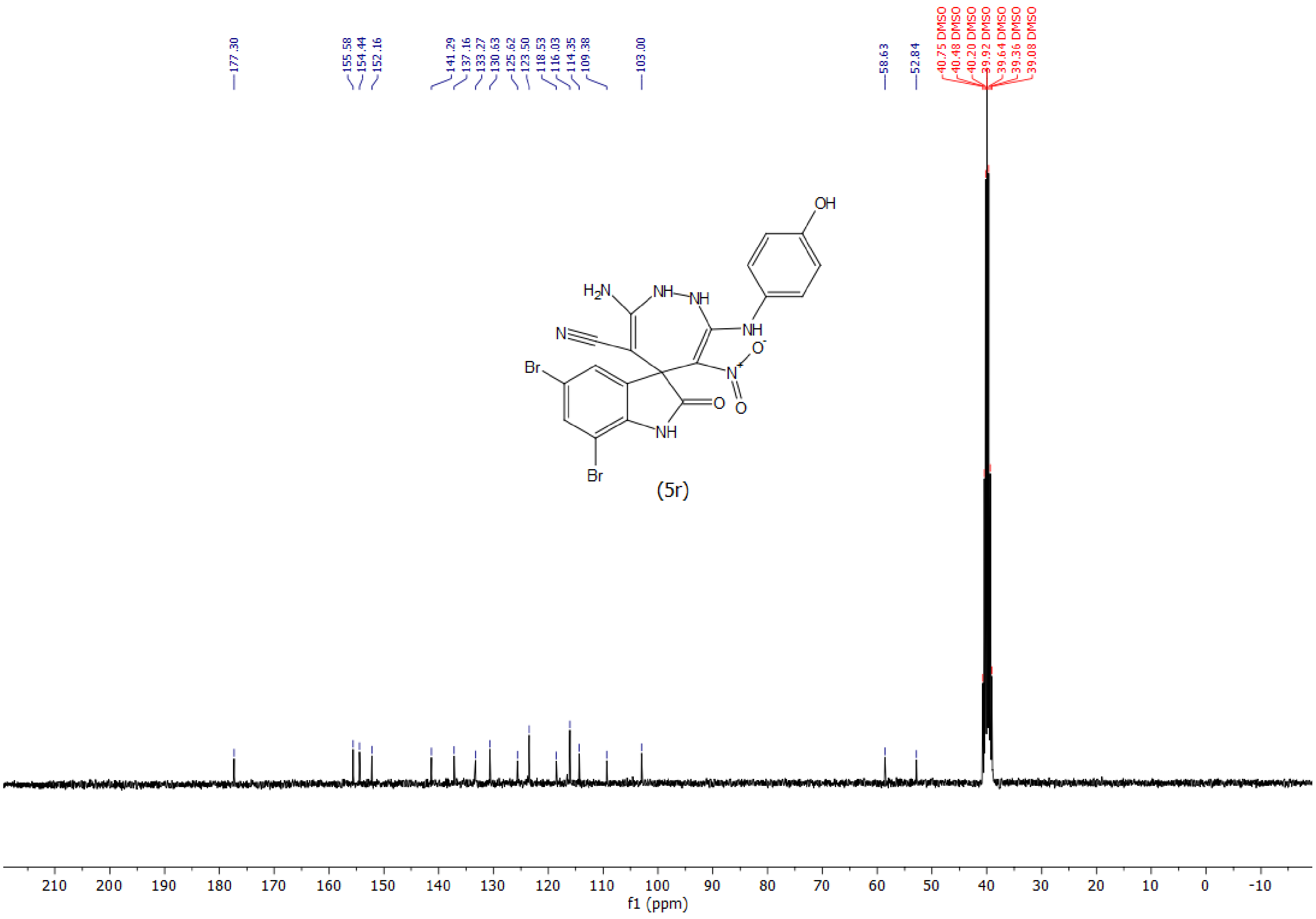
177.30
155.58
154.44
152.16
141.29
137.16
133.27
130.63
125.62
123.50
118.53
116.03
114.35
109.38
103.00
58.63
52.84
40.75 DMSO
40.48 DMSO
40.20 DMSO
39.92 DMSO
39.64 DMSO
39.36 DMSO
39.08 DMSO
OH
H_2N
NH
NH
NH
N
Br
Br
O
O^-
N^+
O
NH
(5r)
210 200 190 180 170 160 150 140 130 120 110 100 90 80 70 60 50 40 30 20 10 0 -10
f1 (ppm)

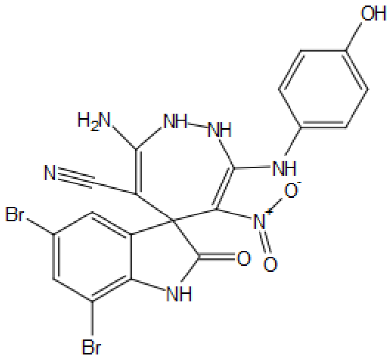
OH
H2N
NH
NH
NH
N
O-
N+
O
Br
O
NH
Br

(5r)

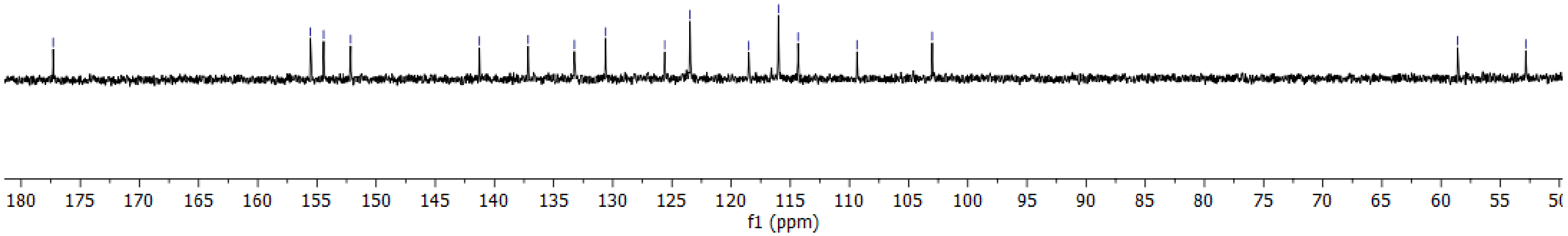
177.30
155.58
154.44
152.16
141.29
137.16
133.27
130.63
125.62
123.50
118.53
116.03
114.35
109.38
103.00
58.63
52.84
180 175 170 165 160 155 150 145 140 135 130 125 120 115 110 105 100 95 90 85 80 75 70 65 60 55 50
f1 (ppm)

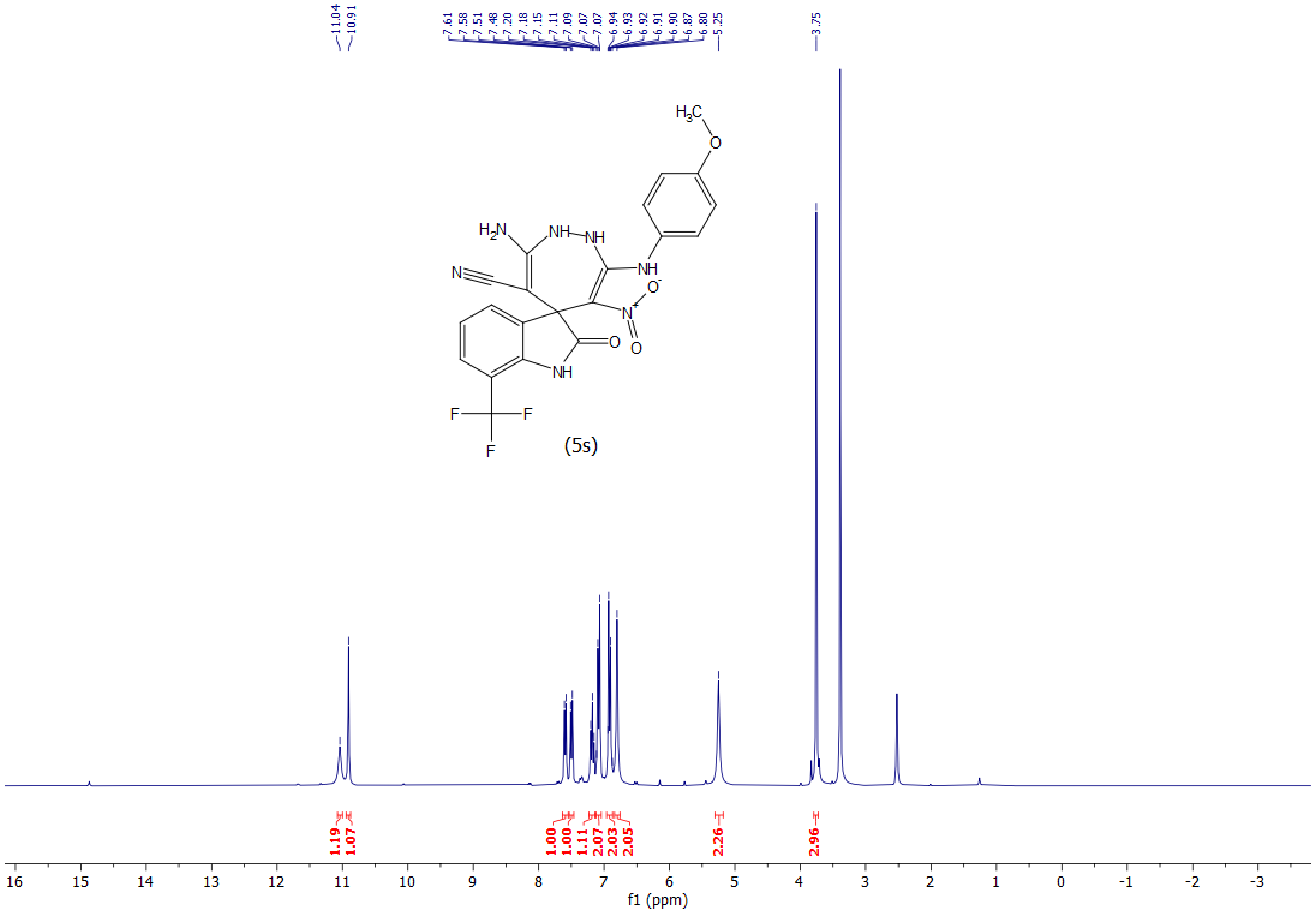
11.04
10.91
7.61
7.58
7.51
7.48
7.20
7.18
7.15
7.11
7.09
7.07
7.07
6.94
6.93
6.92
6.91
6.90
6.87
6.80
5.25
3.75
H3C
O
H2N
NH
NH
NH
N
O-
N+
O
O
NH
F
F
F
(5s)
1.19
1.07
1.00
1.00
1.11
2.07
2.03
2.05
2.26
2.96
16
15
14
13
12
11
10
9
8
7
6
5
4
3
2
1
0
-1
-2
-3
f1 (ppm)

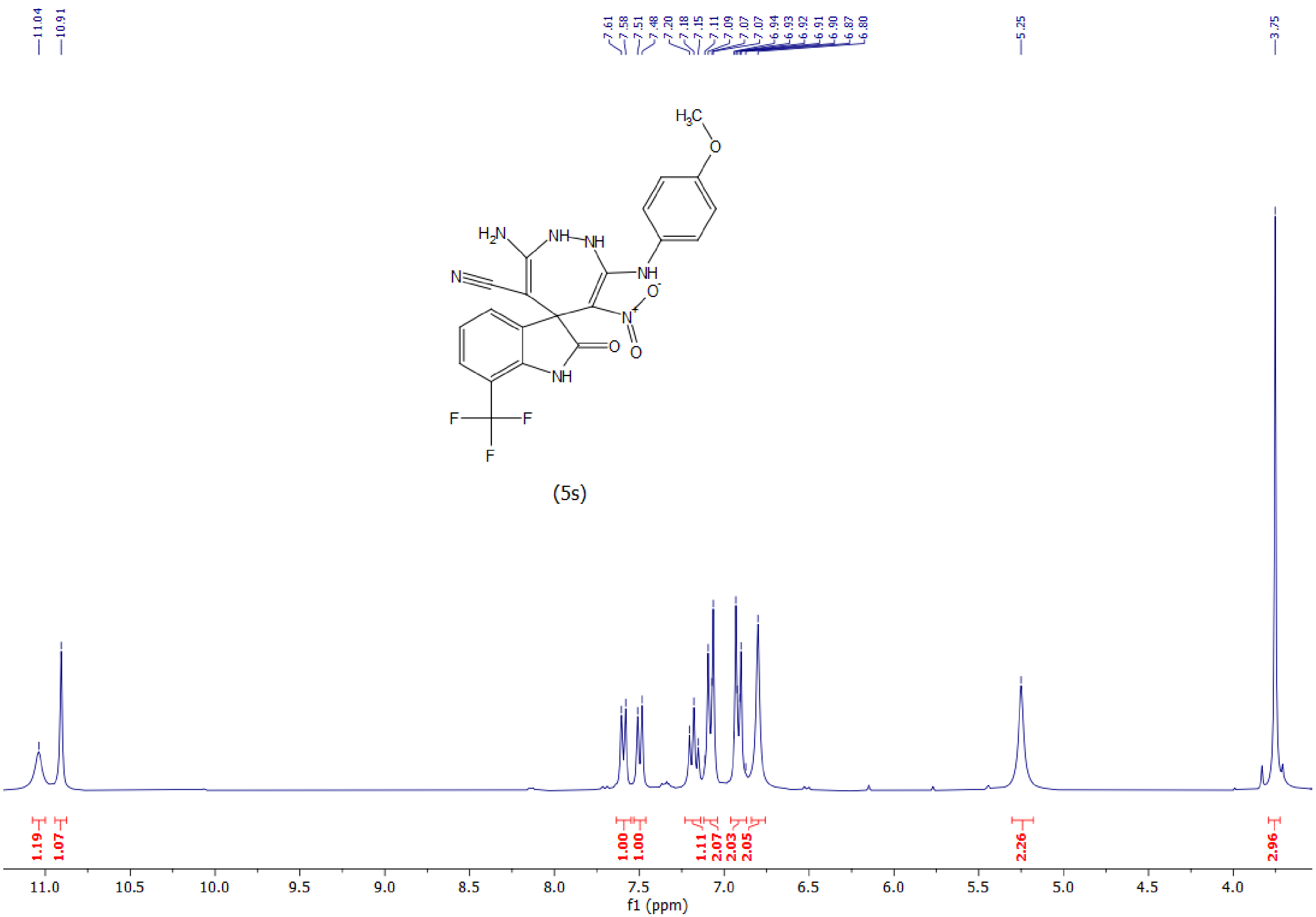
11.04
10.91
7.61
7.58
7.51
7.48
7.20
7.18
7.15
7.11
7.09
7.07
7.07
6.94
6.93
6.92
6.91
6.90
6.87
6.80
5.25
3.75
H_3C
O
H_2N
NH
NH
NH
N
O^-
N^+
O
O
NH
F
F
F
1.19
1.07
1.00
1.00
1.11
2.07
2.03
2.05
2.26
2.96
11.0
10.5
10.0
9.5
9.0
8.5
8.0
7.5
7.0
6.5
6.0
5.5
5.0
4.5
4.0
f1 (ppm)

(5s)

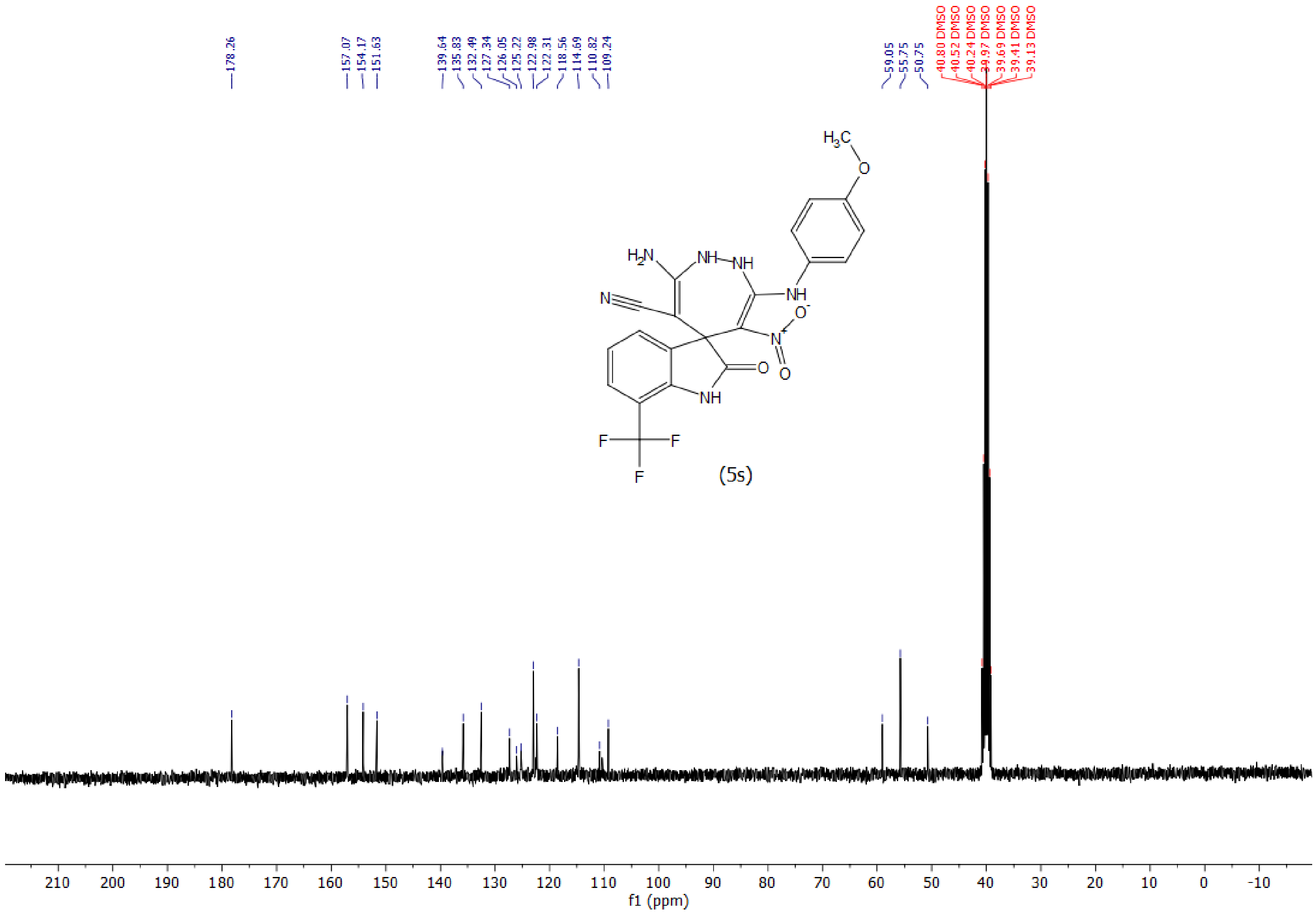
178.26
157.07
154.17
151.63
139.64
135.83
132.49
127.34
126.05
125.22
122.98
122.31
118.56
114.69
110.82
109.24
59.05
55.75
50.75
40.80 DMSO
40.52 DMSO
40.24 DMSO
39.97 DMSO
39.69 DMSO
39.41 DMSO
39.13 DMSO
f1 (ppm)

(5s)

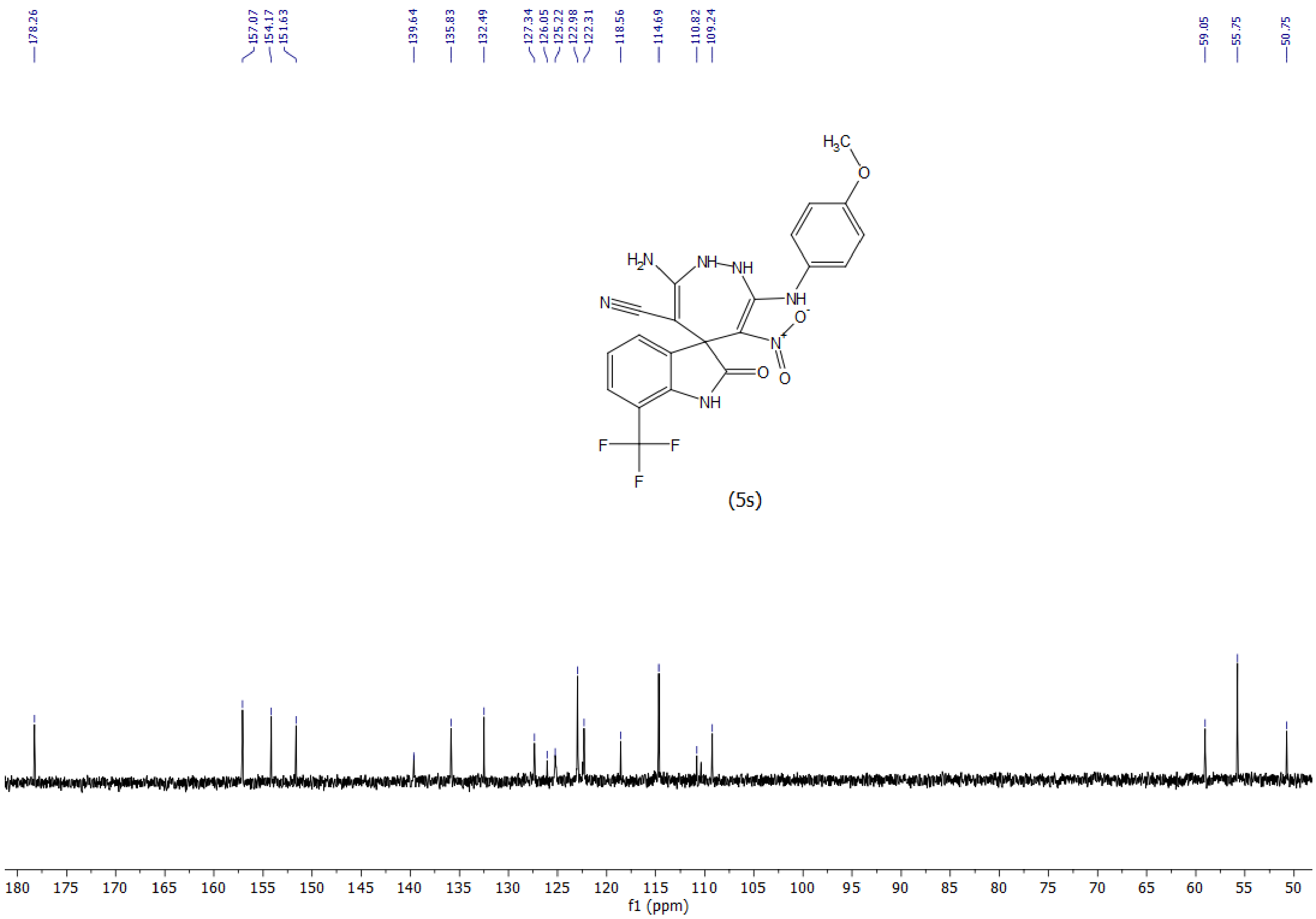
178.26
157.07
154.17
151.63
139.64
135.83
132.49
127.34
126.05
125.22
122.98
122.31
118.56
114.69
110.82
109.24
59.05
55.75
50.75
H3C
O
H2N
NH
NH
NH
N
O-
N+
O
O
NH
F
F
F
(5s)
180 175 170 165 160 155 150 145 140 135 130 125 120 115 110 105 100 95 90 85 80 75 70 65 60 55 50
f1 (ppm)

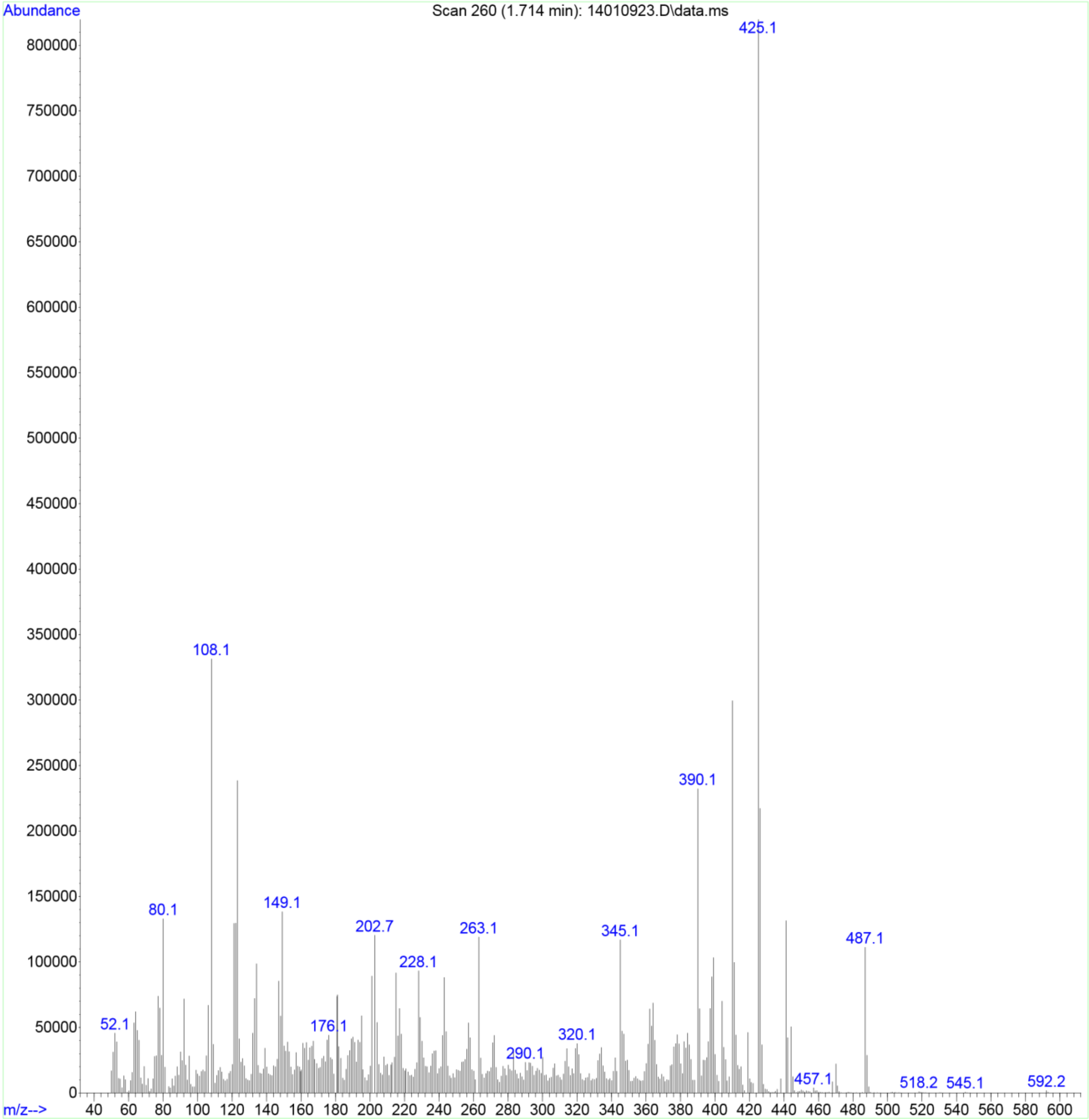

$C_{21}H_{16}F_3N_7O_4$ **(5s)**

(487)

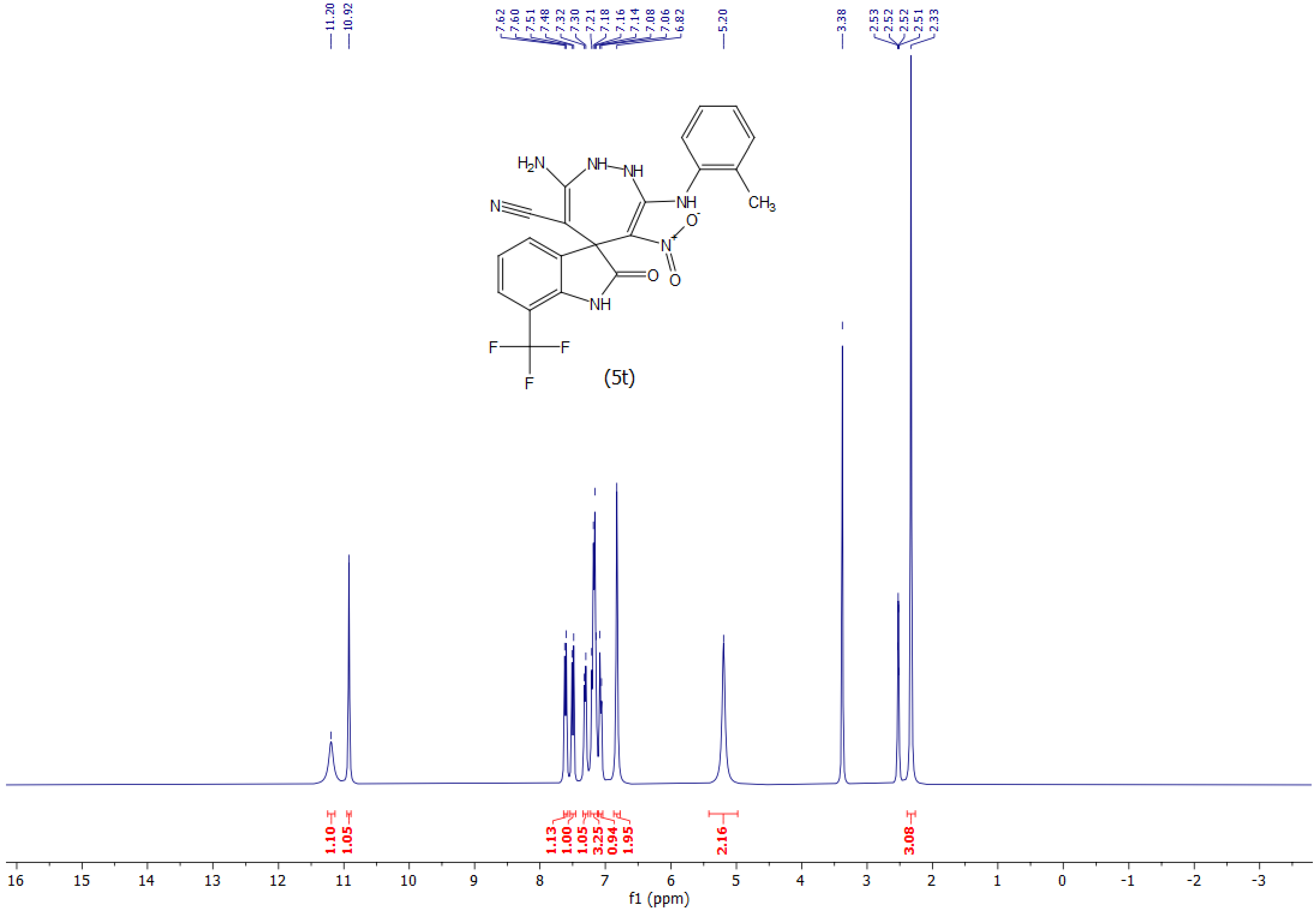
11.20
10.92
7.62
7.60
7.51
7.48
7.32
7.30
7.21
7.18
7.16
7.14
7.08
7.06
6.82
5.20
3.38
2.53
2.52
2.52
2.51
2.33
(5t)
1.10
1.05
1.13
1.00
1.05
3.25
0.94
1.95
2.16
3.08
16
15
14
13
12
11
10
9
8
7
6
5
4
3
2
1
0
-1
-2
-3
f1 (ppm)

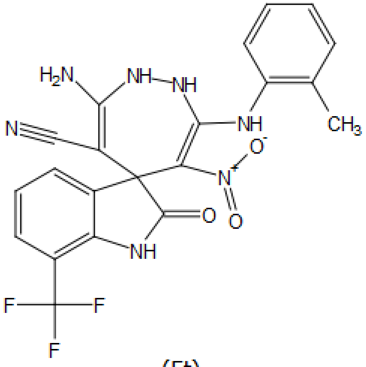

(5t)

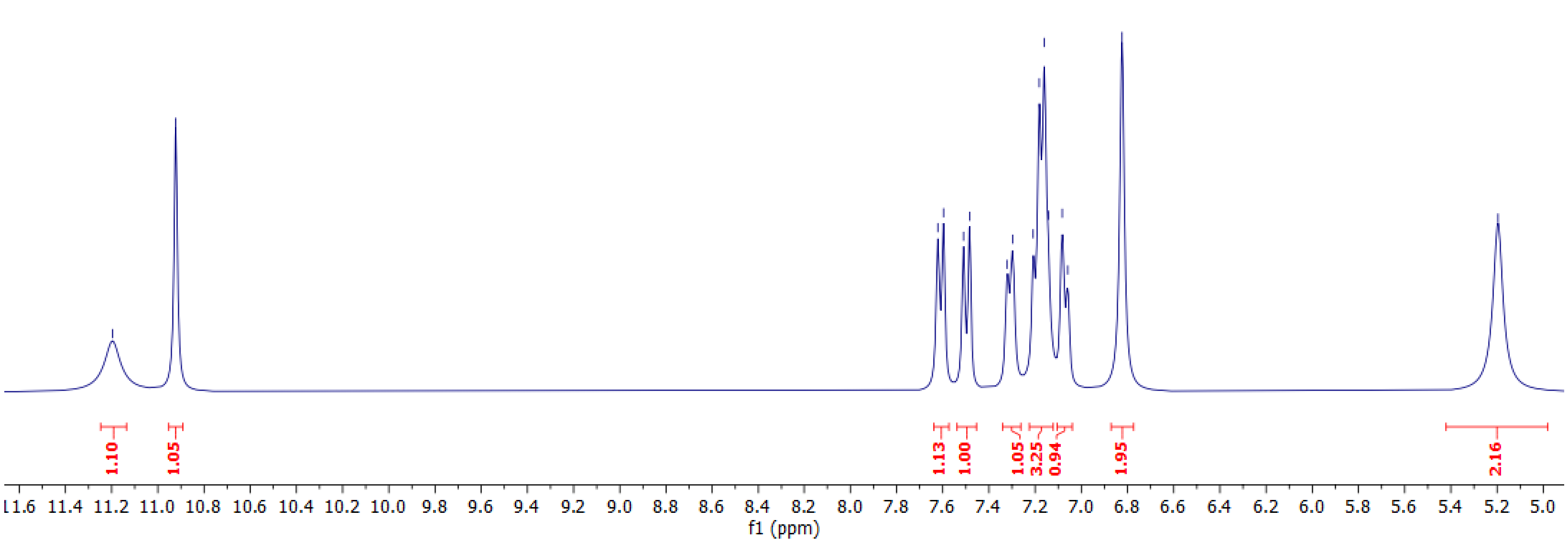

11.20
10.92
7.62
7.60
7.51
7.48
7.32
7.30
7.21
7.18
7.16
7.14
7.08
7.06
6.82
5.20
1.10
1.05
1.13
1.00
1.05
3.25
0.94
1.95
2.16
11.6 11.4 11.2 11.0 10.8 10.6 10.4 10.2 10.0 9.8 9.6 9.4 9.2 9.0 8.8 8.6 8.4 8.2 8.0 7.8 7.6 7.4 7.2 7.0 6.8 6.6 6.4 6.2 6.0 5.8 5.6 5.4 5.2 5.0
f1 (ppm)

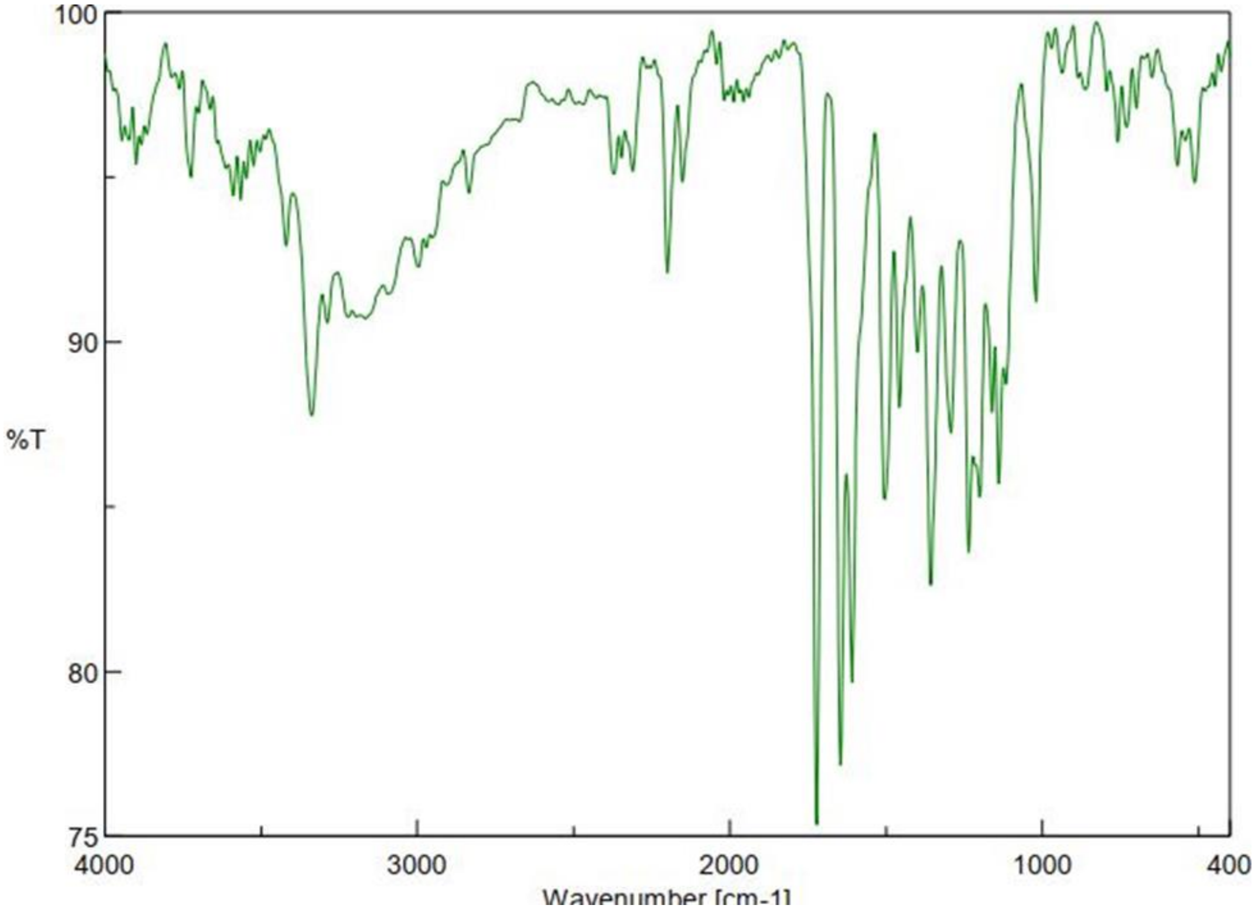

(5t)

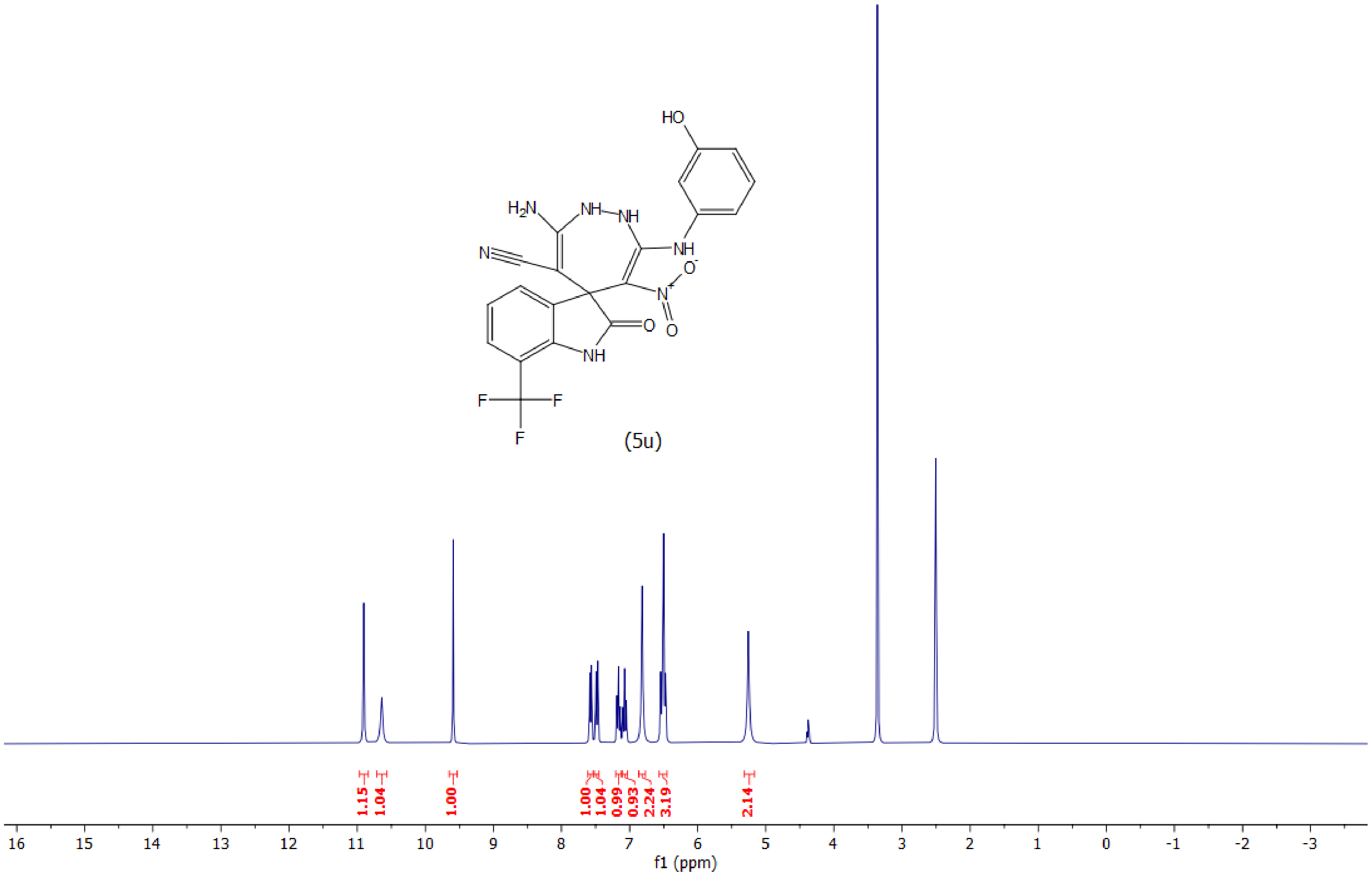
H2N
NH
NH
NH
N
O
N
O
O
HO
NH
F
F
F
(5u)
1.15
1.04
1.00
1.00
1.04
0.99
0.93
2.24
3.19
2.14
16
15
14
13
12
11
10
9
8
7
6
5
4
3
2
1
0
-1
-2
-3
f1 (ppm)

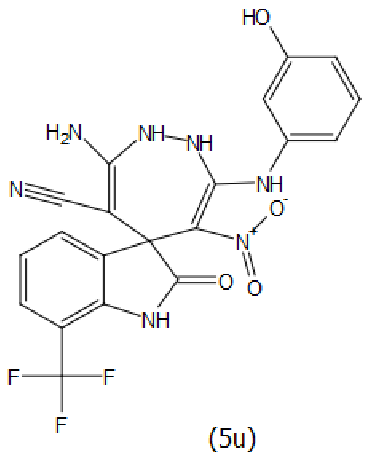
HO
H_2N
NH
NH
N
NH
O^-
N^+
O
O
NH
F
F
F

(5u)

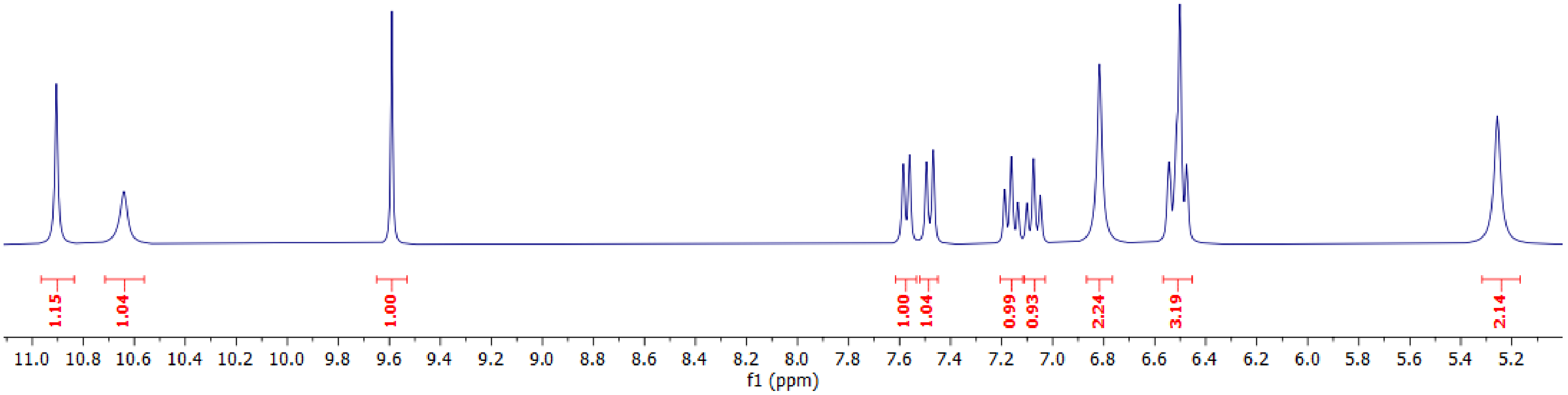
1.15
1.04
1.00
1.00
1.04
0.99
0.93
2.24
3.19
2.14
11.0 10.8 10.6 10.4 10.2 10.0 9.8 9.6 9.4 9.2 9.0 8.8 8.6 8.4 8.2 8.0 7.8 7.6 7.4 7.2 7.0 6.8 6.6 6.4 6.2 6.0 5.8 5.6 5.4 5.2
f1 (ppm)

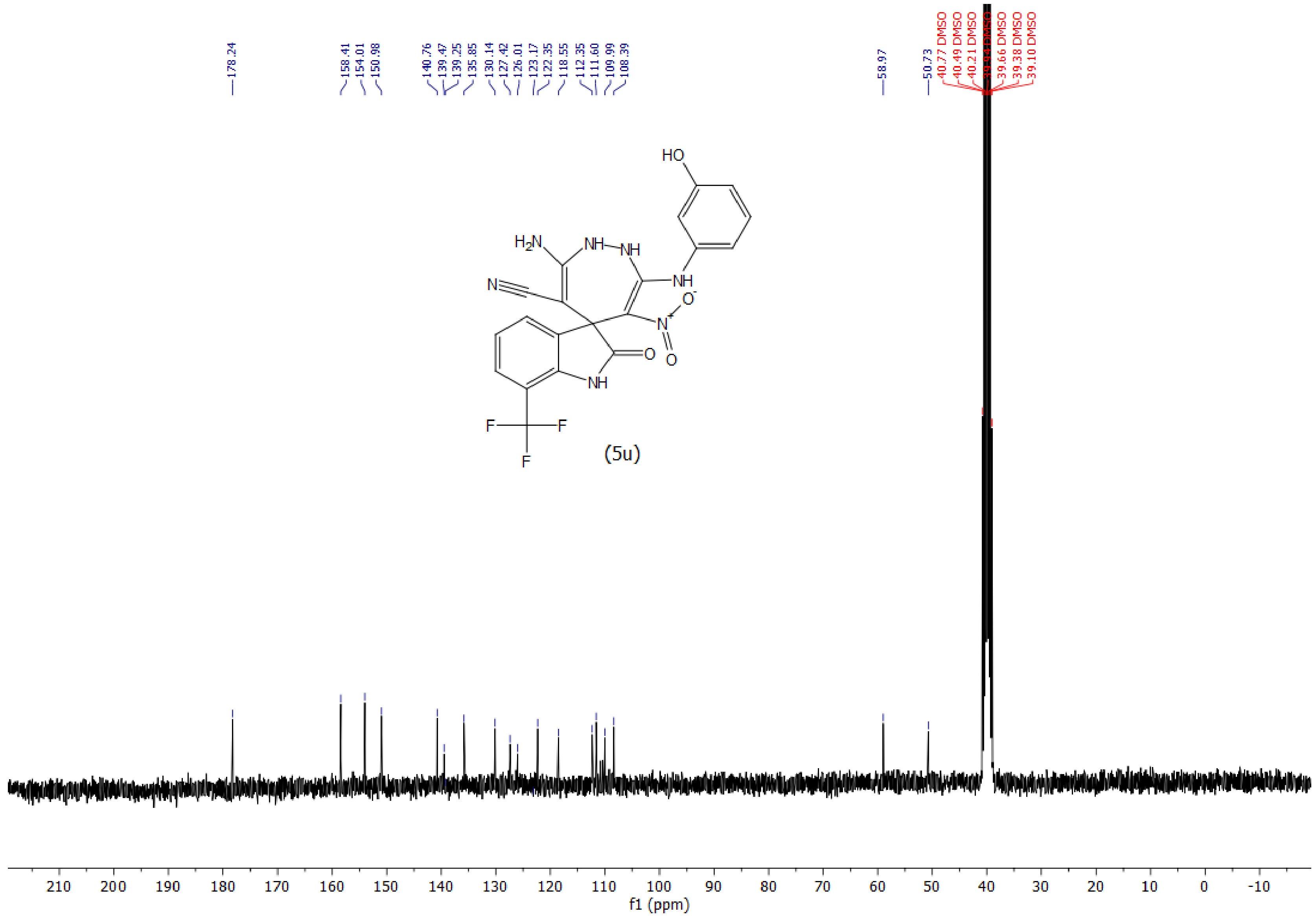
178.24
158.41
154.01
150.98
140.76
139.47
139.25
135.85
130.14
127.42
126.01
123.17
122.35
118.55
112.35
111.60
109.99
108.39
58.97
50.73
40.77 DMSO
40.49 DMSO
40.21 DMSO
39.94 DMSO
39.66 DMSO
39.38 DMSO
39.10 DMSO
HO
H_2N
NH
NH
NH
N
O^-
N^+
O
O
NH
F
F
F
(5u)
210 200 190 180 170 160 150 140 130 120 110 100 90 80 70 60 50 40 30 20 10 0 -10
f1 (ppm)

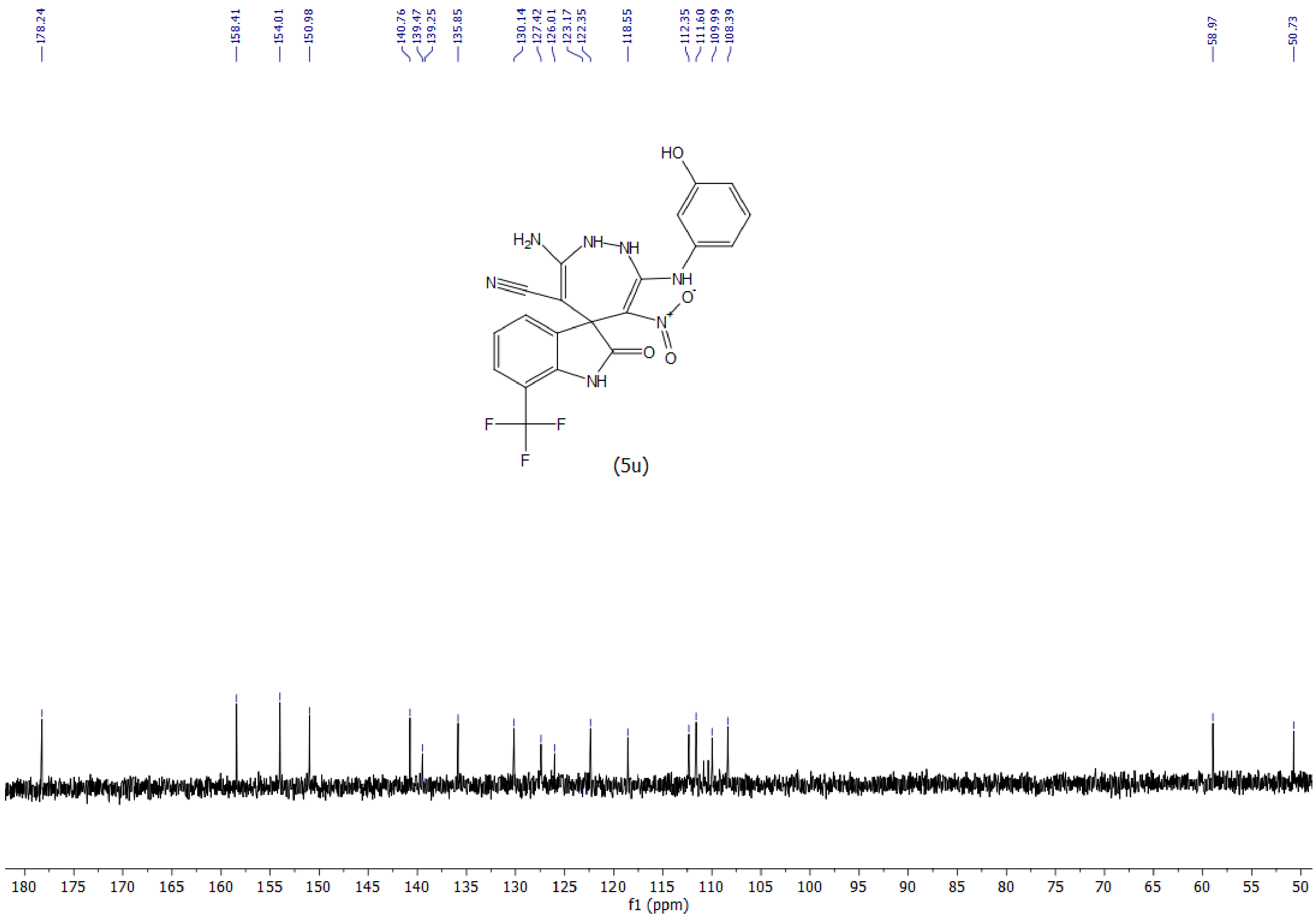
178.24
158.41
154.01
150.98
140.76
139.47
139.25
135.85
130.14
127.42
126.01
123.17
122.35
118.55
112.35
111.60
109.99
108.39
58.97
50.73
HO
H_2N
NH
NH
NH
N
O^-
N^+
O
O
NH
F
F
F
180
175
170
165
160
155
150
145
140
135
130
125
120
115
110
105
100
95
90
85
80
75
70
65
60
55
50
f1 (ppm)

(5u)

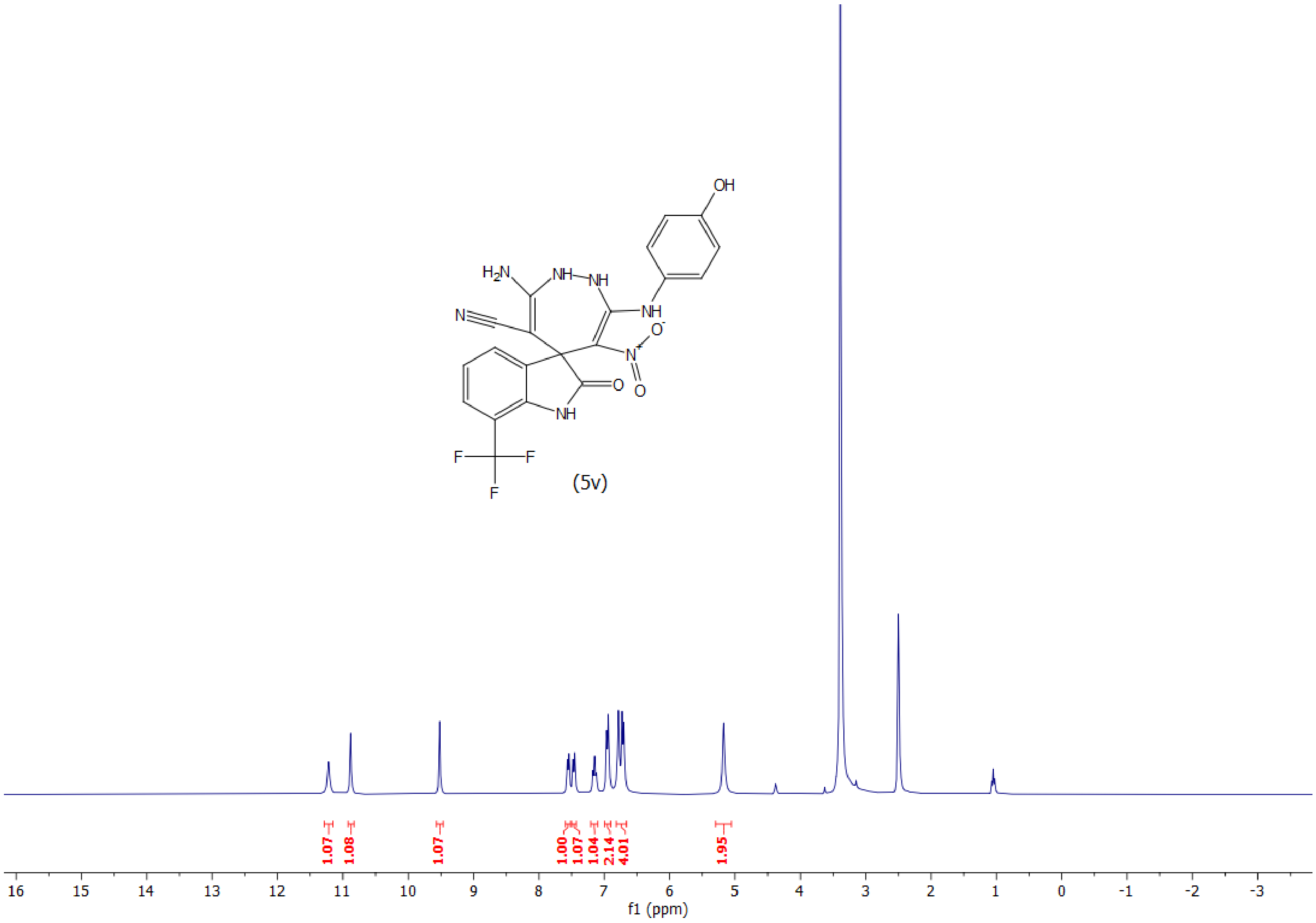
H2N
NH
NH
NH
N
N+
O-
O
O
NH
OH
F
F
F
1.07
1.08
1.07
1.00
1.07
1.04
2.14
4.01
1.95
16 15 14 13 12 11 10 9 8 7 6 5 4 3 2 1 0 -1 -2 -3
f1 (ppm)

(5v)

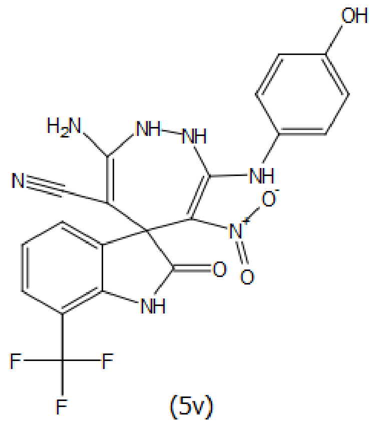
OH
H2N
NH
NH
NH
N
O-
N+
O
O
NH
F
F
F

(5v)

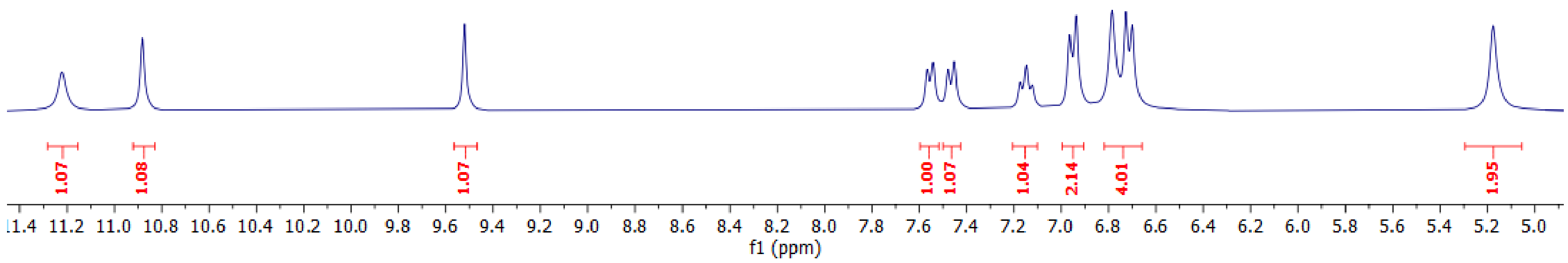
1.07
1.08
1.07
1.00
1.07
1.04
2.14
4.01
1.95
11.4 11.2 11.0 10.8 10.6 10.4 10.2 10.0 9.8 9.6 9.4 9.2 9.0 8.8 8.6 8.4 8.2 8.0 7.8 7.6 7.4 7.2 7.0 6.8 6.6 6.4 6.2 6.0 5.8 5.6 5.4 5.2 5.0
f1 (ppm)

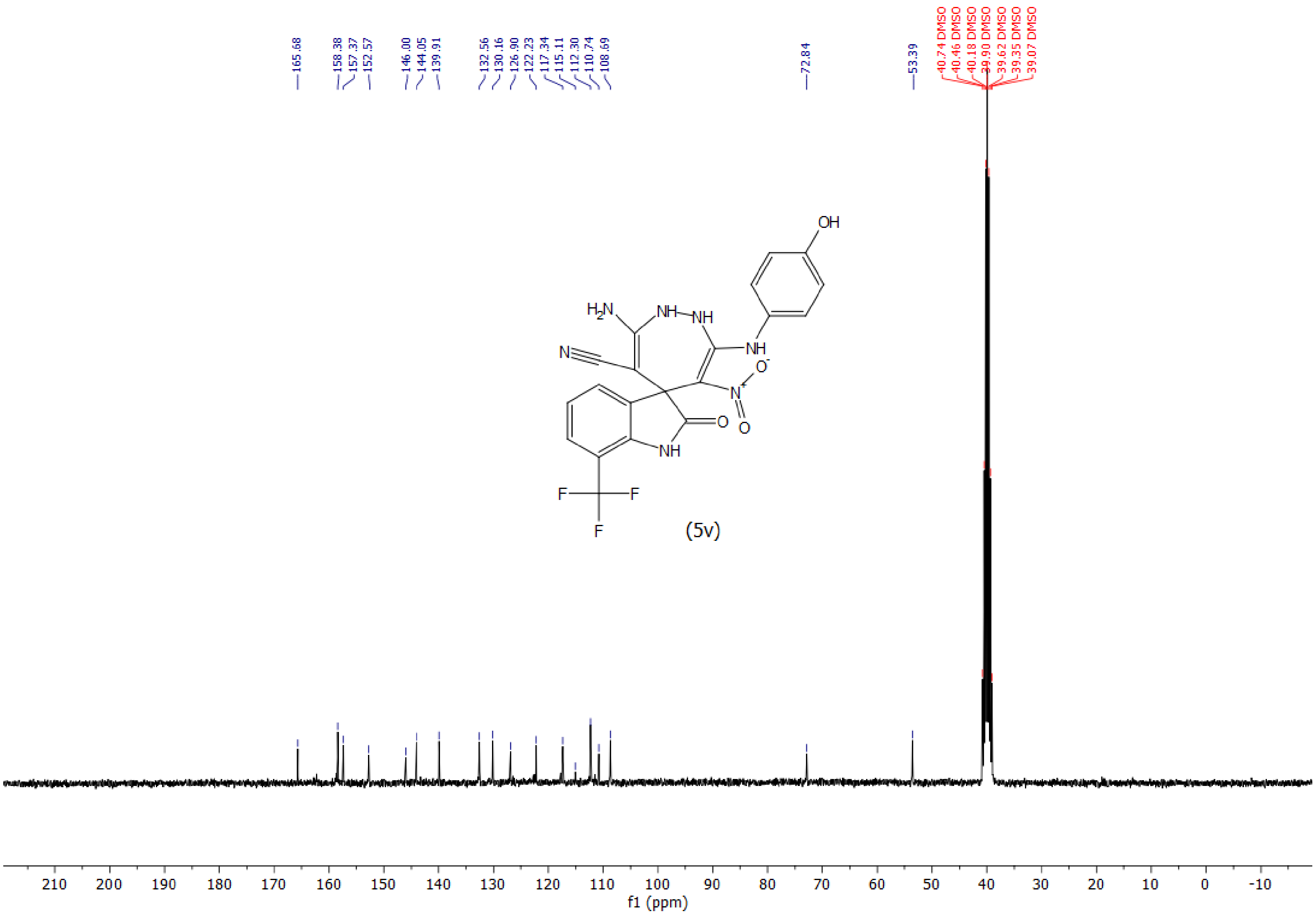
165.68
158.38
157.37
152.57
146.00
144.05
139.91
132.56
130.16
126.90
122.23
117.34
115.11
112.30
110.74
108.69
72.84
53.39
40.74 DMSO
40.46 DMSO
40.18 DMSO
39.90 DMSO
39.62 DMSO
39.35 DMSO
39.07 DMSO
OH
H_2N
NH
NH
NH
N
O
N^+
O^-
O
NH
F
F
F
(5v)
210 200 190 180 170 160 150 140 130 120 110 100 90 80 70 60 50 40 30 20 10 0 -10
f1 (ppm)

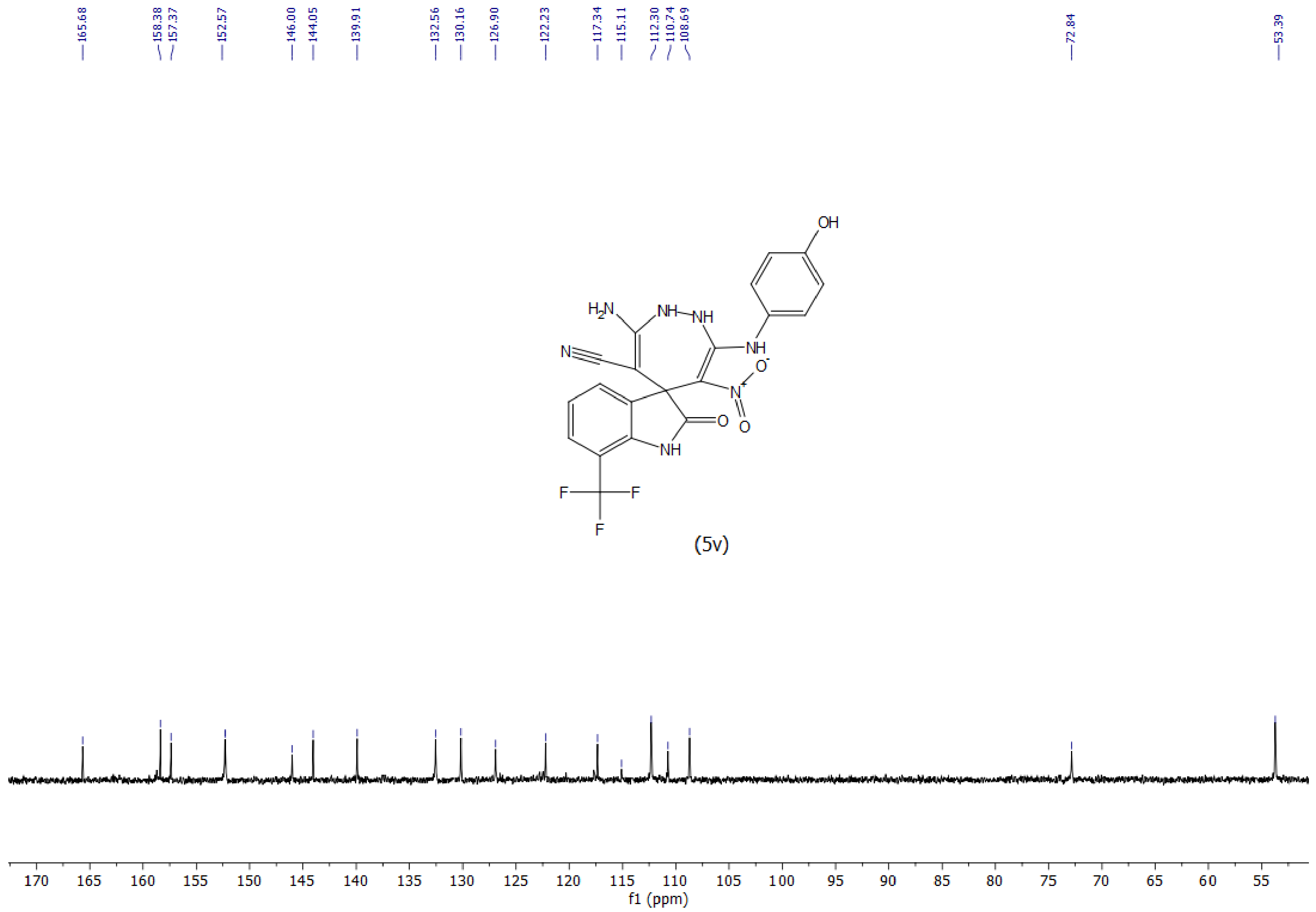
165.68
158.38
157.37
152.57
146.00
144.05
139.91
132.56
130.16
126.90
122.23
117.34
115.11
112.30
110.74
108.69
72.84
53.39
OH
H_2N
NH
NH
NH
N
O^-
N^+
O
O
NH
F
F
F
170
165
160
155
150
145
140
135
130
125
120
115
110
105
100
95
90
85
80
75
70
65
60
55
f1 (ppm)

(5v)
